# Proteomic analysis links alterations of bioenergetics, mitochondria-ER interactions and proteostasis in hippocampal astrocytes from 3xTg-AD mice

**DOI:** 10.1038/s41419-020-02911-1

**Published:** 2020-08-18

**Authors:** Giulia Dematteis, Gabrielė Vydmantaitė, Federico Alessandro Ruffinatti, Malak Chahin, Serena Farruggio, Elettra Barberis, Eleonora Ferrari, Emilio Marengo, Carla Distasi, Ramunė Morkūnienė, Armando A. Genazzani, Mariagrazia Grilli, Elena Grossini, Marco Corazzari, Marcello Manfredi, Dmitry Lim, Aistė Jekabsone, Laura Tapella

**Affiliations:** 1 Department of Pharmaceutical Sciences, Università degli Studi del Piemonte Orientale, Novara, Italy; 2 Institute of Pharmaceutical Technologies, Faculty of Pharmacy, Medical Academy, Lithuanian University of Health Sciences, Kaunas, Lithuania; 3 Department of Translational Medicine, University of Piemonte Orientale, Novara, Italy; 4 Center for Translational Research on Autoimmune and Allergic Diseases (CAAD), University of Piemonte Orientale, Novara, Italy; 5 Department of Health Science, University of Piemonte Orientale, Novara, Italy; 6 DiSIT, University of Piemonte Orientale, Alessandria, Italy; 7 Department of Drug Chemistry, Faculty of Pharmacy, Medical Academy, Lithuanian University of Health Sciences, Kaunas, Lithuania; 8 Interdisciplinary Research Center of Autoimmune Diseases (IRCAD), University of Piemonte Orientale, Novara, Italy

**Keywords:** Cellular neuroscience, Alzheimer's disease

## Abstract

The pathogenesis of Alzheimer’s disease (AD), a slowly-developing age-related neurodegenerative disorder, is a result of the action of multiple factors including deregulation of Ca^2+^ homeostasis, mitochondrial dysfunction, and dysproteostasis. Interaction of these factors in astrocytes, principal homeostatic cells in the central nervous system, is still poorly understood. Here we report that in immortalized hippocampal astrocytes from 3xTg-AD mice (3Tg-iAstro cells) bioenergetics is impaired, including reduced glycolysis and mitochondrial oxygen consumption, and increased production of reactive oxygen species. Shotgun proteomics analysis of mitochondria-ER-enriched fraction showed no alterations in the expression of mitochondrial and OxPhos proteins, while those related to the ER functions and protein synthesis were deregulated. Using ER- and mitochondria-targeted aequorin-based Ca^2+^ probe we show that, in 3Tg-iAstro cells, ER was overloaded with Ca^2+^ while Ca^2+^ uptake by mitochondria upon ATP stimulation was reduced. This was accompanied by the increase in short distance (≈8–10 nm) contact area between mitochondria and ER, upregulation of ER-stress/unfolded protein response genes Atf4, Atf6 and Herp, and reduction of global protein synthesis rate. We suggest that familial AD mutations in 3Tg-iAstro cells induce mitochondria-ER interaction changes that deregulate astrocytic bioenergetics, Ca^2+^ homeostasis and proteostasis. These factors may interact, creating a pathogenic loop compromising homeostatic and defensive functions of astroglial cells predisposing neurons to dysfunction.

## Introduction

Alzheimer’s disease (AD) is a devastating age-related neurodegenerative disorder with a complex and slowly-developing pathogenesis^1^. Modern hypotheses, aiming at explanation the mechanisms of AD development, link ageing with destructive effects of Familial Alzheimer Disease (FAD) mutations and β-amyloid (Aβ) burden. Among such hypotheses are the calcium hypothesis of neurodegeneration^2^ and the mitochondrial cascade hypothesis, the latter including also the oxidative stress and the energy dysbalance hypotheses^3,4^. The Ca^2+^ hypothesis concerns the enhancement of neuronal Ca^2+^ signaling leading to excitotoxicity and neuronal death^5^, and the over-activation of calcineurin (CaN) signaling^6^ linked to altered neuronal plasticity and memory deficit. Mitochondrial dysfunction occurs early in AD^7^. The aging processes and FAD-associated mutations can promote mitochondrial dysfunctions in the central nervous system (CNS)^8–10^. Alterations of oxidative phosphorylation determine the decrease of adenosine triphosphate (ATP) and the increase of reactive oxygen species (ROS) production accompanied by fatty acid peroxidation. Both oxidative stress and decrease in ATP strongly impact neuronal function by impairing all energy-demanding processes, affecting protein folding, and causing DNA damage^11^. Recent evidence indicates that Ca^2+^ and mitochondria can be tandem players in AD development. A significant reduction in expression of mitochondrial Ca^2+^ efflux controlling proteins is observed in prefrontal cortex of both 3xTg-AD mice models and human individuals with AD^12^. Moreover, the study provides substantial experimental evidence that mitochondrial Ca^2+^ overload contributes to AD progression by promoting mitochondrial dysfunction, superoxide generation, and neuronal cell death. Mitochondria-endoplasmic reticulum (mito-ER) interaction has recently been suggested to play an important role in cellular physio-pathology^13,14^. Studies suggest that mito-ER interaction is altered in fibroblasts from FAD patients^15^ and in cellular AD models^16^. Forcing mito-ER tethering in a *Drosophila* model of AD increased lifespan of model animals^17^. These findings were focused and hypotheses considered principally for the neuronal dysfunction, while their application for astroglial cells has not been studied in details.

Astrocytes are fundamental homeostatic cells in the CNS. They provide structural, metabolic, and signaling support to neurons^18,19^. Growing body of evidence suggests that, during AD pathogenesis, astrocytic dysfunction may precede or be parallel to the neuronal dysfunction^20–22^. In this aspect, mitochondrial function in astrocytes is of special interest as it may specifically be involved in deregulation of synaptic transmission^23^. While the role of astrocytic mitochondria in AD is being now acknowledged^24,25^, it appears difficult to dissect the astrocytic versus neuronal mitochondrial dysfunction in AD brain, and the knowledge about astrocyte-specific mitochondrial alterations, their link to astrocytic Ca^2+^ signaling and other cellular processes as endoplasmic reticulum (ER)-stress proteostasis remains limited^26^.

Recently, we have generated and characterized immortalized astrocytic cell lines from hippocampi of 3xTg-AD mice, a popular and well-studied AD mouse model^27^. These lines, named WT- and 3xTg-AD immortalized astrocytes (WT-iAstro and 3Tg-iAstro) recapitulate the features of primary astrocytic cultures from 3xTg-AD mice in terms of gene profiling, protein expression and Ca^2+^ signaling^27,28^. Here we used WT-iAstro and 3Tg-iAstro lines to study mitochondrial functions and their association with Ca^2+^ signaling, mito-ER interaction and proteostasis. Our data suggest that the functional impairment of mitochondrial respiration in FAD astrocytes may be associated with deregulations of cellular Ca^2+^ homeostasis and protein synthesis through altered mitochondria-ER interaction.

## Results

### 3Tg-iAstro astrocytes have impaired ATP synthesis and mitochondrial functions

**Fig. 1:**
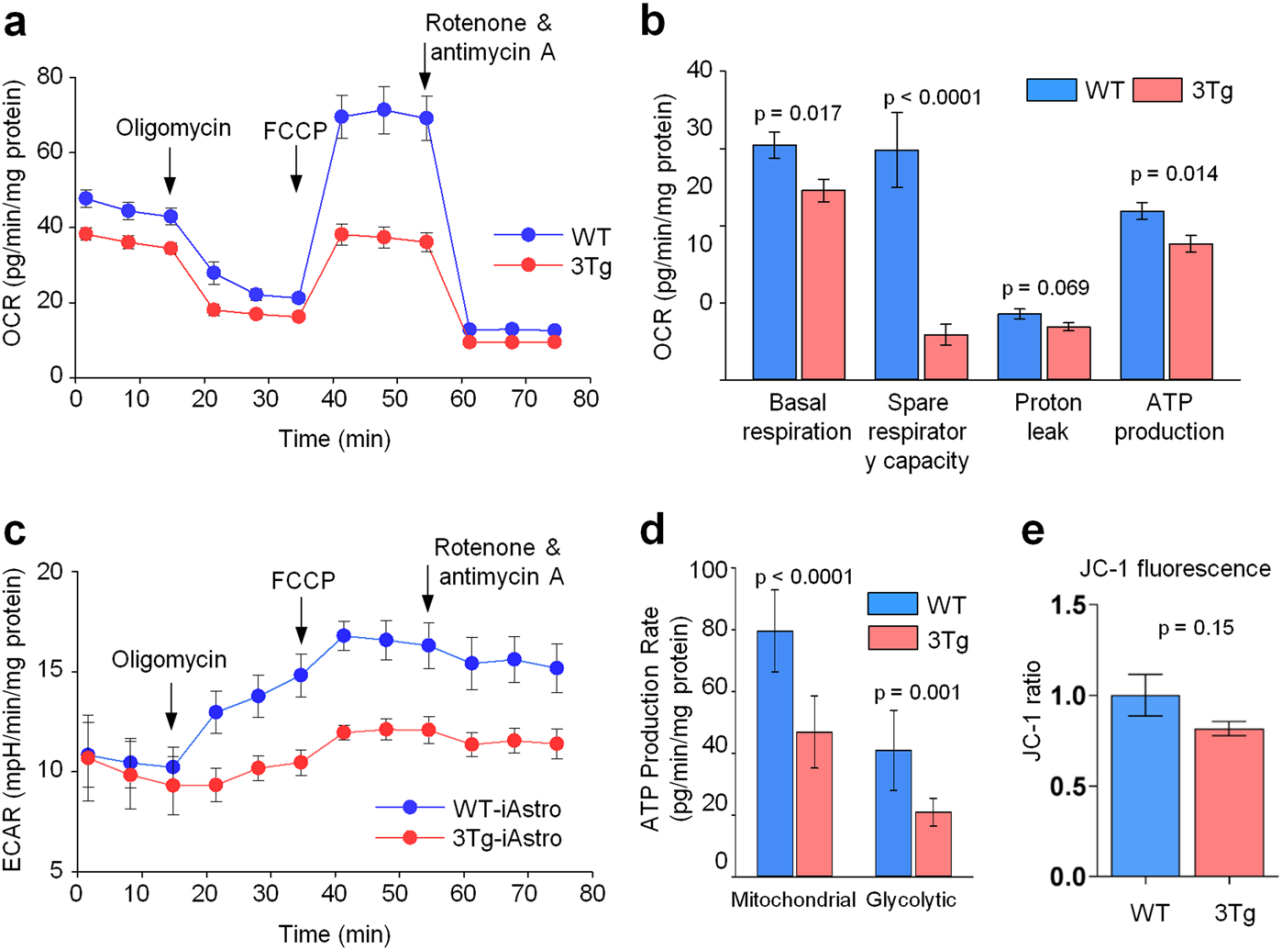
Mitochondrial and glycolytic energy metabolism is impaired in 3Tg-iAstro compared with WT-iAstro cells. Bioenergetics of iAstro cells were assessed by Seahorse Flux Analyzer using Cell Mito Stress Kit. In **a**, there are mitochondrial oxygen consumption curves presented as averages ± standard deviations of each measurement time point (*n* = 9). Initial three measurement points represent basal mitochondrial respiration, the next three points after blocking adenine nucleotide translocating by adding oligomycin represent proton leak-stimulated oxygen consumption, then follow three points representing maximal mitochondrial oxygen consumption capacity when mitochondrial inner membrane is uncoupled by FCCP, and the last three points are for non-mitochondrial oxygen consumption when mitochondrial respiratory chain is inhibited by rotenone and antimycin A (see more detailed explanation on manufacturer’s website https://www.agilent.com/en/products/cell-analysis/seahorse-xf-consumables/kits-reagents-media/seahorse-xf-cell-mito-stress-test-kit). The summary data calculated from the curves in **a** are shown in **b**. In **c** representing efficiency of glycolysis, there are curves of pH changes in the cell culture medium measured simultaneously with oxygen consumption rate. Section (**d**) shows mitochondrial and glycolytic ATP production in iAstro cells assessed by Seahorse Flux Analyzer using Real-Time ATP Rate Assay (*n* = 9). **e** Mitochondrial membrane potential was measured using JC-1 fluorescent ratiometric probe on *n* = 3 independent cultures assayed in quadriplicates (*p* = 0.15).

Investigation of metabolic activity by Seahorse XF Cell Mito Stress Assay revealed significant decrease in basal mitochondrial oxygen consumption rate (OCR) of 3Tg-iAstro cells compared to the WT-iAstro line (Fig. 1a, b). The average OCR in 3Tg-iAstros was 19% lower than in astrocytes without AD mutations. The same tendency remained for the mitochondrial OCR sensitive to ATPase inhibitor oligomycin; ATP production-coupled OCR in 3Tg-iAstro cells was also by 19% lower than in WT-iAstro cells. Moreover, when mitochondria were stressed by permeabilizing inner membrane for H^+^ with carbonyl cyanide 4-(trifluoromethoxy)phenylhydrazone (FCCP) to reveal maximal mitochondrial respiratory capacity, the responsive increase in OCR of 3Tg-iAstro cell mitochondria was five times lower compared to that in healthy WT-iAstro cells (see OCR after addition of FCCP in Fig. 1a and Spare respiratory capacity bars in Fig. 1b). There was no significant difference in proton leak-driven OCR observed between 3Tg and WT-iAstro cell lines.

Glycolysis activity of the cells was assessed as extracellular acidification rate (ECAR) simultaneously with the OCR in the same samples. There was no difference in ECAR between the two cell lines when the cells were not stressed by mitochondrial inhibitors (Fig. 1c). After blocking phosphorylation by oligomycin, the ECAR induced by WT-iAstro cells started to increase indicating glycolysis activation to restore energetic balance. However, in the probes containing 3Tg-iAstro cells, this increase was significantly lower. The impairment of both mitochondrial and glycolytic energy metabolism in 3Tg-iAstro cells was also confirmed by Seahorse XF Real-Time ATP Rate Assay (Fig. 1d). Compared to the WT-iAstro cells, the average decrease in calculated ATP amount produced by mitochondrial and glycolytic pathways was 41% and 49%, respectively.

Altered OCR and ECAR may be associated with impairment of mitochondrial membrane potential (Δψm). However, we found that, although there was a trend to a lower Δψm in 3Tg-iAstro cells, the difference was not significant (81.67 ± 3.87% of WT-iAstro, *p* = 0.15) (Fig. 1e).

### Increased mitochondrial ROS production in 3Tg-iAstro cells

**Fig. 2:**
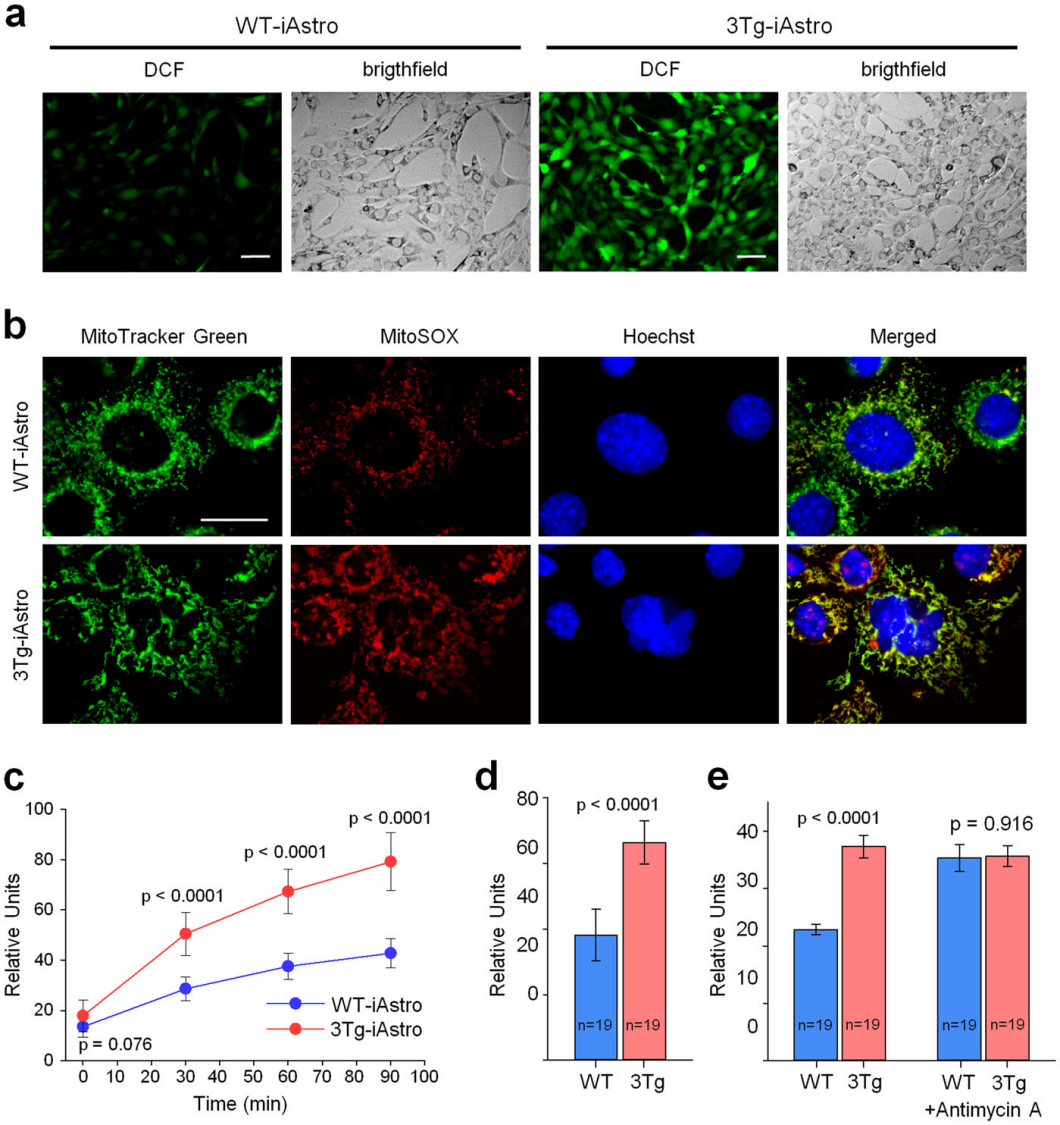
Intracellular ROS and intra-mitochondrial superoxide production are increased in 3Tg-iAstro vs WT-iAstro cells. Panels **a**, **c**, and **d** show 2’,7’–dichlorofluorescein (DCF) fluorescence in 2’,7’–dichlorofluorescin diacetate (DCFDA)-treated iAstro cells. In **a**, the bright-field images of the same fields are presented next to the fluorescent images to show the total cell number in the field, scale bar is 100 μm. Panel **c** demonstrates DCF fluorescence in iAstro cells measured at several time points after leading with DCFDA (mean ± SD of seven biological replicates) in a fluorometric plate reader. In **d**, the fluorescence intensity of the cell covered area in micrographs was assessed by ImageJ software (Huang algorithm). Panels **b** and **c** show MitoSOX fluorescence upon binding mitochondrial superoxide. In **b**, there are representative images of total mitochondrial network images visualized by MitoTracker Green and mitochondrial superoxide radicals visualized by MitoSOX, scale bar is 50 μm. The quantitative data for MitoSOX fluorescence are presented in panel **e**.

Mitochondrial dysfunction is usually related with ROS production, and both features are characteristic to neuronal pathology in AD. To investigate if mitochondrial failure is also accompanied by ROS generation in hippocampal astrocytes from AD mouse model, we have compared ROS levels in 3Tg-iAstro and WT-iAstro cells. Assessment of intracellular hydroxyl, peroxyl and other ROS by DCFDA assay revealed significant increase of the species inside 3Tg-iAstro compared to WT-iAstro cells (Fig. 2a, c, d). The difference in the level of 2,7-dichlorofluorescein (DCF) fluorescence between 3Tg-iAstro and WT-iAstro samples increased during the first 30 min of monitoring in a plate reader, and was by 76% higher in 3Tg-iAstro cells at this time point (Fig. 2c). The fluorescence intensity in 3Tg-iAstro cells remained higher by 78% and 85% after 60 and 90 min, respectively. Similarly, 73% higher DCF fluorescence intensity in 3Tg-iAstro cells compared to WT-iAstro samples was found by evaluation of cell fluorescence intensity in microscope images by ImageJ software (Fig. 2d).

The primary ROS produced within mitochondria is superoxide, most of which is converted to hydrogen peroxide by superoxide dismutase^29^. Hydrogen peroxide is membrane permeable and freely diffuses to cytosol where it might be further converted to other ROS such as hydroxyl radical by Fenton reaction. On the contrary, superoxide cannot pass membranes and remains at the production site. If the source of cytosolic ROS is mitochondrial hydrogen peroxide, the levels of mitochondrial superoxide should also be increased. Indeed, images of 3Tg-iASTRO cells loaded with mitochondrially targeted fluorescent superoxide indicator MitoSOX^TM^ Red had more intensively stained mitochondrial network compared to WT-iAstro cells (Fig. 2b, Supplementary Fig. 1). Quantitative evaluation of the images revealed 63% higher MitoSOX fluorescence in 3Tg-iAstro cells (Fig. 2e). This was equal to the level induced by mitochondrial respiratory complex III inhibitor 100 μM Antimycin A, a well-known inducer of mitochondrial superoxide, which was applied as a positive control of the assay.

### Proteomic analysis of MERE fraction from WT- and 3Tg-iAstro lines shows absence of protein expression alterations in mitochondria

**Fig. 3:**
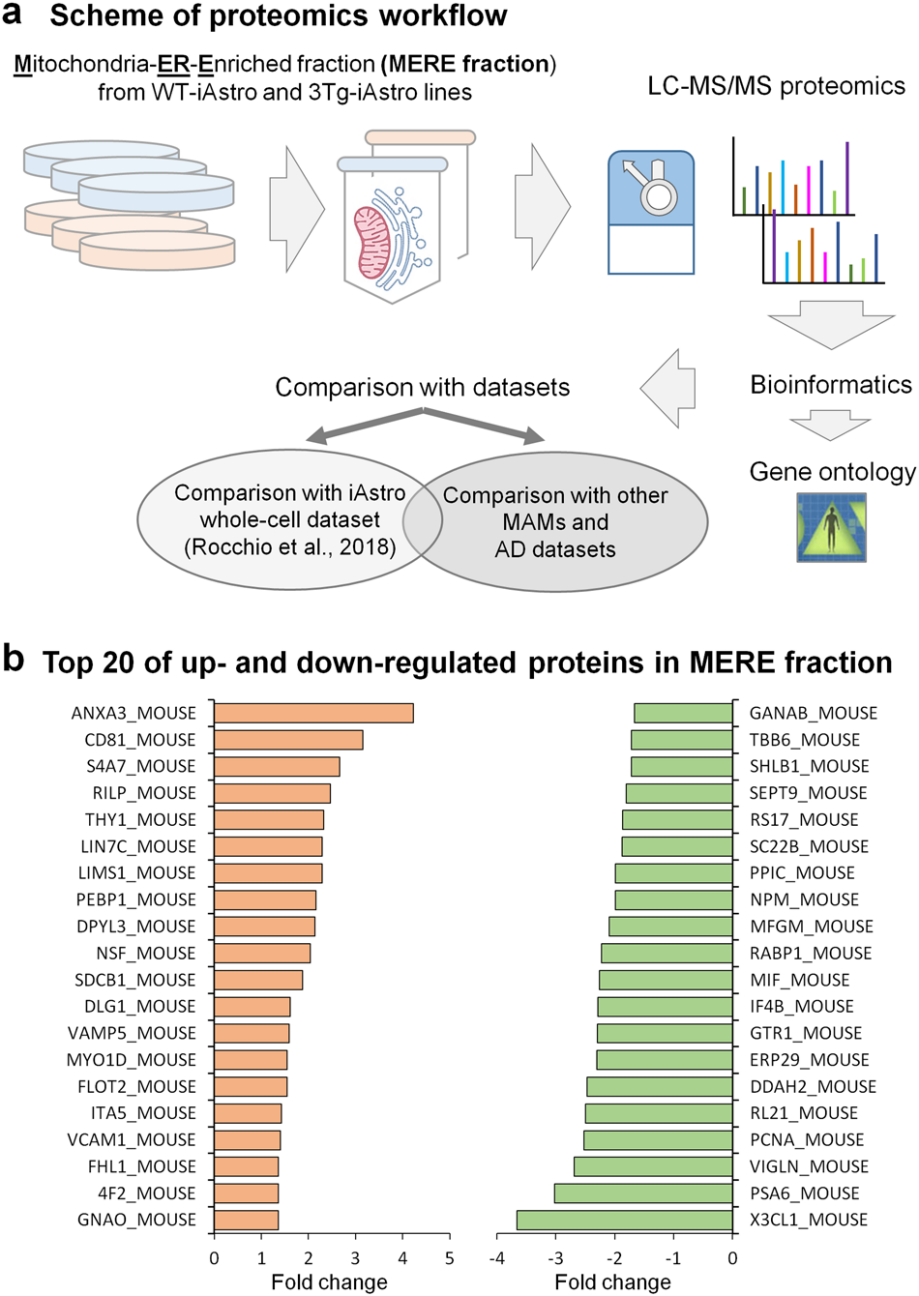
Mass spectrometry proteomic analysis of mitochondria-ER-enriched fractions form WT- and 3Tg-iAstro lines. **a** Cartoon shown workflow of proteomic and bioinformatic analysis of mitochondria-ER-enriched (MERE) fractions form WT- and 3Tg-iAstro lines. **b** Top 20 of up- and downregulated differentially expressed proteins (DEPs).

**Fig. 4:**
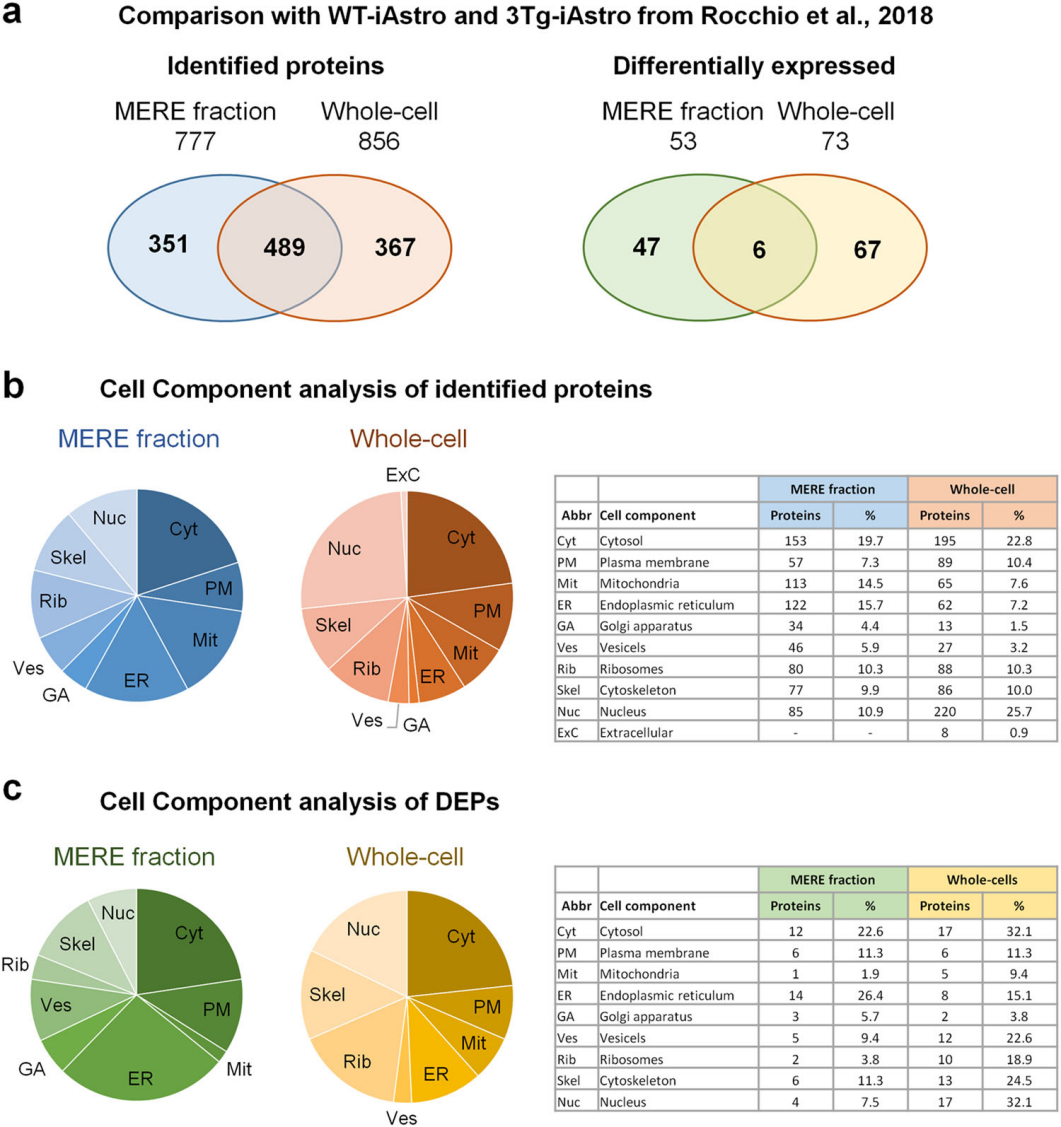
Comparison of proteomic analyses of mitochondria-ER-enriched fractions and previously published whole-cells proteomics of WT-iAstro vs 3Tg-iAstro cells. **a** comparison of identified and differentially expressed proteins in MERE fractions (this study) and whole-cell proteomics from WT- and 3Tg-iAstro cells. Cellular component analysis of identified (**b**) and differentially expressed proteins (**c**) in MERE fractions and whole-cell proteomics from WT-iAstro and 3Tg-iAstro cells.

Next, we thought to investigate if the impairment of cellular bioenergetics was a result of altered expression of protein components including glycolysis and mitochondrial OXPHOS pathway as it was recurrently suggested for AD^30–32^. For this, we have isolated Mitochondria/Endoplasmic Reticulum-Enriched (MERE) fractions and performed shotgun mass spectrometry (SG-MS) proteomics followed by bioinformatic analysis (Fig. 3a). 1089 and 928 proteins have been identified in WT-iAstro and 3Tg-iAstro lines, respectively, with 777 proteins common for WT and 3Tg samples (Supplementary Table 1a). Quantification of proteins resulted in 53 differentially expressed proteins (DEPs) in 3Tg-iAstro vs WT-iAstro cells (Fig. 3b and Supplementary Table 1b). Comparison with our previously published proteome dataset obtained on whole-cell lysates from same WT-iAstro and 3Tg-iAstro cells^27^ showed 489 common proteins between MERE fraction and whole-cell lysate, while only six DEPs were found to be common between MERE fractions and whole-cell preparations (Fig. 4a). Next, we analyzed the sub-cell compartment composition of identified and DEPs proteins in MERE fraction in comparison with whole-cell dataset^27^. For this, proteins were assigned to one of the following ten groups according to Genecards database (https://www.genecards.org/): *cytosol*, *plasma membrane*, *mitochondria*, *ER*, *Golgi apparatus (GA)*, *intracellular vesicles*, *ribosomes*, *cytoskeleton*, *nucleus*, and *extracellular proteins*. In the identified proteins list we found near two-fold enrichment of mitochondrial and ER proteins and three-fold enrichment of GA proteins in MERE fraction compared with whole-cell proteins with 30% decrease of PM and the absence of extracellular proteins (Fig. 4b). Surprisingly, in MERE fraction DEPs, there was only one mitochondrial protein, dimethylarginine dimethylaminohydrolase 2 (Uniprot_ID DDAH2_MOUSE, mouse gene Ddah2). Instead, there were 43 and 33% more ER and GA proteins, respectively (Fig. 4c). Cytosolic, intracellular vesicle, ribosomal, cytoskeletal, and nuclear proteins were less abundant (by 30%, 59%, 79%, 54%, and 77%, respectively). Of note, components of the glycolytic pathway were absent in DEPs of MERE fraction, while whole-cell lysate DEPs list contained two glycolytic enzymes, namely fructose-bisphosphate aldolase A (Aldoa, 1.47 fold change) and of pyruvate kinase isoform M1 (Pkm, −2.21 fold change).

### Bioinformatic analysis of MERE fraction and whole-cell lysate DEPs from WT- and 3Tg-iAstro lines shows alterations in adhesion, ER and protein synthesis

**Fig. 5:**
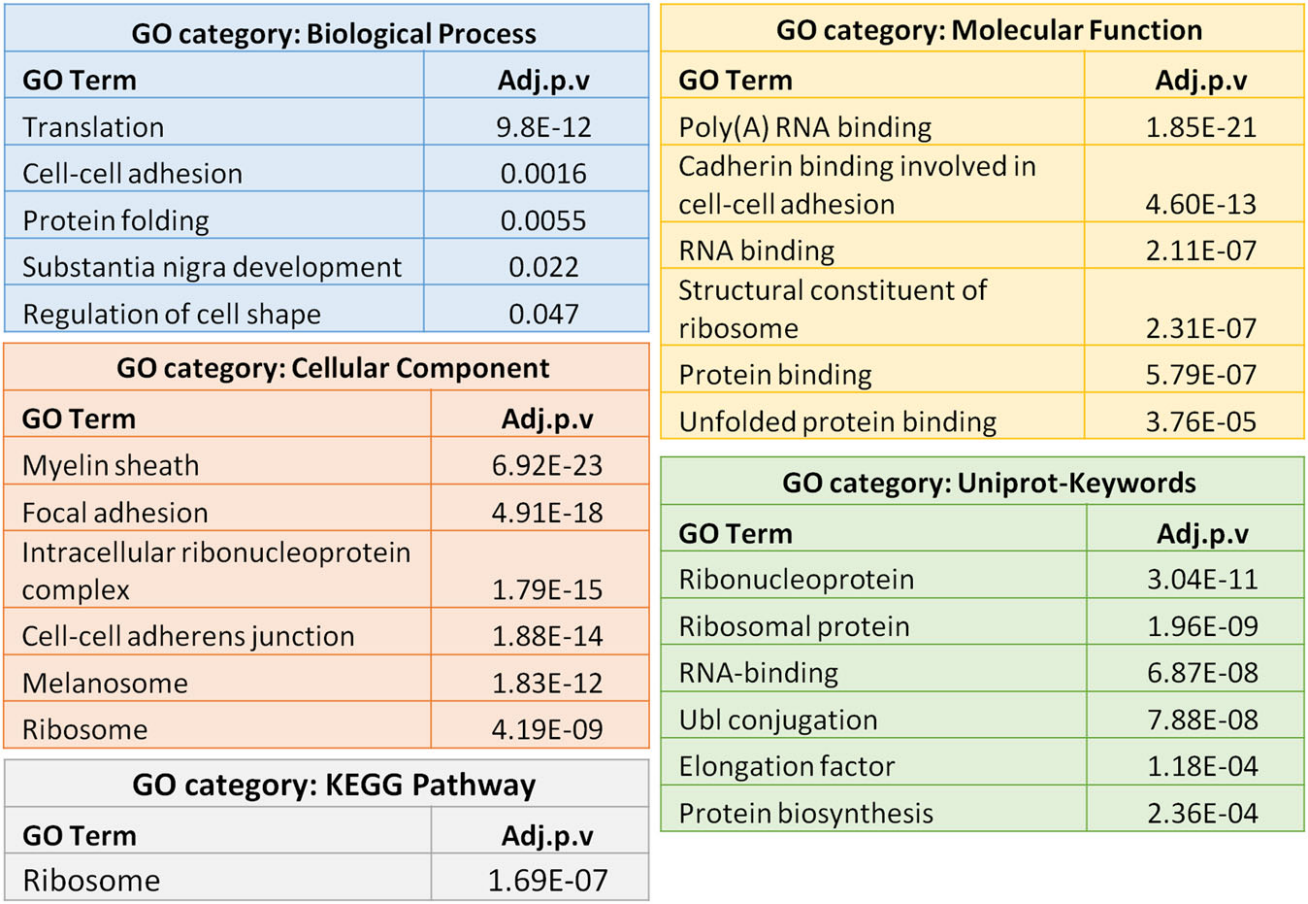
Analysis of protein-protein interaction network in merged DEPs list from MERE fractions and whole-cell proteomics from 3Tg-iAstro vs WT-iAstro cells using DAVID tool. List of 120 DEPs of joined lists of MERE fractions and whole-cell lysates from 3Tg-iAstro vs WT-iAstro cells was subjected to DAVID gene ontology tool analysis. The most significantly overrepresented specific GO terms are listed for Biological Process, Cellular Component, Molecular Function, Uniprot-Keywords, and KEGG Pathway categories. Complete GO analysis results are provided in Supplementary Table 2.

To investigate the functional significance of protein expression alterations we merged MERE fraction DEPs with those of whole-cell lysates. DAVID gene ontology analysis of 120 DEPs of the merged list resulted in 81 significantly overrepresented GO terms. Top overrepresented GO terms were as follows: Biological Processes – *translation*, *cell-cell adhesion,* and *protein folding*; among Cell Component terms – *myelin sheath*, *focal adhesion*, *intracellular ribonucleoprotein complex*, *cell-cell adherens junction,* and *ribosome*; among Molecular Function terms – *poly(A) RNA binding*, *cadherin binding involved in cell-cell adhesion*, *RNA binding*, *structural constituent of ribosome*, *protein binding*, *unfolded protein binding*. The overrepresented KEGG pathway was *Ribosome* (Fig. 5 and Supplementary Table 2).

### Poor overlap with MAMs and mitochondrial datasets from mouse AD models

Next, we compared MERE fraction and whole cells 3Tg-iAstro datasets with mitochondria-associated membranes (MAMs) and mitochondrial proteome datasets reported by Völgyi et al.^33^ and Yu et al.^31^, respectively. Völgyi et al. reported deregulation of many ribosomal proteins in MAMs from 3-month-old APP/PS1 AD mouse. However, there were only two proteins common with our MERE fraction dataset: oppositely regulated ribosomal protein 21 (Rpl21) and co-downregulated Syntenin-1 (Sdcbp) (Supplementary Table 3a, b). Dataset by Yu et al. features mitochondrial proteome from hippocampus and cortex of symptomatic 15-month-old 3xTg-AD mice, the same from which 3Tg-iAstro originated. Also in this case two protein were common with our MERE fraction dataset: co-downregulated vacuolar proton pump subunit B2 (Atp6v1b2) and oppositely regulated vesicular-fusion ATPase NSF (Supplementary Table 3c, d).

Taken together, these results suggest that the expression of mitochondrial proteins, which have been identified in MERE fractions, was largely unaltered in WT-iAstro and 3Tg-iAstro lines. Instead, the protein expression alterations are mainly related to ER and protein synthesis and processing. We reasoned that, in terms of association with protein expression, OXPHOS deficiency in 3Tg-iAstro cells may be associated with extra-mitochondrial factors. Therefore, we decided to focus on investigation of the following aspects: (i) relationships between Ca^2+^ signaling in the cytosol, ER and mitochondria; (ii) mitochondria-ER interaction; and (iii) assessment of the ER-stress/UPR and protein synthesis in 3Tg-iAstro vs WT-iAstro cells.

### Mitochondrial Ca^2+^ signaling is inhibited in 3Tg-iAstro compared with WT-iAstro cells

**Fig. 6:**
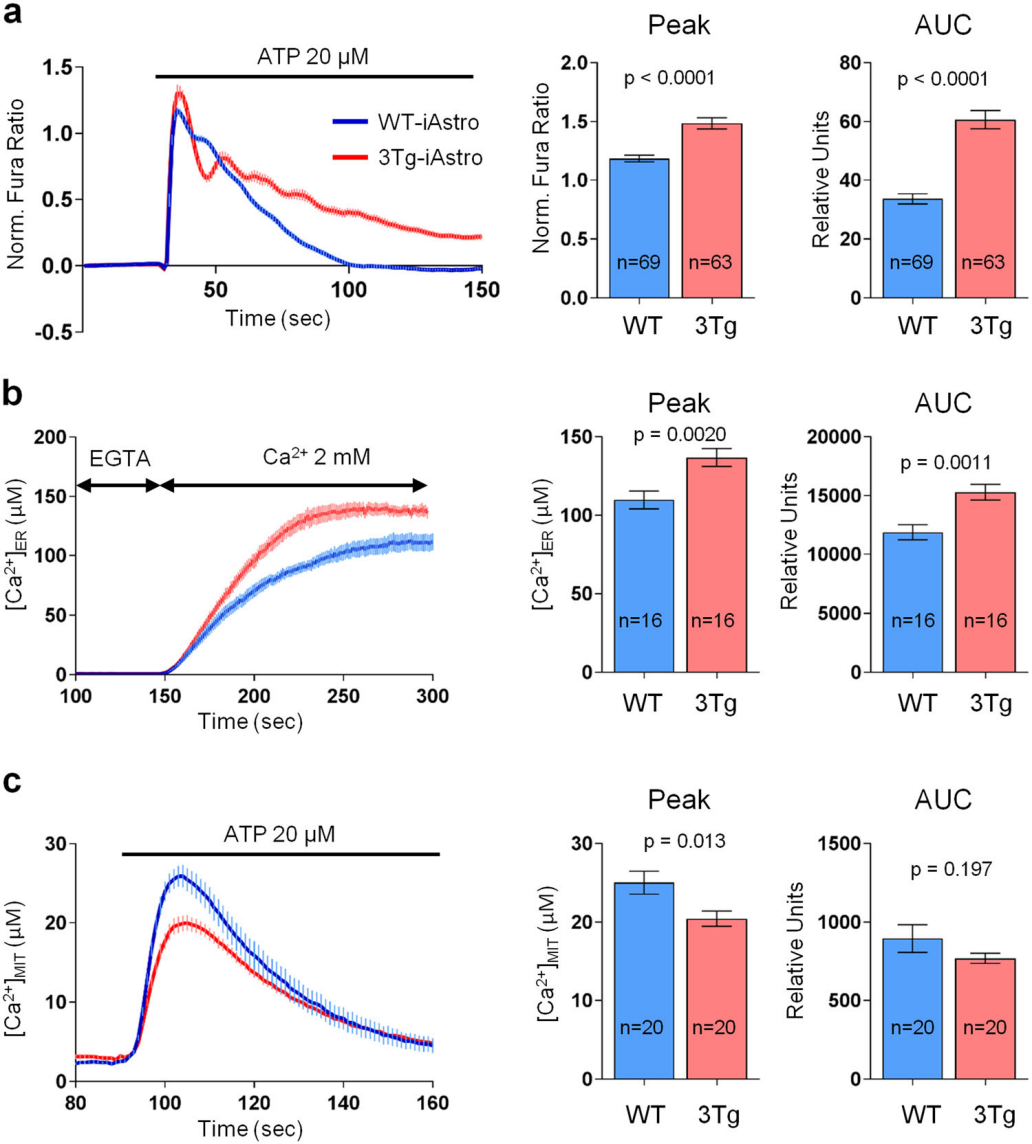
Mitochondrial Ca^2+^ signaling is impaired in 3Tg-iAstro as compared with WT-iAstro cells. **a** WT- and 3Tg-iAstro cells, previously loaded with Fura-2, were stimulated with 20 μM ATP in Ca^2+^-containing KRB solution. Data are expressed as mean of peak ± SEM (*p* < 0.0001) or mean and of area under the curve (AUC) ± SEM (*p* < 0.0001) of 69 WT-iAstro cells and 63 3Tg-iAstro cells from nine coverslips from three independent experiments. **b** WT- and 3Tg-iAstro cells, stably expressing ER-AEQ Ca^2+^ sensor, were reconstituted with coelenterazine n (low light-emitting coelenterazine) in KRB solution supplied with 600 μM EGTA and 3 μM ionomycin at 4 °C for 1 h. After reconstitution and washing, the cells were perfused first with KRB containing 100 μM EGTA and then with 2 mM Ca^2+^. Until the traces reach steady-state levels. The data are summarized in histograms and expressed as mean ± SEM of μM steady-state [Ca^2+^] (*p* = 0.0020) and areas under the curves (AUC) (*p* = 0.0011) of 16 coverslips from four independent experiments. **c** WT- and 3Tg-iAstro cells, stably expressing MIT-AEQ Ca^2+^ sensor, were reconstituted with coelenterazine wt (native coelenterazine) in complete culture medium for 1 h. After reconstitution and washing, the cells were perfused first with KRB containing 2 mM Ca^2+^. When indicated, perfusion was switched to KRB supplemented with 2 mM Ca^2+^ and 20 μM ATP. The data are expressed as mean ± SEM μM of peak of [Ca^2+^] (*p* = 0.013) and of the AUC (*p* = 0.197) from 20 coverslips from four independent experiments (biological replicates).

First, we investigated if ATP-induced mitochondrial Ca^2+^ signals in 3Tg-iAstro cells in comparison with WT-iAstro cells were different from those detected in the cytosol and if the levels of Ca^2+^ in the ER may be associated with cytosolic and mitochondrial Ca^2+^ elevations. In the previous report we found that ATP-induced Ca^2+^ signals in 3Tg-iAstro lines were augmented as compared with WT-iAstro cells^27^. Here, using Fura-2 Ca^2+^ imaging we confirm this finding both in terms of the amplitude of Ca^2+^ increase (1.18 ± 0.028 norm. Fura-2 ratio for 3Tg-iAstro, *n* = 63, vs 1.48 ± 0.047 for 3Tg-iAstro, *n* = 69, *p* < 0.0001) and of the area under the Ca^2+^ trace (33.69 ± 1.73 arbitrary units for 3Tg-iAstro, *n* = 63, vs 60.61 ± 3.11 for 3Tg-iAstro, *n* = 69, *p* < 0.0001) (Fig. 6a). Next, using ER-targeted aequorin Ca^2+^ sensor ER-AEQ^34^, we assessed whether the ATP-induced cytosolic Ca^2+^ transient may be associated with an augmented ER Ca^2+^ load. As shown in Fig. 6b, the steady-state ER luminal Ca^2+^ level was significantly higher in 3Tg-iAstro cells (136.8 ± 5.65 μM, *n* = 16) as compared with WT-iAstro cells (109.7 ± 5.66 μM, *n* = 16, *p* = 0.002), confirming the higher ER Ca^2+^ load hypothesis. After this, employing MIT-AEQ aequorin Ca^2+^ sensor targeted to the mitochondrial matrix^34^ we assessed ATP-evoked mitochondrial Ca^2+^ transients. Figure 6c shows that ATP-induced Ca^2+^ uptake in 3Tg-iAstro mitochondria was significantly lower than that in WT-iAstro mitochondria (20.41 ± 0.98 μM, *n* = 20 vs 25.01 ± 1.47 μM, *n* = 20, respectively, *p* = 0.013). These results indicate that, in spite of the augmented ER Ca^2+^ content and increased ATP-induced cytosolic Ca^2+^ release, mitochondria in 3Tg-iAstro cells are defective in up-taking Ca^2+^ in comparison with WT-iAstro cells.

### Shortened mitochondrial-ER distance in 3Tg-iAstro cells

**Fig. 7:**
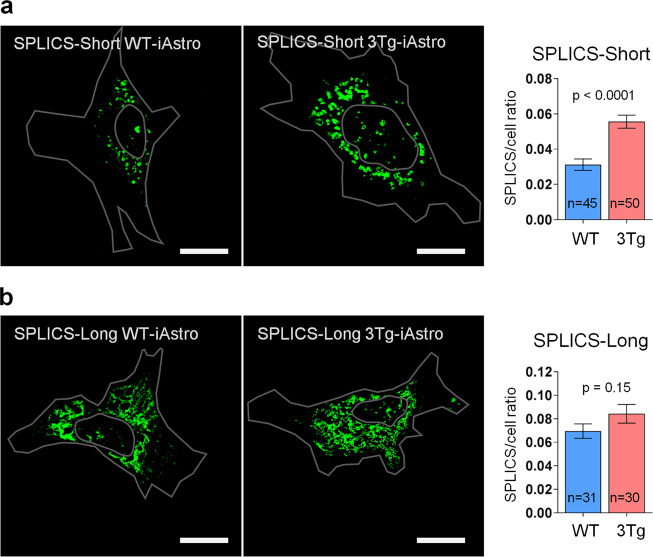
Increased short-distance mitochondrial-ER tethering in 3Tg-iAstro as compared with WT-iAstro cells. **a** SPLICS-Short (≈8–10 nm) fluorescence in WT-iAstro (left panel) and in 3Tg-iAstro cells (right panel). For each cell fluorescence area was normalized to cell surface area and expressed as mean ± SEM of *n* = 45 (WT-iAstro) and *n* = 50 (3Tg-iAstro), *p* < 0.0001. **b** SPLICS-Long (≈40–50 nm) fluorescence in WT-iAstro (left panel) and in 3Tg-iAstro cells (right panel). Data expressed as mean ± SEM SPLICS-Long/cell area of *n* = 31 (WT-iAstro) and *n* = 30 (3Tg-iAstro), *p* = 0.15. With gray contour is depicted area of the cell and the nucleus. Bar, 20 μm.

The deficient mitochondrial Ca^2+^ signaling, among other reasons, may be mediated by the alterations in the relationships between mitochondria and ER^35^. We have investigated this exploiting the recently generated SPLICS probe which allows visualizing mitochondrial-ER interaction point at two defined distances: ≈8–10 nm (SPLICS-Short) and ≈40–50 nm (SPLICS-Long)^15^. In WT-iAstro cells SPLICS-Short was visualized as fluorescent dots localized mainly in the perinuclear zone (Fig. 7a). In 3Tg-iAstro cells SPLICS-Short expression resulted in a significantly higher number of fluorescent dots some of them appeared to be fused. Quantification of fluorescence area normalized for cell surface showed near doubling of the short-distance mito-ER contacts in 3Tg-iAstro cells as compared with WT-iAstro cells (0.055 ± 0.0037 SPLICS/cell ratio vs 0.031 ± 0.0032 SPLICS/cell ratio, respectively, *p* < 0.0001) (Fig. 7a). SPLICS-Long expression in both WT-iAstro and 3Tg-iAstro cells was visualized as structures with diverse morphology, from dots of different size to tubular structures reminiscent shape of mitochondria. Overall, the SPLICS-Long fluorescence area in WT-iAstros was significantly higher than that of SPLICS-Short (*p* < 0.0001). Nevertheless, the difference in SPLICS-Long/cell area ratio between 3Tg-iAstro and WT-iAstro cells was not significative (0.084 ± 0.008 SPLICS/cell ratio vs 0.069 ± 0.0062 SPLICS/cell ratio, respectively, *p* = 0.15) (Fig. 7b) indicating that specifically short distance mito-ER interaction is augmented in 3Tg-iAstro cells.

### Activation of unfolded-protein response and reduced protein synthesis in 3Tg-iAstro compare to WT-iAstro cells

**Fig. 8:**
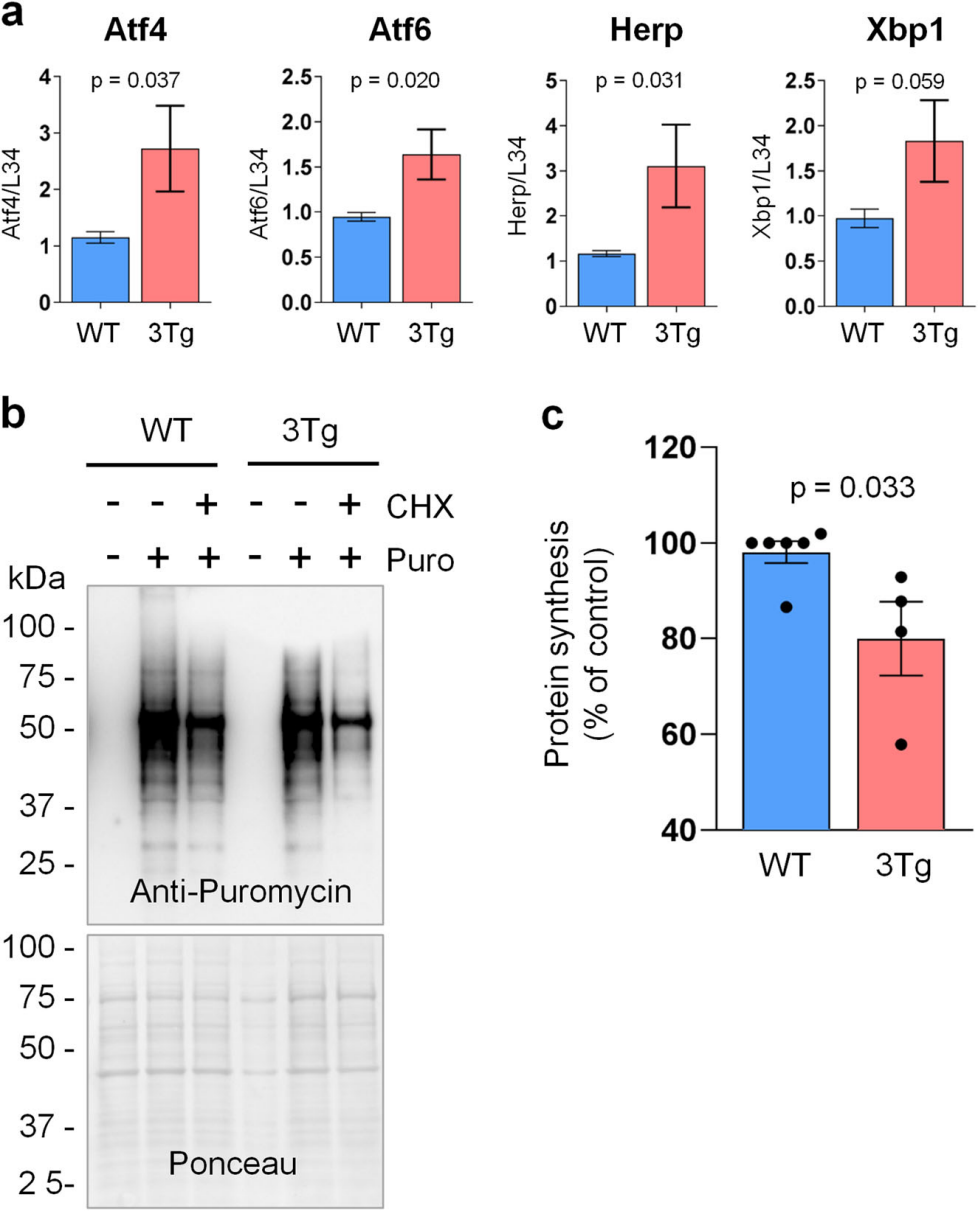
Activation of ER-stress and reduction of protein synthesis in 3Tg-iAstro vs WT-iAstro cells. **a** Real-time PCR of ER-stress/UPR response-related genes Atf4 (*p* = 0.037), Atf6 (*p* = 0.02), Herp (*p* = 0.031) and Xbp1 (*p* = 0.059) of 3Tg-iAstro vs WT-iAstro cells. Data are expressed as mean ± SEM ΔC(t), *n* = 14 for WT-iAstro, *n* = 12 biological replicates for 3Tg-iAstro cells. **b** WT and 3Tg-iAstro were pulsed with puromycin for 3 h. Where indicated, cells were also treated with cycloheximide (CHX, 10 μM) ten minutes before adding puromycin. Anti-puromycin antibody was used to detect neo-synthesized peptides. Ponceau staining was used for band normalization. **c** Quantification of anti-puromycin detected bands. Data are expressed as mean ± SEM, six and four independent cultures were used for WT and Tg-iAstro, respectively (*p* = 0.033; two-tailed unpaired Students’s *t* test with Mann–Whitney correction).

Proteomic analysis suggests that protein synthesis and processing may be impaired in 3Tg-iAstro cells. Therefore, we first measured mRNA levels of ER-stress and UPR-related gene. Real-time PCR analysis shows that Atf4, Atf6, and Herp mRNA were significantly upregulated in 3Tg-iAstro cells by 136% (*p* = 0.037), 73% (*p* = 0.020) and 166% (*p* = 0.031), respectively (Fig. 8a). Xbp1 showed a trend of upregulation (by 88%) although the difference failed to reach significance (*p* = 0.059). To investigate protein translation efficiency, we used a method of surface sensing of translation (SUnSET)^36,37^. After puromycin pulsing of WT and Tg-iAstro we observed a 20% (*p* = 0.033) decrease of puromycin incorporation in 3Tg-iAstro compare to WT-iAstro (Fig. 8b, c).

## Discussion

In the present contribution we took advantage of the recently generated AD astrocytic cellular model, immortalized hippocampal astrocytes from 3xTg-AD mice (3Tg-iAstro and WT-iAstro cells) to investigate the possible association between astrocytic bioenergetic status, ER and mitochondrial Ca^2+^ homeostasis and proteostasis, which includes protein synthesis and degradation machinery. We found that in 3Tg-iAstros (i) glycolysis, OCR and ECAR are significantly lower compared to WT-iAstros, (ii) intracellular and intramitochondrial ROS production is elevated, however (iii) the changes are not due to alterations in expression of mitochondrial proteins, but rather due to extra-mitochondrial protein expression changes, resulting in (iv) ER Ca^2+^ overload and reduced ATP-evoked mitochondrial Ca^2+^ transients; (v) increase of area with a shorter (8–10 nm) mitochondria-ER interaction distance; (vi) increased expression of ER-stress/UPR-associated genes; and (vii) reduced protein synthesis rate in 3Tg-iAstro compare with WT-iAstro cells.

Two glycolytic proteins were found to be changed in our dataset collected on whole-cell iAstro cells, namely fructose-bisphosphate aldolase A (Aldoa) and isoform M1 of pyruvate kinase PKM (Pkm). Aldoa catalyzes the reversible conversion of fructose-1,6-bisphosphate to glyceraldehyde 3-phosphate and dihydroxyacetone phosphate and is critically involved in glycogen storage: deficiency of this enzyme causes Glycogen Storage Disease XII leading to excessive liver accumulation of glycogen^38^. Therefore, increase of Aldoa expression in 3Tg-iAstro cells may potentially lead to the depletion of glycogen from astrocytes which would reduce bioenergetic reserve of the AD CNS. The second enzyme, Pkm, a member of pyruvate kinases (PK) family, catalyzes the last, and rate-limiting, step of glycolysis by the transfer of a phosphoryl group from phosphoenolpyruvate to ADP generating ATP and pyruvate^38^. Downregulated glycolytic response to mitochondrial stress was also confirmed in human iPSC-derived astrocytes with PS1-ΔE9 mutations^39^. Astrocytes have significantly higher glycolytic metabolism than neurons through which they feed neurons, store glycogen and metabolize and recycle neurotransmitters^40,41^. Slowing down astrocytic glycolysis may have detrimental effects on neuronal activity and survival.

Ca^2+^ is an important regulator of mitochondrial oxidative metabolism and is required for the activity of enzymes of the tricarboxylic acid cycle, the proteins of the electron transport chain, and the ATP synthase^42,43^. In these terms, the decrease of mitochondrial Ca^2+^ may explain the reduced OCR and respiratory capacity of 3Tg-iAstro cells. Moreover, our results are in line with those obtained on APPswe/PS1A246E-expressing astroglioma cells in which reduced mitochondrial OCR was accompanied by insignificant change in mitochondrial Ca^2+^ in front of strongly potentiated ATP-induced cytosolic Ca^2+^ increase^44^. However, it has been reported that in iPSC-derived astrocytes form patients bearing PS1-ΔE9 mutation basal mitochondrial respiration was increased^39^. Such discrepancies may arise from differences in generation of cell models as well as from different AD-related mutations.

**Fig. 9:**
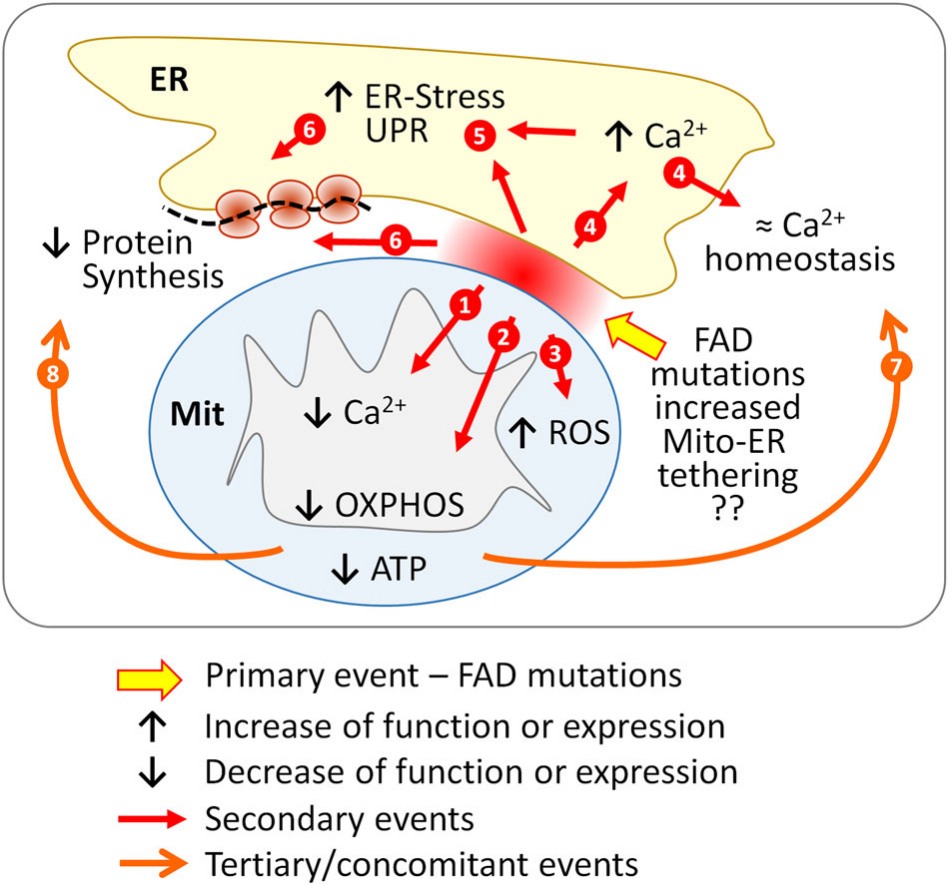
Scheme of possible relationships between FAD mutations, mitochondrial-ER interaction, mitochondrial and ER Ca^2+^ signaling, bioenergetic status of mitochondria, ER-stress/UPR and protein synthesis. FAD mutations-provoked increase in short distance mitochondrial-ER contact sites may lead to (1) deficient mitochondrial Ca^2+^ signaling, (2) decrease of OxPhos function and production of ATP and (3) to increase of ROS. From the ER side it may lead to (4) overload of the ER with Ca^2+^ and deregulation of cellular Ca^2+^ homeostasis, (5) induction of ER-stress/UPR response as well as (6) to inhibition of protein synthesis (red arrows). Secondary events may include negative feedbacks of the ATP deficit and the increased ROS on (7) the cellular Ca^2+^ homeostasis and (8) protein synthesis (orange arrows).

A growing body of evidence suggests that the mitochondrial-ER interaction, in terms of both distance and protein composition, may be central for deregulation of Ca^2+^ signaling, mitochondrial functions and ER-stress^14,35,45,46^. It has been found that the distance between ER and mitochondria is kept in a range of ≈10–50 nm^13^. Increase as well as decrease of this distance affects multiple aspects of mitochondrial functions: Ca^2+^ signaling, lipid transfer and biogenesis, protein synthesis and transport. We found that in 3Tg-iAstro cells the short distance (≈8–10 nm) sites area is increased by 77%. Although at the moment we cannot draw cause-effect relationships between the mito-ER contact sites shortening and the other findings of this work, we propose a hypothetical mechanistic sequence of events which may occur in astrocytes and has in its center FAD mutations-induced increase in mito-ER tethering at a short distance. First of all it explains reduced mitochondrial Ca^2+^ signals in concomitance with ER Ca^2+^ overload. The mito-ER distance shorter than 10-12 nm creates steric encumbrance for IP_3_Rs which hampers Ca^2+^ transfer from IP_3_Rs to mitochondria^13^. Reduced mitochondrial Ca^2+^ signals may lead to reduced OXPHOS efficiency and ATP production^42^, while cellular Ca^2+^ overload may be a cause for ER-stress/UPR^47^. Both mitochondrial and ER-related abnormalities due to increased agonist-induced Ca^2+^ release and impaired supply of ATP may lead to global deregulation of cellular Ca^2+^ homeostasis. At the same time ER-stress and energy deficit may slow down protein synthesis and degradation machinery as these processes are among the most energy consuming in the cell. While details of this hypothetic scenario, illustrated in Fig. 9, are still to be elucidated, recent publication corroborate our results. It has been show that mito-ER tethering in a short mito-ER distance range is augmented in both presenilin 2 N141I mutant human fibroblasts^15^ and upon overexpression of AD-relevant caspase 3-cleaved WT Tau, which is localized specifically at mito-ER contact sites^48^.

## Conclusions

In the CNS, neuronal performance, survival and resilience in stressful or diseased conditions strongly depends on homeostatic support of astroglial cells^19^. During AD pathogenesis, astrocytes, in their turn, undergo complex, brain region-specific and disease stage-dependent remodeling^21,49^. Our data suggest that these changes concern multiple aspects of astroglial biology, characteristic for AD as the deregulation of cellular bioenergetics and Ca^2+^ signaling, mitochondria-ER interaction and proteostasis. The effects of these changes, in turn, may have multiple consequences, e.g., we recently have shown that conditioned medium from 3xTg-AD astrocytes reduces synaptic proteins in cultured neurons^50^, while 3Tg-iAstros fail to support the integrity of the blood-brain barrier in vitro^51^. In light of this, our work exemplifies the importance of multidisciplinary approach in studying such complex phenomena as AD and illustrates the use of iAstro cells as versatile and cost-effective astrocytic cellular model of AD. To conclude, our results highlight the important role that astrocytes play in AD pathogenesis and suggest that the alterations in their homeostatic capabilities contribute to AD progression at early stages of the disease.

## Material and methods

### Immortalized hippocampal astrocytes from 3xTg-AD mice

Generation of immortalized astrocytes from hippocampi of WT and 3xTg-AD mice (WT- and 3Tg-iAstro cells) was described elsewhere^27^. iAstro lines were maintained in complete culture media containing Dulbecco’s modified Eagle’s medium (DMEM; Sigma-Aldrich, Cat. No. D5671) supplemented with 10% fetal bovine serum (Gibco, Cat. No. 10270), 2 mM L-glutamine (Sigma-Aldrich), and 1% penicillin/streptomycin solution (Sigma-Aldrich). Cells were passaged once a week and used for experiments between passages 12 and 20 from establishment^27^.

### Evaluation of iAstro energetic metabolism and ROS production

#### Mitochondrial respiration

Assessment of mitochondrial and glycolytic functionality was performed by Seahorse XFp Analyser (Agilent Technologies, Santa Clara, CA, USA) using Seahorse XFp Cell Mito Stress Test Kit (Agilent Technologies) and Seahorse XFp Real-Time ATP Rate Assay Kit (Agilent Technologies) according to manufacturer’s instructions. Briefly, iAstro cells were seeded into Agilent Seahorse XFp well plates at a density of 100,000 cells/cm^2^ and kept in complete culture medium (described in 4.1.) in incubator for 14–16 h. One hour before the measurement, the medium was replaced with Seahorse XF Assay Medium supplemented with 2 mM L-glutamine, 1 mM sodium pyruvate and 10 mM glucose and cells were placed to non-CO_2_ incubator. Just before the measurement, medium was changed again to the fresh Assay Medium with the same supplements. Final inhibitor concentrations in the wells were 1.5 μM oligomycin, 1 μM carbonyl cyanide-4-phenylhydrazone (FCCP), 0.5 μM antimycin A, and 0.5 μM rotenone. Oxygen consumption rate (OCR) and extracellular acidification rate (ECAR) were normalized to total cellular protein content determined directly in the plate immediately after each experimental run by Bradford assay. Optical density after reaction with the Bradford reagent (Cat. No. B6916, Merck) was assessed by a multifunctional plate reader Infinite 200 Pro M Nano Plex (Tecan, Männedorf, Switzerland). The data were analyzed and single run reports were generated by Wave software (Agilent Technologies), and graphical images of summary data were created by SigmaPlot vs.13 (Systat Software, Slough, UK).

#### Reactive oxygen species determination

For intracellular ROS measurement, cells were seeded in black (for plater reader) and transparent (for microscopy) 96-well plates at a density of 60,000 cells/cm^2^ and grown for 48 h in the conditions described in 4.1. After three washes with 37 °C 200 µl Hank’s Balanced Salt Solution with Ca^2+^ and Mg^2+^ (HBSS/Ca/Mg, Thermo Fisher Scientific), the cells were incubated with 10 μM 2’,7’-dichlorofluorescin diacetate (DCFDA, Cat. No. D6883, Sigma-Aldrich) in HBSS/Ca/Mg in the dark at 37 °C for 30 min. After incubation, not incorportated DCFDA is washed away by three times replacing fresh 200 µl 37 °C-warm HBSS/Ca/Mg. After last wash, the fluorescence was read in a multifunctional plate reader Infinite 200 Pro M Nano Plex (Tecan, Männedorf, Switzerland) at excitation/emission wavelengths 485 nm/535 nm. The images of DCFDA-loaded iAstro cells were also taken under fluorescent microscope Zeiss Axio Observer.Z1 (Carl Zeiss, Oberkochen, Germany) and fluorescence intensity of the cells (excluding cell uncovered areas) was evaluated by ImageJ freeware.

#### Mitochondrial superoxide determination

For evaluation of mitochondrial superoxide, the cells were grown in clear 96-well plates the same way as for intracellular ROS assessment. 48 h after plating, the cells were 3x washed with HBSS and incubated with 2 µM MitoSox^TM^ Red (Cat. No. M36008, Thermo Fisher Scientific) in HBSS at 37 °C in the dark for 15 min. The images were taken by fluorescent microscope Zeiss Axio Observer.Z1 and fluorescence intensity in the images was evaluated by ImageJ software. For higher magnification images for monitoring MitoSox staining colocalization with mitochondrial network, the cells were grown on glass-bottom Petri dishes (Catalog No. 0030740009, Eppendorf). For visualization of total mitochondrial network, 500 nM MitoTracker Green FM (Cat. No. M7514, Thermo Fisher Scientific) and for nuclear chromatin staining, 6 μg/ml Hoechst33342 (Sigma-Aldrich) was added to incubation together with MitoSox prior to microscope imaging.

#### Mitochondrial membrane potential determination

Variation in mitochondrial membrane potential (Δψm) is an important parameter of mitochondrial function and is an indicator of cell health. Mitochondrial membrane potential (Δψm) was measured by using 5,51,6,61-tetrachloro-1,11,3,31tetraethylbenzimidazolyl carbocyanine iodide (JC-1; Cayman, Ann Arbor, Michigan, USA) according to manufacturer’s instructions as has been previously describes^52,53^. Briefly, WT- and 3Tg-iAstro cells were plated (10 000 cells/well in 96-well plate) 24 h before measurement. Then the cells were incubated with JC-1 diluted in Assay Buffer for 15 min at 37 °C. After washing (twice with Assay Buffer), the mitochondrial membrane potential was determined by measuring the red (excitation 535 nm/emission 595 nm) and green (excitation 485 nm/emission 535 nm) fluorescence using a spectrophotometer (VICTOR™ X Multilabel Plate Reader, PerkinElmer). Δψm was calculated as ratio of red/green fluorescence intensities and the data are expressed as a fold change of WT-iAstro cells.

### Preparation of mitochondria-ER enriched fractions

WT-iAstro and Tg-iAstro were plated at concentration of 0.5 × 10^6 cells/dish in 10 cm Petri dishes (50 dishes per line) and grown until 80–90% confluence. On the day of experiment, the cells were washed twice with PBS, detached with trypsin, counted and collected 20 × 10^6 cells/tube. Fractionation was performed using (Cat. 89874, ThermoFisher, Milan, Italy) according to manufacturer’s instructions. Final pellets containing mitochondrial-ER enriched fraction (MERE fraction) were lysed with 50 µL of lysis buffer (50 mM TrisHCl (pH 7.4), sodium dodecyl sulphate (SDS) 0.5%, 5 mM EDTA), 5 µL of protease inhibitors cocktail (PIC, Calbiochem). After quantification (QuantiPro BCA Assay Kit, QPBSA-1kit, ThermoFisher, Milan, Italy), MERE fractions were snap frozen and stored at -80 °C. Four independently prepared samples of each, WT-iAstro and Tg-iAstro, were subjected to proteomic analysis.

### Shotgun mass spectrometry proteomics

Cell lysates were reduced using 2.5 μL of dithiothreitol (200 mM DTT stock solution) (Sigma) at 90 °C for 20 min, and alkylated with 10 μl of Cysteine Blocking Reagent (Iodoacetamide, IAM, 200 mM Sigma) for 1 h at room temperature in the dark. Trypsin (Promega, Sequence Grade) was added and digestion was performed overnight at 37 °C. Then, peptides digests were desalted on the Discovery® DSC-18 solid-phase extraction (SPE) 96-well Plate (25 mg/well) (Sigma-Aldrich Inc., St. Louis, MO, USA) and the samples were ready for the analysis^27^.

LC–MS/MS analyses were performed using a micro-LC Eksigent Technologies (Dublin, USA) system with a stationary phase of a Halo Fused C18 column (0.5 × 100 mm, 2.7 μm; Eksigent Technologies, Dublin, USA). The mobile phase was a mixture of 0.1% (v/v) formic acid in water (A) and 0.1% (v/v) formic acid in acetonitrile (B), eluting at a flow-rate of 15.0 μL min^−1^ at an increasing concentration of solvent B from 2 to 40% in 30 min. Samples used to generate the SWATH-MS (Sequential window acquisition of all theoretical mass spectra) spectral library were subjected to the traditional data-dependent acquisition (DDA): the mass spectrometer analysis was performed using a mass range of 100–1500 Da (TOF scan with an accumulation time of 0.25 s), followed by a MS/MS product ion scan from 200 to 1250 Da (accumulation time of 5.0 ms) with the abundance threshold set at 30 cps (35 candidate ions can be monitored during every cycle). Samples were then subjected to cyclic data independent analysis (DIA) of the mass spectra, using a 25-Da window. A 50-ms survey scan (TOF-MS) was performed, followed by MS/MS experiments on all precursors. These MS/MS experiments were performed in a cyclic manner using an accumulation time of 40 ms per 25-Da swath (36 swaths in total) for a total cycle time of 1.5408 s. The ions were fragmented for each MS/MS experiment in the collision cell using the rolling collision energy. The MS data were acquired with Analyst TF 1.7 (SCIEX, Concord, Canada). Three instrumental replicates for each sample were subjected to the DIA analysis^54,55^.

The mass spectrometry files were searched using Protein Pilot (AB SCIEX, Concord, Canada) and Mascot (Matrix Science Inc., Boston, USA). Samples were input in the Protein Pilot software v. 4.2 (AB SCIEX, Concord, Canada), with the following parameters: cysteine alkylation, digestion by trypsin, no special factors and False Discovery Rate at 1%. The UniProt Swiss-Prot reviewed database containing mouse proteins (version 12/10/2018, containing 25137 sequence entries). The Mascot search was performed on Mascot v. 2.4, the digestion enzyme selected was trypsin, with two missed cleavages and a search tolerance of 50 ppm was specified for the peptide mass tolerance, and 0.1 Da for the MS/MS tolerance. The charges of the peptides to search for were set to 2+, 3+ and 4+, and the search was set on monoisotopic mass. The instrument was set to ESI-QUAD-TOF and the following modifications were specified for the search: carbamidomethyl cysteines as fixed modification and oxidized methionine as variable modification.

The quantification was performed by integrating the extracted ion chromatogram of all the unique ions for a given peptide. The quantification was carried out with PeakView 2.0 and MarkerView 1.2. (Sciex, Concord, ON, Canada). Six peptides per protein and six transitions per peptide were extracted from the SWATH files. Shared peptides were excluded as well as peptides with modifications. Peptides with FDR lower than 1.0% were exported in MarkerView for the *t*-test.

### Fura-2 calcium imaging

iAstro lines, grown onto 24 mm round coverslips (3×10^4^ cell/coverslip), were loaded with 2.5 μM Fura-2/AM (Cat. No. F1201, Life Technologies, Milan, Italy) in the presence of 0.005% Pluronic F-127 (Cat. No. P6867, Life Technologies) and 10 μM sulfinpyrazone (Cat. S9509, Sigma) in KRB solution (125 mM NaCl, 5 mM KCl, 1 mM Na_3_PO_4_, 1 mM MgSO_4_, 5.5 mM glucose, 20 mM HEPES, pH 7.4) supplemented with 2 mM CaCl_2_. After loading (30 min in a dark place) cells were washed once with KRB solution and allowd to de-esterify for 30 min. After this the coverslips were mounted in an acquisition chamber and placed on the stage of a Leica epifluorescence microscope equipped with a S Fluor 40×/1.3 objective. Cells were alternatively excited at 340/380 nm by the monochromator Polichrome V (Till Photonics, Munich, Germany) and the fluorescent signal was collected by a CCD camera (Hamamatsu, Japan) through bandpass 510 nm filter. The fluorescent signals were captured by MetaFluor (Molecular Devices, Sunnyvale, CA, USA) software. The cells were stimulated by 20 μM ATP. To quantify the difference in the amplitude of Ca^2+^ transients, the ratio values were normalized according to the formula (ΔF)/F0 (referred to as norm. Fura ratio).

### Generation of stable iAstro lines expressing MIT-AEQ and ER-AEQ

For measurement of Ca2+ inside the ER and mitochondria, either mitochondrially or ER-targeted photoprotein aequorin (MIT-AEQ and ER-AEQ probes, respectively) were used. The details of generation of producation of lentiviral vectors expressing aequorin Ca^2+^ probes is described elswhere^34^. 24 h after plating (10E4 cell/well in 24-well plate), WT- and Tg-iAstros were infected with lentiviral particles experssing MIT-AEQ and ER-AEQ at MOI from 5 to 20. The dilution of virus that gave more than 50% of infected cells, as was detected by fluorescence of reporter proteins^34^, were further processed. Upon reaching confluence, the cells were expanded, and MIT-AEQ and ER-AEQ expressing WT- and 3Tg-iAstro cells were inriched using fluorescence-activated cell sorting (S3e Cell Sorter, Bio-Rad, Segrate, Milano). Sorted cells were expanded and frozen until needed.

### Aequorin Ca^2+^ measurements

For Ca^2+^ measurement in the ER lumen, WT- and 3Tg-iAstro cells expressing ER-AEQ Ca^2+^ sensor were plated on 13 mm round coverslips placed in 24-well plates (5 × 10^4^ cell/well). 24 h after plating che cells were reconstituted in modified Krebs–Ringer buffer (KRB, 135 mM NaCl, 5 mM KCl, 0.4 mM KH2PO4, 1 mM MgSO4, 5.5 mM glucose, 20 mM HEPES (pH 7.4) supplemented with 600 μM EGTA, 5 μM coelenterazine-n (low light-emitting coelenterazine variant) and 3 μM ionomycin (all reagents from Sigma) for 1 h at 4 °C. After reconstitution the cells were washed three times with KRB containing 600 μM EGTA and 2% BSA, followed by three washes with KRB containing 100 μM EGTA, after which the coverslips were trans-ferred into perfusion chamber of a custom-built aequorinometer (CAIRN research, UK). The cells were perfused with KRB supplemented with 100 μM EGTA at 37 °C. After 3 min of baseline recording the perfusion solution was switched to KRB supplemented with 2 mM Ca^2+^ and recording continued until the [Ca^2+^] in the ER did reach the steady-state level. At the end of each experiment, for quantification of the ER Ca^2+^ levels, the cells were perfused with distilled water containing 100 mM Ca^2+^ and 0.1% Triton X-100 to disrupt cells and discharge the remaining AEQ pool.

For assessment of ATP-induced Ca^2+^ transients in the mitochondrial matrix, MIT-AEQ-expressing WT- and 3Tg-iAstro cells were reconstituted with coelenterazine wt (5 μM) in complete culture medium in 37 °C and 5% CO2 for 1 h. After washing (3 times) with KRB supplemented with 2 mM Ca^2+^, cells were transferred into perfusion chamber of a custom-built aequorinometer and acquisition started in perfutio with KRB supplemented with 2 mM Ca^2+^. After baseline recording, perfusion was switched to KRB supplemented with 2 mM Ca^2+^ and 20 μM ATP. At the end of each experiment, for quantification of the ER Ca^2+^ levels, the cells were perfused with distilled water containing 100 mM Ca^2+^ and 0.1% Triton X-100 to disrupt cells and discharge the remaining AEQ pool. The light signals were calibrated off-line into [Ca^2+^] values using an algorithm described in^34^.

### Visualization of mitochondria-ER contact sites

Mitochondria-ER contact sites were visualized using SPLICS probe as described in^15^. Briefly, WT-iAstro and 3Tg-iAstro were plated onto 13 mm glass coverslips in 24-well plates (3x10E4 cells/well). 24 h after plating cells were transfected with pairs of plasmids SPLICS-Mit-Short + SPLICS-ER (hereafter referred as SPLICS-Short) and SPLICS-Mit-Long + SPLICS-ER (hereafter referred as SPLICS-Long) in 1:1 ratio using Lipofectamine 2000 (Thermofisher, Milan, Italy). 24 h after transfection cells were washed with PBS and fixed in 4% formaldehyde (Sigma, Milan, Italy). Fluorescent image stacks were taken using Zeiss 710 confocal laser scanning microscope equipped with Plan-Apochromat 63x/1.40 Oil M27 objective and Zen software. Image stacks were processed offline to major intensity projections. Thresholding and area calculations were done using Fiji package of ImageJ software v.1.52p. Data are expressed as SPLICS/cell area ratio.

### RNA extractiona and real-time PCR

Total RNA was extract by using the TripleXtractor reagent (Bio-Cell) as indicated by the supplier, and the AMV Reverse Transcriptase kit (Promega) was used to produce cDNA following the manufacturer’s recommendations, by using 2 μg of total RNA. Quantitative PCR reactions were performed by using the Bio-Rad CFX96 qPCR thermocycler. The primer pair sequences for selected amplicons were designed using the online IDT PrimerQuest Tool software (IDT; https://eu.idtdna.com/Primerquest/Home/Index). Primer sequences are available in Supplementary Table 1. Gapdh mRNA level was used as an internal control and results were expressed as previously described^56^.

### Puromycin incorporation method (Surface sensing of translation, SUnSET)

iAstro-WT and TG were incubated with 4 µM puromycin dihydrochloride (Sigma Cat. P8833) supplemented in normal medium at 37 °C with 5% CO2 for 3 h^36^. Subsequently, cells lysates were subjected to Western blot assay.

### Cell lysis and western blot

iAstro-cultures were lysed with 50 µL of lysis buffer (50 mM TrisHCl (pH 67.4), sodium dodecyl sulphate (SDS) 0.5%, 5 mM EDTA), 5 µL of protease inhibitors cocktail (PIC, Calbiochem) and 1 µL of phosphatase inhibitor Na_3_VO_4_ 1 M; removed with a scraper, and collected in a 1,5 ml tube. Lysates were then boiled for 5’, and quantified with QuantiPro BCA Assay Kit (Sigma, cat. SLBF3463).

30 µg of proteins were mixed with the right amount of Laemmli Sample Buffer 4X (Bio-Rad), and boiled for 7’. Then samples were loaded on a 12% polyacrylamide-sodium dodecyl sulphate gel for electrophoresis. Proteins were transferred onto nitrocellulose membrane, using Mini Transfer Packs or Midi Transfer Packs, with Trans-Blot® Turbo TM (Bio-Rad) according to manufacturer’s instructions (Bio-Rad).

The membrane was blocked in 5% skim milk (Sigma, Cat. 70166) for 45’ at room temperature. Subsequently, membrane was incubated with indicated primary antibody, overnight at 4 °C in agitation. Primary antibodies used were: anti-puromycin (1:1000, Millipore, Cat. MABE343), and ponceau staining was used to normalize protein load. Goat anti-mouse IgG (H + L) horseradisch peroxidase-conjugated secondary antibody (1:5000; Cat. 170-6516, Bio-Rad) and Goat anti-mouse Igg (H + L) horseradisch peroxidase-conjugated secondary antibody (1:5000; Cat. 170-6515, Bio-Rad). Detection was carried out with SuperSignalTM West Pico PLUS Chemiluminescent Substrate (Thermo Scientific), based on the chemiluminescence of luminol and developed using ChemiDocTM Imaging System (Bio-Rad). Quantitative densitometry of protein bands analysis was performed with ImageLab software.

### Statistical analysis

The data for mitochondrial and glycolytic efficiency and ROS production (Figs. 1 and 2 except Fig. 2e) are presented as mean ± SD; statistical analysis was performed by SigmaPlot vs.13 (Systat Software, Slough, UK). Other data (Fig. 2e; Figs. 6–8) are presented as mean ± SEM and were analyzed using GraphPad Prism v.7. No samples/results were excluded from the analysis. Unpaired two-tailed Student’s *t* test was used in all experiments and differences were considered significant at *p* < 0.05.

## Supplementary information

Supplemental Material

Supplemental Table 1a

Supplemental Table 1b

Supplemental Table 2

Supplemental Table 3a

Supplemental Table 3b

Supplemental Table 3c

Supplemental Table 3d
