# Supplementary material for: Proteomic analysis links alterations of bioenergetics, mitochondria-ER interactions and proteostasis in hippocampal astrocytes from 3xTg-AD mice
Source: Cell Death Dis. 2020 Aug 18;11(8):645. doi: 10.1038/s41419-020-02911-1 (PMC7434916; doi:10.1038/s41419-020-02911-1)
Supplement: Supplementary file 1 — Supplemental Material [file 41419_2020_2911_MOESM1_ESM.pdf]

Supplementary Methods and Figure

Proteomic analysis links alterations of bioenergetics, mitochondria-ER interactions and proteostasis in hippocampal astrocytes from 3xTg-AD mice

Giulia Dematteis[1]*, Gabrielė Vydmantaitė[2]*, Federico Alessandro Ruffinatti[1], Malak Chahin[1], Serena Farruggio[3], Elettra Barberis[3,4], Eleonora Ferrari[4,5], Emilio Marengo[6], Carla Distasi[1], Ramunė Morkūnienė[7], Armando A Genazzani[1], Mariagrazia Grilli[1], Elena Grossini[3], Marco Corazzari[4,5,8], Marcello Manfredi[3,4], Dmitry Lim[1#], Aistė Jekabsone[2#], Laura Tapella[1]

[1] Department of Pharmaceutical Sciences, Università degli Studi del Piemonte Orientale, Novara, Italy
[2] Institute of Pharmaceutical Technologies, Faculty of Pharmacy, Medical Academy, Lithuanian University of Health Sciences, Kaunas, Lithuania.
[3] Department of Translational Medicine, University of Piemonte Orientale, Novara, Italy.
[4] Center for Translational Research on Autoimmune and Allergic Diseases (CAAD), University of Piemonte Orientale, Novara, Italy.
[5] Department of Health Science, University of Piemonte Orientale, Novara, Italy
[6] DiSIT, University of Piemonte Orientale, Alessandria, Italy.
[7] Department of Drug Chemistry, Faculty of Pharmacy, Medical Academy, Lithuanian University of Health Sciences, Kaunas, Lithuania.
[8] Interdisciplinary Research Center of Autoimmune Diseases (IRCAD), University of Piemonte Orientale, Novara, Italy.

* These Authors contributed equally
# Correspondence should be sent to: Dmitry Lim, dmitry.lim@uniupo.it, Tel.: +39-0321 375822; Aisté Jekabsone, Aiste.Jekabsone@lsmuni.lt.

Index

Content	Page
Supplementary Methods. Table 1. **List of oligonucleotide primers used for qPCR**	**2**
Supplementary Figure 1. **MitoSOX fluorescence in WT-iAstro and 3Tg-iAstro cells**	**3**

Supplementary Table 1. List of oligonucleotide primers used in this work.

**Gene**	**Accession number**	**Forward Reverse**	**Sequence 5' to 3'**
Atf4	NM_009716.3	Forward<br>Reverse	GTTTAGAGCTAGGCAGTGAAG<br>CCTTTACACATGGAGGGATTAG
Atf6	NM_001081304.1	Forward<br>Reverse	GATGGTGACAACCAGAAAGA<br>TGGAGGTGGAGGCATATAA
Xbp1(s) (Xbp1)	NM_001271730.1	Forward<br>Reverse	AGTCCGCAGCAGGTG<br>GGTCCAACTTGTCCAGAATG
Herpud1 (Herp)	NM_022331.2	Forward<br>Reverse	GTGGAGGAAGATGATGAGATAAA<br>CTCAGCGAGGAGTAGAAGTA
GAPDH	NM_001289726.1	Forward<br>Reverse	TTCAACGGCACAGTCAAG<br>CCAGTAGACTCCACGACATA

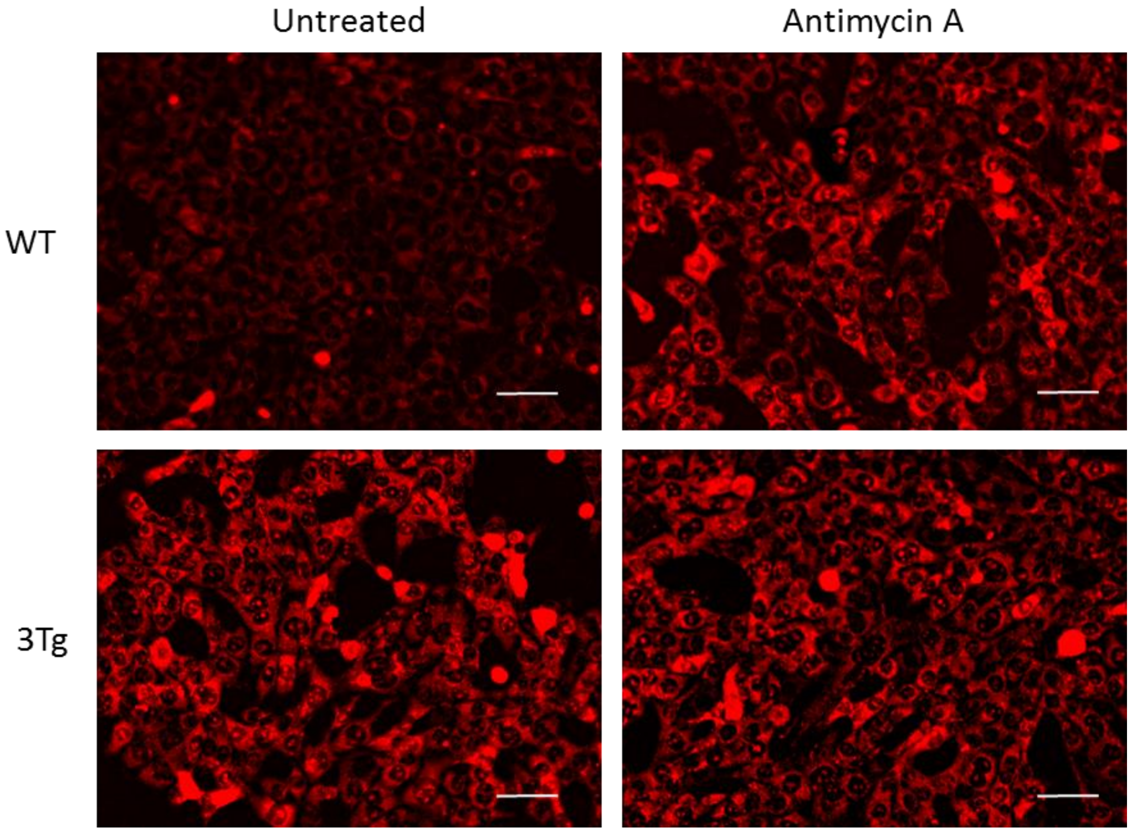

Supplementary Figure 1 Representative images of MitoSOX fluorescence in WT-iAstro (WT) and 3Tg-iAstro (3Tg) cells used for quantitative evaluation of mitochondrial superoxide. The microscope images of the same size were taken 30 min after loading with mitoSOX Red. In parallel, 30 min treatment with 100 μM Antimycin A was performed for a positive control of the assay. Scale bar is 100 μm.
