# Supplementary material for: Proteomic analysis links alterations of bioenergetics, mitochondria-ER interactions and proteostasis in hippocampal astrocytes from 3xTg-AD mice
Source: Cell Death Dis. 2020 Aug 18;11(8):645. doi: 10.1038/s41419-020-02911-1 (PMC7434916; doi:10.1038/s41419-020-02911-1)
Supplement: Supplementary file 2 — Supplemental Table 1a [file 41419_2020_2911_MOESM2_ESM.pdf]

Supplementary Table 1a. Identified proteins in MERE fraction of WT-iAstro and 3Tg-iAstro cells.

N = 5 biological replicates were used for each genotype

3Tg and/or WT	Num	Uniprot_ID	Description	Gene
3Tg and WT	777	RL22_MOUSE	60S ribosomal protein L22 (Heparin-binding protein HBp15)	Rpl22
3Tg and WT		TRXR2_MOUSE	Thioredoxin reductase 2, mitochondrial (EC 1.8.1.9) (Thioredoxin reductase TR3)	Txnrd2
3Tg and WT		VASP_MOUSE	Vasodilator-stimulated phosphoprotein (VASP)	Vasp
3Tg and WT		H14_MOUSE	Histone H1.4 (H1 VAR.2) (H1e)	H1-4
3Tg and WT		IF2H_MOUSE	Eukaryotic translation initiation factor 2 subunit 3, Y-linked (Eukaryotic translation initiation factor 2 subunit gamma, Y-linked) (eIF-2-gamma Y) (Spermatogonial proliferation factor) (Spy)	Eif2s3y
3Tg and WT		CYTSB_MOUSE	Cytospin-B (Sperm antigen with calponin homology and coiled-coil domains 1)	Specc1
3Tg and WT		VA0D1_MOUSE	V-type proton ATPase subunit d 1 (V-ATPase subunit d 1) (P39) (Physophilin) (V-ATPase 40 kDa accessory protein) (V-ATPase AC39 subunit) (Vacuolar proton pump subunit d 1)	Atp6v0d1
3Tg and WT		CLH1_MOUSE	Clathrin heavy chain 1	Cltc
3Tg and WT		RS24_MOUSE	40S ribosomal protein S24	Rps24
3Tg and WT		IMB1_MOUSE	Importin subunit beta-1 (Karyopherin subunit beta-1) (Nuclear factor p97) (Pore targeting complex 97 kDa subunit) (PTAC97) (SCG)	Kpnb1
3Tg and WT		EHD4_MOUSE	EH domain-containing protein 4 (PAST homolog 2) (mPAST2)	Ehd4
3Tg and WT		RS27L_MOUSE	40S ribosomal protein S27-like	Rps27l
3Tg and WT		RS13_MOUSE	40S ribosomal protein S13	Rps13
3Tg and WT		AT1A1_MOUSE	Sodium/potassium-transporting ATPase subunit alpha-1 (Na(+)/K(+) ATPase alpha-1 subunit) (EC 7.2.2.13) (Sodium pump subunit alpha-1)	Atp1a1
3Tg and WT		PRS6A_MOUSE	26S proteasome regulatory subunit 6A (26S proteasome AAA-ATPase subunit RPT5) (Proteasome 26S subunit ATPase 3) (Tat-binding protein 1) (TBP-1)	Psmc3
3Tg and WT		UBC12_MOUSE	NEDD8-conjugating enzyme Ubc12 (EC 2.3.2.-) (NEDD8 carrier protein) (Ubiquitin-conjugating enzyme E2 M)	Ube2m
3Tg and WT		NPTN_MOUSE	Neuroplastin (Stromal cell-derived receptor 1) (SDR-1)	Nptn
3Tg and WT		SAHH2_MOUSE	S-adenosylhomocysteine hydrolase-like protein 1 (IP3R-binding protein released with inositol 1,4,5-trisphosphate) (Putative adenosylhomocysteinase 2) (S-adenosyl-L-homocysteine hydrolase 2) (AdoHcyase 2)	Ahcyl1
3Tg and WT		EHD1_MOUSE	EH domain-containing protein 1 (PAST homolog 1) (mPAST1)	Ehd1
3Tg and WT		NMT1_MOUSE	Glycylpeptide N-tetradecanoyltransferase 1 (EC 2.3.1.97) (Myristoyl-CoA:protein N-myristoyltransferase 1) (NMT 1) (Type I N-myristoyltransferase) (Peptide N-myristoyltransferase 1)	Nmt1
3Tg and WT		CATD_MOUSE	Cathepsin D (EC 3.4.23.5)	Ctsd
3Tg and WT		RS3_MOUSE	40S ribosomal protein S3 (EC 4.2.99.18)	Rps3

3Tg and WT		UCHL1_MOUSE	Ubiquitin carboxyl-terminal hydrolase isozyme L1 (UCH-L1) (EC 3.4.19.12) (Neuron cytoplasmic protein 9.5) (PGP 9.5) (PGP9.5) (Ubiquitin thioesterase L1)	Uchl1
3Tg and WT		RS2_MOUSE	40S ribosomal protein S2 (40S ribosomal protein S4) (Protein LLRep3)	Rps2
3Tg and WT		TAGL2_MOUSE	Transgelin-2 (SM22-beta)	Tagln2
3Tg and WT		TCPH_MOUSE	T-complex protein 1 subunit eta (TCP-1-eta) (CCT-eta)	Cct7
3Tg and WT		MYO10_MOUSE	Unconventional myosin-X (Unconventional myosin-10)	Myo10
3Tg and WT		TOM20_MOUSE	Mitochondrial import receptor subunit TOM20 homolog (Mitochondrial 20 kDa outer membrane protein) (Outer mitochondrial membrane receptor Tom20)	Tomm20
3Tg and WT		S29A1_MOUSE	Equilibrative nucleoside transporter 1 (Equilibrative nitrobenzylmercaptopurine riboside-sensitive nucleoside transporter) (Equilibrative NBMPR-sensitive nucleoside transporter) (Nucleoside transporter, es-type) (Solute carrier family 29 member 1)	Slc29a1
3Tg and WT		PGK1_MOUSE	Phosphoglycerate kinase 1 (EC 2.7.2.3)	Pgk1
3Tg and WT		H2B2B_MOUSE	Histone H2B type 2-B (H2b 616)	Hist2h2bb
3Tg and WT		MYL9_MOUSE	Myosin regulatory light polypeptide 9 (Myosin regulatory light chain 2, smooth muscle isoform) (Myosin regulatory light chain 9)	Myl9
3Tg and WT		PLCB3_MOUSE	1-phosphatidylinositol 4,5-bisphosphate phosphodiesterase beta-3 (EC 3.1.4.11) (Phosphoinositide phospholipase C-beta-3) (Phospholipase C-beta-3) (PLC-beta-3)	Plcb3
3Tg and WT		MUC18_MOUSE	Cell surface glycoprotein MUC18 (Gicerin) (Melanoma cell adhesion molecule) (Melanoma-associated antigen MUC18) (CD antigen CD146)	Mcam
3Tg and WT		CYC_MOUSE	Cytochrome c, somatic	Cycs
3Tg and WT		CAZA2_MOUSE	F-actin-capping protein subunit alpha-2 (CapZ alpha-2)	Capza2
3Tg and WT		GLU2B_MOUSE	Glucosidase 2 subunit beta (80K-H protein) (Glucosidase II subunit beta) (Protein kinase C substrate 60.1 kDa protein heavy chain) (PKCSH)	Prkcsh
3Tg and WT		UBA1_MOUSE	Ubiquitin-like modifier-activating enzyme 1 (EC 6.2.1.45) (Ubiquitin-activating enzyme E1) (Ubiquitin-activating enzyme E1 X) (Ubiquitin-like modifier-activating enzyme 1 X)	Uba1
3Tg and WT		RRAS_MOUSE	Ras-related protein R-Ras (p23)	Rras
3Tg and WT		IPYR_MOUSE	Inorganic pyrophosphatase (EC 3.6.1.1) (Pyrophosphate phospho-hydrolase) (PPase)	Ppa1
3Tg and WT		ERP29_MOUSE	Endoplasmic reticulum resident protein 29 (ERp29)	Erp29
3Tg and WT		RS5_MOUSE	40S ribosomal protein S5 [Cleaved into: 40S ribosomal protein S5, N-terminally processed]	Rps5
3Tg and WT		SEPT5_MOUSE	Septin-5 (Cell division control-related protein 1) (CDCrel-1) (Peanut-like protein 1)	Septin5
3Tg and WT		BAF_MOUSE	Barrier-to-autointegration factor (Breakpoint cluster region protein 1) (LAP2-binding protein 1) [Cleaved into: Barrier-to-autointegration factor, N-terminally processed]	Banf1
3Tg and WT		TLN2_MOUSE	Talin-2	Tln2

3Tg and WT		AP2S1_MOUSE	AP-2 complex subunit sigma (Adaptor protein complex AP-2 subunit sigma) (Adaptor-related protein complex 2 subunit sigma) (Clathrin assembly protein 2 sigma small chain) (Clathrin coat assembly protein AP17) (Clathrin coat-associated protein AP17) (Plasma membrane adaptor AP-2 17 kDa protein) (Sigma-adaptin 3b) (Sigma2-adaptin)	Ap2s1
3Tg and WT		ESTD_MOUSE	S-formylglutathione hydrolase (FGH) (EC 3.1.2.12) (Esterase 10) (Esterase D) (Sid 478)	Esd
3Tg and WT		FXR1_MOUSE	Fragile X mental retardation syndrome-related protein 1 (mFxr1p)	Fxr1
3Tg and WT		RL17_MOUSE	60S ribosomal protein L17	Rpl17
3Tg and WT		VCAM1_MOUSE	Vascular cell adhesion protein 1 (V-CAM 1) (VCAM-1) (CD antigen CD106)	Vcam1
3Tg and WT		RS12_MOUSE	40S ribosomal protein S12	Rps12
3Tg and WT		RL15_MOUSE	60S ribosomal protein L15	Rpl15
3Tg and WT		IF5A2_MOUSE	Eukaryotic translation initiation factor 5A-2 (eIF-5A-2) (eIF-5A2) (Eukaryotic initiation factor 5A isoform 2)	Eif5a2
3Tg and WT		EF1A1_MOUSE	Elongation factor 1-alpha 1 (EF-1-alpha-1) (Elongation factor Tu) (EF-Tu) (Eukaryotic elongation factor 1 A-1) (eEF1A-1)	Eef1a1
3Tg and WT		DYL1_MOUSE	Dynein light chain 1, cytoplasmic (8 kDa dynein light chain) (DLC8) (Dynein light chain LC8-type 1) (Protein inhibitor of neuronal nitric oxide synthase) (PIN) (mPIN)	Dynll1
3Tg and WT		RAB13_MOUSE	Ras-related protein Rab-13	Rab13
3Tg and WT		ALDOA_MOUSE	Fructose-bisphosphate aldolase A (EC 4.1.2.13) (Aldolase 1) (Muscle-type aldolase)	Aldoa
3Tg and WT		XRP2_MOUSE	Protein XRP2	Rp2
3Tg and WT		H31_MOUSE	Histone H3.1	H3c1
3Tg and WT		DDX6_MOUSE	Probable ATP-dependent RNA helicase DDX6 (EC 3.6.4.13) (ATP-dependent RNA helicase p54) (DEAD box protein 6) (Oncogene RCK homolog)	Ddx6
3Tg and WT		FLNC_MOUSE	Filamin-C (FLN-C) (ABP-280-like protein) (ABP-L) (Actin-binding-like protein) (Filamin-2) (Gamma-filamin)	Flnc
3Tg and WT		TCPE_MOUSE	T-complex protein 1 subunit epsilon (TCP-1-epsilon) (CCT-epsilon)	Cct5
3Tg and WT		SORCN_MOUSE	Sorcin	Sri
3Tg and WT		P5CR3_MOUSE	Pyrroline-5-carboxylate reductase 3 (P5C reductase 3) (P5CR 3) (EC 1.5.1.2) (Pyrroline-5-carboxylate reductase-like protein)	Pycr3
3Tg and WT		RL10A_MOUSE	60S ribosomal protein L10a (CSA-19) (Neural precursor cell expressed developmentally down-regulated protein 6) (NEDD-6)	Rpl10a
3Tg and WT		AQP1_MOUSE	Aquaporin-1 (AQP-1) (Aquaporin-CHIP) (Delayed early response protein 2) (DER2) (Water channel protein for red blood cells and kidney proximal tubule)	Aqp1
3Tg and WT		UB2D3_MOUSE	Ubiquitin-conjugating enzyme E2 D3 (EC 2.3.2.23) ((E3-independent) E2 ubiquitin-conjugating enzyme D3) (EC 2.3.2.24) (E2 ubiquitin-conjugating enzyme D3) (Ubiquitin carrier protein D3) (Ubiquitin-conjugating enzyme E2(17)KB 3) (Ubiquitin-conjugating enzyme E2-17 kDa 3) (Ubiquitin-protein ligase D3)	Ube2d3

3Tg and WT		RACK1_MOUSE	Receptor of activated protein C kinase 1 (12-3) (Guanine nucleotide-binding protein subunit beta-2-like 1) (Receptor for activated C kinase) (Receptor of activated protein kinase C 1) (p205) [Cleaved into: Receptor of activated protein C kinase 1, N-terminally processed (Guanine nucleotide-binding protein subunit beta-2-like 1, N-terminally processed)]	Rack1
3Tg and WT		FBX41_MOUSE	F-box only protein 41	Fbxo41
3Tg and WT		TCPD_MOUSE	T-complex protein 1 subunit delta (TCP-1-delta) (A45) (CCT-delta)	Cct4
3Tg and WT		ZN579_MOUSE	Zinc finger protein 579	Znf579
3Tg and WT		RAB35_MOUSE	Ras-related protein Rab-35	Rab35
3Tg and WT		SC61B_MOUSE	Protein transport protein Sec61 subunit beta	Sec61b
3Tg and WT		EF2_MOUSE	Elongation factor 2 (EF-2)	Eef2
3Tg and WT		DDX3L_MOUSE	Putative ATP-dependent RNA helicase Pl10 (EC 3.6.4.13)	D1Pas1
3Tg and WT		LAMP1_MOUSE	Lysosome-associated membrane glycoprotein 1 (LAMP-1) (Lysosome-associated membrane protein 1) (120 kDa lysosomal membrane glycoprotein) (CD107 antigen-like family member A) (LGP-120) (Lysosomal membrane glycoprotein A) (LGP-A) (P2B) (CD antigen CD107a)	Lamp1
3Tg and WT		FLNA_MOUSE	Filamin-A (FLN-A) (Actin-binding protein 280) (ABP-280) (Alpha-filamin) (Endothelial actin-binding protein) (Filamin-1) (Non-muscle filamin)	Flna
3Tg and WT		BASI_MOUSE	Basigin (Basic immunoglobulin superfamily) (HT7 antigen) (Membrane glycoprotein gp42) (CD antigen CD147)	Bsg
3Tg and WT		PLP2_MOUSE	Proteolipid protein 2	Plp2
3Tg and WT		RS10_MOUSE	40S ribosomal protein S10	Rps10
3Tg and WT		MDHC_MOUSE	Malate dehydrogenase, cytoplasmic (EC 1.1.1.37) (Cytosolic malate dehydrogenase)	Mdh1
3Tg and WT		DX39B_MOUSE	Spliceosome RNA helicase Ddx39b (EC 3.6.4.13) (56 kDa U2AF65-associated protein) (DEAD box protein UAP56) (HLA-B-associated transcript 1 protein)	Ddx39b
3Tg and WT		PRDX1_MOUSE	Peroxiredoxin-1 (EC 1.11.1.15) (Macrophage 23 kDa stress protein) (Osteoblast-specific factor 3) (OSF-3) (Thioredoxin peroxidase 2) (Thioredoxin-dependent peroxide reductase 2)	Prdx1
3Tg and WT		CALX_MOUSE	Calnexin	Canx
3Tg and WT		ITB1_MOUSE	Integrin beta-1 (Fibronectin receptor subunit beta) (VLA-4 subunit beta) (CD antigen CD29)	Itgb1
3Tg and WT		PRDX4_MOUSE	Peroxiredoxin-4 (EC 1.11.1.15) (Antioxidant enzyme AOE372) (Peroxiredoxin IV) (Prx-IV) (Thioredoxin peroxidase AO372) (Thioredoxin-dependent peroxide reductase A0372)	Prdx4
3Tg and WT		VPS35_MOUSE	Vacuolar protein sorting-associated protein 35 (Maternal-embryonic 3) (Vesicle protein sorting 35)	Vps35
3Tg and WT		SDCB1_MOUSE	Syntenin-1 (Scaffold protein Pbp1) (Syndecan-binding protein 1)	Sdcbp
3Tg and WT		GLRX1_MOUSE	Glutaredoxin-1 (Thioltransferase-1) (TTase-1)	Glrx
3Tg and WT		CHM4B_MOUSE	Charged multivesicular body protein 4b (Chromatin-modifying protein 4b) (CHMP4b)	Chmp4b
3Tg and WT		RS18_MOUSE	40S ribosomal protein S18 (Ke-3) (Ke3)	Rps18
3Tg and WT		HYOU1_MOUSE	Hypoxia up-regulated protein 1 (GRP-170) (140 kDa Ca(2+)-binding protein) (CBP-140)	Hyou1

3Tg and WT		RS17_MOUSE	40S ribosomal protein S17	Rps17
3Tg and WT		STXB1_MOUSE	Syntaxin-binding protein 1 (Protein unc-18 homolog 1) (Unc18-1) (Protein unc-18 homolog A) (Unc-18A)	Stxbp1
3Tg and WT		DPYL3_MOUSE	Dihydropyrimidinase-related protein 3 (DRP-3) (Unc-33-like phosphoprotein 1) (ULIP-1)	Dpysl3
3Tg and WT		CAZA1_MOUSE	F-actin-capping protein subunit alpha-1 (CapZ alpha-1)	Capza1
3Tg and WT		SHPS1_MOUSE	Tyrosine-protein phosphatase non-receptor type substrate 1 (SHP substrate 1) (SHPS-1) (Brain Ig-like molecule with tyrosine-based activation motifs) (Bit) (CD172 antigen-like family member A) (Inhibitory receptor SHPS-1) (MyD-1 antigen) (Signal-regulatory protein alpha-1) (Sirp-alpha-1) (mSIRP-alpha1) (p84) (CD antigen CD172a)	Sirpa
3Tg and WT		GNAQ_MOUSE	Guanine nucleotide-binding protein G(q) subunit alpha (Guanine nucleotide-binding protein alpha-q)	Gnaq
3Tg and WT		JAM3_MOUSE	Junctional adhesion molecule C (JAM-C) (JAM-2) (Junctional adhesion molecule 3) (JAM-3) [Cleaved into: Soluble form of JAM-C (sJAM-C)]	Jam3
3Tg and WT		AMPL_MOUSE	Cytosol aminopeptidase (EC 3.4.11.1) (Leucine aminopeptidase 3) (LAP-3) (Leucyl aminopeptidase) (Proline aminopeptidase) (EC 3.4.11.5) (Prolyl aminopeptidase)	Lap3
3Tg and WT		AOFA_MOUSE	Amine oxidase [flavin-containing] A (EC 1.4.3.4) (Monoamine oxidase type A) (MAO-A)	Maoa
3Tg and WT		ROA2_MOUSE	Heterogeneous nuclear ribonucleoproteins A2/B1 (hnRNP A2/B1)	Hnrnpa2b1
3Tg and WT		STOM_MOUSE	Erythrocyte band 7 integral membrane protein (Protein 7.2b) (Stomatin)	Stom
3Tg and WT		THIO_MOUSE	Thioredoxin (Trx) (ATL-derived factor) (ADF)	Txn
3Tg and WT		FHL1_MOUSE	Four and a half LIM domains protein 1 (FHL-1) (KyoT) (RBP-associated molecule 14-1) (RAM14-1) (Skeletal muscle LIM-protein 1) (SLIM) (SLIM-1)	Fhl1
3Tg and WT		MYPT1_MOUSE	Protein phosphatase 1 regulatory subunit 12A (Myosin phosphatase-targeting subunit 1) (Myosin phosphatase target subunit 1)	Ppp1r12a
3Tg and WT		SERPH_MOUSE	Serpin H1 (47 kDa heat shock protein) (Collagen-binding protein) (Colligin) (Serine protease inhibitor J6)	Serpinh1
3Tg and WT		RS16_MOUSE	40S ribosomal protein S16	Rps16
3Tg and WT		VIME_MOUSE	Vimentin	Vim
3Tg and WT		GANAB_MOUSE	Neutral alpha-glucosidase AB (EC 3.2.1.207) (Alpha-glucosidase 2) (Glucosidase II subunit alpha)	Ganab
3Tg and WT		PYRG1_MOUSE	CTP synthase 1 (EC 6.3.4.2) (CTP synthetase 1) (UTP--ammonia ligase 1)	Ctps1
3Tg and WT		EHD2_MOUSE	EH domain-containing protein 2	Ehd2
3Tg and WT		RL21_MOUSE	60S ribosomal protein L21	Rpl21
3Tg and WT		MACF1_MOUSE	Microtubule-actin cross-linking factor 1 (Actin cross-linking family 7)	Macf1
3Tg and WT		RHEB_MOUSE	GTP-binding protein Rheb (Ras homolog enriched in brain)	Rheb
3Tg and WT		AT1B1_MOUSE	Sodium/potassium-transporting ATPase subunit beta-1 (Sodium/potassium-dependent ATPase subunit beta-1)	Atp1b1

3Tg and WT		PRDX6_MOUSE	Peroxiredoxin-6 (EC 1.11.1.15) (1-Cys peroxiredoxin) (1-Cys PRX) (Acidic calcium-independent phospholipase A2) (aiPLA2) (EC 3.1.1.4) (Antioxidant protein 2) (Non-selenium glutathione peroxidase) (NSGPx)	Prdx6
3Tg and WT		RAB5C_MOUSE	Ras-related protein Rab-5C	Rab5c
3Tg and WT		VDAC2_MOUSE	Voltage-dependent anion-selective channel protein 2 (VDAC-2) (mVDAC2) (Outer mitochondrial membrane protein porin 2) (Voltage-dependent anion-selective channel protein 6) (VDAC-6) (mVDAC6)	Vdac2
3Tg and WT		RALA_MOUSE	Ras-related protein Ral-A	Rala
3Tg and WT		GBG5_MOUSE	Guanine nucleotide-binding protein G(I)/G(S)/G(O) subunit gamma-5	Gng5
3Tg and WT		CALM1_MOUSE	Calmodulin-1	Calm1
3Tg and WT		ANXA3_MOUSE	Annexin A3 (35-alpha calcimedin) (Annexin III) (Annexin-3) (Lipocortin III) (Placental anticoagulant protein III) (PAP-III)	Anxa3
3Tg and WT		RS3A_MOUSE	40S ribosomal protein S3a (Protein TU-11)	Rps3a
3Tg and WT		THIL_MOUSE	Acetyl-CoA acetyltransferase, mitochondrial (EC 2.3.1.9) (Acetoacetyl-CoA thiolase)	Acat1
3Tg and WT		ECHA_MOUSE	Trifunctional enzyme subunit alpha, mitochondrial (Monolysocardiolipin acyltransferase) (EC 2.3.1.-) (TP-alpha) [Includes: Long-chain enoyl-CoA hydratase (EC 4.2.1.17); Long chain 3-hydroxyacyl-CoA dehydrogenase (EC 1.1.1.211)]	Hadha
3Tg and WT		2AAA_MOUSE	Serine/threonine-protein phosphatase 2A 65 kDa regulatory subunit A alpha isoform (PP2A subunit A isoform PR65-alpha) (PP2A subunit A isoform R1-alpha)	Ppp2r1a
3Tg and WT		ITB5_MOUSE	Integrin beta-5	Itgb5
3Tg and WT		RL24_MOUSE	60S ribosomal protein L24	Rpl24
3Tg and WT		H12_MOUSE	Histone H1.2 (H1 VAR.1) (H1c)	H1-2
3Tg and WT		PSB1_MOUSE	Proteasome subunit beta type-1 (EC 3.4.25.1) (Macropain subunit C5) (Multicatalytic endopeptidase complex subunit C5) (Proteasome component C5) (Proteasome gamma chain)	Psmb1
3Tg and WT		K1C13_MOUSE	Keratin, type I cytoskeletal 13 (47 kDa cytokeratin) (Cytokeratin-13) (CK-13) (Keratin-13) (K13)	Krt13
3Tg and WT		ROA1_MOUSE	Heterogeneous nuclear ribonucleoprotein A1 (hnRNP A1) (HDP-1) (Helix-destabilizing protein) (Single-strand-binding protein) (Topoisomerase-inhibitor suppressed) (hnRNP core protein A1) [Cleaved into: Heterogeneous nuclear ribonucleoprotein A1, N-terminally processed]	Hnrnpa1
3Tg and WT		RL3_MOUSE	60S ribosomal protein L3 (J1 protein)	Rpl3
3Tg and WT		CRIP2_MOUSE	Cysteine-rich protein 2 (CRP-2) (Heart LIM protein)	Crip2
3Tg and WT		GLIS3_MOUSE	Zinc finger protein GLIS3 (GLI-similar 3)	Glis3
3Tg and WT		RL4_MOUSE	60S ribosomal protein L4	Rpl4
3Tg and WT		GSK3B_MOUSE	Glycogen synthase kinase-3 beta (GSK-3 beta) (EC 2.7.11.26) (Serine/threonine-protein kinase GSK3B) (EC 2.7.11.1)	Gsk3b
3Tg and WT		HMCS2_MOUSE	Hydroxymethylglutaryl-CoA synthase, mitochondrial (HMG-CoA synthase) (EC 2.3.3.10) (3-hydroxy-3-methylglutaryl coenzyme A synthase)	Hmgcs2
3Tg and WT		CTNA1_MOUSE	Catenin alpha-1 (102 kDa cadherin-associated protein) (Alpha E-catenin) (CAP102)	Ctnna1

3Tg and WT		SCOT1_MOUSE	Succinyl-CoA:3-ketoacid coenzyme A transferase 1, mitochondrial (EC 2.8.3.5) (3-oxoacid CoA-transferase 1) (Somatic-type succinyl-CoA:3-oxoacid CoA-transferase) (SCOT-s)	Oxct1
3Tg and WT		DYSF_MOUSE	Dysferlin (Dystrophy-associated fer-1-like protein) (Fer-1-like protein 1)	Dysf
3Tg and WT		TOLIP_MOUSE	Toll-interacting protein	Tollip
3Tg and WT		RS20_MOUSE	40S ribosomal protein S20	Rps20
3Tg and WT		CTNB1_MOUSE	Catenin beta-1 (Beta-catenin)	Ctnnb1
3Tg and WT		MATR3_MOUSE	Matrin-3	Matr3
3Tg and WT		MYADM_MOUSE	Myeloid-associated differentiation marker (Myeloid up-regulated protein)	Myadm
3Tg and WT		ANXA4_MOUSE	Annexin A4 (Annexin IV) (Annexin-4)	Anxa4
3Tg and WT		IF4A1_MOUSE	Eukaryotic initiation factor 4A-I (eIF-4A-I) (eIF4A-I) (EC 3.6.4.13) (ATP-dependent RNA helicase eIF4A-1)	Eif4a1
3Tg and WT		SH3L3_MOUSE	SH3 domain-binding glutamic acid-rich-like protein 3	Sh3bgrl3
3Tg and WT		CALM3_MOUSE	Calmodulin-3	Calm3
3Tg and WT		RS15_MOUSE	40S ribosomal protein S15 (RIG protein)	Rps15
3Tg and WT		LRC59_MOUSE	Leucine-rich repeat-containing protein 59 [Cleaved into: Leucine-rich repeat-containing protein 59, N-terminally processed]	Lrrc59
3Tg and WT		COR1B_MOUSE	Coronin-1B (Coronin-2)	Coro1b
3Tg and WT		IFM3_MOUSE	Interferon-induced transmembrane protein 3 (Dispanin subfamily A member 2b) (DSPA2b) (Fragilis protein) (Interferon-inducible protein 15) (Mouse ifitm-like protein 1) (Mil-1)	Ifitm3
3Tg and WT		SPTB2_MOUSE	Spectrin beta chain, non-erythrocytic 1 (Beta-II spectrin) (Embryonic liver fodrin) (Fodrin beta chain)	Sptbn1
3Tg and WT		TBB4B_MOUSE	Tubulin beta-4B chain (Tubulin beta-2C chain)	Tubb4b
3Tg and WT		TCPZ_MOUSE	T-complex protein 1 subunit zeta (TCP-1-zeta) (CCT-zeta-1)	Cct6a
3Tg and WT		AP2A1_MOUSE	AP-2 complex subunit alpha-1 (100 kDa coated vesicle protein A) (Adaptor protein complex AP-2 subunit alpha-1) (Adaptor-related protein complex 2 subunit alpha-1) (Alpha-adaptin A) (Alpha1-adaptin) (Clathrin assembly protein complex 2 alpha-A large chain) (Plasma membrane adaptor HA2/AP2 adaptin alpha A subunit)	Ap2a1
3Tg and WT		ERP44_MOUSE	Endoplasmic reticulum resident protein 44 (ER protein 44) (ERp44) (Thioredoxin domain-containing protein 4)	Erp44
3Tg and WT		GDIB_MOUSE	Rab GDP dissociation inhibitor beta (Rab GDI beta) (GDI-3) (Guanosine diphosphate dissociation inhibitor 2) (GDI-2)	Gdi2
3Tg and WT		PARK7_MOUSE	Protein/nucleic acid deglycase DJ-1 (EC 3.1.2.-) (EC 3.5.1.-) (EC 3.5.1.124) (Maillard deglycase) (Parkinson disease protein 7 homolog) (Parkinsonism-associated deglycase) (Protein DJ-1) (DJ-1)	Park7
3Tg and WT		CHD4_MOUSE	Chromodomain-helicase-DNA-binding protein 4 (CHD-4) (EC 3.6.4.12)	Chd4
3Tg and WT		PP1A_MOUSE	Serine/threonine-protein phosphatase PP1-alpha catalytic subunit (PP-1A) (EC 3.1.3.16)	Ppp1ca
3Tg and WT		TMEDA_MOUSE	Transmembrane emp24 domain-containing protein 10 (21 kDa transmembrane-trafficking protein) (Transmembrane protein Tmp21) (p24 family protein delta-1) (p24delta1)	Tmed10

3Tg and WT		PLXB2_MOUSE	Plexin-B2	Plxnb2
3Tg and WT		CNPY2_MOUSE	Protein canopy homolog 2 (MIR-interacting saposin-like protein) (Putative secreted protein ZSIG9) (Transmembrane protein 4)	Cnpy2
3Tg and WT		HYEP_MOUSE	Epoxide hydrolase 1 (EC 3.3.2.9) (Epoxide hydratase) (Microsomal epoxide hydrolase) (mEH)	Ephx1
3Tg and WT		EIF2A_MOUSE	Eukaryotic translation initiation factor 2A (eIF-2A) [Cleaved into: Eukaryotic translation initiation factor 2A, N-terminally processed]	Eif2a
3Tg and WT		TWF1_MOUSE	Twinfilin-1 (Protein A6)	Twf1
3Tg and WT		CDC42_MOUSE	Cell division control protein 42 homolog (EC 3.6.5.2) (G25K GTP-binding protein)	Cdc42
3Tg and WT		TSP1_MOUSE	Thrombospondin-1 (Glycoprotein G)	Thbs1
3Tg and WT		THY1_MOUSE	Thy-1 membrane glycoprotein (Thy-1 antigen) (CD antigen CD90)	Thy1
3Tg and WT		BAP31_MOUSE	B-cell receptor-associated protein 31 (BCR-associated protein 31) (Bap31) (p28)	Bcap31
3Tg and WT		COX6C_MOUSE	Cytochrome c oxidase subunit 6C (Cytochrome c oxidase polypeptide VIc)	Cox6c
3Tg and WT		RAP2B_MOUSE	Ras-related protein Rap-2b	Rap2b
3Tg and WT		MYO1C_MOUSE	Unconventional myosin-Ic (Myosin I beta) (MMI-beta) (MMIb)	Myo1c
3Tg and WT		IF2A_MOUSE	Eukaryotic translation initiation factor 2 subunit 1 (Eukaryotic translation initiation factor 2 subunit alpha) (eIF-2-alpha) (eIF-2A) (eIF-2alpha)	Eif2s1
3Tg and WT		X3CL1_MOUSE	Fractalkine (C-X3-C motif chemokine 1) (CX3C membrane-anchored chemokine) (Neurotactin) (Small-inducible cytokine D1) [Cleaved into: Processed fractalkine]	Cx3cl1
3Tg and WT		FPPS_MOUSE	Farnesyl pyrophosphate synthase (FPP synthase) (FPS) (EC 2.5.1.10) ((2E,6E)-farnesyl diphosphate synthase) (Cholesterol-regulated 39 kDa protein) (CR 39) (Dimethylallyltranstransferase) (EC 2.5.1.1) (Farnesyl diphosphate synthase) (Geranyltranstransferase)	Fdps
3Tg and WT		K2C1_MOUSE	Keratin, type II cytoskeletal 1 (67 kDa cytokeratin) (Cytokeratin-1) (CK-1) (Keratin-1) (K1) (Type-II keratin Kb1)	Krt1
3Tg and WT		RL39_MOUSE	60S ribosomal protein L39	Rpl39
3Tg and WT		AKAP2_MOUSE	A-kinase anchor protein 2 (AKAP-2) (AKAP expressed in kidney and lung) (AKAP-KL) (Protein kinase A-anchoring protein 2) (PRKA2)	Akap2
3Tg and WT		PLAK_MOUSE	Junction plakoglobin (Desmoplakin III) (Desmoplakin-3)	Jup
3Tg and WT		ACTA_MOUSE	Actin, aortic smooth muscle (Alpha-actin-2) [Cleaved into: Actin, aortic smooth muscle, intermediate form]	Acta2
3Tg and WT		MRP1_MOUSE	Multidrug resistance-associated protein 1 (EC 7.6.2.2) (ATP-binding cassette sub-family C member 1) (Glutathione-S-conjugate-translocating ATPase ABCC1) (EC 7.6.2.3) (Leukotriene C(4) transporter) (LTC4 transporter)	Abcc1
3Tg and WT		TECR_MOUSE	Very-long-chain enoyl-CoA reductase (EC 1.3.1.93) (Synaptic glycoprotein SC2) (Trans-2,3-enoyl-CoA reductase) (TER)	Tecr
3Tg and WT		CHIP_MOUSE	STIP1 homology and U box-containing protein 1 (EC 2.3.2.27) (Carboxy terminus of Hsp70-interacting protein) (E3 ubiquitin-protein ligase CHIP) (RING-type E3 ubiquitin transferase CHIP)	Stub1

3Tg and WT		MPP6_MOUSE	MAGUK p55 subfamily member 6 (Dlgh4 protein) (P55T protein) (Protein associated with Lin-7 2)	Mpp6
3Tg and WT		C1TM_MOUSE	Monofunctional C1-tetrahydrofolate synthase, mitochondrial (EC 6.3.4.3) (Formyltetrahydrofolate synthetase)	Mthfd1l
3Tg and WT		RS6_MOUSE	40S ribosomal protein S6 (Phosphoprotein NP33)	Rps6
3Tg and WT		PTMS_MOUSE	Parathymosin	Ptms
3Tg and WT		CD109_MOUSE	CD109 antigen (GPI-anchored alpha-2 macroglobulin-related protein) (CD antigen CD109)	Cd109
3Tg and WT		PPIA_MOUSE	Peptidyl-prolyl cis-trans isomerase A (PPIase A) (EC 5.2.1.8) (Cyclophilin A) (Cyclosporin A-binding protein) (Rotamase A) (SP18) [Cleaved into: Peptidyl-prolyl cis-trans isomerase A, N-terminally processed]	Ppia
3Tg and WT		TFR1_MOUSE	Transferrin receptor protein 1 (TR) (TfR) (TfR1) (Trfr) (CD antigen CD71)	Tfrc
3Tg and WT		CD47_MOUSE	Leukocyte surface antigen CD47 (Integrin-associated protein) (IAP) (CD antigen CD47)	Cd47
3Tg and WT		CDC37_MOUSE	Hsp90 co-chaperone Cdc37 (Hsp90 chaperone protein kinase-targeting subunit) (p50Cdc37) [Cleaved into: Hsp90 co-chaperone Cdc37, N-terminally processed]	Cdc37
3Tg and WT		SEP15_MOUSE	Selenoprotein F (15 kDa selenoprotein)	Selenof
3Tg and WT		DRG1_MOUSE	Developmentally-regulated GTP-binding protein 1 (DRG-1) (Neural precursor cell expressed developmentally down-regulated protein 3) (NEDD-3) (Translation factor GTPase DRG1) (TRAFAC GTPase DRG1) (EC 3.6.5.-)	Drg1
3Tg and WT		NUCL_MOUSE	Nucleolin (Protein C23)	Ncl
3Tg and WT		GAPR1_MOUSE	Golgi-associated plant pathogenesis-related protein 1 (GAPR-1) (Golgi-associated PR-1 protein) (Glioma pathogenesis-related protein 2) (GliPR 2)	Glipr2
3Tg and WT		SURF4_MOUSE	Surfeit locus protein 4	Surf4
3Tg and WT		SHLB1_MOUSE	Endophilin-B1 (SH3 domain-containing GRB2-like protein B1)	Sh3glb1
3Tg and WT		SYFB_MOUSE	Phenylalanine--tRNA ligase beta subunit (EC 6.1.1.20) (Phenylalanyl-tRNA synthetase beta subunit) (PheRS)	Farsb
3Tg and WT		COF2_MOUSE	Cofilin-2 (Cofilin, muscle isoform)	Cfl2
3Tg and WT		RL18A_MOUSE	60S ribosomal protein L18a	Rpl18a
3Tg and WT		IMA1_MOUSE	Importin subunit alpha-1 (Importin alpha P1) (Karyopherin subunit alpha-2) (Pendulin) (Pore targeting complex 58 kDa subunit) (PTAC58) (RAG cohort protein 1) (SRP1-alpha)	Kpna2
3Tg and WT		PPIC_MOUSE	Peptidyl-prolyl cis-trans isomerase C (PPIase C) (EC 5.2.1.8) (Cyclophilin C) (Rotamase C)	Ppic
3Tg and WT		TPM1_MOUSE	Tropomyosin alpha-1 chain (Alpha-tropomyosin) (Tropomyosin-1)	Tpm1
3Tg and WT		TBCA_MOUSE	Tubulin-specific chaperone A (TCP1-chaperonin cofactor A) (Tubulin-folding cofactor A) (CFA)	Tbca
3Tg and WT		RPN2_MOUSE	Dolichyl-diphosphooligosaccharide--protein glycosyltransferase subunit 2 (Dolichyl-diphosphooligosaccharide--protein glycosyltransferase 63 kDa subunit) (Ribophorin II) (RPN-II) (Ribophorin-2)	Rpn2

3Tg and WT		AP2M1_MOUSE	AP-2 complex subunit mu (AP-2 mu chain) (Adaptor protein complex AP-2 subunit mu) (Adaptor-related protein complex 2 subunit mu) (Clathrin assembly protein complex 2 mu medium chain) (Clathrin coat assembly protein AP50) (Clathrin coat-associated protein AP50) (Mu2-adaptin) (Plasma membrane adaptor AP-2 50 kDa protein)	Ap2m1
3Tg and WT		SCYL2_MOUSE	SCY1-like protein 2 (Coated vesicle-associated kinase of 104 kDa)	Scyl2
3Tg and WT		CSRP2_MOUSE	Cysteine and glycine-rich protein 2 (Cysteine-rich protein 2) (CRP2) (Double LIM protein 1) (DLP-1)	Csrp2
3Tg and WT		SYRC_MOUSE	Arginine--tRNA ligase, cytoplasmic (EC 6.1.1.19) (Arginyl-tRNA synthetase) (ArgRS)	Rars
3Tg and WT		DLG1_MOUSE	Disks large homolog 1 (Embryo-dlg/synapse-associated protein 97) (E-dlg/SAP97) (Synapse-associated protein 97) (SAP-97) (SAP97)	Dlg1
3Tg and WT		RL7_MOUSE	60S ribosomal protein L7	Rpl7
3Tg and WT		LRP1_MOUSE	Prolow-density lipoprotein receptor-related protein 1 (LRP-1) (Alpha-2-macroglobulin receptor) (A2MR) (CD antigen CD91) [Cleaved into: Low-density lipoprotein receptor-related protein 1 85 kDa subunit (LRP-85); Low-density lipoprotein receptor-related protein 1 515 kDa subunit (LRP-515); Low-density lipoprotein receptor-related protein 1 intracellular domain (LRPICD)]	Lrp1
3Tg and WT		FAS_MOUSE	Fatty acid synthase (EC 2.3.1.85) [Includes: [Acyl-carrier-protein] S-acetyltransferase (EC 2.3.1.38); [Acyl-carrier-protein] S-malonyltransferase (EC 2.3.1.39); 3-oxoacyl-[acyl-carrier-protein] synthase (EC 2.3.1.41); 3-oxoacyl-[acyl-carrier-protein] reductase (EC 1.1.1.100); 3-hydroxyacyl-[acyl-carrier-protein] dehydratase (EC 4.2.1.59); Enoyl-[acyl-carrier-protein] reductase (EC 1.3.1.39); Oleoyl-[acyl-carrier-protein] hydrolase (EC 3.1.2.14)]	Fasn
3Tg and WT		ITA7_MOUSE	Integrin alpha-7 [Cleaved into: Integrin alpha-7 heavy chain; Integrin alpha-7 light chain]	Itga7
3Tg and WT		NPM_MOUSE	Nucleophosmin (NPM) (Nucleolar phosphoprotein B23) (Nucleolar protein NO38) (Numatrin)	Npm1
3Tg and WT		RCN1_MOUSE	Reticulocalbin-1	Rcn1
3Tg and WT		SEPT7_MOUSE	Septin-7 (CDC10 protein homolog)	Septin7
3Tg and WT		TES_MOUSE	Testin (TES1/TES2)	Tes
3Tg and WT		RTN4_MOUSE	Reticulon-4 (Neurite outgrowth inhibitor) (Nogo protein)	Rtn4
3Tg and WT		H2A1B_MOUSE	Histone H2A type 1-B	H2ac4
3Tg and WT		GT251_MOUSE	Procollagen galactosyltransferase 1 (EC 2.4.1.50) (Collagen beta(1-O)galactosyltransferase 1) (Glycosyltransferase 25 family member 1) (Hydroxylysine galactosyltransferase 1)	Colgalt1
3Tg and WT		AP1B1_MOUSE	AP-1 complex subunit beta-1 (Adaptor protein complex AP-1 subunit beta-1) (Adaptor-related protein complex 1 subunit beta-1) (Beta-1-adaptin) (Beta-adaptin 1) (Clathrin assembly protein complex 1 beta large chain) (Golgi adaptor HA1/AP1 adaptin beta subunit)	Ap1b1
3Tg and WT		RAB31_MOUSE	Ras-related protein Rab-31	Rab31

3Tg and WT		SNAG_MOUSE	Gamma-soluble NSF attachment protein (SNAP-gamma) (N-ethylmaleimide-sensitive factor attachment protein gamma)	Napg
3Tg and WT		RLA1_MOUSE	60S acidic ribosomal protein P1	Rplp1
3Tg and WT		RS29_MOUSE	40S ribosomal protein S29	Rps29
3Tg and WT		MARCS_MOUSE	Myristoylated alanine-rich C-kinase substrate (MARCKS)	Marcks
3Tg and WT		PDIA4_MOUSE	Protein disulfide-isomerase A4 (EC 5.3.4.1) (Endoplasmic reticulum resident protein 72) (ER protein 72) (ERp-72) (ERp72)	Pdia4
3Tg and WT		RRBP1_MOUSE	Ribosome-binding protein 1 (Ribosome receptor protein) (RRp) (mRRp)	Rrbp1
3Tg and WT		PEBP1_MOUSE	Phosphatidylethanolamine-binding protein 1 (PEBP-1) (HCNPpp) [Cleaved into: Hippocampal cholinergic neurostimulating peptide (HCNP)]	Pebp1
3Tg and WT		LC7L2_MOUSE	Putative RNA-binding protein Luc7-like 2 (CGI-74 homolog)	Luc7l2
3Tg and WT		RL9_MOUSE	60S ribosomal protein L9	Rpl9
3Tg and WT		PGAM1_MOUSE	Phosphoglycerate mutase 1 (EC 5.4.2.11) (EC 5.4.2.4) (BPG-dependent PGAM 1) (Phosphoglycerate mutase isozyme B) (PGAM-B)	Pgam1
3Tg and WT		PCNA_MOUSE	Proliferating cell nuclear antigen (PCNA) (Cyclin)	Pcna
3Tg and WT		CLIC4_MOUSE	Chloride intracellular channel protein 4 (mc3s5/mtCLIC)	Clic4
3Tg and WT		ATLA3_MOUSE	Atlastin-3 (EC 3.6.5.-)	Atl3
3Tg and WT		DHE3_MOUSE	Glutamate dehydrogenase 1, mitochondrial (GDH 1) (EC 1.4.1.3)	Glud1
3Tg and WT		K2C79_MOUSE	Keratin, type II cytoskeletal 79 (Cytokeratin-79) (CK-79) (Keratin-79) (K79) (Type-II keratin Kb38)	Krt79
3Tg and WT		LITAF_MOUSE	Lipopolysaccharide-induced tumor necrosis factor-alpha factor homolog (LPS-induced TNF-alpha factor homolog) (Estrogen-enhanced transcript protein) (mEET) (LITAF-like protein) (NEDD4 WW domain-binding protein 3)	Litaf
3Tg and WT		AKA12_MOUSE	A-kinase anchor protein 12 (AKAP-12) (Germ cell lineage protein gercelin) (Src-suppressed C kinase substrate) (SSeCKS)	Akap12
3Tg and WT		RL27A_MOUSE	60S ribosomal protein L27a (L29)	Rpl27a
3Tg and WT		IF2G_MOUSE	Eukaryotic translation initiation factor 2 subunit 3, X-linked (Eukaryotic translation initiation factor 2 subunit gamma, X-linked) (eIF-2-gamma X)	Eif2s3x
3Tg and WT		SEP10_MOUSE	Septin-10	Septin10
3Tg and WT		PA2G4_MOUSE	Proliferation-associated protein 2G4 (IRES-specific cellular trans-acting factor 45 kDa) (ITAF45) (Mpp1) (Proliferation-associated protein 1) (Protein p38-2G4)	Pa2g4
3Tg and WT		PGRC1_MOUSE	Membrane-associated progesterone receptor component 1 (mPR)	Pgrmc1
3Tg and WT		CAV1_MOUSE	Caveolin-1	Cav1
3Tg and WT		SAR1A_MOUSE	GTP-binding protein SAR1a	Sar1a
3Tg and WT		EF1G_MOUSE	Elongation factor 1-gamma (EF-1-gamma) (eEF-1B gamma)	Eef1g
3Tg and WT		HINT1_MOUSE	Histidine triad nucleotide-binding protein 1 (EC 3.-.-.-) (Adenosine 5'-monophosphoramidase) (Protein kinase C inhibitor 1) (Protein kinase C-interacting protein 1) (PKCI-1)	Hint1
3Tg and WT		ACK1_MOUSE	Activated CDC42 kinase 1 (ACK-1) (EC 2.7.10.2) (EC 2.7.11.1) (Non-receptor protein tyrosine kinase Ack) (Tyrosine kinase non-receptor protein 2)	Tnk2

3Tg and WT		AAPK1_MOUSE	5'-AMP-activated protein kinase catalytic subunit alpha-1 (AMPK subunit alpha-1) (EC 2.7.11.1) (Acetyl-CoA carboxylase kinase) (ACACA kinase) (EC 2.7.11.27) (Hydroxymethylglutaryl-CoA reductase kinase) (HMGCR kinase) (EC 2.7.11.31) (Tau-protein kinase PRKAA1) (EC 2.7.11.26)	Prkaa1
3Tg and WT		DYN2_MOUSE	Dynamin-2 (EC 3.6.5.5) (Dynamin UDNM)	Dnm2
3Tg and WT		NEST_MOUSE	Nestin	Nes
3Tg and WT		DAG1_MOUSE	Dystroglycan (Dystrophin-associated glycoprotein 1) [Cleaved into: Alpha-dystroglycan (Alpha-DG); Beta-dystroglycan (Beta-DG)]	Dag1
3Tg and WT		IBP2_MOUSE	Insulin-like growth factor-binding protein 2 (IBP-2) (IGF-binding protein 2) (IGFBP-2) (mIGFBP-2)	Igfbp2
3Tg and WT		RL23_MOUSE	60S ribosomal protein L23	Rpl23
3Tg and WT		EZRI_MOUSE	Ezrin (Cytovillin) (Villin-2) (p81)	Ezr
3Tg and WT		FMNL2_MOUSE	Formin-like protein 2 (Protein Man)	Fmnl2
3Tg and WT		RLA0_MOUSE	60S acidic ribosomal protein P0 (60S ribosomal protein L10E)	Rplp0
3Tg and WT		PCBP3_MOUSE	Poly(rC)-binding protein 3 (Alpha-CP3)	Pcbp3
3Tg and WT		SERA_MOUSE	D-3-phosphoglycerate dehydrogenase (3-PGDH) (EC 1.1.1.95) (A10)	Phgdh
3Tg and WT		NLTP_MOUSE	Non-specific lipid-transfer protein (NSL-TP) (EC 2.3.1.176) (Propanoyl-CoA C-acyltransferase) (SCP-chi) (SCPX) (Sterol carrier protein 2) (SCP-2) (Sterol carrier protein X) (SCP-X)	Scp2
3Tg and WT		OSTF1_MOUSE	Osteoclast-stimulating factor 1 (SH3 domain protein 3)	Ostf1
3Tg and WT		EIF3D_MOUSE	Eukaryotic translation initiation factor 3 subunit D (eIF3d) (Eukaryotic translation initiation factor 3 subunit 7) (eIF-3-zeta) (eIF3 p66)	Eif3d
3Tg and WT		RSSA_MOUSE	40S ribosomal protein SA (37 kDa laminin receptor precursor) (37LRP) (37 kDa oncofetal antigen) (37/67 kDa laminin receptor) (LRP/LR) (67 kDa laminin receptor) (67LR) (Laminin receptor 1) (LamR) (Laminin-binding protein precursor p40) (LBP/p40) (OFA/iLRP)	Rpsa
3Tg and WT		PPIB_MOUSE	Peptidyl-prolyl cis-trans isomerase B (PPIase B) (EC 5.2.1.8) (CYP-S1) (Cyclophilin B) (Rotamase B) (S-cyclophilin) (SCYLP)	Ppib
3Tg and WT		CAP1_MOUSE	Adenylyl cyclase-associated protein 1 (CAP 1)	Cap1
3Tg and WT		K2C6A_MOUSE	Keratin, type II cytoskeletal 6A (Cytokeratin-6A) (CK-6A) (Keratin-6-alpha) (mK6-alpha) (Keratin-6A) (K6A)	Krt6a
3Tg and WT		IPO5_MOUSE	Importin-5 (Imp5) (Importin subunit beta-3) (Karyopherin beta-3) (Ran-binding protein 5) (RanBP5)	Ipo5
3Tg and WT		ASSY_MOUSE	Argininosuccinate synthase (EC 6.3.4.5) (Citrulline--aspartate ligase)	Ass1
3Tg and WT		S10A6_MOUSE	Protein S100-A6 (5B10) (Calcyclin) (Prolactin receptor-associated protein) (S100 calcium-binding protein A6)	S100a6
3Tg and WT		OST48_MOUSE	Dolichyl-diphosphooligosaccharide--protein glycosyltransferase 48 kDa subunit (DDOST 48 kDa subunit) (Oligosaccharyl transferase 48 kDa subunit)	Ddost
3Tg and WT		MYO1E_MOUSE	Unconventional myosin-Ie (Unconventional myosin 1E)	Myo1e
3Tg and WT		BORG5_MOUSE	Cdc42 effector protein 1 (Binder of Rho GTPases 5)	Cdc42ep1

3Tg and WT		PDLI7_MOUSE	PDZ and LIM domain protein 7 (LIM mineralization protein) (LMP) (Protein enigma)	Pdlim7
3Tg and WT		RS21_MOUSE	40S ribosomal protein S21	Rps21
3Tg and WT		GELS_MOUSE	Gelsolin (Actin-depolymerizing factor) (ADF) (Brevin)	Gsn
3Tg and WT		MYEF2_MOUSE	Myelin expression factor 2 (MEF-2) (MyEF-2)	Myef2
3Tg and WT		OSTC_MOUSE	Oligosaccharyltransferase complex subunit OSTC	Ostc
3Tg and WT		RAN_MOUSE	GTP-binding nuclear protein Ran (GTPase Ran) (Ras-like protein TC4) (Ras-related nuclear protein)	Ran
3Tg and WT		PDLI5_MOUSE	PDZ and LIM domain protein 5 (Enigma homolog) (Enigma-like PDZ and LIM domains protein)	Pdlim5
3Tg and WT		VAPA_MOUSE	Vesicle-associated membrane protein-associated protein A (VAMP-A) (VAMP-associated protein A) (VAP-A) (33 kDa VAMP-associated protein) (VAP-33)	Vapa
3Tg and WT		CD81_MOUSE	CD81 antigen (26 kDa cell surface protein TAPA-1) (Target of the antiproliferative antibody 1) (CD antigen CD81)	Cd81
3Tg and WT		ABI1_MOUSE	Abl interactor 1 (Abelson interactor 1) (Abi-1) (Ablphilin-1) (Eps8 SH3 domain-binding protein) (Eps8-binding protein) (Spectrin SH3 domain-binding protein 1) (e3B1)	Abi1
3Tg and WT		COR1C_MOUSE	Coronin-1C (Coronin-3)	Coro1c
3Tg and WT		MPRIP_MOUSE	Myosin phosphatase Rho-interacting protein (Rho-interacting protein 3) (RIP3) (p116Rip)	Mprip
3Tg and WT		SRC_MOUSE	Neuronal proto-oncogene tyrosine-protein kinase Src (EC 2.7.10.2) (Proto-oncogene c-Src) (pp60c-src) (p60-Src)	Src
3Tg and WT		ACTY_MOUSE	Beta-centractin (Actin-related protein 1B) (ARP1B)	Actr1b
3Tg and WT		G3P_MOUSE	Glyceraldehyde-3-phosphate dehydrogenase (GAPDH) (EC 1.2.1.12) (Peptidyl-cysteine S-nitrosylase GAPDH) (EC 2.6.99.-)	Gapdh
3Tg and WT		RASN_MOUSE	GTPase NRas (Transforming protein N-Ras)	Nras
3Tg and WT		TBB5_MOUSE	Tubulin beta-5 chain	Tubb5
3Tg and WT		RL13_MOUSE	60S ribosomal protein L13 (A52)	Rpl13
3Tg and WT		HMOX2_MOUSE	Heme oxygenase 2 (HO-2) (EC 1.14.14.18)	Hmox2
3Tg and WT		FLOT1_MOUSE	Flotillin-1	Flot1
3Tg and WT		1433B_MOUSE	14-3-3 protein beta/alpha (Protein kinase C inhibitor protein 1) (KCIP-1) [Cleaved into: 14-3-3 protein beta/alpha, N-terminally processed]	Ywhab
3Tg and WT		ASAH1_MOUSE	Acid ceramidase (AC) (ACDase) (Acid CDase) (EC 3.5.1.23) (Acylsphingosine deacylase) (N-acylethanolamine hydrolase ASAH1) (EC 3.5.1.-) (N-acylsphingosine amidohydrolase) [Cleaved into: Acid ceramidase subunit alpha; Acid ceramidase subunit beta]	Asah1
3Tg and WT		LTOR1_MOUSE	Ragulator complex protein LAMTOR1 (Late endosomal/lysosomal adaptor and MAPK and MTOR activator 1) (Lipid raft adaptor protein p18)	Lamtor1
3Tg and WT		COX2_MOUSE	Cytochrome c oxidase subunit 2 (Cytochrome c oxidase polypeptide II)	Mtco2
3Tg and WT		CPNS1_MOUSE	Calpain small subunit 1 (CSS1) (Calcium-activated neutral proteinase small subunit) (CANP small subunit) (Calcium-dependent protease small subunit) (CDPS) (Calcium-dependent protease small subunit 1) (Calpain regulatory subunit)	Capns1

3Tg and WT		ROCK2_MOUSE	Rho-associated protein kinase 2 (EC 2.7.11.1) (Rho-associated, coiled-coil-containing protein kinase 2) (Rho-associated, coiled-coil-containing protein kinase II) (ROCK-II) (p164 ROCK-2)	Rock2
3Tg and WT		F10A1_MOUSE	Hsc70-interacting protein (Hip) (Protein FAM10A1) (Protein ST13 homolog)	St13
3Tg and WT		VP26A_MOUSE	Vacuolar protein sorting-associated protein 26A (H<beta>58 protein) (H beta 58) (Vesicle protein sorting 26A) (mVPS26)	Vps26a
3Tg and WT		NDKB_MOUSE	Nucleoside diphosphate kinase B (NDK B) (NDP kinase B) (EC 2.7.4.6) (Histidine protein kinase NDKB) (EC 2.7.13.3) (P18) (nm23-M2)	Nme2
3Tg and WT		SDC4_MOUSE	Syndecan-4 (SYND4) (Ryudocan core protein)	Sdc4
3Tg and WT		RS7_MOUSE	40S ribosomal protein S7	Rps7
3Tg and WT		BZW1_MOUSE	Basic leucine zipper and W2 domain-containing protein 1	Bzw1
3Tg and WT		RL34_MOUSE	60S ribosomal protein L34	Rpl34
3Tg and WT		SEP11_MOUSE	Septin-11	Septin11
3Tg and WT		RAB18_MOUSE	Ras-related protein Rab-18	Rab18
3Tg and WT		MANF_MOUSE	Mesencephalic astrocyte-derived neurotrophic factor (Arginine-rich protein) (Protein ARMET)	Manf
3Tg and WT		COPA_MOUSE	Coatomer subunit alpha (Alpha-coat protein) (Alpha-COP) [Cleaved into: Xenin (Xenopsin-related peptide); Proxenin]	Copa
3Tg and WT		GBB2_MOUSE	Guanine nucleotide-binding protein G(I)/G(S)/G(T) subunit beta-2 (G protein subunit beta-2) (Transducin beta chain 2)	Gnb2
3Tg and WT		LYN_MOUSE	Tyrosine-protein kinase Lyn (EC 2.7.10.2) (V-yes-1 Yamaguchi sarcoma viral related oncogene homolog) (p53Lyn) (p56Lyn)	Lyn
3Tg and WT		ARF4_MOUSE	ADP-ribosylation factor 4	Arf4
3Tg and WT		AP3D1_MOUSE	AP-3 complex subunit delta-1 (AP-3 complex subunit delta) (Adaptor-related protein complex 3 subunit delta-1) (Delta-adaptin) (mBLVR1)	Ap3d1
3Tg and WT		MDHM_MOUSE	Malate dehydrogenase, mitochondrial (EC 1.1.1.37)	Mdh2
3Tg and WT		ACTN1_MOUSE	Alpha-actinin-1 (Alpha-actinin cytoskeletal isoform) (F-actin cross-linking protein) (Non-muscle alpha-actinin-1)	Actn1
3Tg and WT		LXN_MOUSE	Latexin (Endogenous carboxypeptidase inhibitor) (ECI) (Tissue carboxypeptidase inhibitor) (TCI)	Lxn
3Tg and WT		CAND1_MOUSE	Cullin-associated NEDD8-dissociated protein 1 (Cullin-associated and neddylation-dissociated protein 1) (p120 CAND1)	Cand1
3Tg and WT		PEF1_MOUSE	Peflin (PEF protein with a long N-terminal hydrophobic domain) (Penta-EF hand domain-containing protein 1)	Pef1
3Tg and WT		AT1B3_MOUSE	Sodium/potassium-transporting ATPase subunit beta-3 (Sodium/potassium-dependent ATPase subunit beta-3) (ATPB-3) (CD antigen CD298)	Atp1b3
3Tg and WT		RS27_MOUSE	40S ribosomal protein S27	Rps27
3Tg and WT		ITA1_MOUSE	Integrin alpha-1 (CD49 antigen-like family member A) (Laminin and collagen receptor) (VLA-1) (CD antigen CD49a)	Itga1
3Tg and WT		SRC8_MOUSE	Src substrate cortactin	Cttn
3Tg and WT		RAC1_MOUSE	Ras-related C3 botulinum toxin substrate 1 (EC 3.6.5.2) (p21-Rac1)	Rac1
3Tg and WT		GNA11_MOUSE	Guanine nucleotide-binding protein subunit alpha-11 (G alpha-11) (G-protein subunit alpha-11)	Gna11

3Tg and WT		BLK_MOUSE	Tyrosine-protein kinase Blk (EC 2.7.10.2) (B lymphocyte kinase) (p55-Blk)	Blk
3Tg and WT		TXND5_MOUSE	Thioredoxin domain-containing protein 5 (Endoplasmic reticulum resident protein 46) (ER protein 46) (ERp46) (Plasma cell-specific thioredoxin-related protein) (PC-TRP) (Thioredoxin-like protein p46)	Txndc5
3Tg and WT		TPM3_MOUSE	Tropomyosin alpha-3 chain (Gamma-tropomyosin) (Tropomyosin-3)	Tpm3
3Tg and WT		PKHO2_MOUSE	Pleckstrin homology domain-containing family O member 2 (PH domain-containing family O member 2) (Pleckstrin homology domain-containing family Q member 1) (PH domain-containing family Q member 1)	Plekho2
3Tg and WT		STMN1_MOUSE	Stathmin (Leukemia-associated gene protein) (Leukemia-associated phosphoprotein p18) (Metablastin) (Oncoprotein 18) (Op18) (Phosphoprotein p19) (pp19) (Prosolin) (Protein Pr22) (pp17)	Stmn1
3Tg and WT		TKT_MOUSE	Transketolase (TK) (EC 2.2.1.1) (P68)	Tkt
3Tg and WT		ARPC4_MOUSE	Actin-related protein 2/3 complex subunit 4 (Arp2/3 complex 20 kDa subunit) (p20-ARC)	Arpc4
3Tg and WT		RL27_MOUSE	60S ribosomal protein L27	Rpl27
3Tg and WT		LAT1_MOUSE	Large neutral amino acids transporter small subunit 1 (4F2 light chain) (4F2 LC) (4F2LC) (L-type amino acid transporter 1) (LAT1) (Solute carrier family 7 member 5)	Slc7a5
3Tg and WT		ARPC5_MOUSE	Actin-related protein 2/3 complex subunit 5 (Arp2/3 complex 16 kDa subunit) (p16-ARC)	Arpc5
3Tg and WT		LMAN1_MOUSE	Protein ERGIC-53 (ER-Golgi intermediate compartment 53 kDa protein) (Lectin mannose-binding 1) (p58)	Lman1
3Tg and WT		HMOX1_MOUSE	Heme oxygenase 1 (HO-1) (EC 1.14.14.18) (P32 protein)	Hmox1
3Tg and WT		PHLB1_MOUSE	Pleckstrin homology-like domain family B member 1 (Protein LL5-alpha)	Phldb1
3Tg and WT		SH3G1_MOUSE	Endophilin-A2 (Endophilin-2) (SH3 domain protein 2B) (SH3 domain-containing GRB2-like protein 1) (SH3p8)	Sh3gl1
3Tg and WT		TXNL1_MOUSE	Thioredoxin-like protein 1 (32 kDa thioredoxin-related protein)	Txnl1
3Tg and WT		LPP_MOUSE	Lipoma-preferred partner homolog	Lpp
3Tg and WT		HNRPD_MOUSE	Heterogeneous nuclear ribonucleoprotein D0 (hnRNP D0) (AU-rich element RNA-binding protein 1)	Hnrnpd
3Tg and WT		H2B1F_MOUSE	Histone H2B type 1-F/J/L (H2B 291A)	H2bc7
3Tg and WT		ERF3B_MOUSE	Eukaryotic peptide chain release factor GTP-binding subunit ERF3B (Eukaryotic peptide chain release factor subunit 3b) (eRF3b) (G1 to S phase transition protein 2 homolog)	Gspt2
3Tg and WT		DDX5_MOUSE	Probable ATP-dependent RNA helicase DDX5 (EC 3.6.4.13) (DEAD box RNA helicase DEAD1) (mDEAD1) (DEAD box protein 5) (RNA helicase p68)	Ddx5
3Tg and WT		RS28_MOUSE	40S ribosomal protein S28	Rps28
3Tg and WT		RL23A_MOUSE	60S ribosomal protein L23a	Rpl23a
3Tg and WT		SNAA_MOUSE	Alpha-soluble NSF attachment protein (SNAP-alpha) (N-ethylmaleimide-sensitive factor attachment protein alpha)	Napa

3Tg and WT		UB2V1_MOUSE	Ubiquitin-conjugating enzyme E2 variant 1 (UEV-1) (CROC-1)	Ube2v1
3Tg and WT		PRS7_MOUSE	26S proteasome regulatory subunit 7 (26S proteasome AAA-ATPase subunit RPT1) (Proteasome 26S subunit ATPase 2) (Protein MSS1)	Psmc2
3Tg and WT		TERA_MOUSE	Transitional endoplasmic reticulum ATPase (TER ATPase) (EC 3.6.4.6) (15S Mg(2+)-ATPase p97 subunit) (Valosin-containing protein) (VCP)	Vcp
3Tg and WT		H32_MOUSE	Histone H3.2	Hist1h3b
3Tg and WT		SYK_MOUSE	Lysine--tRNA ligase (EC 2.7.7.-) (EC 6.1.1.6) (Lysyl-tRNA synthetase) (LysRS)	Kars1
3Tg and WT		CISY_MOUSE	Citrate synthase, mitochondrial (EC 2.3.3.1) (Citrate (Si)-synthase)	Cs
3Tg and WT		SERC_MOUSE	Phosphoserine aminotransferase (PSAT) (EC 2.6.1.52) (Endometrial progesterone-induced protein) (EPIP) (Phosphohydroxythreonine aminotransferase)	Psat1
3Tg and WT		RAB21_MOUSE	Ras-related protein Rab-21 (Rab-12)	Rab21
3Tg and WT		CSRP1_MOUSE	Cysteine and glycine-rich protein 1 (Cysteine-rich protein 1) (CRP) (CRP1)	Csrp1
3Tg and WT		NP1L1_MOUSE	Nucleosome assembly protein 1-like 1 (Brain protein DN38) (NAP-1-related protein)	Nap1l1
3Tg and WT		KCRB_MOUSE	Creatine kinase B-type (EC 2.7.3.2) (B-CK) (Creatine kinase B chain) (Creatine phosphokinase B-type) (CPK-B)	Ckb
3Tg and WT		TGFI1_MOUSE	Transforming growth factor beta-1-induced transcript 1 protein (Androgen receptor-associated protein of 55 kDa) (Hydrogen peroxide-inducible clone 5 protein) (Hic-5) (TGF beta-stimulated clone 5) (TSC-5)	Tgfb1i1
3Tg and WT		COPG1_MOUSE	Coatomer subunit gamma-1 (Gamma-1-coat protein) (Gamma-1-COP)	Copg1
3Tg and WT		DYL2_MOUSE	Dynein light chain 2, cytoplasmic (8 kDa dynein light chain b) (DLC8) (DLC8b) (Dynein light chain LC8-type 2)	Dynll2
3Tg and WT		AK1A1_MOUSE	Aldo-keto reductase family 1 member A1 (EC 1.1.1.2) (EC 1.1.1.33) (EC 1.1.1.372) (EC 1.1.1.54) (Alcohol dehydrogenase [NADP(+)]) (Aldehyde reductase) (Glucuronate reductase) (EC 1.1.1.19) (Glucuronolactone reductase) (EC 1.1.1.20)	Akr1a1
3Tg and WT		RL35A_MOUSE	60S ribosomal protein L35a	Rpl35a
3Tg and WT		ACON_MOUSE	Aconitate hydratase, mitochondrial (Aconitase) (EC 4.2.1.3) (Citrate hydro-lyase)	Aco2
3Tg and WT		CAD11_MOUSE	Cadherin-11 (OSF-4) (Osteoblast cadherin) (OB-cadherin)	Cdh11
3Tg and WT		SCAM3_MOUSE	Secretory carrier-associated membrane protein 3 (Secretory carrier membrane protein 3)	Scamp3
3Tg and WT		SND1_MOUSE	Staphylococcal nuclease domain-containing protein 1 (EC 3.1.31.1) (100 kDa coactivator) (p100 co-activator)	Snd1
3Tg and WT		RAGP1_MOUSE	Ran GTPase-activating protein 1 (RanGAP1)	Rangap1
3Tg and WT		PAIRB_MOUSE	Plasminogen activator inhibitor 1 RNA-binding protein (PAI1 RNA-binding protein 1) (PAI-RBP1) (SERPINE1 mRNA-binding protein 1)	Serbp1
3Tg and WT		MAP4_MOUSE	Microtubule-associated protein 4 (MAP-4)	Map4
3Tg and WT		COBL1_MOUSE	Cordon-bleu protein-like 1 (Cobl-related protein 1)	Cobll1

3Tg and WT		PUR9_MOUSE	Bifunctional purine biosynthesis protein PURH [Includes: Phosphoribosylaminoimidazolecarboxamide formyltransferase (EC 2.1.2.3) (5-aminoimidazole-4-carboxamide ribonucleotide formyltransferase) (AICAR transformylase); IMP cyclohydrolase (EC 3.5.4.10) (ATIC) (IMP synthase) (Inosinicase)]	Atic
3Tg and WT		FARP1_MOUSE	FERM, ARHGEF and pleckstrin domain-containing protein 1 (FERM, RhoGEF and pleckstrin domain-containing protein 1)	Farp1
3Tg and WT		TBB6_MOUSE	Tubulin beta-6 chain	Tubb6
3Tg and WT		SNP23_MOUSE	Synaptosomal-associated protein 23 (SNAP-23) (Syndet) (Vesicle-membrane fusion protein SNAP-23)	Snap23
3Tg and WT		PZP_MOUSE	Pregnancy zone protein (Alpha-2-macroglobulin) (Alpha-2-M) [Cleaved into: Alpha-2-macroglobulin 165 kDa subunit; Alpha-2-macroglobulin 35 kDa subunit]	Pzp
3Tg and WT		PDLI1_MOUSE	PDZ and LIM domain protein 1 (C-terminal LIM domain protein 1) (Elfin) (LIM domain protein CLP-36)	Pdlim1
3Tg and WT		ELAV1_MOUSE	ELAV-like protein 1 (Elav-like generic protein) (Hu-antigen R) (HuR) (MelG)	Elavl1
3Tg and WT		TLN1_MOUSE	Talin-1	Tln1
3Tg and WT		RHOC_MOUSE	Rho-related GTP-binding protein RhoC (Silica-induced gene 61 protein) (SIG-61)	Rhoc
3Tg and WT		RL5_MOUSE	60S ribosomal protein L5	Rpl5
3Tg and WT		RS25_MOUSE	40S ribosomal protein S25	Rps25
3Tg and WT		ACTZ_MOUSE	Alpha-centractin (Centractin) (ARP1) (Actin-RPV) (Centrosome-associated actin homolog)	Actr1a
3Tg and WT		RL26_MOUSE	60S ribosomal protein L26 (Silica-induced gene 20 protein) (SIG-20)	Rpl26
3Tg and WT		RASH_MOUSE	GTPase HRas (H-Ras-1) (Transforming protein p21) (c-H-ras) (p21ras) [Cleaved into: GTPase HRas, N-terminally processed]	Hras
3Tg and WT		ACLY_MOUSE	ATP-citrate synthase (EC 2.3.3.8) (ATP-citrate (pro-S-)-lyase) (Citrate cleavage enzyme)	Acly
3Tg and WT		NUCB2_MOUSE	Nucleobindin-2 (DNA-binding protein NEFA) (Prepronesfatin) [Cleaved into: Nesfatin-1]	Nucb2
3Tg and WT		HNRPK_MOUSE	Heterogeneous nuclear ribonucleoprotein K (hnRNP K)	Hnrnpk
3Tg and WT		HSPB1_MOUSE	Heat shock protein beta-1 (HspB1) (Growth-related 25 kDa protein) (Heat shock 25 kDa protein) (HSP 25) (Heat shock 27 kDa protein) (HSP 27) (p25)	Hspb1
3Tg and WT		ICAL_MOUSE	Calpastatin (Calpain inhibitor)	Cast
3Tg and WT		XIRP2_MOUSE	Xin actin-binding repeat-containing protein 2 (Beta-xin) (Cardiomyopathy-associated protein 3) (Myogenic MEF2-activated Xin-related protein) (Myomaxin) (mXinbeta)	Xirp2
3Tg and WT		LAP2B_MOUSE	Lamina-associated polypeptide 2, isoforms beta/delta/epsilon/gamma (Thymopoietin isoforms beta/delta/epsilon/gamma) (TP beta/delta/epsilon/gamma)	Tmpo
3Tg and WT		SAHH3_MOUSE	Putative adenosylhomocysteinase 3 (AdoHcyase 3) (EC 3.3.1.1) (Long-IRBIT) (S-adenosyl-L-homocysteine hydrolase 3) (S-adenosylhomocysteine hydrolase-like protein 2)	Ahcyl2

3Tg and WT		SYNC_MOUSE	Asparagine--tRNA ligase, cytoplasmic (EC 6.1.1.22) (Asparaginyl-tRNA synthetase) (AsnRS) (Asparaginyl-tRNA synthetase 1)	NARS1
3Tg and WT		RB22A_MOUSE	Ras-related protein Rab-22A (Rab-22) (Rab-14)	Rab22a
3Tg and WT		ANXA6_MOUSE	Annexin A6 (67 kDa calelectrin) (Annexin VI) (Annexin-6) (Calphobindin-II) (CPB-II) (Chromobindin-20) (Lipocortin VI) (Protein III) (p68) (p70)	Anxa6
3Tg and WT		HNRPF_MOUSE	Heterogeneous nuclear ribonucleoprotein F (hnRNP F) [Cleaved into: Heterogeneous nuclear ribonucleoprotein F, N-terminally processed]	Hnrnpf
3Tg and WT		STXB3_MOUSE	Syntaxin-binding protein 3 (MUNC-18-3) (Mammalian homolog of Unc-18c) (Munc-18c) (Protein unc-18 homolog 3) (Unc18-3) (Protein unc-18 homolog C) (Unc-18C)	Stxbp3
3Tg and WT		PSB6_MOUSE	Proteasome subunit beta type-6 (EC 3.4.25.1) (Low molecular mass protein 19) (Macropain delta chain) (Multicatalytic endopeptidase complex delta chain) (Proteasome delta chain) (Proteasome subunit Y)	Psmb6
3Tg and WT		HGS_MOUSE	Hepatocyte growth factor-regulated tyrosine kinase substrate	Hgs
3Tg and WT		CPNE1_MOUSE	Copine-1 (Copine I)	Cpne1
3Tg and WT		1433T_MOUSE	14-3-3 protein theta (14-3-3 protein tau)	Ywhaq
3Tg and WT		HS90A_MOUSE	Heat shock protein HSP 90-alpha (Heat shock 86 kDa) (HSP 86) (HSP86) (Tumor-specific transplantation 86 kDa antigen) (TSTA)	Hsp90aa1
3Tg and WT		CRYAB_MOUSE	Alpha-crystallin B chain (Alpha(B)-crystallin) (P23)	Cryab
3Tg and WT		P5CS_MOUSE	Delta-1-pyrroline-5-carboxylate synthase (P5CS) (Aldehyde dehydrogenase family 18 member A1) [Includes: Glutamate 5-kinase (GK) (EC 2.7.2.11) (Gamma-glutamyl kinase); Gamma-glutamyl phosphate reductase (GPR) (EC 1.2.1.41) (Glutamate-5-semialdehyde dehydrogenase) (Glutamyl-gamma-semialdehyde dehydrogenase)]	Aldh18a1
3Tg and WT		HS90B_MOUSE	Heat shock protein HSP 90-beta (Heat shock 84 kDa) (HSP 84) (HSP84) (Tumor-specific transplantation 84 kDa antigen) (TSTA)	Hsp90ab1
3Tg and WT		FIGL1_MOUSE	Fidgetin-like protein 1 (EC 3.6.4.-)	Fignl1
3Tg and WT		NACAM_MOUSE	Nascent polypeptide-associated complex subunit alpha, muscle-specific form (Alpha-NAC, muscle-specific form) (skNAC)	Naca
3Tg and WT		HS71L_MOUSE	Heat shock 70 kDa protein 1-like (Heat shock 70 kDa protein 1L) (Heat shock 70 kDa-like protein 1) (Spermatid-specific heat shock protein 70)	Hspa1l
3Tg and WT		CH60_MOUSE	60 kDa heat shock protein, mitochondrial (EC 5.6.1.7) (60 kDa chaperonin) (Chaperonin 60) (CPN60) (HSP-65) (Heat shock protein 60) (HSP-60) (Hsp60) (Mitochondrial matrix protein P1)	Hspd1
3Tg and WT		DNJB1_MOUSE	DnaJ homolog subfamily B member 1 (Heat shock 40 kDa protein 1) (HSP40) (Heat shock protein 40)	Dnajb1
3Tg and WT		RL28_MOUSE	60S ribosomal protein L28	Rpl28
3Tg and WT		RS14_MOUSE	40S ribosomal protein S14	Rps14
3Tg and WT		CAPZB_MOUSE	F-actin-capping protein subunit beta (CapZ beta)	Capzb
3Tg and WT		FLNB_MOUSE	Filamin-B (FLN-B) (ABP-280-like protein) (Actin-binding-like protein) (Beta-filamin)	Flnb

3Tg and WT		4F2_MOUSE	4F2 cell-surface antigen heavy chain (4F2hc) (Solute carrier family 3 member 2) (CD antigen CD98)	Slc3a2
3Tg and WT		TF_MOUSE	Tissue factor (TF) (Coagulation factor III) (CD antigen CD142)	F3
3Tg and WT		CD34_MOUSE	Hematopoietic progenitor cell antigen CD34 (CD antigen CD34)	Cd34
3Tg and WT		SNX3_MOUSE	Sorting nexin-3 (SDP3 protein)	Snx3
3Tg and WT		ATPB_MOUSE	ATP synthase subunit beta, mitochondrial (EC 7.1.2.2) (ATP synthase F1 subunit beta)	Atp5f1b
3Tg and WT		SPB6_MOUSE	Serpin B6 (Placental thrombin inhibitor) (Proteinase inhibitor 6) (PI-6)	Serpinb6
3Tg and WT		DLRB1_MOUSE	Dynein light chain roadblock-type 1 (Dynein light chain 2A, cytoplasmic)	Dynlrb1
3Tg and WT		ANXA1_MOUSE	Annexin A1 (Annexin I) (Annexin-1) (Calpactin II) (Calpactin-2) (Chromobindin-9) (Lipocortin I) (Phospholipase A2 inhibitory protein) (p35)	Anxa1
3Tg and WT		SCRIB_MOUSE	Protein scribble homolog (Scribble) (Protein LAP4)	Scrib
3Tg and WT		IF4G1_MOUSE	Eukaryotic translation initiation factor 4 gamma 1 (eIF-4-gamma 1) (eIF-4G 1) (eIF-4G1)	Eif4g1
3Tg and WT		E41L3_MOUSE	Band 4.1-like protein 3 (4.1B) (Differentially expressed in adenocarcinoma of the lung protein 1) (DAL-1) (DAL1P) (mDAL-1) [Cleaved into: Band 4.1-like protein 3, N-terminally processed]	Epb41l3
3Tg and WT		NDUA4_MOUSE	Cytochrome c oxidase subunit NDUFA4	Ndufa4
3Tg and WT		RADI_MOUSE	Radixin (ESP10)	Rdx
3Tg and WT		CP51A_MOUSE	Lanosterol 14-alpha demethylase (LDM) (EC 1.14.14.154) (CYPLI) (Cytochrome P450 51A1) (Cytochrome P450-14DM) (Cytochrome P45014DM) (Cytochrome P450LI) (Sterol 14-alpha demethylase)	Cyp51a1
3Tg and WT		CPNE3_MOUSE	Copine-3 (Copine III)	Cpne3
3Tg and WT		CSPG4_MOUSE	Chondroitin sulfate proteoglycan 4 (Chondroitin sulfate proteoglycan NG2) (Proteoglycan AN2)	Cspg4
3Tg and WT		AP2B1_MOUSE	AP-2 complex subunit beta (AP105B) (Adaptor protein complex AP-2 subunit beta) (Adaptor-related protein complex 2 subunit beta) (Beta-2-adaptin) (Beta-adaptin) (Clathrin assembly protein complex 2 beta large chain) (Plasma membrane adaptor HA2/AP2 adaptin beta subunit)	Ap2b1
3Tg and WT		SC6A6_MOUSE	Sodium- and chloride-dependent taurine transporter (Solute carrier family 6 member 6)	Slc6a6
3Tg and WT		RELL1_MOUSE	RELT-like protein 1	Rell1
3Tg and WT		RL37_MOUSE	60S ribosomal protein L37	Rpl37
3Tg and WT		PRAX_MOUSE	Periaxin	Prx
3Tg and WT		MOES_MOUSE	Moesin (Membrane-organizing extension spike protein)	Msn
3Tg and WT		DHB12_MOUSE	Very-long-chain 3-oxoacyl-CoA reductase (EC 1.1.1.330) (17-beta-hydroxysteroid dehydrogenase 12) (17-beta-HSD 12) (3-ketoacyl-CoA reductase) (KAR) (Estradiol 17-beta-dehydrogenase 12) (EC 1.1.1.62) (KIK-I)	Hsd17b12
3Tg and WT		EPDR1_MOUSE	Mammalian ependymin-related protein 1 (MERP-1)	Epdr1
3Tg and WT		ATP5H_MOUSE	ATP synthase subunit d, mitochondrial (ATPase subunit d) (ATP synthase peripheral stalk subunit d)	Atp5pd
3Tg and WT		RAB10_MOUSE	Ras-related protein Rab-10	Rab10
3Tg and WT		ARP3_MOUSE	Actin-related protein 3 (Actin-like protein 3)	Actr3

3Tg and WT		RAB1A_MOUSE	Ras-related protein Rab-1A (YPT1-related protein)	Rab1A
3Tg and WT		ILK_MOUSE	Integrin-linked protein kinase (EC 2.7.11.1)	Ilk
3Tg and WT		MYH10_MOUSE	Myosin-10 (Cellular myosin heavy chain, type B) (Myosin heavy chain 10) (Myosin heavy chain, non-muscle IIb) (Non-muscle myosin heavy chain B) (NMMHC-B) (Non-muscle myosin heavy chain IIb) (NMMHC II-b) (NMMHC-IIB)	Myh10
3Tg and WT		CYFP1_MOUSE	Cytoplasmic FMR1-interacting protein 1 (Specifically Rac1-associated protein 1) (Sra-1)	Cyfip1
3Tg and WT		SSRA_MOUSE	Translocon-associated protein subunit alpha (TRAP-alpha) (Signal sequence receptor subunit alpha) (SSR-alpha)	Ssr1
3Tg and WT		H15_MOUSE	Histone H1.5 (H1 VAR.5) (H1b)	H1-5
3Tg and WT		LIN7C_MOUSE	Protein lin-7 homolog C (Lin-7C) (mLin7C) (Mammalian lin-seven protein 3) (MALS-3) (Vertebrate lin-7 homolog 3) (Veli-3)	Lin7c
3Tg and WT		LIPB1_MOUSE	Liprin-beta-1 (Protein tyrosine phosphatase receptor type f polypeptide-interacting protein-binding protein 1) (PTPRF-interacting protein-binding protein 1)	Ppfibp1
3Tg and WT		HNRPC_MOUSE	Heterogeneous nuclear ribonucleoproteins C1/C2 (hnRNP C1/C2)	Hnrnpc
3Tg and WT		TPIS_MOUSE	Triosephosphate isomerase (TIM) (EC 5.3.1.1) (Methylglyoxal synthase) (EC 4.2.3.3) (Triose-phosphate isomerase)	Tpi1
3Tg and WT		1433Z_MOUSE	14-3-3 protein zeta/delta (Protein kinase C inhibitor protein 1) (KCIP-1) (SEZ-2)	Ywhaz
3Tg and WT		SYLC_MOUSE	Leucine--tRNA ligase, cytoplasmic (EC 6.1.1.4) (Leucyl-tRNA synthetase) (LeuRS)	Lars
3Tg and WT		MPCP_MOUSE	Phosphate carrier protein, mitochondrial (Phosphate transport protein) (PTP) (Solute carrier family 25 member 3)	Slc25a3
3Tg and WT		WASF2_MOUSE	Wiskott-Aldrich syndrome protein family member 2 (WASP family protein member 2) (Protein WAVE-2)	Wasf2
3Tg and WT		PP1B_MOUSE	Serine/threonine-protein phosphatase PP1-beta catalytic subunit (PP-1B) (EC 3.1.3.16) (EC 3.1.3.53)	Ppp1cb
3Tg and WT		MYH11_MOUSE	Myosin-11 (Myosin heavy chain 11) (Myosin heavy chain, smooth muscle isoform) (SMMHC)	Myh11
3Tg and WT		6PGL_MOUSE	6-phosphogluconolactonase (6PGL) (EC 3.1.1.31)	Pgls
3Tg and WT		RL6_MOUSE	60S ribosomal protein L6 (TAX-responsive enhancer element-binding protein 107) (TAXREB107)	Rpl6
3Tg and WT		RHOA_MOUSE	Transforming protein RhoA (EC 3.6.5.2)	Rhoa
3Tg and WT		RAP1B_MOUSE	Ras-related protein Rap-1b (GTP-binding protein smg p21B)	Rap1b
3Tg and WT		CALR_MOUSE	Calreticulin (CRP55) (Calregulin) (Endoplasmic reticulum resident protein 60) (ERp60) (HACBP)	Calr
3Tg and WT		ETFA_MOUSE	Electron transfer flavoprotein subunit alpha, mitochondrial (Alpha-ETF)	Etfa
3Tg and WT		GRP75_MOUSE	Stress-70 protein, mitochondrial (75 kDa glucose-regulated protein) (GRP-75) (Heat shock 70 kDa protein 9) (Mortalin) (Peptide-binding protein 74) (PBP74) (p66 MOT)	Hspa9
3Tg and WT		CD151_MOUSE	CD151 antigen (GP27) (Membrane glycoprotein SFA-1) (Platelet-endothelial tetraspan antigen 3) (PETA-3) (CD antigen CD151)	Cd151

3Tg and WT		NDUS1_MOUSE	NADH-ubiquinone oxidoreductase 75 kDa subunit, mitochondrial (EC 1.6.99.3) (EC 7.1.1.2) (Complex I-75kD) (CI-75kD)	Ndufs1
3Tg and WT		PICAL_MOUSE	Phosphatidylinositol-binding clathrin assembly protein (Clathrin assembly lymphoid myeloid leukemia) (CALM)	Picalm
3Tg and WT		PSB7_MOUSE	Proteasome subunit beta type-7 (EC 3.4.25.1) (Macropain chain Z) (Multicatalytic endopeptidase complex chain Z) (Proteasome subunit Z)	Psmb7
3Tg and WT		CLIC1_MOUSE	Chloride intracellular channel protein 1 (Nuclear chloride ion channel 27) (NCC27)	Clic1
3Tg and WT		TALDO_MOUSE	Transaldolase (EC 2.2.1.2)	Taldo1
3Tg and WT		CAN2_MOUSE	Calpain-2 catalytic subunit (EC 3.4.22.53) (80 kDa M-calpain subunit) (CALP80) (Calcium-activated neutral proteinase 2) (CANP 2) (Calpain M-type) (Calpain-2 large subunit) (Millimolar-calpain) (M-calpain)	Capn2
3Tg and WT		DBNL_MOUSE	Drebrin-like protein (Actin-binding protein 1) (SH3 domain-containing protein 7)	Dbnl
3Tg and WT		NCS1_MOUSE	Neuronal calcium sensor 1 (NCS-1) (Frequenin homolog)	Ncs1
3Tg and WT		IF6_MOUSE	Eukaryotic translation initiation factor 6 (eIF-6) (B4 integrin interactor) (CAB) (p27(BBP))	Eif6
3Tg and WT		AIMP2_MOUSE	Aminoacyl tRNA synthase complex-interacting multifunctional protein 2 (Multisynthase complex auxiliary component p38) (Protein JTV-1)	Aimp2
3Tg and WT		ATOX1_MOUSE	Copper transport protein ATOX1 (Metal transport protein ATX1)	Atox1
3Tg and WT		CNBP_MOUSE	Cellular nucleic acid-binding protein (CNBP) (Zinc finger protein 9)	Cnbp
3Tg and WT		EIF3I_MOUSE	Eukaryotic translation initiation factor 3 subunit I (eIF3i) (Eukaryotic translation initiation factor 3 subunit 2) (TGF-beta receptor-interacting protein 1) (TRIP-1) (eIF-3-beta) (eIF3 p36)	Eif3i
3Tg and WT		KANK2_MOUSE	KN motif and ankyrin repeat domain-containing protein 2 (Ankyrin repeat domain-containing protein 25)	Kank2
3Tg and WT		CH10_MOUSE	10 kDa heat shock protein, mitochondrial (Hsp10) (10 kDa chaperonin) (Chaperonin 10) (CPN10)	Hspe1
3Tg and WT		HNRPL_MOUSE	Heterogeneous nuclear ribonucleoprotein L (hnRNP L)	Hnrnpl
3Tg and WT		MCM4_MOUSE	DNA replication licensing factor MCM4 (EC 3.6.4.12) (CDC21 homolog) (P1-CDC21)	Mcm4
3Tg and WT		FSCN1_MOUSE	Fascin (Singed-like protein)	Fscn1
3Tg and WT		THIKB_MOUSE	3-ketoacyl-CoA thiolase B, peroxisomal (EC 2.3.1.155) (EC 2.3.1.16) (EC 2.3.1.9) (Acetyl-CoA acyltransferase B) (Beta-ketothiolase B) (Peroxisomal 3-oxoacyl-CoA thiolase B)	Acaa1b
3Tg and WT		HNRPQ_MOUSE	Heterogeneous nuclear ribonucleoprotein Q (hnRNP Q) (Glycine- and tyrosine-rich RNA-binding protein) (GRY-RBP) (NS1-associated protein 1) (Synaptotagmin-binding, cytoplasmic RNA-interacting protein) (pp68)	Syncrip
3Tg and WT		RAB1B_MOUSE	Ras-related protein Rab-1B	Rab1b
3Tg and WT		PTK7_MOUSE	Inactive tyrosine-protein kinase 7 (Protein chuzhoi) (Protein-tyrosine kinase 7) (Pseudo tyrosine kinase receptor 7) (Tyrosine-protein kinase-like 7)	Ptk7

3Tg and WT		SDHA_MOUSE	Succinate dehydrogenase [ubiquinone] flavoprotein subunit, mitochondrial (EC 1.3.5.1) (Flavoprotein subunit of complex II) (Fp)	Sdha
3Tg and WT		LMNA_MOUSE	Prelamin-A/C [Cleaved into: Lamin-A/C]	Lmna
3Tg and WT		MTPN_MOUSE	Myotrophin (Granule cell differentiation protein) (Protein V-1)	Mtpn
3Tg and WT		RL36_MOUSE	60S ribosomal protein L36	Rpl36
3Tg and WT		RAP1A_MOUSE	Ras-related protein Rap-1A (Ras-related protein Krev-1)	Rap1a
3Tg and WT		ACTG_MOUSE	Actin, cytoplasmic 2 (Gamma-actin) [Cleaved into: Actin, cytoplasmic 2, N-terminally processed]	Actg1
3Tg and WT		ANLN_MOUSE	Anillin	Anln
3Tg and WT		ACSL4_MOUSE	Long-chain-fatty-acid--CoA ligase 4 (EC 6.2.1.3) (Arachidonate--CoA ligase) (EC 6.2.1.15) (Long-chain acyl-CoA synthetase 4) (LACS 4) (mACS4)	Acsl4
3Tg and WT		ARC1B_MOUSE	Actin-related protein 2/3 complex subunit 1B (Arp2/3 complex 41 kDa subunit) (p41-ARC)	Arpc1b
3Tg and WT		RS27A_MOUSE	Ubiquitin-40S ribosomal protein S27a (Ubiquitin carboxyl extension protein 80) [Cleaved into: Ubiquitin; 40S ribosomal protein S27a]	Rps27a
3Tg and WT		CNN2_MOUSE	Calponin-2 (Calponin H2, smooth muscle) (Neutral calponin)	Cnn2
3Tg and WT		RRAS2_MOUSE	Ras-related protein R-Ras2	Rras2
3Tg and WT		COX5A_MOUSE	Cytochrome c oxidase subunit 5A, mitochondrial (Cytochrome c oxidase polypeptide Va)	Cox5a
3Tg and WT		GSTM1_MOUSE	Glutathione S-transferase Mu 1 (EC 2.5.1.18) (GST 1-1) (GST class-mu 1) (Glutathione S-transferase GT8.7) (pmGT10)	Gstm1
3Tg and WT		LMNB1_MOUSE	Lamin-B1	Lmnb1
3Tg and WT		SH3L1_MOUSE	SH3 domain-binding glutamic acid-rich-like protein	Sh3bgrl
3Tg and WT		BASP1_MOUSE	Brain acid soluble protein 1 (22 kDa neuronal tissue-enriched acidic protein) (Neuronal axonal membrane protein NAP-22)	Basp1
3Tg and WT		TCTP_MOUSE	Translationally-controlled tumor protein (TCTP) (21 kDa polypeptide) (p21) (p23)	Tpt1
3Tg and WT		VATB2_MOUSE	V-type proton ATPase subunit B, brain isoform (V-ATPase subunit B 2) (Endomembrane proton pump 58 kDa subunit) (Vacuolar proton pump subunit B 2)	Atp6v1b2
3Tg and WT		ADA10_MOUSE	Disintegrin and metalloproteinase domain-containing protein 10 (ADAM 10) (EC 3.4.24.81) (Kuzbanian protein homolog) (Mammalian disintegrin-metalloprotease) (CD antigen CD156c)	Adam10
3Tg and WT		KPYM_MOUSE	Pyruvate kinase PKM (EC 2.7.1.40) (Pyruvate kinase muscle isozyme)	Pkm
3Tg and WT		RL14_MOUSE	60S ribosomal protein L14	Rpl14
3Tg and WT		FUBP2_MOUSE	Far upstream element-binding protein 2 (FUSE-binding protein 2) (KH type-splicing regulatory protein) (KSRP)	Khsrp
3Tg and WT		FERM2_MOUSE	Fermitin family homolog 2 (Kindlin-2) (Pleckstrin homology domain-containing family C member 1)	Fermt2
3Tg and WT		ITA5_MOUSE	Integrin alpha-5 (CD49 antigen-like family member E) (Fibronectin receptor subunit alpha) (Integrin alpha-F) (VLA-5) (CD antigen CD49e) [Cleaved into: Integrin alpha-5 heavy chain; Integrin alpha-5 light chain]	Itga5

3Tg and WT		IF5A1_MOUSE	Eukaryotic translation initiation factor 5A-1 (eIF-5A-1) (eIF-5A1) (Eukaryotic initiation factor 5A isoform 1) (eIF-5A) (eIF-4D)	Eif5a
3Tg and WT		CADH9_MOUSE	Cadherin-9 (T1-cadherin)	Cdh9
3Tg and WT		TCPG_MOUSE	T-complex protein 1 subunit gamma (TCP-1-gamma) (CCT-gamma) (Matricin) (mTRiC-P5)	Cct3
3Tg and WT		LEG1_MOUSE	Galectin-1 (Gal-1) (14 kDa lectin) (Beta-galactoside-binding lectin L-14-I) (Galaptin) (Lactose-binding lectin 1) (Lectin galactoside-binding soluble 1) (S-Lac lectin 1)	Lgals1
3Tg and WT		DLDH_MOUSE	Dihydrolipoyl dehydrogenase, mitochondrial (EC 1.8.1.4) (Dihydrolipoamide dehydrogenase)	Dld
3Tg and WT		PSMD9_MOUSE	26S proteasome non-ATPase regulatory subunit 9 (26S proteasome regulatory subunit p27)	Psmd9
3Tg and WT		RINI_MOUSE	Ribonuclease inhibitor (Ribonuclease/angiogenin inhibitor 1)	Rnh1
3Tg and WT		RL32P_MOUSE	Putative 60S ribosomal protein L32'	Rpl32-ps
3Tg and WT		B2MG_MOUSE	Beta-2-microglobulin	B2m
3Tg and WT		PUR6_MOUSE	Multifunctional protein ADE2 [Includes: Phosphoribosylaminoimidazole-succinocarboxamide synthase (EC 6.3.2.6) (SAICAR synthetase); Phosphoribosylaminoimidazole carboxylase (EC 4.1.1.21) (AIR carboxylase) (AIRC)]	Paics
3Tg and WT		DHRS1_MOUSE	Dehydrogenase/reductase SDR family member 1 (EC 1.1.-.-)	Dhrs1
3Tg and WT		RASK_MOUSE	GTPase KRas (K-Ras 2) (Ki-Ras) (c-K-ras) (c-Ki-ras) [Cleaved into: GTPase KRas, N-terminally processed]	Kras
3Tg and WT		NSF_MOUSE	Vesicle-fusing ATPase (EC 3.6.4.6) (N-ethylmaleimide-sensitive fusion protein) (NEM-sensitive fusion protein) (Suppressor of K(+) transport growth defect 2) (Protein SKD2) (Vesicular-fusion protein NSF)	Nsf
3Tg and WT		PROF1_MOUSE	Profilin-1 (Profilin I)	Pfn1
3Tg and WT		RL18_MOUSE	60S ribosomal protein L18	Rpl18
3Tg and WT		TXD12_MOUSE	Thioredoxin domain-containing protein 12 (EC 1.8.4.2) (Endoplasmic reticulum resident protein 19) (ER protein 19) (ERp19) (Thioredoxin-like protein p19)	Txndc12
3Tg and WT		MIF_MOUSE	Macrophage migration inhibitory factor (MIF) (EC 5.3.2.1) (Delayed early response protein 6) (DER6) (Glycosylation-inhibiting factor) (GIF) (L-dopachrome isomerase) (L-dopachrome tautomerase) (EC 5.3.3.12) (Phenylpyruvate tautomerase)	Mif
3Tg and WT		SYTC_MOUSE	Threonine--tRNA ligase 1, cytoplasmic (EC 6.1.1.3) (Threonine--tRNA ligase, cytoplasmic) (Threonyl-tRNA synthetase) (ThrRS) (Threonyl-tRNA synthetase 1)	Tars1
3Tg and WT		GSTP1_MOUSE	Glutathione S-transferase P 1 (Gst P1) (EC 2.5.1.18) (GST YF-YF) (GST class-pi) (GST-piB) (Preadipocyte growth factor)	Gstp1
3Tg and WT		SYNE2_MOUSE	Nesprin-2 (KASH domain-containing protein 2) (KASH2) (Nuclear envelope spectrin repeat protein 2) (Nucleus and actin connecting element protein) (Protein NUANCE) (Synaptic nuclear envelope protein 2) (Syne-2)	Syne2
3Tg and WT		RS15A_MOUSE	40S ribosomal protein S15a	Rps15a

3Tg and WT		ENOA_MOUSE	Alpha-enolase (EC 4.2.1.11) (2-phospho-D-glycerate hydro-lyase) (Enolase 1) (Non-neural enolase) (NNE)	Eno1
3Tg and WT		G3BP1_MOUSE	Ras GTPase-activating protein-binding protein 1 (G3BP-1) (EC 3.6.4.12) (EC 3.6.4.13) (ATP-dependent DNA helicase VIII) (GAP SH3 domain-binding protein 1) (HDH-VIII)	G3bp1
3Tg and WT		ML12B_MOUSE	Myosin regulatory light chain 12B (Myosin regulatory light chain 2-B, smooth muscle isoform) (Myosin regulatory light chain 20 kDa) (MLC20) (Myosin regulatory light chain MRLC2)	Myl12b
3Tg and WT		ALDR_MOUSE	Aldo-keto reductase family 1 member B1 (EC 1.1.1.300) (EC 1.1.1.372) (EC 1.1.1.54) (Aldehyde reductase) (Aldo-keto reductase family 1 member B3) (Aldose reductase) (AR) (EC 1.1.1.21)	Akr1b1
3Tg and WT		COPD_MOUSE	Coatomer subunit delta (Archain) (Delta-coat protein) (Delta-COP)	Arcn1
3Tg and WT		CKAP4_MOUSE	Cytoskeleton-associated protein 4 (63-kDa cytoskeleton-linking membrane protein) (Climp-63) (p63)	Ckap4
3Tg and WT		RENT1_MOUSE	Regulator of nonsense transcripts 1 (EC 3.6.4.-) (ATP-dependent helicase RENT1) (Nonsense mRNA reducing factor 1) (NORF1) (Up-frameshift suppressor 1 homolog) (mUpf1)	Upf1
3Tg and WT		GNAS2_MOUSE	Guanine nucleotide-binding protein G(s) subunit alpha isoforms short (Adenylate cyclase-stimulating G alpha protein)	Gnas
3Tg and WT		VAPB_MOUSE	Vesicle-associated membrane protein-associated protein B (VAMP-B) (VAMP-associated protein B) (VAP-B) (VAMP-associated protein 33b)	Vapb
3Tg and WT		VINC_MOUSE	Vinculin (Metavinculin)	Vcl
3Tg and WT		GNAS1_MOUSE	Guanine nucleotide-binding protein G(s) subunit alpha isoforms XLas (Adenylate cyclase-stimulating G alpha protein) (Extra large alphas protein) (XLalphas)	Gnas
3Tg and WT		PRDX2_MOUSE	Peroxiredoxin-2 (EC 1.11.1.15) (Thiol-specific antioxidant protein) (TSA) (Thioredoxin peroxidase 1) (Thioredoxin-dependent peroxide reductase 1)	Prdx2
3Tg and WT		RS9_MOUSE	40S ribosomal protein S9	Rps9
3Tg and WT		ENV1_MOUSE	MLV-related proviral Env polyprotein [Cleaved into: Surface protein (SU); Transmembrane protein (TM)]	Viral
3Tg and WT		RL19_MOUSE	60S ribosomal protein L19	Rpl19
3Tg and WT		GBB1_MOUSE	Guanine nucleotide-binding protein G(I)/G(S)/G(T) subunit beta-1 (Transducin beta chain 1)	Gnb1
3Tg and WT		LRRF1_MOUSE	Leucine-rich repeat flightless-interacting protein 1 (LRR FLII-interacting protein 1) (FLI-LRR-associated protein 1) (Flap-1) (H186 FLAP)	Lrrfip1
3Tg and WT		K1671_MOUSE	Uncharacterized protein KIAA1671	Kiaa1671
3Tg and WT		PFKAP_MOUSE	ATP-dependent 6-phosphofructokinase, platelet type (ATP-PFK) (PFK-P) (EC 2.7.1.11) (6-phosphofructokinase type C) (Phosphofructo-1-kinase isozyme C) (PFK-C) (Phosphohexokinase)	Pfkp
3Tg and WT		RB11B_MOUSE	Ras-related protein Rab-11B	Rab11b
3Tg and WT		TBA1A_MOUSE	Tubulin alpha-1A chain (Alpha-tubulin 1) (Alpha-tubulin isotype M-alpha-1) (Tubulin alpha-1 chain) [Cleaved into: Detyrosinated tubulin alpha-1A chain]	Tuba1a

3Tg and WT		EMC8_MOUSE	ER membrane protein complex subunit 8 (Neighbor of COX4)	Emc8
3Tg and WT		ATPA_MOUSE	ATP synthase subunit alpha, mitochondrial (ATP synthase F1 subunit alpha)	Atp5f1a
3Tg and WT		GNAI2_MOUSE	Guanine nucleotide-binding protein G(i) subunit alpha-2 (Adenylate cyclase-inhibiting G alpha protein)	Gnai2
3Tg and WT		ARF6_MOUSE	ADP-ribosylation factor 6	Arf6
3Tg and WT		NRADD_MOUSE	Death domain-containing membrane protein NRADD (Neurotrophin receptor homolog-2) (NRH2) (Neurotrophin receptor-alike death domain protein)	Nradd
3Tg and WT		AP2A2_MOUSE	AP-2 complex subunit alpha-2 (100 kDa coated vesicle protein C) (Adaptor protein complex AP-2 subunit alpha-2) (Adaptor-related protein complex 2 subunit alpha-2) (Alpha-adaptin C) (Alpha2-adaptin) (Clathrin assembly protein complex 2 alpha-C large chain) (Plasma membrane adaptor HA2/AP2 adaptin alpha C subunit)	Ap2a2
3Tg and WT		NEDD4_MOUSE	E3 ubiquitin-protein ligase NEDD4 (EC 2.3.2.26) (HECT-type E3 ubiquitin transferase NEDD4) (Neural precursor cell expressed developmentally down-regulated protein 4) (NEDD-4)	Nedd4
3Tg and WT		ERBIN_MOUSE	Erbin (Densin-180-like protein) (Erbb2-interacting protein) (Protein LAP2)	Erbin
3Tg and WT		IGF1R_MOUSE	Insulin-like growth factor 1 receptor (EC 2.7.10.1) (Insulin-like growth factor I receptor) (IGF-I receptor) (CD antigen CD221) [Cleaved into: Insulin-like growth factor 1 receptor alpha chain; Insulin-like growth factor 1 receptor beta chain]	Igf1r
3Tg and WT		DC1I2_MOUSE	Cytoplasmic dynein 1 intermediate chain 2 (Cytoplasmic dynein intermediate chain 2) (Dynein intermediate chain 2, cytosolic) (DH IC-2)	Dync1i2
3Tg and WT		ESYT1_MOUSE	Extended synaptotagmin-1 (E-Syt1) (Membrane-bound C2 domain-containing protein)	Esyt1
3Tg and WT		RUVB2_MOUSE	RuvB-like 2 (EC 3.6.4.12) (p47 protein)	Ruvbl2
3Tg and WT		C1TC_MOUSE	C-1-tetrahydrofolate synthase, cytoplasmic (C1-THF synthase) [Cleaved into: C-1-tetrahydrofolate synthase, cytoplasmic, N-terminally processed] [Includes: Methylenetetrahydrofolate dehydrogenase (EC 1.5.1.5); Methenyltetrahydrofolate cyclohydrolase (EC 3.5.4.9); Formyltetrahydrofolate synthetase (EC 6.3.4.3)]	Mthfd1
3Tg and WT		COX41_MOUSE	Cytochrome c oxidase subunit 4 isoform 1, mitochondrial (Cytochrome c oxidase polypeptide IV) (Cytochrome c oxidase subunit IV isoform 1) (COX IV-1)	Cox4i1
3Tg and WT		UGDH_MOUSE	UDP-glucose 6-dehydrogenase (UDP-Glc dehydrogenase) (UDP-GlcDH) (UDPGDH) (EC 1.1.1.22)	Ugdh
3Tg and WT		MLEC_MOUSE	Malectin	Mlec
3Tg and WT		HMCS1_MOUSE	Hydroxymethylglutaryl-CoA synthase, cytoplasmic (HMG-CoA synthase) (EC 2.3.3.10) (3-hydroxy-3-methylglutaryl coenzyme A synthase)	Hmgcs1
3Tg and WT		DDAH1_MOUSE	N(G),N(G)-dimethylarginine dimethylaminohydrolase 1 (DDAH-1) (Dimethylarginine dimethylaminohydrolase 1) (EC 3.5.3.18) (DDAHI) (Dimethylargininase-1)	Ddah1

3Tg and WT		EF1D_MOUSE	Elongation factor 1-delta (EF-1-delta)	Eef1d
3Tg and WT		RALB_MOUSE	Ras-related protein Ral-B	Ralb
3Tg and WT		DREB_MOUSE	Drebrin (Developmentally-regulated brain protein)	Dbn1
3Tg and WT		HDAC4_MOUSE	Histone deacetylase 4 (HD4) (EC 3.5.1.98)	Hdac4
3Tg and WT		PRDX5_MOUSE	Peroxiredoxin-5, mitochondrial (EC 1.11.1.15) (Antioxidant enzyme B166) (AOEB166) (Liver tissue 2D-page spot 2D-0014IV) (PLP) (Peroxiredoxin V) (Prx-V) (Peroxisomal antioxidant enzyme) (Thioredoxin peroxidase PMP20)	Prdx5
3Tg and WT		REXO4_MOUSE	RNA exonuclease 4 (EC 3.1.-.-) (Exonuclease XPMC2) (Prevents mitotic catastrophe 2 protein homolog)	Rexo4
3Tg and WT		RS8_MOUSE	40S ribosomal protein S8	Rps8
3Tg and WT		H4_MOUSE	Histone H4	H4c1
3Tg and WT		RAB5B_MOUSE	Ras-related protein Rab-5B	Rab5b
3Tg and WT		HS105_MOUSE	Heat shock protein 105 kDa (42 degrees C-HSP) (Heat shock 110 kDa protein) (Heat shock-related 100 kDa protein E7I) (HSP-E7I)	Hsph1
3Tg and WT		GTR1_MOUSE	Solute carrier family 2, facilitated glucose transporter member 1 (Glucose transporter type 1, erythrocyte/brain) (GLUT-1) (GT1)	Slc2a1
3Tg and WT		RL32_MOUSE	60S ribosomal protein L32	Rpl32
3Tg and WT		DPYL2_MOUSE	Dihydropyrimidinase-related protein 2 (DRP-2) (Unc-33-like phosphoprotein 2) (ULIP-2)	Dpysl2
3Tg and WT		NDKA_MOUSE	Nucleoside diphosphate kinase A (NDK A) (NDP kinase A) (EC 2.7.4.6) (Metastasis inhibition factor NM23) (NDPK-A) (Tumor metastatic process-associated protein) (nm23-M1)	Nme1
3Tg and WT		SGPL1_MOUSE	Sphingosine-1-phosphate lyase 1 (S1PL) (SP-lyase 1) (SPL 1) (mSPL) (EC 4.1.2.27) (Sphingosine-1-phosphate aldolase)	Sgpl1
3Tg and WT		RAB7A_MOUSE	Ras-related protein Rab-7a	Rab7a
3Tg and WT		RS26_MOUSE	40S ribosomal protein S26	Rps26
3Tg and WT		NCKP1_MOUSE	Nck-associated protein 1 (NAP 1) (Brain protein H19) (MH19) (Membrane-associated protein HEM-2) (p125Nap1)	Nckap1
3Tg and WT		COF1_MOUSE	Cofilin-1 (Cofilin, non-muscle isoform)	Cfl1
3Tg and WT		CPNE2_MOUSE	Copine-2 (Copine II)	Cpne2
3Tg and WT		MYL6_MOUSE	Myosin light polypeptide 6 (17 kDa myosin light chain) (LC17) (Myosin light chain 3) (MLC-3) (Myosin light chain alkali 3) (Myosin light chain A3) (Smooth muscle and nonmuscle myosin light chain alkali 6)	Myl6
3Tg and WT		SYEP_MOUSE	Bifunctional glutamate/proline--tRNA ligase (Bifunctional aminoacyl-tRNA synthetase) [Includes: Glutamate--tRNA ligase (EC 6.1.1.17) (Glutamyl-tRNA synthetase) (GluRS); Proline--tRNA ligase (EC 6.1.1.15) (Prolyl-tRNA synthetase) (ProRS)]	Eprs
3Tg and WT		ANXA2_MOUSE	Annexin A2 (Annexin II) (Annexin-2) (Calpactin I heavy chain) (Calpactin-1 heavy chain) (Chromobindin-8) (Lipocortin II) (Placental anticoagulant protein IV) (PAP-IV) (Protein I) (p36)	Anxa2
3Tg and WT		SYSC_MOUSE	Serine--tRNA ligase, cytoplasmic (EC 6.1.1.11) (Seryl-tRNA synthetase) (SerRS) (Seryl-tRNA(Ser/Sec) synthetase)	Sars

3Tg and WT		TCPA_MOUSE	T-complex protein 1 subunit alpha (TCP-1-alpha) (CCT-alpha) (Tailless complex polypeptide 1A) (TCP-1-A) (Tailless complex polypeptide 1B) (TCP-1-B)	Tcp1
3Tg and WT		ADT2_MOUSE	ADP/ATP translocase 2 (ADP,ATP carrier protein 2) (Adenine nucleotide translocator 2) (ANT 2) (Solute carrier family 25 member 5) [Cleaved into: ADP/ATP translocase 2, N-terminally processed]	Slc25a5
3Tg and WT		UGGG1_MOUSE	UDP-glucose:glycoprotein glucosyltransferase 1 (UGT1) (EC 2.4.1.-) (UDP--Glc:glycoprotein glucosyltransferase) (UDP-glucose ceramide glucosyltransferase-like 1)	Uggt1
3Tg and WT		PNO1_MOUSE	RNA-binding protein PNO1	Pno1
3Tg and WT		VAT1_MOUSE	Synaptic vesicle membrane protein VAT-1 homolog (EC 1.-.-.-)	Vat1
3Tg and WT		PSD13_MOUSE	26S proteasome non-ATPase regulatory subunit 13 (26S proteasome regulatory subunit RPN9) (26S proteasome regulatory subunit S11) (26S proteasome regulatory subunit p40.5)	Psmd13
3Tg and WT		DHB4_MOUSE	Peroxisomal multifunctional enzyme type 2 (MFE-2) (17-beta-hydroxysteroid dehydrogenase 4) (17-beta-HSD 4) (D-bifunctional protein) (DBP) (Multifunctional protein 2) (MPF-2) [Cleaved into: (3R)-hydroxyacyl-CoA dehydrogenase (EC 1.1.1.n12); Enoyl-CoA hydratase 2 (EC 4.2.1.107) (EC 4.2.1.119) (3-alpha,7-alpha,12-alpha-trihydroxy-5-beta-cholest-24-enoyl-CoA hydratase)]	Hsd17b4
3Tg and WT		SEPT2_MOUSE	Septin-2 (Neural precursor cell expressed developmentally down-regulated protein 5) (NEDD-5)	Septin2
3Tg and WT		NCAM1_MOUSE	Neural cell adhesion molecule 1 (N-CAM-1) (NCAM-1) (CD antigen CD56)	Ncam1
3Tg and WT		SUMO3_MOUSE	Small ubiquitin-related modifier 3 (SUMO-3) (SMT3 homolog 1) (Ubiquitin-like protein SMT3A) (Smt3A)	Sumo3
3Tg and WT		DYHC1_MOUSE	Cytoplasmic dynein 1 heavy chain 1 (Cytoplasmic dynein heavy chain 1) (Dynein heavy chain, cytosolic)	Dync1h1
3Tg and WT		ENPL_MOUSE	Endoplasmin (94 kDa glucose-regulated protein) (GRP-94) (Endoplasmic reticulum resident protein 99) (ERp99) (Heat shock protein 90 kDa beta member 1) (Polymorphic tumor rejection antigen 1) (Tumor rejection antigen gp96)	Hsp90b1
3Tg and WT		RB11A_MOUSE	Ras-related protein Rab-11A (Rab-11)	Rab11a
3Tg and WT		KAPCA_MOUSE	cAMP-dependent protein kinase catalytic subunit alpha (PKA C-alpha) (EC 2.7.11.11)	Prkaca
3Tg and WT		CD9_MOUSE	CD9 antigen (CD antigen CD9)	Cd9
3Tg and WT		NONO_MOUSE	Non-POU domain-containing octamer-binding protein (NonO protein)	Nono
3Tg and WT		H13_MOUSE	Histone H1.3 (H1 VAR.4) (H1d)	H1-3
3Tg and WT		TPM2_MOUSE	Tropomyosin beta chain (Beta-tropomyosin) (Tropomyosin-2)	Tpm2
3Tg and WT		AACS_MOUSE	Acetoacetyl-CoA synthetase (EC 6.2.1.16)	Aacs
3Tg and WT		NIBL1_MOUSE	Protein Niban 2 (Meg-3) (Niban-like protein 1) (Protein FAM129B)	Niban2
3Tg and WT		BTF3_MOUSE	Transcription factor BTF3 (Nascent polypeptide-associated complex subunit beta) (NAC-beta) (RNA polymerase B transcription factor 3)	Btf3

3Tg and WT		NCEH1_MOUSE	Neutral cholesterol ester hydrolase 1 (NCEH) (EC 3.1.1.-) (Arylacetamide deacetylase-like 1) (Chlorpyrifos oxon-binding protein) (CPO-BP)	Nceh1
3Tg and WT		TIF1B_MOUSE	Transcription intermediary factor 1-beta (TIF1-beta) (E3 SUMO-protein ligase TRIM28) (EC 2.3.2.27) (KRAB-A-interacting protein) (KRIP-1) (RING-type E3 ubiquitin transferase TIF1-beta) (Tripartite motif-containing protein 28)	Trim28
3Tg and WT		TEBP_MOUSE	Prostaglandin E synthase 3 (EC 5.3.99.3) (Cytosolic prostaglandin E2 synthase) (cPGES) (Hsp90 co-chaperone) (Progesterone receptor complex p23) (Sid 3177) (Telomerase-binding protein p23)	Ptges3
3Tg and WT		ADT1_MOUSE	ADP/ATP translocase 1 (ADP,ATP carrier protein 1) (ADP,ATP carrier protein, heart/skeletal muscle isoform T1) (Adenine nucleotide translocator 1) (ANT 1) (Solute carrier family 25 member 4) (mANC1)	Slc25a4
3Tg and WT		KINH_MOUSE	Kinesin-1 heavy chain (Conventional kinesin heavy chain) (Ubiquitous kinesin heavy chain) (UKHC)	Kif5b
3Tg and WT		CAPG_MOUSE	Macrophage-capping protein (Actin regulatory protein CAP-G) (Actin-capping protein GCAP39) (Myc basic motif homolog 1)	Capg
3Tg and WT		IF4B_MOUSE	Eukaryotic translation initiation factor 4B (eIF-4B)	Eif4b
3Tg and WT		GRN_MOUSE	Progranulin (PGRN) (Acrogranin) (Epithelin/granulin precursor) (Glycoprotein of 88 Kda) (GP88) (Glycoprotein 88) (PC cell-derived growth factor) (PCDGF) (Proepithelin) (PEPI) [Cleaved into: Paragranulin; Granulin-1; Granulin-2; Granulin-3; Granulin-4; Granulin-5; Granulin-6; Granulin-7]	Grn
3Tg and WT		CADM1_MOUSE	Cell adhesion molecule 1 (Immunoglobulin superfamily member 4) (IgSF4) (Nectin-like protein 2) (NECL-2) (Spermatogenic immunoglobulin superfamily) (SgIgSF) (Synaptic cell adhesion molecule) (SynCAM) (Tumor suppressor in lung cancer 1) (TSLC-1)	Cadm1
3Tg and WT		TBB2A_MOUSE	Tubulin beta-2A chain	Tubb2a
3Tg and WT		P4HA1_MOUSE	Prolyl 4-hydroxylase subunit alpha-1 (4-PH alpha-1) (EC 1.14.11.2) (Procollagen-proline,2-oxoglutarate-4-dioxygenase subunit alpha-1)	P4ha1
3Tg and WT		FKB1A_MOUSE	Peptidyl-prolyl cis-trans isomerase FKBP1A (PPIase FKBP1A) (EC 5.2.1.8) (12 kDa FK506-binding protein) (12 kDa FKBP) (FKBP-12) (Calstabin-1) (FK506-binding protein 1A) (FKBP-1A) (Immunophilin FKBP12) (Rotamase)	Fkbp1a
3Tg and WT		UB2V2_MOUSE	Ubiquitin-conjugating enzyme E2 variant 2 (Ubc-like protein MMS2)	Ube2v2
3Tg and WT		LDHA_MOUSE	L-lactate dehydrogenase A chain (LDH-A) (EC 1.1.1.27) (LDH muscle subunit) (LDH-M)	Ldha
3Tg and WT		EF1B_MOUSE	Elongation factor 1-beta (EF-1-beta)	Eef1b
3Tg and WT		VAMP3_MOUSE	Vesicle-associated membrane protein 3 (VAMP-3) (Cellubrevin) (CEB) (Synaptobrevin-3)	Vamp3
3Tg and WT		LIMS1_MOUSE	LIM and senescent cell antigen-like-containing domain protein 1 (Particularly interesting new Cys-His protein 1) (PINCH-1)	Lims1
3Tg and WT		IDHP_MOUSE	Isocitrate dehydrogenase [NADP], mitochondrial (IDH) (EC 1.1.1.42) (ICD-M) (IDP) (NADP(+)-specific ICDH) (Oxalosuccinate decarboxylase)	Idh2

3Tg and WT		IST1_MOUSE	IST1 homolog	Ist1
3Tg and WT		ANXA7_MOUSE	Annexin A7 (Annexin VII) (Annexin-7) (Synexin)	Anxa7
3Tg and WT		AT2B4_MOUSE	Plasma membrane calcium-transporting ATPase 4 (PMCA4) (EC 7.2.2.10)	Atp2b4
3Tg and WT		CNN3_MOUSE	Calponin-3 (Calponin, acidic isoform)	Cnn3
3Tg and WT		GRP78_MOUSE	Endoplasmic reticulum chaperone BiP (EC 3.6.4.10) (78 kDa glucose-regulated protein) (GRP-78) (Binding-immunoglobulin protein) (BiP) (Heat shock protein 70 family protein 5) (HSP70 family protein 5) (Heat shock protein family A member 5) (Immunoglobulin heavy chain-binding protein)	Hspa5
3Tg and WT		H2B1C_MOUSE	Histone H2B type 1-C/E/G	H2bc4
3Tg and WT		CADH2_MOUSE	Cadherin-2 (Neural cadherin) (N-cadherin) (CD antigen CD325)	Cdh2
3Tg and WT		BSN_MOUSE	Protein bassoon	Bsn
3Tg and WT		EIF3A_MOUSE	Eukaryotic translation initiation factor 3 subunit A (eIF3a) (Centrosomin) (Eukaryotic translation initiation factor 3 subunit 10) (eIF-3-theta) (eIF3 p167) (eIF3 p180) (eIF3 p185) (p162)	Eif3a
3Tg and WT		RL31_MOUSE	60S ribosomal protein L31	Rpl31
3Tg and WT		E41L2_MOUSE	Band 4.1-like protein 2 (Generally expressed protein 4.1) (4.1G)	Epb41l2
3Tg and WT		UBP2L_MOUSE	Ubiquitin-associated protein 2-like	Ubap2l
3Tg and WT		KAD1_MOUSE	Adenylate kinase isoenzyme 1 (AK 1) (EC 2.7.4.3) (EC 2.7.4.6) (ATP-AMP transphosphorylase 1) (ATP:AMP phosphotransferase) (Adenylate monophosphate kinase) (Myokinase)	Ak1
3Tg and WT		GDIR1_MOUSE	Rho GDP-dissociation inhibitor 1 (Rho GDI 1) (GDI-1) (Rho-GDI alpha)	Arhgdia
3Tg and WT		PTTG_MOUSE	Pituitary tumor-transforming gene 1 protein-interacting protein (Pituitary tumor-transforming gene protein-binding factor) (PBF) (PTTG-binding factor)	Pttg1ip
3Tg and WT		RL7A_MOUSE	60S ribosomal protein L7a (Surfeit locus protein 3)	Rpl7a
3Tg and WT		VDAC1_MOUSE	Voltage-dependent anion-selective channel protein 1 (VDAC-1) (mVDAC1) (Outer mitochondrial membrane protein porin 1) (Plasmalemmal porin) (Voltage-dependent anion-selective channel protein 5) (VDAC-5) (mVDAC5)	Vdac1
3Tg and WT		ANXA5_MOUSE	Annexin A5 (Anchorin CII) (Annexin V) (Annexin-5) (Calphobindin I) (CBP-I) (Endonexin II) (Lipocortin V) (Placental anticoagulant protein 4) (PP4) (Placental anticoagulant protein I) (PAP-I) (Thromboplastin inhibitor) (Vascular anticoagulant-alpha) (VAC-alpha)	Anxa5
3Tg and WT		PHB_MOUSE	Prohibitin (B-cell receptor-associated protein 32) (BAP 32)	Phb
3Tg and WT		6PGD_MOUSE	6-phosphogluconate dehydrogenase, decarboxylating (EC 1.1.1.44)	Pgd
3Tg and WT		SEPT9_MOUSE	Septin-9 (SL3-3 integration site 1 protein)	Septin9
3Tg and WT		PALM_MOUSE	Paralemmin-1 (Paralemmin)	Palm
3Tg and WT		AT2A1_MOUSE	Sarcoplasmic/endoplasmic reticulum calcium ATPase 1 (SERCA1) (SR Ca(2+)-ATPase 1) (EC 7.2.2.10) (Calcium pump 1) (Calcium-transporting ATPase sarcoplasmic reticulum type, fast twitch skeletal muscle isoform) (Endoplasmic reticulum class 1/2 Ca(2+) ATPase)	Atp2a1
3Tg and WT		ACTN4_MOUSE	Alpha-actinin-4 (Non-muscle alpha-actinin 4)	Actn4

3Tg and WT		MIC60_MOUSE	MICOS complex subunit Mic60 (Mitochondrial inner membrane protein) (Mitofilin)	Immt
3Tg and WT		TCPQ_MOUSE	T-complex protein 1 subunit theta (TCP-1-theta) (CCT-theta)	Cct8
3Tg and WT		TCPB_MOUSE	T-complex protein 1 subunit beta (TCP-1-beta) (CCT-beta)	Cct2
3Tg and WT		PTBP1_MOUSE	Polypyrimidine tract-binding protein 1 (PTB) (Heterogeneous nuclear ribonucleoprotein I) (hnRNP I)	Ptbp1
3Tg and WT		STIP1_MOUSE	Stress-induced-phosphoprotein 1 (STI1) (mSTI1) (Hsc70/Hsp90-organizing protein) (Hop)	Stip1
3Tg and WT		RAB5A_MOUSE	Ras-related protein Rab-5A (EC 3.6.5.2)	Rab5a
3Tg and WT		RS19_MOUSE	40S ribosomal protein S19	Rps19
3Tg and WT		RL29_MOUSE	60S ribosomal protein L29	Rpl29
3Tg and WT		MK01_MOUSE	Mitogen-activated protein kinase 1 (MAP kinase 1) (MAPK 1) (EC 2.7.11.24) (ERT1) (Extracellular signal-regulated kinase 2) (ERK-2) (MAP kinase isoform p42) (p42-MAPK) (Mitogen-activated protein kinase 2) (MAP kinase 2) (MAPK 2)	Mapk1
3Tg and WT		CTND1_MOUSE	Catenin delta-1 (Cadherin-associated Src substrate) (CAS) (p120 catenin) (p120(ctn)) (p120(cas))	Ctnnd1
3Tg and WT		RAB14_MOUSE	Ras-related protein Rab-14	Rab14
3Tg and WT		RL8_MOUSE	60S ribosomal protein L8	Rpl8
3Tg and WT		RHOG_MOUSE	Rho-related GTP-binding protein RhoG (Sid 10750)	Rhog
3Tg and WT		1433G_MOUSE	14-3-3 protein gamma [Cleaved into: 14-3-3 protein gamma, N-terminally processed]	Ywhag
3Tg and WT		IQGA1_MOUSE	Ras GTPase-activating-like protein IQGAP1	Iqgap1
3Tg and WT		PALLD_MOUSE	Palladin	Palld
3Tg and WT		MARE1_MOUSE	Microtubule-associated protein RP/EB family member 1 (APC-binding protein EB1) (End-binding protein 1) (EB1)	Mapre1
3Tg and WT		KPCA_MOUSE	Protein kinase C alpha type (PKC-A) (PKC-alpha) (EC 2.7.11.13)	Prkca
3Tg and WT		PDIA3_MOUSE	Protein disulfide-isomerase A3 (EC 5.3.4.1) (58 kDa glucose-regulated protein) (58 kDa microsomal protein) (p58) (Disulfide isomerase ER-60) (Endoplasmic reticulum resident protein 57) (ER protein 57) (ERp57) (Endoplasmic reticulum resident protein 60) (ER protein 60) (ERp60)	Pdia3
3Tg and WT		LASP1_MOUSE	LIM and SH3 domain protein 1 (LASP-1) (Metastatic lymph node gene 50 protein) (MLN 50)	Lasp1
3Tg and WT		FKBP9_MOUSE	Peptidyl-prolyl cis-trans isomerase FKBP9 (PPIase FKBP9) (EC 5.2.1.8) (63 kDa FK506-binding protein) (63 kDa FKBP) (FKBP-63) (FK506-binding protein 9) (FKBP-9) (FKBP65RS) (Rotamase)	Fkbp9
3Tg and WT		AATM_MOUSE	Aspartate aminotransferase, mitochondrial (mAspAT) (EC 2.6.1.1) (EC 2.6.1.7) (Fatty acid-binding protein) (FABP-1) (Glutamate oxaloacetate transaminase 2) (Kynurenine aminotransferase 4) (Kynurenine aminotransferase IV) (Kynurenine--oxoglutarate transaminase 4) (Kynurenine--oxoglutarate transaminase IV) (Plasma membrane-associated fatty acid-binding protein) (FABPpm) (Transaminase A)	Got2

3Tg and WT		FKB10_MOUSE	Peptidyl-prolyl cis-trans isomerase FKBP10 (PPIase FKBP10) (EC 5.2.1.8) (65 kDa FK506-binding protein) (65 kDa FKBP) (FKBP-65) (FK506-binding protein 10) (FKBP-10) (Immunophilin FKBP65) (Rotamase)	Fkbp10
3Tg and WT		WDR1_MOUSE	WD repeat-containing protein 1 (Actin-interacting protein 1) (AIP1)	Wdr1
3Tg and WT		RL11_MOUSE	60S ribosomal protein L11	Rpl11
3Tg and WT		ARP2_MOUSE	Actin-related protein 2 (Actin-like protein 2)	Actr2
3Tg and WT		COX5B_MOUSE	Cytochrome c oxidase subunit 5B, mitochondrial (Cytochrome c oxidase polypeptide Vb)	Cox5b
3Tg and WT		SPTN1_MOUSE	Spectrin alpha chain, non-erythrocytic 1 (Alpha-II spectrin) (Fodrin alpha chain)	Sptan1
3Tg and WT		RL30_MOUSE	60S ribosomal protein L30	Rpl30
3Tg and WT		CAVN1_MOUSE	Caveolae-associated protein 1 (Cav-p60) (Cavin-1) (Polymerase I and transcript release factor)	Cavin1
3Tg and WT		HNRPU_MOUSE	Heterogeneous nuclear ribonucleoprotein U (hnRNP U) (Scaffold-attachment factor A) (SAF-A)	Hnrnpu
3Tg and WT		AT2A2_MOUSE	Sarcoplasmic/endoplasmic reticulum calcium ATPase 2 (SERCA2) (SR Ca(2+)-ATPase 2) (EC 7.2.2.10) (Calcium pump 2) (Calcium-transporting ATPase sarcoplasmic reticulum type, slow twitch skeletal muscle isoform) (Endoplasmic reticulum class 1/2 Ca(2+) ATPase)	Atp2a2
3Tg and WT		PYR1_MOUSE	CAD protein [Includes: Glutamine-dependent carbamoyl-phosphate synthase (EC 6.3.5.5); Aspartate carbamoyltransferase (EC 2.1.3.2); Dihydroorotase (EC 3.5.2.3)]	Cad
3Tg and WT		ATPO_MOUSE	ATP synthase subunit O, mitochondrial (ATP synthase peripheral stalk subunit OSCP) (Oligomycin sensitivity conferral protein) (OSCP)	Atp5po
3Tg and WT		SODC_MOUSE	Superoxide dismutase [Cu-Zn] (EC 1.15.1.1)	Sod1
3Tg and WT		ATPG_MOUSE	ATP synthase subunit gamma, mitochondrial (ATP synthase F1 subunit gamma) (F-ATPase gamma subunit)	Atp5f1c
3Tg and WT		MRCKA_MOUSE	Serine/threonine-protein kinase MRCK alpha (EC 2.7.11.1) (CDC42-binding protein kinase alpha)	Cdc42bpa
3Tg and WT		ZYX_MOUSE	Zyxin	Zyx
3Tg and WT		PARVA_MOUSE	Alpha-parvin (Actopaxin)	Parva
3Tg and WT		PLEC_MOUSE	Plectin (PCN) (PLTN) (Plectin-1) (Plectin-6)	Plec
3Tg and WT		RS4X_MOUSE	40S ribosomal protein S4, X isoform	Rps4x
3Tg and WT		AIDA_MOUSE	Axin interactor, dorsalization-associated protein (Axin interaction partner and dorsalization antagonist)	Aida
3Tg and WT		DDX3X_MOUSE	ATP-dependent RNA helicase DDX3X (EC 3.6.4.13) (D1Pas1-related sequence 2) (DEAD box RNA helicase DEAD3) (mDEAD3) (DEAD box protein 3, X-chromosomal) (Embryonic RNA helicase)	Ddx3x
3Tg and WT		CD44_MOUSE	CD44 antigen (Extracellular matrix receptor III) (ECMR-III) (GP90 lymphocyte homing/adhesion receptor) (HUTCH-I) (Hermes antigen) (Hyaluronate receptor) (Lymphocyte antigen 24) (Ly-24) (Phagocytic glycoprotein 1) (PGP-1) (Phagocytic glycoprotein I) (PGP-I) (CD antigen CD44)	Cd44
3Tg and WT		RL13A_MOUSE	60S ribosomal protein L13a (Transplantation antigen P198) (Tum-P198 antigen)	Rpl13a
3Tg and WT		CNN1_MOUSE	Calponin-1 (Basic calponin) (Calponin H1, smooth muscle)	Cnn1

3Tg and WT		PDIA1_MOUSE	Protein disulfide-isomerase (PDI) (EC 5.3.4.1) (Cellular thyroid hormone-binding protein) (Endoplasmic reticulum resident protein 59) (ER protein 59) (ERp59) (Prolyl 4-hydroxylase subunit beta) (p55)	P4hb
3Tg and WT		MYH9_MOUSE	Myosin-9 (Cellular myosin heavy chain, type A) (Myosin heavy chain 9) (Myosin heavy chain, non-muscle IIa) (Non-muscle myosin heavy chain A) (NMMHC-A) (Non-muscle myosin heavy chain IIa) (NMMHC II-a) (NMMHC-IIA)	Myh9
3Tg and WT		RBM3_MOUSE	RNA-binding protein 3 (RNA-binding motif protein 3)	Rbm3
3Tg and WT		GNAO_MOUSE	Guanine nucleotide-binding protein G(o) subunit alpha	Gnao1
3Tg and WT		K22O_MOUSE	Keratin, type II cytoskeletal 2 oral (Keratin-76) (K76) (Type-II keratin Kb9)	Krt76
3Tg and WT		1433E_MOUSE	14-3-3 protein epsilon (14-3-3E)	Ywhae
3Tg and WT		PABP1_MOUSE	Polyadenylate-binding protein 1 (PABP-1) (Poly(A)-binding protein 1)	Pabpc1
3Tg and WT		S10AB_MOUSE	Protein S100-A11 (Calgizzarin) (Endothelial monocyte-activating polypeptide) (EMAP) (Protein S100-C) (S100 calcium-binding protein A11)	S100a11
3Tg and WT		EPHA2_MOUSE	Ephrin type-A receptor 2 (EC 2.7.10.1) (Epithelial cell kinase) (Tyrosine-protein kinase receptor ECK) (Tyrosine-protein kinase receptor MPK-5) (Tyrosine-protein kinase receptor SEK-2)	Epha2
3Tg and WT		TMOD3_MOUSE	Tropomodulin-3 (Ubiquitous tropomodulin) (U-Tmod)	Tmod3
3Tg and WT		HNRPM_MOUSE	Heterogeneous nuclear ribonucleoprotein M (hnRNP M)	Hnrnpm
3Tg and WT		NB5R3_MOUSE	NADH-cytochrome b5 reductase 3 (B5R) (Cytochrome b5 reductase) (EC 1.6.2.2) (Diaphorase-1) [Cleaved into: NADH-cytochrome b5 reductase 3 membrane-bound form; NADH-cytochrome b5 reductase 3 soluble form]	Cyb5r3
3Tg and WT		HSP7C_MOUSE	Heat shock cognate 71 kDa protein (Heat shock 70 kDa protein 8)	Hspa8
3Tg and WT		AT2B1_MOUSE	Plasma membrane calcium-transporting ATPase 1 (EC 7.2.2.10) (Plasma membrane calcium ATPase isoform 1) (PMCA1) (Plasma membrane calcium pump isoform 1)	Atp2b1
3Tg and WT		FLOT2_MOUSE	Flotillin-2 (Epidermal surface antigen) (ESA) (Membrane component chromosome 17 surface marker 1 homolog)	Flot2
3Tg and WT		TAGL_MOUSE	Transgelin (Actin-associated protein p27) (Smooth muscle protein 22-alpha) (SM22-alpha)	Tagln
3Tg and WT		ARPC2_MOUSE	Actin-related protein 2/3 complex subunit 2 (Arp2/3 complex 34 kDa subunit) (p34-ARC)	Arpc2
3Tg and WT		TBA1B_MOUSE	Tubulin alpha-1B chain (Alpha-tubulin 2) (Alpha-tubulin isotype M-alpha-2) (Tubulin alpha-2 chain) [Cleaved into: Detyrosinated tubulin alpha-1B chain]	Tuba1b
3Tg and WT		RL10_MOUSE	60S ribosomal protein L10 (Protein QM homolog) (Ribosomal protein L10)	Rpl10
3Tg and WT		PRAF2_MOUSE	PRA1 family protein 2	Praf2
3Tg and WT		RL12_MOUSE	60S ribosomal protein L12	Rpl12
3Tg and WT		HNRH1_MOUSE	Heterogeneous nuclear ribonucleoprotein H (hnRNP H) [Cleaved into: Heterogeneous nuclear ribonucleoprotein H, N-terminally processed]	Hnrnph1

3Tg and WT		HPRT_MOUSE	Hypoxanthine-guanine phosphoribosyltransferase (HGPRT) (HGPRTase) (EC 2.4.2.8) (HPRT B)	Hprt1
3Tg and WT		RAB23_MOUSE	Ras-related protein Rab-23 (Protein open brain) (Rab-15)	Rab23
3Tg and WT		SNTA1_MOUSE	Alpha-1-syntrophin (59 kDa dystrophin-associated protein A1 acidic component 1) (Syntrophin-1)	Snta1
3Tg and WT		SAP_MOUSE	Prosaposin (Sulfated glycoprotein 1) (SGP-1) [Cleaved into: Saposin-A; Saposin-B-Val; Saposin-B; Saposin-C; Saposin-D]	Psap
3Tg and WT		RAB8A_MOUSE	Ras-related protein Rab-8A (Oncogene c-mel)	Rab8a
3Tg and WT		H2B1M_MOUSE	Histone H2B type 1-M (H2B 291B)	H2bc14
3Tg and WT		RAB2A_MOUSE	Ras-related protein Rab-2A	Rab2a
3Tg and WT		NUCB1_MOUSE	Nucleobindin-1 (CALNUC)	Nucb1
3Tg and WT		PDIA6_MOUSE	Protein disulfide-isomerase A6 (EC 5.3.4.1) (Thioredoxin domain-containing protein 7)	Pdia6
3Tg and WT		TS101_MOUSE	Tumor susceptibility gene 101 protein (ESCRT-I complex subunit TSG101)	Tsg101
3Tg and WT		STX12_MOUSE	Syntaxin-12	Stx12
3Tg and WT		ROAA_MOUSE	Heterogeneous nuclear ribonucleoprotein A/B (hnRNP A/B) (CArG-binding factor-A) (CBF-A)	Hnrnpab
3Tg and WT		NHRF1_MOUSE	Na(+)/H(+) exchange regulatory cofactor NHE-RF1 (NHERF-1) (Ezrin-radixin-moesin-binding phosphoprotein 50) (EBP50) (Regulatory cofactor of Na(+)/H(+) exchanger) (Sodium-hydrogen exchanger regulatory factor 1) (Solute carrier family 9 isoform A3 regulatory factor 1)	Slc9a3r1
3Tg and WT		SSRD_MOUSE	Translocon-associated protein subunit delta (TRAP-delta) (Signal sequence receptor subunit delta) (SSR-delta)	Ssr4
3Tg and WT		CALU_MOUSE	Calumenin (Crocalbin)	Calu
3Tg and WT		HSP74_MOUSE	Heat shock 70 kDa protein 4 (Heat shock 70-related protein APG-2)	Hspa4
3Tg and WT		CLCB_MOUSE	Clathrin light chain B (Lcb)	Cltb
3Tg and WT		PKN2_MOUSE	Serine/threonine-protein kinase N2 (EC 2.7.11.13) (PKN gamma) (Protein kinase C-like 2) (Protein-kinase C-related kinase 2)	Pkn2
3Tg and WT		1433F_MOUSE	14-3-3 protein eta	Ywhah
3Tg and WT		RS11_MOUSE	40S ribosomal protein S11	Rps11
3Tg and WT		ITAV_MOUSE	Integrin alpha-V (Vitronectin receptor subunit alpha) (CD antigen CD51) [Cleaved into: Integrin alpha-V heavy chain; Integrin alpha-V light chain]	Itgav
3Tg and WT		RANG_MOUSE	Ran-specific GTPase-activating protein (HpaII tiny fragments locus 9a protein) (Ran-binding protein 1) (RANBP1)	Ranbp1
3Tg and WT		DEST_MOUSE	Destrin (Actin-depolymerizing factor) (ADF) (Sid 23)	Dstn
3Tg and WT		CMC1_MOUSE	Calcium-binding mitochondrial carrier protein Aralar1 (Mitochondrial aspartate glutamate carrier 1) (Solute carrier family 25 member 12)	Slc25a12
3Tg and WT		ITA3_MOUSE	Integrin alpha-3 (CD49 antigen-like family member C) (Galactoprotein B3) (GAPB3) (VLA-3 subunit alpha) (CD antigen CD49c) [Cleaved into: Integrin alpha-3 heavy chain; Integrin alpha-3 light chain]	Itga3
3Tg and WT		GBG12_MOUSE	Guanine nucleotide-binding protein G(I)/G(S)/G(O) subunit gamma-12	Gng12
3Tg and WT		KCC2A_MOUSE	Calcium/calmodulin-dependent protein kinase type II subunit alpha (CaM kinase II subunit alpha) (CaMK-II subunit alpha) (EC 2.7.11.17)	Camk2a

3Tg and WT		TPM4_MOUSE	Tropomyosin alpha-4 chain (Tropomyosin-4)	Tpm4
3Tg and WT		MYOF_MOUSE	Myoferlin (Fer-1-like protein 3)	Myof
3Tg and WT		ETFB_MOUSE	Electron transfer flavoprotein subunit beta (Beta-ETF)	Etfb
3Tg and WT		ACTB_MOUSE	Actin, cytoplasmic 1 (Beta-actin) [Cleaved into: Actin, cytoplasmic 1, N-terminally processed]	Actb
3Tg and WT		IDH3A_MOUSE	Isocitrate dehydrogenase [NAD] subunit alpha, mitochondrial (EC 1.1.1.41) (Isocitric dehydrogenase subunit alpha) (NAD(+)-specific ICDH subunit alpha)	Idh3a
3Tg and WT		ARL8B_MOUSE	ADP-ribosylation factor-like protein 8B (ADP-ribosylation factor-like protein 10C) (Novel small G protein indispensable for equal chromosome segregation 1)	Arl8b
3Tg and WT		PLST_MOUSE	Plastin-3 (T-plastin)	Pls3
3Tg and WT		RS30_MOUSE	40S ribosomal protein S30	Fau
3Tg and WT		DCTN1_MOUSE	Dynactin subunit 1 (150 kDa dynein-associated polypeptide) (DAP-150) (DP-150) (p150-glued)	Dctn1
3Tg and WT		SC23A_MOUSE	Protein transport protein Sec23A (SEC23-related protein A)	Sec23a
3Tg and WT		PCBP1_MOUSE	Poly(rC)-binding protein 1 (Alpha-CP1) (Heterogeneous nuclear ribonucleoprotein E1) (hnRNP E1)	Pcbp1
3Tg and WT		RPN1_MOUSE	Dolichyl-diphosphooligosaccharide--protein glycosyltransferase subunit 1 (Dolichyl-diphosphooligosaccharide--protein glycosyltransferase 67 kDa subunit) (Ribophorin I) (RPN-I) (Ribophorin-1)	Rpn1
3Tg and WT		S10AA_MOUSE	Protein S100-A10 (Calpactin I light chain) (Calpactin-1 light chain) (Cellular ligand of annexin II) (S100 calcium-binding protein A10) (p10 protein) (p11)	S100a10
3Tg and WT		UBE2N_MOUSE	Ubiquitin-conjugating enzyme E2 N (EC 2.3.2.23) (Bendless-like ubiquitin-conjugating enzyme) (E2 ubiquitin-conjugating enzyme N) (Ubc13) (Ubiquitin carrier protein N) (Ubiquitin-protein ligase N)	Ube2n
WT only	312	ACTC_MOUSE	Actin, alpha cardiac muscle 1 (Alpha-cardiac actin) [Cleaved into: Actin, alpha cardiac muscle 1, intermediate form]	Actc1
WT only		H10_MOUSE	Histone H1.0 (Histone H1') (Histone H1(0)) (MyD196) [Cleaved into: Histone H1.0, N-terminally processed]	H1-0
WT only		UBQL2_MOUSE	Ubiquilin-2 (Chap1) (DSK2 homolog) (Protein linking IAP with cytoskeleton 2) (PLIC-2) (Ubiquitin-like product Chap1/Dsk2)	Ubqln2
WT only		TMX2_MOUSE	Thioredoxin-related transmembrane protein 2 (Thioredoxin domain-containing protein 14)	Tmx2
WT only		MAP1B_MOUSE	Microtubule-associated protein 1B (MAP-1B) (MAP1(X)) (MAP1.2) [Cleaved into: MAP1B heavy chain; MAP1 light chain LC1]	Map1b
WT only		AMOT_MOUSE	Angiomotin	Amot
WT only		APMAP_MOUSE	Adipocyte plasma membrane-associated protein (Protein DD16)	Apmap
WT only		H3C_MOUSE	Histone H3.3C (Embryonic)	H3f3c
WT only		RFA1_MOUSE	Replication protein A 70 kDa DNA-binding subunit (RP-A p70) (Replication factor A protein 1) (RF-A protein 1)	Rpa1

WT only		ROA3_MOUSE	Heterogeneous nuclear ribonucleoprotein A3 (hnRNP A3)	Hnrnpa3
WT only		AOXC_MOUSE	Aldehyde oxidase 3 (EC 1.2.3.1) (Aldehyde oxidase homolog 1) (Azaheterocycle hydroxylase 3) (EC 1.17.3.-)	Aox3
WT only		KC1G1_MOUSE	Casein kinase I isoform gamma-1 (CKI-gamma 1) (EC 2.7.11.1)	Csnk1g1
WT only		PRD16_MOUSE	Histone-lysine N-methyltransferase PRDM16 (EC 2.1.1.-) (PR domain zinc finger protein 16) (PR domain-containing protein 16) (Transcription factor MEL1) (MDS1/EVI1-like gene 1)	Prdm16
WT only		PDXD1_MOUSE	Pyridoxal-dependent decarboxylase domain-containing protein 1 (EC 4.1.1.-)	Pdxdc1
WT only		EMD_MOUSE	Emerin	Emd
WT only		BTG3_MOUSE	Protein BTG3 (Abundant in neuroepithelium area protein) (BTG family member 3) (Protein Tob5)	Btg3
WT only		PLOD3_MOUSE	Multifunctional procollagen lysine hydroxylase and glycosyltransferase LH3 [Includes: Procollagen-lysine,2-oxoglutarate 5-dioxygenase 3 (EC 1.14.11.4) (Lysyl hydroxylase 3) (LH3); Procollagen glycosyltransferase (EC 2.4.1.50) (EC 2.4.1.66) (Galactosylhydroxylysine-glucosyltransferase) (Procollagen galactosyltransferase) (Procollagen glucosyltransferase)]	Plod3
WT only		TASOR_MOUSE	Protein TASOR (Transgene activation suppressor protein)	Tasor
WT only		LAMC3_MOUSE	Laminin subunit gamma-3 (Laminin-12 subunit gamma) (Laminin-14 subunit gamma) (Laminin-15 subunit gamma)	Lamc3
WT only		PTN13_MOUSE	Tyrosine-protein phosphatase non-receptor type 13 (EC 3.1.3.48) (PTP36) (Protein tyrosine phosphatase DPZPTP) (Protein tyrosine phosphatase PTP-BL) (Protein-tyrosine phosphatase RIP)	Ptpn13
WT only		UFC1_MOUSE	Ubiquitin-fold modifier-conjugating enzyme 1 (Ufm1-conjugating enzyme 1)	Ufc1
WT only		CKAP5_MOUSE	Cytoskeleton-associated protein 5	Ckap5
WT only		PRS10_MOUSE	26S proteasome regulatory subunit 10B (26S proteasome AAA-ATPase subunit RPT4) (Proteasome 26S subunit ATPase 6) (Proteasome subunit p42)	Psmc6
WT only		KIRR1_MOUSE	Kin of IRRE-like protein 1 (Kin of irregular chiasm-like protein 1) (Nephrin-like protein 1)	Kirrel1
WT only		ARHG2_MOUSE	Rho guanine nucleotide exchange factor 2 (Guanine nucleotide exchange factor H1) (GEF-H1) (LBC'S first cousin) (Lymphoid blast crisis-like 1) (Oncogene LFC) (Rhobin)	Arhgef2
WT only		LDHB_MOUSE	L-lactate dehydrogenase B chain (LDH-B) (EC 1.1.1.27) (LDH heart subunit) (LDH-H)	Ldhb
WT only		STXB6_MOUSE	Syntaxin-binding protein 6	Stxbp6
WT only		CKLF6_MOUSE	CKLF-like MARVEL transmembrane domain-containing protein 6 (Chemokine-like factor superfamily member 6)	Cmtm6

WT only		ODO2_MOUSE	Dihydrolipoyllysine-residue succinyltransferase component of 2-oxoglutarate dehydrogenase complex, mitochondrial (EC 2.3.1.61) (2-oxoglutarate dehydrogenase complex component E2) (OGDC-E2) (Dihydrolipoamide succinyltransferase component of 2-oxoglutarate dehydrogenase complex) (E2K)	Dlst
WT only		HM13_MOUSE	Minor histocompatibility antigen H13 (EC 3.4.23.-) (Presenilin-like protein 3) (Signal peptide peptidase)	Hm13
WT only		DZIP3_MOUSE	E3 ubiquitin-protein ligase DZIP3 (EC 2.3.2.27) (DAZ-interacting protein 3 homolog) (RING-type E3 ubiquitin transferase DZIP3)	Dzip3
WT only		IF5_MOUSE	Eukaryotic translation initiation factor 5 (eIF-5)	Eif5
WT only		U1SBP_MOUSE	U11/U12 small nuclear ribonucleoprotein 35 kDa protein (U11/U12 snRNP 35 kDa protein) (U1 snRNP-binding protein homolog)	Snrnp35
WT only		YJ005_MOUSE	Uncharacterized protein FLJ45252 homolog	YJ005_MOUSE
WT only		XPO1_MOUSE	Exportin-1 (Exp1) (Chromosome region maintenance 1 protein homolog)	Xpo1
WT only		COEA1_MOUSE	Collagen alpha-1(XIV) chain	Col14a1
WT only		MRP_MOUSE	MARCKS-related protein (Brain protein F52) (MARCKS-like protein 1) (Macrophage myristoylated alanine-rich C kinase substrate) (Mac-MARCKS) (MacMARCKS)	Marcksl1
WT only		CEP78_MOUSE	Centrosomal protein of 78 kDa (Cep78)	Cep78
WT only		H2A2C_MOUSE	Histone H2A type 2-C (H2a-613B)	Hist2h2ac
WT only		RABP1_MOUSE	Cellular retinoic acid-binding protein 1 (Cellular retinoic acid-binding protein I) (CRABP-I)	Crabp1
WT only		SPEE_MOUSE	Spermidine synthase (SPDSY) (EC 2.5.1.16) (Putrescine aminopropyltransferase)	Srm
WT only		SET_MOUSE	Protein SET (Phosphatase 2A inhibitor I2PP2A) (I-2PP2A) (Template-activating factor I) (TAF-I)	Set
WT only		CLIC6_MOUSE	Chloride intracellular channel protein 6	Clic6
WT only		TOM6_MOUSE	Mitochondrial import receptor subunit TOM6 homolog (Overexpressed breast tumor protein homolog) (Translocase of outer membrane 6 kDa subunit homolog)	Tomm6
WT only		CTGF_MOUSE	CCN family member 2 (Cellular communication network factor 2) (Connective tissue growth factor) (Hypertrophic chondrocyte-specific protein 24) (Protein FISP-12)	Ccn2
WT only		VIGLN_MOUSE	Vigilin (High density lipoprotein-binding protein) (HDL-binding protein)	Hdlbp
WT only		AT5F1_MOUSE	ATP synthase F(0) complex subunit B1, mitochondrial (ATP synthase peripheral stalk-membrane subunit b) (ATP synthase subunit b) (ATPase subunit b)	Atp5pb
WT only		CBX5_MOUSE	Chromobox protein homolog 5 (Heterochromatin protein 1 homolog alpha) (HP1 alpha)	Cbx5
WT only		P20D1_MOUSE	N-fatty-acyl-amino acid synthase/hydrolase PM20D1 (EC 3.5.1.-) (EC 4.3.-.-) (Peptidase M20 domain-containing protein 1)	Pm20d1
WT only		RBMS2_MOUSE	RNA-binding motif, single-stranded-interacting protein 2	Rbms2
WT only		RL36A_MOUSE	60S ribosomal protein L36a (60S ribosomal protein L44)	Rpl36a
WT only		ATX10_MOUSE	Ataxin-10 (Brain protein E46) (Spinocerebellar ataxia type 10 protein homolog)	Atxn10

WT only		DHRS4_MOUSE	Dehydrogenase/reductase SDR family member 4 (EC 1.1.1.184) (NADPH-dependent carbonyl reductase/NADP-retinol dehydrogenase) (CR) (PHCR) (NADPH-dependent retinol dehydrogenase/reductase) (NDRD) (mouNRDR) (Peroxisomal short-chain alcohol dehydrogenase) (PSCD)	Dhrs4
WT only		RL35_MOUSE	60S ribosomal protein L35	Rpl35
WT only		H2AX_MOUSE	Histone H2AX (H2a/x) (Histone H2A.X)	H2afx
WT only		FINC_MOUSE	Fibronectin (FN) [Cleaved into: Anastellin]	Fn1
WT only		HFM1_MOUSE	Probable ATP-dependent DNA helicase HFM1 (EC 3.6.4.12)	Hfm1
WT only		CAPR1_MOUSE	Caprin-1 (Cytoplasmic activation- and proliferation-associated protein 1) (GPI-anchored membrane protein 1) (GPI-anchored protein p137) (GPI-p137) (p137GPI) (Membrane component chromosome 11 surface marker 1) (RNA granule protein 105)	Caprin1
WT only		FRIL2_MOUSE	Ferritin light chain 2 (Ferritin L subunit 2) (Ferritin subunit LG)	Ftl2
WT only		DDAH2_MOUSE	N(G),N(G)-dimethylarginine dimethylaminohydrolase 2 (DDAH-2) (Dimethylarginine dimethylaminohydrolase 2) (EC 3.5.3.18) (DDAHII) (Dimethylargininase-2)	Ddah2
WT only		SH3G2_MOUSE	Endophilin-A1 (Endophilin-1) (SH3 domain protein 2A) (SH3 domain-containing GRB2-like protein 2) (SH3p4)	Sh3gl2
WT only		C10_MOUSE	Protein C10	Grcc10
WT only		IPO4_MOUSE	Importin-4 (Imp4) (Importin-4a) (Imp4a) (Ran-binding protein 4) (RanBP4)	Ipo4
WT only		DCTN2_MOUSE	Dynactin subunit 2 (50 kDa dynein-associated polypeptide) (Dynactin complex 50 kDa subunit) (DCTN-50) (Growth cone membrane protein 23-48K) (GMP23-48K) (p50 dynamitin)	Dctn2
WT only		IF2B_MOUSE	Eukaryotic translation initiation factor 2 subunit 2 (Eukaryotic translation initiation factor 2 subunit beta) (eIF-2-beta)	Eif2s2
WT only		EIF3B_MOUSE	Eukaryotic translation initiation factor 3 subunit B (eIF3b) (Eukaryotic translation initiation factor 3 subunit 9) (eIF-3-eta) (eIF3 p116)	Eif3b
WT only		ODPB_MOUSE	Pyruvate dehydrogenase E1 component subunit beta, mitochondrial (PDHE1-B) (EC 1.2.4.1)	Pdhb
WT only		CMC2_MOUSE	Calcium-binding mitochondrial carrier protein Aralar2 (Citrin) (Mitochondrial aspartate glutamate carrier 2) (Solute carrier family 25 member 13)	Slc25a13
WT only		PHAR4_MOUSE	Phosphatase and actin regulator 4 (Protein Humpty dumpty) (Humdy)	Phactr4
WT only		CYB5_MOUSE	Cytochrome b5	Cyb5a
WT only		MCM2_MOUSE	DNA replication licensing factor MCM2 (EC 3.6.4.12) (Minichromosome maintenance protein 2 homolog) (Nuclear protein BM28)	Mcm2
WT only		COTL1_MOUSE	Coactosin-like protein	Cotl1
WT only		ZN706_MOUSE	Zinc finger protein 706	Znf706
WT only		SARNP_MOUSE	SAP domain-containing ribonucleoprotein (Nuclear protein Hcc-1)	Sarnp
WT only		PTH2_MOUSE	Peptidyl-tRNA hydrolase 2, mitochondrial (PTH 2) (EC 3.1.1.29)	Ptrh2
WT only		COR2A_MOUSE	Coronin-2A	Coro2a

WT only		AATC_MOUSE	Aspartate aminotransferase, cytoplasmic (cAspAT) (EC 2.6.1.1) (EC 2.6.1.3) (Cysteine aminotransferase, cytoplasmic) (Cysteine transaminase, cytoplasmic) (cCAT) (Glutamate oxaloacetate transaminase 1) (Transaminase A)	Got1
WT only		ANO6_MOUSE	Anoctamin-6 (Small-conductance calcium-activated nonselective cation channel) (SCAN channel) (Transmembrane protein 16F)	Ano6
WT only		NCLN_MOUSE	Nicalin (Nicastrin-like protein)	Ncln
WT only		ARFG2_MOUSE	ADP-ribosylation factor GTPase-activating protein 2 (ARF GAP 2) (GTPase-activating protein ZNF289) (Zinc finger protein 289)	Arfgap2
WT only		HMGCL_MOUSE	Hydroxymethylglutaryl-CoA lyase, mitochondrial (HL) (HMG-CoA lyase) (EC 4.1.3.4) (3-hydroxy-3-methylglutarate-CoA lyase)	Hmgcl
WT only		GSH0_MOUSE	Glutamate--cysteine ligase regulatory subunit (GCS light chain) (Gamma-ECS regulatory subunit) (Gamma-glutamylcysteine synthetase regulatory subunit) (Glutamate--cysteine ligase modifier subunit)	Gclm
WT only		RSU1_MOUSE	Ras suppressor protein 1 (RSP-1) (Rsu-1)	Rsu1
WT only		HSPB6_MOUSE	Heat shock protein beta-6 (HspB6)	Hspb6
WT only		CELF2_MOUSE	CUGBP Elav-like family member 2 (CELF-2) (Bruno-like protein 3) (CUG triplet repeat RNA-binding protein 2) (CUG-BP2) (CUG-BP- and ETR-3-like factor 2) (ELAV-type RNA-binding protein 3) (ETR-3) (mETR-3) (Neuroblastoma apoptosis-related RNA-binding protein) (mNapor) (RNA-binding protein BRUNOL-3)	Celf2
WT only		GLRX3_MOUSE	Glutaredoxin-3 (PKC-interacting cousin of thioredoxin) (PICOT) (PKC-theta-interacting protein) (PKCq-interacting protein) (Thioredoxin-like protein 2)	Glrx3
WT only		CLPP_MOUSE	ATP-dependent Clp protease proteolytic subunit, mitochondrial (EC 3.4.21.92) (Endopeptidase Clp)	Clpp
WT only		K2C75_MOUSE	Keratin, type II cytoskeletal 75 (Cytokeratin-75) (CK-75) (Keratin-6 hair follicle) (mK6hf) (Keratin-75) (K75) (Type II keratin-K6hf) (Type-II keratin Kb18)	Krt75
WT only		SFXN3_MOUSE	Sideroflexin-3	Sfxn3
WT only		PK3C3_MOUSE	Phosphatidylinositol 3-kinase catalytic subunit type 3 (PI3-kinase type 3) (PI3K type 3) (PtdIns-3-kinase type 3) (EC 2.7.1.137) (Phosphoinositide-3-kinase class 3)	Pik3c3
WT only		CC178_MOUSE	Coiled-coil domain-containing protein 178	Ccdc178
WT only		FKBP2_MOUSE	Peptidyl-prolyl cis-trans isomerase FKBP2 (PPIase FKBP2) (EC 5.2.1.8) (13 kDa FK506-binding protein) (13 kDa FKBP) (FKBP-13) (FK506-binding protein 2) (FKBP-2) (Immunophilin FKBP13) (Rotamase)	Fkbp2
WT only		ESYT2_MOUSE	Extended synaptotagmin-2 (E-Syt2)	Esyt2
WT only		RBBP7_MOUSE	Histone-binding protein RBBP7 (Histone acetyltransferase type B subunit 2) (Nucleosome-remodeling factor subunit RBAP46) (Retinoblastoma-binding protein 7) (RBBP-7) (Retinoblastoma-binding protein p46)	Rbbp7
WT only		RAI14_MOUSE	Ankycorbin (Ankyrin repeat and coiled-coil structure-containing protein) (Novel retinal pigment epithelial cell protein) (Retinoic acid-induced protein 14) (p125)	Rai14

WT only		H2AJ_MOUSE	Histone H2A.J (H2a/j)	H2afj
WT only		H2B1B_MOUSE	Histone H2B type 1-B (h2B-143)	Hist1h2bb
WT only		CXAR_MOUSE	Coxsackievirus and adenovirus receptor homolog (CAR) (mCAR)	Cxadr
WT only		PSMD2_MOUSE	26S proteasome non-ATPase regulatory subunit 2 (26S proteasome regulatory subunit RPN1) (26S proteasome regulatory subunit S2) (26S proteasome subunit p97)	Psmd2
WT only		RL38_MOUSE	60S ribosomal protein L38	Rpl38
WT only		ACY1_MOUSE	Aminoacylase-1 (ACY-1) (EC 3.5.1.14) (N-acyl-L-amino-acid amidohydrolase)	Acy1
WT only		ELOB_MOUSE	Elongin-B (EloB) (Elongin 18 kDa subunit) (RNA polymerase II transcription factor SIII subunit B) (SIII p18) (Transcription elongation factor B polypeptide 2)	Elob
WT only		SGTA_MOUSE	Small glutamine-rich tetratricopeptide repeat-containing protein alpha (Alpha-SGT)	Sgta
WT only		ERLN2_MOUSE	Erlin-2 (Endoplasmic reticulum lipid raft-associated protein 2) (Stomatin-prohibitin-flotillin-HflC/K domain-containing protein 2) (SPFH domain-containing protein 2)	Erlin2
WT only		AMPB_MOUSE	Aminopeptidase B (AP-B) (EC 3.4.11.6) (Arginine aminopeptidase) (Arginyl aminopeptidase) (Cytosol aminopeptidase IV)	Rnpep
WT only		G6PI_MOUSE	Glucose-6-phosphate isomerase (GPI) (EC 5.3.1.9) (Autocrine motility factor) (AMF) (Neuroleukin) (NLK) (Phosphoglucose isomerase) (PGI) (Phosphohexose isomerase) (PHI)	Gpi
WT only		CHD5_MOUSE	Chromodomain-helicase-DNA-binding protein 5 (CHD-5) (EC 3.6.4.12) (ATP-dependent helicase CHD5)	Chd5
WT only		SF01_MOUSE	Splicing factor 1 (CW17) (Mammalian branch point-binding protein) (BBP) (mBBP) (Transcription factor ZFM1) (mZFM) (Zinc finger gene in MEN1 locus) (Zinc finger protein 162)	Sf1
WT only		IMDH2_MOUSE	Inosine-5'-monophosphate dehydrogenase 2 (IMP dehydrogenase 2) (IMPD 2) (IMPDH 2) (EC 1.1.1.205) (IMPDH-II)	Impdh2
WT only		SNX12_MOUSE	Sorting nexin-12 (SDP8 protein)	Snx12
WT only		ADA24_MOUSE	Disintegrin and metalloproteinase domain-containing protein 24 (ADAM 24) (EC 3.4.24.-) (Testase-1)	Adam24
WT only		TANC2_MOUSE	Protein TANC2 (Tetratricopeptide repeat, ankyrin repeat and coiled-coil domain-containing protein 2)	Tanc2
WT only		GLSK_MOUSE	Glutaminase kidney isoform, mitochondrial (GLS) (EC 3.5.1.2) [Cleaved into: Glutaminase kidney isoform, mitochondrial 68 kDa chain; Glutaminase kidney isoform, mitochondrial 65 kDa chain]	Gls
WT only		RAB8B_MOUSE	Ras-related protein Rab-8B	Rab8b
WT only		SYFA_MOUSE	Phenylalanine--tRNA ligase alpha subunit (EC 6.1.1.20) (Phenylalanyl-tRNA synthetase alpha subunit) (PheRS)	Farsa
WT only		LPPRC_MOUSE	Leucine-rich PPR motif-containing protein, mitochondrial (130 kDa leucine-rich protein) (LRP 130) (mLRP130)	Lrpprc

WT only		TMX3_MOUSE	Protein disulfide-isomerase TMX3 (EC 5.3.4.1) (Thioredoxin domain-containing protein 10) (Thioredoxin-related transmembrane protein 3)	Tmx3
WT only		RBBP4_MOUSE	Histone-binding protein RBBP4 (Chromatin assembly factor 1 subunit C) (CAF-1 subunit C) (Chromatin assembly factor I p48 subunit) (CAF-I 48 kDa subunit) (CAF-I p48) (Nucleosome-remodeling factor subunit RBAP48) (Retinoblastoma-binding protein 4) (RBBP-4) (Retinoblastoma-binding protein p48)	Rbbp4
WT only		BAX_MOUSE	Apoptosis regulator BAX	Bax
WT only		SSBP_MOUSE	Single-stranded DNA-binding protein, mitochondrial (Mt-SSB) (MtSSB)	Ssbp1
WT only		IPO7_MOUSE	Importin-7 (Imp7) (Ran-binding protein 7) (RanBP7)	Ipo7
WT only		KAP0_MOUSE	cAMP-dependent protein kinase type I-alpha regulatory subunit [Cleaved into: cAMP-dependent protein kinase type I-alpha regulatory subunit, N-terminally processed]	Prkar1a
WT only		UBAP2_MOUSE	Ubiquitin-associated protein 2 (UBAP-2) (Protein lingerer homolog 1) (mLig-1)	Ubap2
WT only		OCC1_MOUSE	Overexpressed in colon carcinoma 1 protein homolog (OCC-1)	OCC1_MOUSE
WT only		PTBP2_MOUSE	Polypyrimidine tract-binding protein 2 (Brain-enriched polypyrimidine tract-binding protein) (Brain-enriched PTB) (Neural polypyrimidine tract-binding protein) (RRM-type RNA-binding protein brPTB)	Ptbp2
WT only		VPS4A_MOUSE	Vacuolar protein sorting-associated protein 4A (EC 3.6.4.6)	Vps4a
WT only		DEK_MOUSE	Protein DEK	Dek
WT only		KS6B2_MOUSE	Ribosomal protein S6 kinase beta-2 (S6K-beta-2) (S6K2) (EC 2.7.11.1) (70 kDa ribosomal protein S6 kinase 2) (p70 ribosomal S6 kinase beta) (p70 S6 kinase beta) (p70 S6K-beta) (p70 S6KB)	Rps6kb2
WT only		TPPC4_MOUSE	Trafficking protein particle complex subunit 4 (Synbindin) (TRS23 homolog)	Trappc4
WT only		AT5G1_MOUSE	ATP synthase F(0) complex subunit C1, mitochondrial (ATP synthase lipid-binding protein) (ATP synthase membrane subunit c locus 1) (ATP synthase proteolipid P1) (ATPase protein 9) (ATPase subunit c)	Atp5mc1
WT only		BAIP2_MOUSE	Brain-specific angiogenesis inhibitor 1-associated protein 2 (BAI-associated protein 2) (BAI1-associated protein 2) (Insulin receptor substrate protein of 53 kDa) (IRSp53) (Insulin receptor substrate p53) (Insulin receptor tyrosine kinase 53 kDa substrate)	Baiap2
WT only		CREL2_MOUSE	Protein disulfide isomerase Creld2 (EC 5.3.4.1) (Cysteine-rich with EGF-like domain protein 2)	Creld2
WT only		RL40_MOUSE	Ubiquitin-60S ribosomal protein L40 (Ubiquitin A-52 residue ribosomal protein fusion product 1) [Cleaved into: Ubiquitin; 60S ribosomal protein L40 (CEP52)]	Uba52
WT only		ECI1_MOUSE	Enoyl-CoA delta isomerase 1, mitochondrial (EC 5.3.3.8) (3,2-trans-enoyl-CoA isomerase) (Delta(3),Delta(2)-enoyl-CoA isomerase) (D3,D2-enoyl-CoA isomerase) (Dodecenoyl-CoA isomerase)	Eci1

WT only		MTAP_MOUSE	S-methyl-5'-thioadenosine phosphorylase (EC 2.4.2.28) (5'-methylthioadenosine phosphorylase) (MTA phosphorylase) (MTAP) (MTAPase)	Mtap
WT only		SIAS_MOUSE	Sialic acid synthase (N-acetylneuraminate-9-phosphate synthase) (EC 2.5.1.57) (N-acetylneuraminic acid phosphate synthase)	Nans
WT only		SRSF2_MOUSE	Serine/arginine-rich splicing factor 2 (Protein PR264) (Putative myelin regulatory factor 1) (MRF-1) (Splicing component, 35 kDa) (Splicing factor SC35) (SC-35) (Splicing factor, arginine/serine-rich 2)	Srsf2
WT only		DOCK7_MOUSE	Dedicator of cytokinesis protein 7 (Protein moonlight)	Dock7
WT only		FUS_MOUSE	RNA-binding protein FUS (Protein pigpen)	Fus
WT only		MFGM_MOUSE	Lactadherin (MFGM) (Milk fat globule-EGF factor 8) (MFG-E8) (SED1) (Sperm surface protein SP47) (MP47)	Mfge8
WT only		PPCE_MOUSE	Prolyl endopeptidase (PE) (EC 3.4.21.26) (Post-proline cleaving enzyme)	Prep
WT only		ARL8A_MOUSE	ADP-ribosylation factor-like protein 8A (ADP-ribosylation factor-like protein 10B) (Novel small G protein indispensable for equal chromosome segregation 2)	Arl8a
WT only		SYIC_MOUSE	Isoleucine--tRNA ligase, cytoplasmic (EC 6.1.1.5) (Isoleucyl-tRNA synthetase) (IRS) (IleRS)	Iars
WT only		NUDT9_MOUSE	ADP-ribose pyrophosphatase, mitochondrial (EC 3.6.1.13) (ADP-ribose diphosphatase) (ADP-ribose phosphohydrolase) (Adenosine diphosphoribose pyrophosphatase) (ADPR-PPase) (Nucleoside diphosphate-linked moiety X motif 9) (Nudix motif 9)	Nudt9
WT only		BAG2_MOUSE	BAG family molecular chaperone regulator 2 (BAG-2) (Bcl-2-associated athanogene 2)	Bag2
WT only		AFAD_MOUSE	Afadin (Afadin adherens junction formation factor) (Protein Af-6)	Afdn
WT only		EFTU_MOUSE	Elongation factor Tu, mitochondrial	Tufm
WT only		PSB4_MOUSE	Proteasome subunit beta type-4 (EC 3.4.25.1) (Low molecular mass protein 3) (Macropain beta chain) (Multicatalytic endopeptidase complex beta chain) (Proteasome beta chain) (Proteasome chain 3)	Psmb4
WT only		PAWR_MOUSE	PRKC apoptosis WT1 regulator protein (Prostate apoptosis response 4 protein) (Par-4)	Pawr
WT only		S12A9_MOUSE	Solute carrier family 12 member 9 (Cation-chloride cotransporter-interacting protein 1) (Potassium-chloride transporter 9)	Slc12a9
WT only		SRSF3_MOUSE	Serine/arginine-rich splicing factor 3 (Pre-mRNA-splicing factor SRP20) (Protein X16) (Splicing factor, arginine/serine-rich 3)	Srsf3
WT only		TBB2B_MOUSE	Tubulin beta-2B chain	Tubb2b
WT only		LOX12_MOUSE	Arachidonate 12-lipoxygenase, 12S-type (12S-LOX) (12S-lipoxygenase) (EC 1.13.11.31) (Arachidonate 15-lipoxygenase,15S-type) (EC 1.13.11.33) (Linoleate 13S-lipoxygenase) (EC 1.13.11.12) (Lipoxin synthase 12-LO) (EC 3.3.2.-) (Platelet-type lipoxygenase 12) (P-12LO)	Alox12
WT only		KIF23_MOUSE	Kinesin-like protein KIF23	Kif23

WT only		FMR1_MOUSE	Synaptic functional regulator FMR1 (Fragile X mental retardation protein 1 homolog) (FMRP) (Protein FMR-1) (mFmr1p)	Fmr1
WT only		PACN2_MOUSE	Protein kinase C and casein kinase substrate in neurons protein 2 (Syndapin-2) (Syndapin-II) (SdpII)	Pacsin2
WT only		DX39A_MOUSE	ATP-dependent RNA helicase DDX39A (EC 3.6.4.13) (DEAD box protein 39)	Ddx39a
WT only		PSA4_MOUSE	Proteasome subunit alpha type-4 (EC 3.4.25.1) (Macropain subunit C9) (Multicatalytic endopeptidase complex subunit C9) (Proteasome component C9) (Proteasome subunit L)	Psma4
WT only		KIF5A_MOUSE	Kinesin heavy chain isoform 5A (Kinesin heavy chain neuron-specific 1) (Neuronal kinesin heavy chain) (NKHC)	Kif5a
WT only		NDUAD_MOUSE	NADH dehydrogenase [ubiquinone] 1 alpha subcomplex subunit 13 (Cell death regulatory protein GRIM-19) (Complex I-B16.6) (CI-B16.6) (Gene associated with retinoic and interferon-induced mortality 19 protein) (GRIM-19) (Gene associated with retinoic and IFN-induced mortality 19 protein) (NADH-ubiquinone oxidoreductase B16.6 subunit)	Ndufa13
WT only		ACTBL_MOUSE	Beta-actin-like protein 2 (Kappa-actin)	Actbl2
WT only		PBDC1_MOUSE	Protein PBDC1 (Polysaccharide biosynthesis domain-containing protein 1)	Pbdc1
WT only		ATIF1_MOUSE	ATPase inhibitor, mitochondrial (ATP synthase F1 subunit epsilon) (Inhibitor of F(1)F(o)-ATPase) (IF(1)) (IF1)	ATP5IF1
WT only		SYVC_MOUSE	Valine--tRNA ligase (EC 6.1.1.9) (Protein G7a) (Valyl-tRNA synthetase) (ValRS)	Vars
WT only		ARF5_MOUSE	ADP-ribosylation factor 5	Arf5
WT only		ATAD1_MOUSE	ATPase family AAA domain-containing protein 1 (EC 3.6.1.3) (Thorase)	Atad1
WT only		CDK12_MOUSE	Cyclin-dependent kinase 12 (EC 2.7.11.22) (EC 2.7.11.23) (Cdc2-related kinase, arginine/serine-rich) (CrkRS) (Cell division cycle 2-related protein kinase 7) (CDC2-related protein kinase 7) (Cell division protein kinase 12)	Cdk12
WT only		EP15R_MOUSE	Epidermal growth factor receptor substrate 15-like 1 (Epidermal growth factor receptor pathway substrate 15-related sequence) (Eps15-rs) (Eps15-related protein) (Eps15R)	Eps15l1
WT only		RFA3_MOUSE	Replication protein A 14 kDa subunit (RP-A p14) (Replication factor A protein 3) (RF-A protein 3)	Rpa3
WT only		HA11_MOUSE	H-2 class I histocompatibility antigen, D-B alpha chain (H-2D(B))	H2-D1
WT only		SGSM3_MOUSE	Small G protein signaling modulator 3 (RUN and TBC1 domain-containing protein 3)	Sgsm3
WT only		NU155_MOUSE	Nuclear pore complex protein Nup155 (155 kDa nucleoporin) (Nucleoporin Nup155)	Nup155
WT only		YBOX1_MOUSE	Y-box-binding protein 1 (YB-1) (CCAAT-binding transcription factor I subunit A) (CBF-A) (DNA-binding protein B) (DBPB) (Enhancer factor I subunit A) (EFI-A) (Nuclease-sensitive element-binding protein 1) (Y-box transcription factor)	Ybx1
WT only		ITIH3_MOUSE	Inter-alpha-trypsin inhibitor heavy chain H3 (ITI heavy chain H3) (ITI-HC3) (Inter-alpha-inhibitor heavy chain 3)	Itih3

WT only		ES1_MOUSE	Glutamine amidotransferase-like class 1 domain-containing protein 3A, mitochondrial	Gatd3a
WT only		TOM34_MOUSE	Mitochondrial import receptor subunit TOM34 (Translocase of outer membrane 34 kDa subunit)	Tomm34
WT only		REPS1_MOUSE	RalBP1-associated Eps domain-containing protein 1 (RalBP1-interacting protein 1)	Reps1
WT only		H2A2B_MOUSE	Histone H2A type 2-B (H2a-613A)	Hist2h2ab
WT only		GNS_MOUSE	N-acetylglucosamine-6-sulfatase (EC 3.1.6.14) (Glucosamine-6-sulfatase) (G6S)	Gns
WT only		DHX9_MOUSE	ATP-dependent RNA helicase A (EC 3.6.4.13) (DEAH box protein 9) (mHEL-5) (Nuclear DNA helicase II) (NDH II) (RNA helicase A) (RHA)	Dhx9
WT only		K2C8_MOUSE	Keratin, type II cytoskeletal 8 (Cytokeratin endo A) (Cytokeratin-8) (CK-8) (Keratin-8) (K8) (Type-II keratin Kb8)	Krt8
WT only		EPHA7_MOUSE	Ephrin type-A receptor 7 (EC 2.7.10.1) (Developmental kinase 1) (mDK-1) (EPH homology kinase 3) (EHK-3) (Embryonic brain kinase) (EBK)	Epha7
WT only		DTNA_MOUSE	Dystrobrevin alpha (DTN-A) (Alpha-dystrobrevin)	Dtna
WT only		SEM5B_MOUSE	Semaphorin-5B (Semaphorin-G) (Sema G)	Sema5b
WT only		PLRKT_MOUSE	Plasminogen receptor (KT) (Plg-R(KT))	Plgrkt
WT only		PFD3_MOUSE	Prefoldin subunit 3 (von Hippel-Lindau-binding protein 1) (VBP-1) (VHL-binding protein 1)	Vbp1
WT only		PRDX3_MOUSE	Thioredoxin-dependent peroxide reductase, mitochondrial (EC 1.11.1.15) (Antioxidant protein 1) (AOP-1) (PRX III) (Perioredoxin-3) (Protein MER5)	Prdx3
WT only		TPP2_MOUSE	Tripeptidyl-peptidase 2 (TPP-2) (EC 3.4.14.10) (Tripeptidyl aminopeptidase) (Tripeptidyl-peptidase II) (TPP-II)	Tpp2
WT only		FA49A_MOUSE	Protein FAM49A	Fam49a
WT only		CUTA_MOUSE	Protein CutA (Brain acetylcholinesterase putative membrane anchor)	Cuta
WT only		IL1AP_MOUSE	Interleukin-1 receptor accessory protein (IL-1 receptor accessory protein) (IL-1RAcP) (Interleukin-33 receptot beta chain)	Il1rap
WT only		LMF1_MOUSE	Lipase maturation factor 1 (Transmembrane protein 112)	Lmf1
WT only		PALM2_MOUSE	Paralemmin-2	Palm2
WT only		H2A3_MOUSE	Histone H2A type 3	Hist3h2a
WT only		VIPR2_MOUSE	Vasoactive intestinal polypeptide receptor 2 (VIP-R-2) (Pituitary adenylate cyclase-activating polypeptide type III receptor) (PACAP type III receptor) (PACAP-R-3) (PACAP-R3)	Vipr2
WT only		K2C7_MOUSE	Keratin, type II cytoskeletal 7 (Cytokeratin-7) (CK-7) (Keratin-7) (K7) (Type-II keratin Kb7)	Krt7
WT only		LIMA1_MOUSE	LIM domain and actin-binding protein 1 (Epithelial protein lost in neoplasm) (mEPLIN)	Lima1
WT only		EIF3G_MOUSE	Eukaryotic translation initiation factor 3 subunit G (eIF3g) (Eukaryotic translation initiation factor 3 RNA-binding subunit) (eIF-3 RNA-binding subunit) (Eukaryotic translation initiation factor 3 subunit 4) (eIF-3-delta) (eIF3 p42) (eIF3 p44)	Eif3g
WT only		XPP1_MOUSE	Xaa-Pro aminopeptidase 1 (EC 3.4.11.9) (Aminoacylproline aminopeptidase) (Cytosolic aminopeptidase P) (Soluble aminopeptidase P) (sAmp) (X-Pro aminopeptidase 1) (X-prolyl aminopeptidase 1, soluble)	Xpnpep1

WT only		DRG2_MOUSE	Developmentally-regulated GTP-binding protein 2 (DRG-2) (Translation factor GTPase DRG2) (TRAFAC GTPase DRG2) (EC 3.6.5.-)	Drg2
WT only		PPIG_MOUSE	Peptidyl-prolyl cis-trans isomerase G (PPIase G) (Peptidyl-prolyl isomerase G) (EC 5.2.1.8) (Cyclophilin G) (Rotamase G)	Ppig
WT only		CNOT1_MOUSE	CCR4-NOT transcription complex subunit 1 (CCR4-associated factor 1)	Cnot1
WT only		DDX21_MOUSE	Nucleolar RNA helicase 2 (EC 3.6.4.13) (DEAD box protein 21) (Gu-alpha) (Nucleolar RNA helicase Gu) (Nucleolar RNA helicase II) (RH II/Gu)	Ddx21
WT only		SDC2_MOUSE	Syndecan-2 (SYND2) (Fibroglycan) (Heparan sulfate proteoglycan core protein) (HSPG) (CD antigen CD362)	Sdc2
WT only		SFPQ_MOUSE	Splicing factor, proline- and glutamine-rich (DNA-binding p52/p100 complex, 100 kDa subunit) (Polypyrimidine tract-binding protein-associated-splicing factor) (PSF) (PTB-associated-splicing factor)	Sfpq
WT only		KIF1B_MOUSE	Kinesin-like protein KIF1B	Kif1b
WT only		NDUS3_MOUSE	NADH dehydrogenase [ubiquinone] iron-sulfur protein 3, mitochondrial (EC 1.6.99.3) (EC 7.1.1.2) (Complex I-30kD) (CI-30kD) (NADH-ubiquinone oxidoreductase 30 kDa subunit)	Ndufs3
WT only		ACOC_MOUSE	Cytoplasmic aconitate hydratase (Aconitase) (EC 4.2.1.3) (Citrate hydro-lyase) (Iron regulatory protein 1) (IRP1) (Iron-responsive element-binding protein 1) (IRE-BP 1)	Aco1
WT only		NH2L1_MOUSE	NHP2-like protein 1 (Fertilization antigen 1) (FA-1) (High mobility group-like nuclear protein 2 homolog 1) (Sperm-specific antigen 1) (U4/U6.U5 small nuclear ribonucleoprotein SNU13) (U4/U6.U5 tri-snRNP 15.5 kDa protein) [Cleaved into: NHP2-like protein 1, N-terminally processed]	Snu13
WT only		CO5A2_MOUSE	Collagen alpha-2(V) chain	Col5a2
WT only		PRS6B_MOUSE	26S proteasome regulatory subunit 6B (26S proteasome AAA-ATPase subunit RPT3) (CIP21) (MB67-interacting protein) (MIP224) (Proteasome 26S subunit ATPase 4) (Tat-binding protein 7) (TBP-7)	Psmc4
WT only		CDK16_MOUSE	Cyclin-dependent kinase 16 (EC 2.7.11.22) (CRK5) (Cell division protein kinase 16) (PCTAIRE-motif protein kinase 1) (Serine/threonine-protein kinase PCTAIRE-1)	Cdk16
WT only		OAT_MOUSE	Ornithine aminotransferase, mitochondrial (EC 2.6.1.13) (Ornithine--oxo-acid aminotransferase)	Oat
WT only		SRSF1_MOUSE	Serine/arginine-rich splicing factor 1 (ASF/SF2) (Pre-mRNA-splicing factor SRp30a) (Splicing factor, arginine/serine-rich 1)	Srsf1
WT only		H2B1P_MOUSE	Histone H2B type 1-P	Hist1h2bp
WT only		CY1_MOUSE	Cytochrome c1, heme protein, mitochondrial (Complex III subunit 4) (Complex III subunit IV) (Cytochrome b-c1 complex subunit 4) (Ubiquinol-cytochrome-c reductase complex cytochrome c1 subunit) (Cytochrome c-1)	Cyc1
WT only		PEA15_MOUSE	Astrocytic phosphoprotein PEA-15 (15 kDa phosphoprotein enriched in astrocytes)	Pea15

WT only		TR112_MOUSE	Multifunctional methyltransferase subunit TRM112-like protein (tRNA methyltransferase 112 homolog)	Trmt112
WT only		2A5E_MOUSE	Serine/threonine-protein phosphatase 2A 56 kDa regulatory subunit epsilon isoform (PP2A B subunit isoform B'-epsilon) (PP2A B subunit isoform B56-epsilon) (PP2A B subunit isoform PR61-epsilon) (PP2A B subunit isoform R5-epsilon)	Ppp2r5e
WT only		C42S2_MOUSE	CDC42 small effector protein 2	Cdc42se2
WT only		HMGB2_MOUSE	High mobility group protein B2 (High mobility group protein 2) (HMG-2)	Hmgb2
WT only		UBP5_MOUSE	Ubiquitin carboxyl-terminal hydrolase 5 (EC 3.4.19.12) (Deubiquitinating enzyme 5) (Isopeptidase T) (Ubiquitin thioesterase 5) (Ubiquitin-specific-processing protease 5)	Usp5
WT only		RBMS3_MOUSE	RNA-binding motif, single-stranded-interacting protein 3	Rbms3
WT only		THIKA_MOUSE	3-ketoacyl-CoA thiolase A, peroxisomal (EC 2.3.1.16) (Acetyl-CoA acyltransferase A) (Beta-ketothiolase A) (Peroxisomal 3-oxoacyl-CoA thiolase A)	Acaa1a
WT only		MCA3_MOUSE	Eukaryotic translation elongation factor 1 epsilon-1 (Elongation factor p18) (Multisynthase complex auxiliary component p18)	Eef1e1
WT only		CTNA2_MOUSE	Catenin alpha-2 (Alpha N-catenin)	Ctnna2
WT only		XYLB_MOUSE	Xylulose kinase (Xylulokinase) (EC 2.7.1.17)	Xylb
WT only		F120A_MOUSE	Constitutive coactivator of PPAR-gamma-like protein 1 (Oxidative stress-associated Src activator) (Protein FAM120A)	FAM120A
WT only		G6PD1_MOUSE	Glucose-6-phosphate 1-dehydrogenase X (G6PD) (EC 1.1.1.49)	G6pdx
WT only		PHB2_MOUSE	Prohibitin-2 (B-cell receptor-associated protein BAP37) (Repressor of estrogen receptor activity)	Phb2
WT only		STX2_MOUSE	Syntaxin-2 (Epimorphin)	Stx2
WT only		SC22B_MOUSE	Vesicle-trafficking protein SEC22b (ER-Golgi SNARE of 24 kDa) (ERS-24) (ERS24) (SEC22 vesicle-trafficking protein homolog B) (SEC22 vesicle-trafficking protein-like 1) (mSec22b)	Sec22b
WT only		PSMD6_MOUSE	26S proteasome non-ATPase regulatory subunit 6 (26S proteasome regulatory subunit RPN7) (26S proteasome regulatory subunit S10) (p42A)	Psmd6
WT only		PSA6_MOUSE	Proteasome subunit alpha type-6 (EC 3.4.25.1) (Macropain iota chain) (Multicatalytic endopeptidase complex iota chain) (Proteasome iota chain)	Psma6
WT only		KRT85_MOUSE	Keratin, type II cuticular Hb5 (Keratin-85) (K85) (Type II hair keratin Hb5) (Type-II keratin Kb25)	Krt85
WT only		STX4_MOUSE	Syntaxin-4	Stx4
WT only		ERF1_MOUSE	Eukaryotic peptide chain release factor subunit 1 (Eukaryotic release factor 1) (eRF1)	Etf1
WT only		PURA2_MOUSE	Adenylosuccinate synthetase isozyme 2 (AMPSase 2) (AdSS 2) (EC 6.3.4.4) (Adenylosuccinate synthetase, acidic isozyme) (Adenylosuccinate synthetase, liver isozyme) (L-type adenylosuccinate synthetase) (IMP--aspartate ligase 2)	Adss2

WT only		IDHC_MOUSE	Isocitrate dehydrogenase [NADP] cytoplasmic (IDH) (EC 1.1.1.42) (Cytosolic NADP-isocitrate dehydrogenase) (IDP) (NADP(+)-specific ICDH) (Oxalosuccinate decarboxylase)	Idh1
WT only		RUVB1_MOUSE	RuvB-like 1 (EC 3.6.4.12) (49 kDa TATA box-binding protein-interacting protein) (49 kDa TBP-interacting protein) (DNA helicase p50) (Pontin 52) (TIP49a)	Ruvbl1
WT only		ANXA8_MOUSE	Annexin A8 (Annexin VIII) (Annexin-8)	Anxa8
WT only		TARA_MOUSE	TRIO and F-actin-binding protein (Protein Tara) (Trio-associated repeat on actin)	Triobp
WT only		RTCB_MOUSE	RNA-splicing ligase RtcB homolog (EC 6.5.1.8) (3'-phosphate/5'-hydroxy nucleic acid ligase) (Focal adhesion-associated protein) (FAAP)	Rtcb
WT only		TRAP1_MOUSE	Heat shock protein 75 kDa, mitochondrial (HSP 75) (TNFR-associated protein 1) (Tumor necrosis factor type 1 receptor-associated protein) (TRAP-1)	Trap1
WT only		ODP2_MOUSE	Dihydrolipoyllysine-residue acetyltransferase component of pyruvate dehydrogenase complex, mitochondrial (EC 2.3.1.12) (Dihydrolipoamide acetyltransferase component of pyruvate dehydrogenase complex) (Pyruvate dehydrogenase complex component E2) (PDC-E2) (PDCE2)	Dlat
WT only		HP1B3_MOUSE	Heterochromatin protein 1-binding protein 3	Hp1bp3
WT only		SPRC_MOUSE	SPARC (Basement-membrane protein 40) (BM-40) (Osteonectin) (ON) (Secreted protein acidic and rich in cysteine)	Sparc
WT only		PACN3_MOUSE	Protein kinase C and casein kinase II substrate protein 3	Pacsin3
WT only		CHRD_MOUSE	Chordin	Chrd
WT only		CYFP2_MOUSE	Cytoplasmic FMR1-interacting protein 2 (p53-inducible protein 121)	Cyfip2
WT only		OBSL1_MOUSE	Obscurin-like protein 1	Obsl1
WT only		PSMD3_MOUSE	26S proteasome non-ATPase regulatory subunit 3 (26S proteasome regulatory subunit RPN3) (26S proteasome regulatory subunit S3) (Proteasome subunit p58) (Transplantation antigen P91A) (Tum-P91A antigen)	Psmd3
WT only		GBG1_MOUSE	Guanine nucleotide-binding protein G(T) subunit gamma-T1 (Transducin gamma chain)	Gngt1
WT only		RCN2_MOUSE	Reticulocalbin-2 (Taipoxin-associated calcium-binding protein 49) (TCBP-49)	Rcn2
WT only		KPCD_MOUSE	Protein kinase C delta type (EC 2.7.11.13) (Tyrosine-protein kinase PRKCD) (EC 2.7.10.2) (nPKC-delta) [Cleaved into: Protein kinase C delta type regulatory subunit; Protein kinase C delta type catalytic subunit (Sphingosine-dependent protein kinase-1) (SDK1)]	Prkcd
WT only		ICE1_MOUSE	Little elongation complex subunit 1 (Interactor of little elongator complex ELL subunit 1)	Ice1
WT only		DDX1_MOUSE	ATP-dependent RNA helicase DDX1 (EC 3.6.4.13) (DEAD box protein 1)	Ddx1
WT only		HA1Y_MOUSE	H-2 class I histocompatibility antigen, alpha chain (Clone PAG64) (Fragment)	H2-D1
WT only		NDUV2_MOUSE	NADH dehydrogenase [ubiquinone] flavoprotein 2, mitochondrial (EC 1.6.99.3) (EC 7.1.1.2) (NADH-ubiquinone oxidoreductase 24 kDa subunit)	Ndufv2

WT only		K22E_MOUSE	Keratin, type II cytoskeletal 2 epidermal (Cytokeratin-2e) (CK-2e) (Epithelial keratin-2e) (Keratin-2 epidermis) (Keratin-2e) (K2e) (Type-II keratin Kb2)	Krt2
WT only		PUF60_MOUSE	Poly(U)-binding-splicing factor PUF60 (60 kDa poly(U)-binding-splicing factor)	Puf60
WT only		SC5A3_MOUSE	Sodium/myo-inositol cotransporter (Na(+)/myo-inositol cotransporter) (Sodium/myo-inositol transporter 1) (SMIT1) (Solute carrier family 5 member 3)	Slc5a3
WT only		NECT2_MOUSE	Nectin-2 (Herpes virus entry mediator B) (Herpesvirus entry mediator B) (HveB) (Murine herpes virus entry protein B) (mHveB) (Nectin cell adhesion molecule 2) (Poliovirus receptor homolog) (Poliovirus receptor-related protein 2) (CD antigen CD112)	Nectin2
WT only		STRAP_MOUSE	Serine-threonine kinase receptor-associated protein (UNR-interacting protein)	Strap
WT only		CX6B1_MOUSE	Cytochrome c oxidase subunit 6B1 (Cytochrome c oxidase subunit VIb isoform 1) (COX VIb-1)	Cox6b1
WT only		ABCB6_MOUSE	ATP-binding cassette sub-family B member 6, mitochondrial	Abcb6
WT only		RASA3_MOUSE	Ras GTPase-activating protein 3 (GAP1(IP4BP)) (GapIII) (Ins P4-binding protein)	Rasa3
WT only		DDX17_MOUSE	Probable ATP-dependent RNA helicase DDX17 (EC 3.6.4.13) (DEAD box protein 17)	Ddx17
WT only		HIP1R_MOUSE	Huntingtin-interacting protein 1-related protein (HIP1-related protein)	Hip1r
WT only		HXK1_MOUSE	Hexokinase-1 (EC 2.7.1.1) (Hexokinase type I) (HK I) (Hexokinase, tumor isozyme)	Hk1
WT only		AKT3_MOUSE	RAC-gamma serine/threonine-protein kinase (EC 2.7.11.1) (Protein kinase Akt-3) (Protein kinase B gamma) (PKB gamma) (RAC-PK-gamma)	Akt3
WT only		HS71B_MOUSE	Heat shock 70 kDa protein 1B (Heat shock 70 kDa protein 1) (HSP70.1)	Hspa1b
WT only		CTL2_MOUSE	Choline transporter-like protein 2 (Solute carrier family 44 member 2)	Slc44a2
WT only		LRC8C_MOUSE	Volume-regulated anion channel subunit LRRC8C (Factor for adipocyte differentiation 158) (Leucine-rich repeat-containing protein 8C)	Lrrc8c
WT only		SC23B_MOUSE	Protein transport protein Sec23B (SEC23-related protein B)	Sec23b
WT only		ALBU_MOUSE	Serum albumin	Alb
WT only		ACBP_MOUSE	Acyl-CoA-binding protein (ACBP) (Diazepam-binding inhibitor) (DBI) (Endozepine) (EP)	Dbi
WT only		FRIL1_MOUSE	Ferritin light chain 1 (Ferritin L subunit 1)	Ftl1
WT only		PSDE_MOUSE	26S proteasome non-ATPase regulatory subunit 14 (EC 3.4.19.-) (26S proteasome regulatory subunit RPN11) (MAD1)	Psmd14
WT only		SGT1_MOUSE	Protein SGT1 homolog (Suppressor of G2 allele of SKP1 homolog)	Sugt1
WT only		TXD17_MOUSE	Thioredoxin domain-containing protein 17 (14 kDa thioredoxin-related protein) (TRP14) (Protein 42-9-9) (Thioredoxin-like protein 5)	Txndc17
WT only		TMC5_MOUSE	Transmembrane channel-like protein 5	Tmc5
WT only		UBA1Y_MOUSE	Ubiquitin-like modifier-activating enzyme 1 Y (EC 6.2.1.45) (Ubiquitin-activating enzyme E1) (Ubiquitin-activating enzyme E1 Y)	Uba1y

WT only		GPDM_MOUSE	Glycerol-3-phosphate dehydrogenase, mitochondrial (GPD-M) (GPDH-M) (EC 1.1.5.3) (Protein TISP38)	Gpd2
WT only		AIMP1_MOUSE	Aminoacyl tRNA synthase complex-interacting multifunctional protein 1 (Multisynthase complex auxiliary component p43) [Cleaved into: Endothelial monocyte-activating polypeptide 2 (EMAP-2) (Endothelial monocyte-activating polypeptide II) (EMAP-II) (Small inducible cytokine subfamily E member 1)]	Aimp1
WT only		IGEB_MOUSE	IgE-binding protein	Iap
WT only		PARVB_MOUSE	Beta-parvin	Parvb
WT only		THIM_MOUSE	3-ketoacyl-CoA thiolase, mitochondrial (EC 2.3.1.16) (Acetyl-CoA acetyltransferase) (EC 2.3.1.9) (Acetyl-CoA acyltransferase) (Acyl-CoA hydrolase, mitochondrial) (EC 3.1.2.-) (EC 3.1.2.1) (EC 3.1.2.2) (Beta-ketothiolase) (Mitochondrial 3-oxoacyl-CoA thiolase)	Acaa2
WT only		ANM1_MOUSE	Protein arginine N-methyltransferase 1 (EC 2.1.1.319) (Histone-arginine N-methyltransferase PRMT1)	Prmt1
WT only		PDC6I_MOUSE	Programmed cell death 6-interacting protein (ALG-2-interacting protein 1) (ALG-2-interacting protein X) (E2F1-inducible protein) (Eig2)	Pdcd6ip
WT only		JUPI1_MOUSE	Jupiter microtubule associated homolog 1 (Hematological and neurological expressed 1 protein) [Cleaved into: Jupiter microtubule associated homolog 1, N-terminally processed]	Jpt1
WT only		K1C10_MOUSE	Keratin, type I cytoskeletal 10 (56 kDa cytokeratin) (Cytokeratin-10) (CK-10) (Keratin, type I cytoskeletal 59 kDa) (Keratin-10) (K10)	Krt10
WT only		SAHH_MOUSE	Adenosylhomocysteinase (AdoHcyase) (EC 3.3.1.1) (CUBP) (Liver copper-binding protein) (S-adenosyl-L-homocysteine hydrolase)	Ahcy
WT only		I20L2_MOUSE	Interferon-stimulated 20 kDa exonuclease-like 2 (EC 3.1.-.-)	Isg20l2
WT only		GNA13_MOUSE	Guanine nucleotide-binding protein subunit alpha-13 (G alpha-13) (G-protein subunit alpha-13)	Gna13
WT only		F193A_MOUSE	Protein FAM193A	Fam193a
WT only		STX11_MOUSE	Syntaxin-11	Stx11
WT only		PSMD5_MOUSE	26S proteasome non-ATPase regulatory subunit 5 (26S protease subunit S5 basic) (26S proteasome subunit S5B)	Psmd5
WT only		PSA3_MOUSE	Proteasome subunit alpha type-3 (EC 3.4.25.1) (Macropain subunit C8) (Multicatalytic endopeptidase complex subunit C8) (Proteasome component C8) (Proteasome subunit K)	Psma3
WT only		DAB2_MOUSE	Disabled homolog 2 (Adaptor molecule disabled-2) (Differentially expressed in ovarian carcinoma 2) (DOC-2) (Mitogen-responsive phosphoprotein)	Dab2
WT only		MUSK_MOUSE	Muscle, skeletal receptor tyrosine-protein kinase (EC 2.7.10.1) (Muscle-specific tyrosine-protein kinase receptor) (MuSK) (Muscle-specific kinase receptor)	Musk
WT only		SRP68_MOUSE	Signal recognition particle subunit SRP68 (SRP68) (Signal recognition particle 68 kDa protein)	Srp68
WT only		U2AF2_MOUSE	Splicing factor U2AF 65 kDa subunit (U2 auxiliary factor 65 kDa subunit) (U2 snRNP auxiliary factor large subunit)	U2af2

WT only		DMXL1_MOUSE	DmX-like protein 1 (X-like 1 protein)	Dmxl1
WT only		H2A1K_MOUSE	Histone H2A type 1-K	H2ac15
WT only		CYB5B_MOUSE	Cytochrome b5 type B (Cytochrome b5 outer mitochondrial membrane isoform)	Cyb5b
WT only		CLAP1_MOUSE	CLIP-associating protein 1 (Cytoplasmic linker-associated protein 1)	Clasp1
WT only		SMC2_MOUSE	Structural maintenance of chromosomes protein 2 (SMC protein 2) (SMC-2) (Chromosome-associated protein E) (FGF-inducible protein 16) (XCAP-E homolog)	Smc2
WT only		FA49B_MOUSE	Protein FAM49B	Fam49b
WT only		CLCA_MOUSE	Clathrin light chain A (Lca)	Clta
WT only		HMGN5_MOUSE	High mobility group nucleosome-binding domain-containing protein 5 (Nucleosome-binding protein 1) (Nucleosome-binding protein 45) (NBP-45) (Protein GARP45)	Hmgn5
WT only		NEP_MOUSE	Neprilysin (EC 3.4.24.11) (Atriopeptidase) (Enkephalinase) (Neutral endopeptidase 24.11) (NEP) (Neutral endopeptidase) (Skin fibroblast elastase) (SFE) (CD antigen CD10)	Mme
3Tg only	151	PLPP3_MOUSE	Phospholipid phosphatase 3 (EC 3.1.3.4) (Lipid phosphate phosphohydrolase 3) (PAP2-beta) (Phosphatidate phosphohydrolase type 2b) (Phosphatidic acid phosphatase 2b) (PAP-2b) (PAP2b)	Plpp3
3Tg only		TYB4_MOUSE	Thymosin beta-4 (T beta 4) [Cleaved into: Hematopoietic system regulatory peptide (Seraspenide)]	Tmsb4x
3Tg only		SSRG_MOUSE	Translocon-associated protein subunit gamma (TRAP-gamma) (Signal sequence receptor subunit gamma) (SSR-gamma)	Ssr3
3Tg only		ANX11_MOUSE	Annexin A11 (Annexin XI) (Annexin-11) (Calcyclin-associated annexin 50) (CAP-50)	Anxa11
3Tg only		SYYC_MOUSE	Tyrosine--tRNA ligase, cytoplasmic (EC 6.1.1.1) (Tyrosyl-tRNA synthetase) (TyrRS) [Cleaved into: Tyrosine--tRNA ligase, cytoplasmic, N-terminally processed]	Yars
3Tg only		K1C14_MOUSE	Keratin, type I cytoskeletal 14 (Cytokeratin-14) (CK-14) (Keratin-14) (K14)	Krt14
3Tg only		EPHA6_MOUSE	Ephrin type-A receptor 6 (EC 2.7.10.1) (EPH homology kinase 2) (EHK-2)	Epha6
3Tg only		MDR1B_MOUSE	ATP-dependent translocase ABCB1 (ATP-binding cassette sub-family B member 1B) (Multidrug resistance protein 1B) (EC 7.6.2.2) (P-glycoprotein 1) (Phospholipid transporter ABCB1) (EC 7.6.2.1) (CD antigen CD243)	Abcb1b
3Tg only		GRB2_MOUSE	Growth factor receptor-bound protein 2 (Adapter protein GRB2) (SH2/SH3 adapter GRB2)	Grb2
3Tg only		CCDC9_MOUSE	Coiled-coil domain-containing protein 9	Ccdc9
3Tg only		UB2D2_MOUSE	Ubiquitin-conjugating enzyme E2 D2 (EC 2.3.2.23) ((E3-independent) E2 ubiquitin-conjugating enzyme D2) (EC 2.3.2.24) (E2 ubiquitin-conjugating enzyme D2) (Ubiquitin carrier protein D2) (Ubiquitin-conjugating enzyme E2(17)KB 2) (Ubiquitin-conjugating enzyme E2-17 kDa 2) (Ubiquitin-protein ligase D2)	Ube2d2
3Tg only		CDV3_MOUSE	Protein CDV3 (Carnitine deficiency-associated protein 3) (Tyrosine-phosphorylated protein 36) (TPP36)	Cdv3

3Tg only		PAK2_MOUSE	Serine/threonine-protein kinase PAK 2 (EC 2.7.11.1) (Gamma-PAK) (p21-activated kinase 2) (PAK-2) [Cleaved into: PAK-2p27; PAK-2p34]	Pak2
3Tg only		CHP1_MOUSE	Calcineurin B homologous protein 1 (Calcineurin B-like protein) (Calcium-binding protein CHP) (Calcium-binding protein p22) (EF-hand calcium-binding domain-containing protein p22) (Sid 470) (p24)	Chp1
3Tg only		EIF3H_MOUSE	Eukaryotic translation initiation factor 3 subunit H (eIF3h) (Eukaryotic translation initiation factor 3 subunit 3) (eIF-3-gamma) (eIF3 p40 subunit)	Eif3h
3Tg only		LMNB2_MOUSE	Lamin-B2	Lmnb2
3Tg only		SEC13_MOUSE	Protein SEC13 homolog (GATOR complex protein SEC13) (SEC13-like protein 1) (SEC13-related protein)	Sec13
3Tg only		LYOX_MOUSE	Protein-lysine 6-oxidase (EC 1.4.3.13) (Lysyl oxidase) (Ras excision protein) [Cleaved into: Protein-lysine 6-oxidase, long form; Protein-lysine 6-oxidase, short form]	Lox
3Tg only		TM1L2_MOUSE	TOM1-like protein 2 (Target of Myb-like protein 2)	Tom1l2
3Tg only		MRCKB_MOUSE	Serine/threonine-protein kinase MRCK beta (EC 2.7.11.1) (CDC42-binding protein kinase beta) (DMPK-like beta) (Myotonic dystrophy kinase-related CDC42-binding kinase beta) (MRCK beta) (Myotonic dystrophy protein kinase-like beta)	Cdc42bpb
3Tg only		RFIP5_MOUSE	Rab11 family-interacting protein 5 (Rab11-FIP5) (Rab11-interacting protein Rip11)	Rab11fip5
3Tg only		K2C5_MOUSE	Keratin, type II cytoskeletal 5 (Cytokeratin-5) (CK-5) (Keratin-5) (K5) (Type-II keratin Kb5)	Krt5
3Tg only		VAMP2_MOUSE	Vesicle-associated membrane protein 2 (VAMP-2) (Synaptobrevin-2)	Vamp2
3Tg only		RILP_MOUSE	Rab-interacting lysosomal protein	Rilp
3Tg only		H2B3B_MOUSE	Histone H2B type 3-B	Hist3h2bb
3Tg only		KLC2_MOUSE	Kinesin light chain 2 (KLC 2)	Klc2
3Tg only		CDK1_MOUSE	Cyclin-dependent kinase 1 (CDK1) (EC 2.7.11.22) (EC 2.7.11.23) (Cell division control protein 2 homolog) (Cell division protein kinase 1) (p34 protein kinase)	Cdk1
3Tg only		CTR1_MOUSE	High affinity cationic amino acid transporter 1 (CAT-1) (CAT1) (Ecotropic retroviral leukemia receptor) (Ecotropic retrovirus receptor) (ERR) (Solute carrier family 7 member 1) (System Y+ basic amino acid transporter)	Slc7a1
3Tg only		EIF3C_MOUSE	Eukaryotic translation initiation factor 3 subunit C (eIF3c) (Eukaryotic translation initiation factor 3 subunit 8) (eIF3 p110)	Eif3c
3Tg only		VAMP5_MOUSE	Vesicle-associated membrane protein 5 (VAMP-5) (Myobrevin)	Vamp5
3Tg only		CD97_MOUSE	CD97 antigen (CD antigen CD97) [Cleaved into: CD97 antigen subunit alpha; CD97 antigen subunit beta]	Cd97
3Tg only		PSA5_MOUSE	Proteasome subunit alpha type-5 (EC 3.4.25.1) (Macropain zeta chain) (Multicatalytic endopeptidase complex zeta chain) (Proteasome zeta chain)	Psma5
3Tg only		NOMO1_MOUSE	Nodal modulator 1	Nomo1
3Tg only		ATPD_MOUSE	ATP synthase subunit delta, mitochondrial (ATP synthase F1 subunit delta) (F-ATPase delta subunit)	Atp5f1d

3Tg only		PCBP2_MOUSE	Poly(rC)-binding protein 2 (Alpha-CP2) (CTBP) (CBP) (Putative heterogeneous nuclear ribonucleoprotein X) (hnRNP X)	Pcbp2
3Tg only		AGRL2_MOUSE	Adhesion G protein-coupled receptor L2 (Calcium-independent alpha-latrotoxin receptor 2) (CIRL-2) (Latrophilin-2)	Adgrl2
3Tg only		ADAM7_MOUSE	Disintegrin and metalloproteinase domain-containing protein 7 (ADAM 7)	Adam7
3Tg only		AP3S1_MOUSE	AP-3 complex subunit sigma-1 (AP-3 complex subunit sigma-3A) (Adaptor-related protein complex 3 subunit sigma-1) (Sigma-3A-adaptin) (Sigma3A-adaptin) (Sigma-adaptin 3a)	Ap3s1
3Tg only		RAI3_MOUSE	Retinoic acid-induced protein 3 (G-protein coupled receptor family C group 5 member A) (Retinoic acid-induced gene 1 protein) (RAIG-1)	Gprc5a
3Tg only		LTOR4_MOUSE	Ragulator complex protein LAMTOR4 (Late endosomal/lysosomal adaptor and MAPK and MTOR activator 4) [Cleaved into: Ragulator complex protein LAMTOR4, N-terminally processed]	Lamtor4
3Tg only		PFD2_MOUSE	Prefoldin subunit 2	Pfdn2
3Tg only		TPBG_MOUSE	Trophoblast glycoprotein (5T4 oncofetal trophoblast glycoprotein) (5T4 oncotrophoblast glycoprotein) (Wnt-activated inhibitory factor 1) (WAIF1)	Tpbg
3Tg only		STK25_MOUSE	Serine/threonine-protein kinase 25 (EC 2.7.11.1) (Ste20-like kinase) (Sterile 20/oxidant stress-response kinase 1) (SOK-1) (Ste20/oxidant stress response kinase 1)	Stk25
3Tg only		H2A2A_MOUSE	Histone H2A type 2-A (H2a-614) (H2a-615) (Histone H2A.2)	Hist2h2aa1;
3Tg only		LYZ1_MOUSE	Lysozyme C-1 (EC 3.2.1.17) (1,4-beta-N-acetylmuramidase C) (Lysozyme C type P)	Lyz1
3Tg only		GNAI3_MOUSE	Guanine nucleotide-binding protein G(i) subunit alpha (G(i) alpha-3)	Gnai3
3Tg only		VATE1_MOUSE	V-type proton ATPase subunit E 1 (V-ATPase subunit E 1) (V-ATPase 31 kDa subunit) (p31) (Vacuolar proton pump subunit E 1)	Atp6v1e1
3Tg only		QCR2_MOUSE	Cytochrome b-c1 complex subunit 2, mitochondrial (Complex III subunit 2) (Core protein II) (Ubiquinol-cytochrome-c reductase complex core protein 2)	Uqcrc2
3Tg only		HRG1_MOUSE	Heme transporter HRG1 (Heme-responsive gene 1 protein homolog) (HRG-1) (Solute carrier family 48 member 1)	Slc48a1
3Tg only		PRPS1_MOUSE	Ribose-phosphate pyrophosphokinase 1 (EC 2.7.6.1) (Phosphoribosyl pyrophosphate synthase I) (PRS-I)	Prps1
3Tg only		L2GL1_MOUSE	Lethal(2) giant larvae protein homolog 1 (LLGL) (Mgl-1) (Mlgl)	Llgl1
3Tg only		K2C1B_MOUSE	Keratin, type II cytoskeletal 1b (Cytokeratin-1B) (CK-1B) (Embryonic type II keratin-1) (Keratin-77) (K77) (Type-II keratin Kb39)	Krt77
3Tg only		PSN1_MOUSE	Presenilin-1 (PS-1) (EC 3.4.23.-) (Protein S182) [Cleaved into: Presenilin-1 NTF subunit; Presenilin-1 CTF subunit; Presenilin-1 CTF12 (PS1-CTF12)]	Psen1
3Tg only		RPGP2_MOUSE	Rap1 GTPase-activating protein 2 (Rap1GAP2) (GTPase-activating Rap/Ran-GAP domain-like protein 4)	Rap1gap2

3Tg only		BI2L2_MOUSE	Brain-specific angiogenesis inhibitor 1-associated protein 2-like protein 2 (BAI1-associated protein 2-like protein 2) (Planar intestinal- and kidney-specific BAR domain protein) (Pinkbar)	Baiap2l2
3Tg only		ATS15_MOUSE	A disintegrin and metalloproteinase with thrombospondin motifs 15 (ADAM-TS 15) (ADAM-TS15) (ADAMTS-15) (EC 3.4.24.-)	Adamts15
3Tg only		S10A4_MOUSE	Protein S100-A4 (Metastasin) (Metastatic cell protein) (PEL98) (Placental calcium-binding protein) (Protein 18A2) (Protein Mts1) (S100 calcium-binding protein A4)	S100a4
3Tg only		USO1_MOUSE	General vesicular transport factor p115 (Protein USO1 homolog) (Transcytosis-associated protein) (TAP) (Vesicle-docking protein)	Uso1
3Tg only		MARK2_MOUSE	Serine/threonine-protein kinase MARK2 (EC 2.7.11.1) (EC 2.7.11.26) (ELKL motif kinase 1) (EMK-1) (MAP/microtubule affinity-regulating kinase 2) (PAR1 homolog) (PAR1 homolog b) (Par-1b) (mPar-1b)	Mark2
3Tg only		NTF2_MOUSE	Nuclear transport factor 2 (NTF-2)	Nutf2
3Tg only		MOB1B_MOUSE	MOB kinase activator 1B (Mob1 homolog 1A) (Mps one binder kinase activator-like 1A)	Mob1b
3Tg only		VAMP7_MOUSE	Vesicle-associated membrane protein 7 (VAMP-7) (Synaptobrevin-like protein 1)	Vamp7
3Tg only		SYPL1_MOUSE	Synaptophysin-like protein 1 (Pantophysin)	Sypl1
3Tg only		HS74L_MOUSE	Heat shock 70 kDa protein 4L (Heat shock 70-related protein APG-1) (Osmotic stress protein 94)	Hspa4l
3Tg only		HSPB8_MOUSE	Heat shock protein beta-8 (HspB8) (Alpha-crystallin C chain) (Small stress protein-like protein HSP22)	Hspb8
3Tg only		CYBP_MOUSE	Calcyclin-binding protein (CacyBP) (Siah-interacting protein)	Cacybp
3Tg only		CDIPT_MOUSE	CDP-diacylglycerol--inositol 3-phosphatidyltransferase (EC 2.7.8.11) (Phosphatidylinositol synthase) (PI synthase) (PtdIns synthase)	Cdipt
3Tg only		RHG01_MOUSE	Rho GTPase-activating protein 1 (Rho-type GTPase-activating protein 1)	Arhgap1
3Tg only		NCK5L_MOUSE	Nck-associated protein 5-like (Centrosomal protein of 169 kDa) (Cep169)	Nckap5l
3Tg only		LMAN2_MOUSE	Vesicular integral-membrane protein VIP36 (Lectin mannose-binding 2) (Vesicular integral-membrane protein 36) (VIP36)	Lman2
3Tg only		FUMH_MOUSE	Fumarate hydratase, mitochondrial (Fumarase) (EC 4.2.1.2) (EF-3)	Fh
3Tg only		HAP28_MOUSE	28 kDa heat- and acid-stable phosphoprotein (PDGF-associated protein) (PAP) (PDGFA-associated protein 1) (PAP1)	Pdap1
3Tg only		KCC2D_MOUSE	Calcium/calmodulin-dependent protein kinase type II subunit delta (CaM kinase II subunit delta) (CaMK-II subunit delta) (EC 2.7.11.17)	Camk2d
3Tg only		VGFR3_MOUSE	Vascular endothelial growth factor receptor 3 (VEGFR-3) (EC 2.7.10.1) (Fms-like tyrosine kinase 4) (FLT-4) (Tyrosine-protein kinase receptor FLT4)	Flt4
3Tg only		GALK1_MOUSE	Galactokinase (EC 2.7.1.6) (Galactose kinase)	Galk1
3Tg only		KPRB_MOUSE	Phosphoribosyl pyrophosphate synthase-associated protein 2 (PRPP synthase-associated protein 2) (41 kDa phosphoribosypyrophosphate synthetase-associated protein) (PAP41)	Prpsap2

3Tg only		PI51A_MOUSE	Phosphatidylinositol 4-phosphate 5-kinase type-1 alpha (PIP5K1-alpha) (PtdIns(4)P-5-kinase 1 alpha) (EC 2.7.1.68) (68 kDa type I phosphatidylinositol 4-phosphate 5-kinase) (Phosphatidylinositol 4-phosphate 5-kinase type I alpha) (PIP5KIalpha) (Phosphatidylinositol 4-phosphate 5-kinase type I beta) (PI4P5KIbeta)	Pip5k1a
3Tg only		LCAP_MOUSE	Leucyl-cystinyl aminopeptidase (Cystinyl aminopeptidase) (EC 3.4.11.3) (Oxytocinase) (OTase)	Lnpep
3Tg only		KRT36_MOUSE	Keratin, type I cuticular Ha6 (Keratin-36) (K36) (Keratin-5) (MHRa-1) (Type I keratin 48 kDa)	Krt36
3Tg only		TX1B3_MOUSE	Tax1-binding protein 3 (Tax interaction protein 1) (TIP-1)	Tax1bp3
3Tg only		S12A7_MOUSE	Solute carrier family 12 member 7 (Electroneutral potassium-chloride cotransporter 4) (K-Cl cotransporter 4)	Slc12a7
3Tg only		S4A7_MOUSE	Sodium bicarbonate cotransporter 3 (Solute carrier family 4 member 7)	Slc4a7
3Tg only		RMP_MOUSE	Unconventional prefoldin RPB5 interactor (Protein NNX3) (Protein phosphatase 1 regulatory subunit 19) (RNA polymerase II subunit 5-mediating protein) (RPB5-mediating protein)	Uri1
3Tg only		MYO1D_MOUSE	Unconventional myosin-Id	Myo1d
3Tg only		SC31A_MOUSE	Protein transport protein Sec31A (SEC31-like protein 1) (SEC31-related protein A)	Sec31a
3Tg only		EMC2_MOUSE	ER membrane protein complex subunit 2 (Tetratricopeptide repeat protein 35) (TPR repeat protein 35)	Emc2
3Tg only		STT3B_MOUSE	Dolichyl-diphosphooligosaccharide--protein glycosyltransferase subunit STT3B (Oligosaccharyl transferase subunit STT3B) (STT3-B) (EC 2.4.99.18) (B6dom1 antigen) (Source of immunodominant MHC-associated peptides)	Stt3b
3Tg only		H2A1H_MOUSE	Histone H2A type 1-H	Hist1h2ah
3Tg only		LRC47_MOUSE	Leucine-rich repeat-containing protein 47	Lrrc47
3Tg only		PURB_MOUSE	Transcriptional activator protein Pur-beta (Purine-rich element-binding protein B) (Vascular actin single-stranded DNA-binding factor 2 p44 component)	Purb
3Tg only		CTDS1_MOUSE	Carboxy-terminal domain RNA polymerase II polypeptide A small phosphatase 1 (EC 3.1.3.16) (Golli-interacting protein) (GIP) (Nuclear LIM interactor-interacting factor 3) (NLI-interacting factor 3) (Small C-terminal domain phosphatase 1) (SCP1) (Small CTD phosphatase 1)	Ctdsp1
3Tg only		ERC2_MOUSE	ERC protein 2 (CAZ-associated structural protein 1) (CAST1)	Erc2
3Tg only		NFAC2_MOUSE	Nuclear factor of activated T-cells, cytoplasmic 2 (NF-ATc2) (NFATc2) (NFAT pre-existing subunit) (NF-ATp) (T-cell transcription factor NFAT1)	Nfatc2
3Tg only		PIEZ1_MOUSE	Piezo-type mechanosensitive ion channel component 1 (Protein FAM38A)	Piezo1
3Tg only		LYRIC_MOUSE	Protein LYRIC (3D3/LYRIC) (Lysine-rich CEACAM1 co-isolated protein) (Metadherin) (Metastasis adhesion protein)	Mtdh
3Tg only		NCPR_MOUSE	NADPH--cytochrome P450 reductase (CPR) (P450R) (EC 1.6.2.4)	Por

3Tg only		EXOC1_MOUSE	Exocyst complex component 1 (Exocyst complex component Sec3)	Exoc1
3Tg only		VINEX_MOUSE	Vinexin (SH3 domain-containing protein SH3P3) (SH3-containing adapter molecule 1) (SCAM-1) (Sorbin and SH3 domain-containing protein 3)	Sorbs3
3Tg only		SYAC_MOUSE	Alanine--tRNA ligase, cytoplasmic (EC 6.1.1.7) (Alanyl-tRNA synthetase) (AlaRS) (Protein sticky) (Sti)	Aars
3Tg only		COPT1_MOUSE	High affinity copper uptake protein 1 (Copper transporter 1) (CTR1) (Solute carrier family 31 member 1)	Slc31a1
3Tg only		ASM3B_MOUSE	Acid sphingomyelinase-like phosphodiesterase 3b (ASM-like phosphodiesterase 3b) (EC 3.1.4.-)	Smpdl3b
3Tg only		DCTN5_MOUSE	Dynactin subunit 5 (Dynactin subunit p25)	Dctn5
3Tg only		CD2AP_MOUSE	CD2-associated protein (Mesenchyme-to-epithelium transition protein with SH3 domains 1) (METS-1)	Cd2ap
3Tg only		OGG1_MOUSE	N-glycosylase/DNA lyase [Includes: 8-oxoguanine DNA glycosylase (EC 3.2.2.-); DNA-(apurinic or apyrimidinic site) lyase (AP lyase) (EC 4.2.99.18)]	Ogg1
3Tg only		EM55_MOUSE	55 kDa erythrocyte membrane protein (p55) (Membrane protein, palmitoylated 1)	Mpp1
3Tg only		EMC7_MOUSE	ER membrane protein complex subunit 7	Emc7
3Tg only		K0754_MOUSE	Uncharacterized protein KIAA0754	Kiaa0754
3Tg only		GPM6B_MOUSE	Neuronal membrane glycoprotein M6-b (M6b)	Gpm6b
3Tg only		SRBS2_MOUSE	Sorbin and SH3 domain-containing protein 2 (Arg-binding protein 2) (ArgBP2) (Arg/Abl-interacting protein 2)	Sorbs2
3Tg only		S12A2_MOUSE	Solute carrier family 12 member 2 (Basolateral Na-K-Cl symporter) (Bumetanide-sensitive sodium-(potassium)-chloride cotransporter 2)	Slc12a2
3Tg only		JAK1_MOUSE	Tyrosine-protein kinase JAK1 (EC 2.7.10.2) (Janus kinase 1) (JAK-1)	Jak1
3Tg only		TGBR3_MOUSE	Transforming growth factor beta receptor type 3 (TGF-beta receptor type 3) (TGFR-3) (Betaglycan) (Transforming growth factor beta receptor III) (TGF-beta receptor type III)	Tgfbr3
3Tg only		ITA6_MOUSE	Integrin alpha-6 (CD49 antigen-like family member F) (VLA-6) (CD antigen CD49f) [Cleaved into: Integrin alpha-6 heavy chain; Integrin alpha-6 light chain; Processed integrin alpha-6 (Alpha6p)]	Itga6
3Tg only		OX2G_MOUSE	OX-2 membrane glycoprotein (MRC OX-2 antigen) (CD antigen CD200)	Cd200
3Tg only		CCD47_MOUSE	Coiled-coil domain-containing protein 47 (Adipocyte-specific protein 4) (Calumin)	Ccdc47
3Tg only		SAP3_MOUSE	Ganglioside GM2 activator (Cerebroside sulfate activator protein) (GM2-AP) (Sphingolipid activator protein 3) (SAP-3)	Gm2a
3Tg only		ALDOC_MOUSE	Fructose-bisphosphate aldolase C (EC 4.1.2.13) (Aldolase 3) (Brain-type aldolase) (Scrapie-responsive protein 2) (Zebrin II)	Aldoc
3Tg only		CDK13_MOUSE	Cyclin-dependent kinase 13 (EC 2.7.11.22) (EC 2.7.11.23) (CDC2-related protein kinase 5) (Cell division cycle 2-like protein kinase 5) (Cell division protein kinase 13)	Cdk13

3Tg only		ADAM9_MOUSE	Disintegrin and metalloproteinase domain-containing protein 9 (ADAM 9) (EC 3.4.24.-) (Meltrin-gamma) (Metalloprotease/disintegrin/cysteine-rich protein 9) (Myeloma cell metalloproteinase)	Adam9
3Tg only		IPO9_MOUSE	Importin-9 (Imp9) (Importin-9a) (Imp9a) (Importin-9b) (Imp9b) (Ran-binding protein 9) (RanBP9)	Ipo9
3Tg only		VATC1_MOUSE	V-type proton ATPase subunit C 1 (V-ATPase subunit C 1) (Vacuolar proton pump subunit C 1)	Atp6v1c1
3Tg only		MMP14_MOUSE	Matrix metalloproteinase-14 (MMP-14) (EC 3.4.24.80) (MMP-X1) (MT-MMP) (Membrane-type matrix metalloproteinase 1) (MT-MMP 1) (MTMMP1) (Membrane-type-1 matrix metalloproteinase) (MT1-MMP) (MT1MMP)	Mmp14
3Tg only		IF4H_MOUSE	Eukaryotic translation initiation factor 4H (eIF-4H) (Williams-Beuren syndrome chromosomal region 1 protein homolog)	Eif4h
3Tg only		KCY_MOUSE	UMP-CMP kinase (EC 2.7.4.14) (Deoxycytidylate kinase) (CK) (dCMP kinase) (Nucleoside-diphosphate kinase) (EC 2.7.4.6) (Uridine monophosphate/cytidine monophosphate kinase) (UMP/CMP kinase) (UMP/CMPK)	Cmpk1
3Tg only		RLA2_MOUSE	60S acidic ribosomal protein P2	Rplp2
3Tg only		GAK_MOUSE	Cyclin-G-associated kinase (EC 2.7.11.1)	Gak
3Tg only		TADBP_MOUSE	TAR DNA-binding protein 43 (TDP-43)	Tardbp
3Tg only		CGT_MOUSE	2-hydroxyacylsphingosine 1-beta-galactosyltransferase (EC 2.4.1.47) (Ceramide UDP-galactosyltransferase) (Cerebroside synthase) (UDP-galactose-ceramide galactosyltransferase)	Ugt8
3Tg only		BAG3_MOUSE	BAG family molecular chaperone regulator 3 (BAG-3) (Bcl-2-associated athanogene 3) (Bcl-2-binding protein Bis)	Bag3
3Tg only		MOB1A_MOUSE	MOB kinase activator 1A (Mob1 homolog 1B) (Mps one binder kinase activator-like 1B)	Mob1a
3Tg only		SH3R3_MOUSE	E3 ubiquitin-protein ligase SH3RF3 (EC 2.3.2.27) (Plenty of SH3s 2) (SH3 domain-containing RING finger protein 3) (SH3 multiple domains protein 4)	Sh3rf3
3Tg only		CSK2B_MOUSE	Casein kinase II subunit beta (CK II beta) (Phosvitin)	Csnk2b
3Tg only		NDUS8_MOUSE	NADH dehydrogenase [ubiquinone] iron-sulfur protein 8, mitochondrial (EC 1.6.99.3) (EC 7.1.1.2) (Complex I-23kD) (CI-23kD) (NADH-ubiquinone oxidoreductase 23 kDa subunit)	Ndufs8
3Tg only		LRC57_MOUSE	Leucine-rich repeat-containing protein 57	Lrrc57
3Tg only		ECHB_MOUSE	Trifunctional enzyme subunit beta, mitochondrial (TP-beta) [Includes: 3-ketoacyl-CoA thiolase (EC 2.3.1.155) (EC 2.3.1.16) (Acetyl-CoA acyltransferase) (Beta-ketothiolase)]	Hadhb
3Tg only		ATP5J_MOUSE	ATP synthase-coupling factor 6, mitochondrial (ATPase subunit F6) (ATP synthase peripheral stalk subunit F6)	Atp5pf
3Tg only		DEGS1_MOUSE	Sphingolipid delta(4)-desaturase DES1 (EC 1.14.19.17) (Degenerative spermatocyte homolog 1)	Degs1
3Tg only		YBOX2_MOUSE	Y-box-binding protein 2 (FRGY2 homolog) (Germ cell-specific Y-box-binding protein)	Ybx2
3Tg only		YES_MOUSE	Tyrosine-protein kinase Yes (EC 2.7.10.2) (Proto-oncogene c-Yes) (p61-Yes)	Yes1
3Tg only		CAV2_MOUSE	Caveolin-2	Cav2

3Tg only		IRS2_MOUSE	Insulin receptor substrate 2 (IRS-2) (4PS)	Irs2
3Tg only		UFO_MOUSE	Tyrosine-protein kinase receptor UFO (EC 2.7.10.1) (Adhesion-related kinase)	Axl
3Tg only		MB12A_MOUSE	Multivesicular body subunit 12A (ESCRT-I complex subunit MVB12A) (Protein FAM125A)	Mvb12a
3Tg only		CBPZ_MOUSE	Carboxypeptidase Z (CPZ) (EC 3.4.17.-)	Cpz
3Tg only		CISD2_MOUSE	CDGSH iron-sulfur domain-containing protein 2 (MitoNEET-related 1 protein) (Miner1) (Nervous system overexpressed protein 70)	Cisd2
3Tg only		DJC10_MOUSE	DnaJ homolog subfamily C member 10 (EC 1.8.4.-) (Endoplasmic reticulum DNA J domain-containing protein 5) (ER-resident protein ERdj5) (ERdj5) (Endoplasmic reticulum DnaJ-PDI fusion protein 1) (J domain-containing protein disulfide isomerase-like protein) (J domain-containing PDI-like protein) (JPDI)	Dnajc10
3Tg only		ASCC2_MOUSE	Activating signal cointegrator 1 complex subunit 2 (ASC-1 complex subunit p100) (Trip4 complex subunit p100)	Ascc2
3Tg only		SYDC_MOUSE	Aspartate--tRNA ligase, cytoplasmic (EC 6.1.1.12) (Aspartyl-tRNA synthetase) (AspRS)	Dars
3Tg only		CSKI2_MOUSE	Caskin-2	Caskin2
3Tg only		DC1L2_MOUSE	Cytoplasmic dynein 1 light intermediate chain 2 (Dynein light intermediate chain 2, cytosolic)	Dync1li2
3Tg only		QCR1_MOUSE	Cytochrome b-c1 complex subunit 1, mitochondrial (Complex III subunit 1) (Core protein I) (Ubiquinol-cytochrome-c reductase complex core protein 1)	Uqcrc1
