# Supplementary material for: Proteomic analysis links alterations of bioenergetics, mitochondria-ER interactions and proteostasis in hippocampal astrocytes from 3xTg-AD mice
Source: Cell Death Dis. 2020 Aug 18;11(8):645. doi: 10.1038/s41419-020-02911-1 (PMC7434916; doi:10.1038/s41419-020-02911-1)
Supplement: Supplementary file 3 — Supplemental Table 1b [file 41419_2020_2911_MOESM3_ESM.pdf]

Supplementary Table 1b. Identified proteins in MERE fraction of WT-iAstro and 3Tg-iAstro cells.

N = 5 biological replicates were used for each genotype

Uniprot_KB	Uniprot_ID	Description	p-value	Log(10)FC
O35639	ANXA3_MOUSE	Annexin A3 OS=Mus musculus GN=Anxa3 PE=1 SV=4	0.00059	0.626
P35762	CD81_MOUSE	CD81 antigen OS=Mus musculus GN=Cd81 PE=1 SV=2	4.38E-05	0.498
Q8BTY2	S4A7_MOUSE	sp|Q8BTY2|S4A7_MOUSE Sodium bicarbonate cotransporter 3 OS=Mus musculus GN=Slc4a7 PE=1 SV=2	0.01468	0.426
Q5ND29	RILP_MOUSE	sp|Q5ND29|RILP_MOUSE Rab-interacting lysosomal protein OS=Mus musculus GN=Rilp PE=1 SV=1	0.008	0.393
P01831	THY1_MOUSE	Thy-1 membrane glycoprotein OS=Mus musculus GN=Thy1 PE=1 SV=1	0.00091	0.366
O88952	LIN7C_MOUSE	sp|O88952|LIN7C_MOUSE Protein lin-7 homolog C OS=Mus musculus GN=Lin7c PE=1 SV=2	0.00184	0.361
Q99JW4	LIMS1_MOUSE	sp|Q99JW4|LIMS1_MOUSE LIM and senescent cell antigen-like-containing domain protein 1 OS=Mus musculus GN=Lims1 PE=1 SV=3	0.04463	0.359
P70296	PEBP1_MOUSE	Phosphatidylethanolamine-binding protein 1 OS=Mus musculus GN=Pebp1 PE=1 SV=3	0.015	0.334
Q62188	DPYL3_MOUSE	Dihydropyrimidinase-related protein 3 OS=Mus musculus GN=Dpysl3 PE=1 SV=1	0.00424	0.331
P46460	NSF_MOUSE	sp|P46460|NSF_MOUSE Vesicle-fusing ATPase OS=Mus musculus GN=Nsf PE=1 SV=2	0.01152	0.309
O08992	SDCB1_MOUSE	sp|O08992|SDCB1_MOUSE Syntenin-1 OS=Mus musculus GN=Sdcbp PE=1 SV=1	0.03294	0.273
Q811D0	DLG1_MOUSE	sp|Q811D0-2|DLG1_MOUSE Isoform 2 of Disks large homolog 1 OS=Mus musculus GN=Dlg1	0.03055	0.209
Q9Z2P8	VAMP5_MOUSE	sp|Q9Z2P8|VAMP5_MOUSE Vesicle-associated membrane protein 5 OS=Mus musculus GN=Vamp5 PE=1 SV=1	0.00884	0.203
Q5SYD0	MYO1D_MOUSE	sp|Q5SYD0|MYO1D_MOUSE Unconventional myosin-Id OS=Mus musculus GN=Myo1d PE=1 SV=1	0.02917	0.191
Q60634	FLOT2_MOUSE	sp|Q60634-2|FLOT2_MOUSE Isoform 2 of Flotillin-2 OS=Mus musculus GN=Flot2	0.04965	0.191
P11688	ITA5_MOUSE	Integrin alpha-5 OS=Mus musculus GN=Itga5 PE=1 SV=3	0.01541	0.155
P29533	VCAM1_MOUSE	sp|P29533|VCAM1_MOUSE Vascular cell adhesion protein 1 OS=Mus musculus GN=Vcam1 PE=1 SV=1	0.00118	0.148
P97447	FHL1_MOUSE	Four and a half LIM domains protein 1 OS=Mus musculus GN=Fhl1 PE=1 SV=3	0.01986	0.135
P10852	4F2_MOUSE	sp|P10852-2|4F2_MOUSE Isoform 2 of 4F2 cell-surface antigen heavy chain OS=Mus musculus GN=Slc3a2	0.01998	0.135
P18872-2	GNAO_MOUSE	sp|P18872-2|GNAO_MOUSE Isoform Alpha-2 of Guanine nucleotide-binding protein G(o) subunit alpha OS=Mus musculus GN=Gnao1	0.00475	0.134
P57776-3	EF1D_MOUSE	Isoform 3 of Elongation factor 1-delta OS=Mus musculus GN=Eef1d	0.0148	-0.115
Q9D8N0	EF1G_MOUSE	sp|Q9D8N0|EF1G_MOUSE Elongation factor 1-gamma OS=Mus musculus GN=Eef1g PE=1 SV=3	0.03828	-0.119
P80314	TCPB_MOUSE	T-complex protein 1 subunit beta OS=Mus musculus GN=Cct2 PE=1 SV=4	0.02552	-0.142
P27773	PDIA3_MOUSE	Protein disulfide-isomerase A3 OS=Mus musculus GN=Pdia3 PE=1 SV=2	0.00527	-0.157
P11276	FINC_MOUSE	sp|P11276|FINC_MOUSE Fibronectin OS=Mus musculus GN=Fn1 PE=1 SV=4	0.001	-0.176
Q9CQ19	MYL9_MOUSE	Myosin regulatory light polypeptide 9 OS=Mus musculus GN=Myl9 PE=1 SV=3	0.03953	-0.178

O08585	CLCA_MOUSE	Clathrin light chain A OS=Mus musculus GN=Clta PE=1 SV=2	0.03658	-0.190
Q9D1D4	TMEDA_MOUSE	Transmembrane emp24 domain-containing protein 10 OS=Mus musculus GN=Tmed10 PE=1 SV=1	0.00407	-0.192
Q9CQU0	TXD12_MOUSE	Thioredoxin domain-containing protein 12 OS=Mus musculus GN=Txndc12 PE=1 SV=1	0.0439	-0.193
Q9R0P5	DEST_MOUSE	Destrin OS=Mus musculus GN=Dstn PE=1 SV=3	0.00093	-0.193
P62814	VATB2_MOUSE	V-type proton ATPase subunit B, brain isoform OS=Mus musculus GN=Atp6v1b2 PE=1 SV=1	0.00674	-0.199
Q9D819	IPYR_MOUSE	Inorganic pyrophosphatase OS=Mus musculus GN=Ppa1 PE=1 SV=1	0.03295	-0.218
O54734	OST48_MOUSE	sp|O54734|OST48_MOUSE Dolichyl-diphosphooligosaccharide--protein glycosyltransferase 48 kDa subunit OS=Mus musculus GN=Ddost PE=1 SV=2	0.03355	-0.220
Q8BHN3	GANAB_MOUSE	sp|Q8BHN3-2|GANAB_MOUSE Isoform 2 of Neutral alpha-glucosidase AB OS=Mus musculus GN=Ganab	0.04194	-0.222
Q922F4	TBB6_MOUSE	Tubulin beta-6 chain OS=Mus musculus GN=Tubb6 PE=1 SV=1	0.01166	-0.234
Q9JK48	SHLB1_MOUSE	Endophilin-B1 OS=Mus musculus GN=Sh3glb1 PE=1 SV=1	0.03568	-0.235
Q80UG5-3	SEPT9_MOUSE	Isoform 3 of Septin-9 OS=Mus musculus GN=Sept9	0.00318	-0.256
P63276	RS17_MOUSE	40S ribosomal protein S17 OS=Mus musculus GN=Rps17 PE=1 SV=2	0.01426	-0.271
O08547	SC22B_MOUSE	sp|O08547|SC22B_MOUSE Vesicle-trafficking protein SEC22b OS=Mus musculus GN=Sec22b PE=1 SV=3	0.02525	-0.273
P30412	PPIC_MOUSE	sp|P30412|PPIC_MOUSE Peptidyl-prolyl cis-trans isomerase C OS=Mus musculus GN=Ppic PE=1 SV=1	0.00717	-0.298
Q61937	NPM_MOUSE	sp|Q61937|NPM_MOUSE Nucleophosmin OS=Mus musculus GN=Npm1 PE=1 SV=1	0.02831	-0.299
P21956	MFGM_MOUSE	sp|P21956-2|MFGM_MOUSE Isoform 2 of Lactadherin OS=Mus musculus GN=Mfge8	0.0283	-0.320
P62965	RABP1_MOUSE	sp|P62965|RABP1_MOUSE Cellular retinoic acid-binding protein 1 OS=Mus musculus GN=Crabp1 PE=1 SV=2	0.02817	-0.347
P34884	MIF_MOUSE	sp|P34884|MIF_MOUSE Macrophage migration inhibitory factor OS=Mus musculus GN=Mif PE=1 SV=2	0.03212	-0.355
Q8BGD9	IF4B_MOUSE	Eukaryotic translation initiation factor 4B OS=Mus musculus GN=Eif4b PE=1 SV=1	0.0233	-0.358
P17809	GTR1_MOUSE	Solute carrier family 2, facilitated glucose transporter member 1 OS=Mus musculus GN=Slc2a1 PE=1 SV=4	0.00869	-0.361
P57759	ERP29_MOUSE	sp|P57759|ERP29_MOUSE Endoplasmic reticulum resident protein 29 OS=Mus musculus GN=Erp29 PE=1 SV=2	0.04315	-0.362
Q99LD8	DDAH2_MOUSE	N(G),N(G)-dimethylarginine dimethylaminohydrolase 2 OS=Mus musculus GN=Ddah2 PE=1 SV=1	0.00091	-0.393
O09167	RL21_MOUSE	sp|O09167|RL21_MOUSE 60S ribosomal protein L21 OS=Mus musculus GN=Rpl21 PE=1 SV=3	0.00961	-0.398
P17918	PCNA_MOUSE	Proliferating cell nuclear antigen OS=Mus musculus GN=Pcna PE=1 SV=2	0.02495	-0.401
Q8VDJ3	VIGLN_MOUSE	sp|Q8VDJ3|VIGLN_MOUSE Vigilin OS=Mus musculus GN=Hdlbp PE=1 SV=1	0.00507	-0.429
Q9QUM9	PSA6_MOUSE	sp|Q9QUM9|PSA6_MOUSE Proteasome subunit alpha type-6 OS=Mus musculus GN=Psma6 PE=1 SV=1	0.00922	-0.480
O35188	X3CL1_MOUSE	sp|O35188|X3CL1_MOUSE Fractalkine OS=Mus musculus GN=Cx3cl1 PE=1 SV=3	0.00015	-0.564
