# Supplementary material for: Proteomic analysis links alterations of bioenergetics, mitochondria-ER interactions and proteostasis in hippocampal astrocytes from 3xTg-AD mice
Source: Cell Death Dis. 2020 Aug 18;11(8):645. doi: 10.1038/s41419-020-02911-1 (PMC7434916; doi:10.1038/s41419-020-02911-1)
Supplement: Supplementary file 4 — Supplemental Table 2 [file 41419_2020_2911_MOESM4_ESM.pdf]

Supplementary Table 2. DAVID GO analysis of merged lists of MERE fraction and whole-cell lysates from WT-iAstro and 3Tg-iAstro cells.					
**Category**	**Term**	**Count**	**Proteins**	**Fold Enrichment**	**Benjamini**
GOTERM_BP_DIRECT	GO:0006412~translation	23	IF4B_MOUSE, RL5_MOUSE, ADT1_MOUSE, RL24_MOUSE, RS15_MOUSE, IF4H_MOUSE, RL17_MOUSE, RL8_MOUSE, SYEP_MOUSE, RS17_MOUSE, RS18_MOUSE, EF1G_MOUSE, EF2_MOUSE, RL13_MOUSE, MCA3_MOUSE, RS8_MOUSE, MPCP_MOUSE, EF1D_MOUSE, RL21_MOUSE, RL7A_MOUSE, IF5A1_MOUSE, RL19_MOUSE, RS30_MOUSE	8.79	9.77E-12
GOTERM_BP_DIRECT	GO:0098609~cell-cell adhesion	10	EF1G_MOUSE, ALDOA_MOUSE, IF4H_MOUSE, CAPZB_MOUSE, FSCN1_MOUSE, ENOA_MOUSE, EF1D_MOUSE, 4F2_MOUSE, VIGLN_MOUSE, RL7A_MOUSE	8.11	0.001643
GOTERM_BP_DIRECT	GO:0006457~protein folding	8	TCPB_MOUSE, TCPG_MOUSE, PPIC_MOUSE, ENPL_MOUSE, PDIA3_MOUSE, ERP29_MOUSE, GRP75_MOUSE, TCPA_MOUSE	9.58	0.005577
GOTERM_BP_DIRECT	GO:0021762~substantia nigra development	5	ACTB_MOUSE, KCRB_MOUSE, CALM_MOUSE, GRP78_MOUSE, 1433E_MOUSE	20.16	0.021895
GOTERM_BP_DIRECT	GO:0008360~regulation of cell shape	7	GDIR1_MOUSE, ALDOA_MOUSE, FERM2_MOUSE, EZRI_MOUSE, FINC_MOUSE, DLG1_MOUSE, MYH10_MOUSE	7.72	0.047989

GOTERM_CC_DIRECT	GO:0070062~extracellular exosome	78	PSA6_MOUSE, ALDOA_MOUSE, NUCL_MOUSE, PEBP1_MOUSE, SERPH_MOUSE, ENPL_MOUSE, DLG1_MOUSE, 1433E_MOUSE, RS17_MOUSE, RS18_MOUSE, EF1G_MOUSE, LIN7C_MOUSE, RLA0_MOUSE, CAPZB_MOUSE, ACTB_MOUSE, MPCP_MOUSE, PCNA_MOUSE, GANAB_MOUSE, TBB6_MOUSE, ANXA3_MOUSE, RL7A_MOUSE, ACTG_MOUSE, RL5_MOUSE, IPYR_MOUSE, SDCB1_MOUSE, GTR1_MOUSE, CD81_MOUSE, GLRX3_MOUSE, SHLB1_MOUSE, GDIR1_MOUSE, MYO1D_MOUSE, TCPB_MOUSE, EF2_MOUSE, VCAM1_MOUSE, K22E_MOUSE, DDAH2_MOUSE, GRP75_MOUSE, VAMP5_MOUSE, KPYM_MOUSE, VDAC1_MOUSE, PRDX5_MOUSE, THY1_MOUSE, PUR4_MOUSE, HNRPM_MOUSE, FINC_MOUSE, FSCN1_MOUSE, ERP29_MOUSE, NSF_MOUSE, UB2V1_MOUSE, HNRPL_MOUSE, RS8_MOUSE, APMAP_MOUSE, S10A6_MOUSE, 4F2_MOUSE, GRP78_MOUSE, PDIA3_MOUSE, MYH10_MOUSE, IF5A1_MOUSE, RMXL1_MOUSE, ANXA6_MOUSE, TCPG_MOUSE, RL24_MOUSE, DEST_MOUSE, PPIC_MOUSE, ATPB_MOUSE, ENOA_MOUSE, CALM_MOUSE, TCPA_MOUSE, MFGM_MOUSE, MIF_MOUSE, MCA3_MOUSE, VATB2_MOUSE, EZRI_MOUSE, KCRB_MOUSE, K2C1B_MOUSE, TMEDA_MOUSE, MYADM_MOUSE, FLOT2_MOUSE	4.78	1.43E-35
GOTERM_CC_DIRECT	GO:0043209~myelin sheath	25	ACTG_MOUSE, TCPG_MOUSE, VDAC1_MOUSE, ADT1_MOUSE, THY1_MOUSE, ALDOA_MOUSE, PEBP1_MOUSE, ATPB_MOUSE, FSCN1_MOUSE, ENOA_MOUSE, NSF_MOUSE, GNAO_MOUSE, TCPA_MOUSE, MYO1D_MOUSE, TCPB_MOUSE, MIF_MOUSE, VATB2_MOUSE, EZRI_MOUSE, ACTB_MOUSE, MPCP_MOUSE, KCRB_MOUSE, GRP78_MOUSE, PDIA3_MOUSE, GRP75_MOUSE, KPYM_MOUSE	21.33	6.92E-23
GOTERM_CC_DIRECT	GO:0005925~focal adhesion	27	NPM_MOUSE, THY1_MOUSE, RL8_MOUSE, ENPL_MOUSE, 1433E_MOUSE, RS18_MOUSE, RS17_MOUSE, LIMS1_MOUSE, RLA0_MOUSE, FHL1_MOUSE, ACTB_MOUSE, RS8_MOUSE, GRP78_MOUSE, PDIA3_MOUSE, RL7A_MOUSE, ACTG_MOUSE, ANXA6_MOUSE, RL5_MOUSE, SDCB1_MOUSE, RS15_MOUSE, CD81_MOUSE, ITA5_MOUSE, FERM2_MOUSE, EZRI_MOUSE, GRP75_MOUSE, RL19_MOUSE, FLOT2_MOUSE	11.31	4.91E-18

GOTERM_CC_DIRECT	GO:0030529~intracellular ribonucleoprotein complex	23	RL5_MOUSE, NPM_MOUSE, RL24_MOUSE, NUCL_MOUSE, RS15_MOUSE, HNRPM_MOUSE, RL17_MOUSE, RL8_MOUSE, SYEP_MOUSE, RS17_MOUSE, RS18_MOUSE, HNRPU_MOUSE, RLA0_MOUSE, EF2_MOUSE, HNRPL_MOUSE, RL13_MOUSE, ACTB_MOUSE, RS8_MOUSE, XPO1_MOUSE, RL21_MOUSE, RL7A_MOUSE, RMXL1_MOUSE, RL19_MOUSE	11.78	1.79E-15
GOTERM_CC_DIRECT	GO:0005913~cell-cell adherens junction	22	ALDOA_MOUSE, RL24_MOUSE, SDCB1_MOUSE, RANG_MOUSE, IF4H_MOUSE, FSCN1_MOUSE, ENOA_MOUSE, DLG1_MOUSE, VIGLN_MOUSE, 1433E_MOUSE, SEPT9_MOUSE, EF1G_MOUSE, SHLB1_MOUSE, EF2_MOUSE, EZRI_MOUSE, CAPZB_MOUSE, EF1D_MOUSE, 4F2_MOUSE, GRP78_MOUSE, RL7A_MOUSE, KPYM_MOUSE, FLOT2_MOUSE	11.41	1.88E-14
GOTERM_CC_DIRECT	GO:0042470~melanosome	14	ANXA6_MOUSE, SC22B_MOUSE, VATB2_MOUSE, SDCB1_MOUSE, NP1L1_MOUSE, GTR1_MOUSE, GANAB_MOUSE, GRP78_MOUSE, ENPL_MOUSE, 4F2_MOUSE, PDIA3_MOUSE, 1433E_MOUSE, ERP29_MOUSE, TMEDA_MOUSE	22.71	1.83E-12
GOTERM_CC_DIRECT	GO:0005737~cytoplasm	80	PSA6_MOUSE, ALDOA_MOUSE, NUCL_MOUSE, PEBP1_MOUSE, NP1L1_MOUSE, SERPH_MOUSE, RL8_MOUSE, DLG1_MOUSE, VIGLN_MOUSE, 1433E_MOUSE, RS18_MOUSE, EF1G_MOUSE, LIN7C_MOUSE, RLA0_MOUSE, CAPZB_MOUSE, ACTB_MOUSE, PCNA_MOUSE, XPO1_MOUSE, RL21_MOUSE, RABP1_MOUSE, TBB6_MOUSE, ANXA3_MOUSE, RL7A_MOUSE, BTF3_MOUSE, ACTG_MOUSE, RL5_MOUSE, IPYR_MOUSE, SDCB1_MOUSE, GTR1_MOUSE, SYEP_MOUSE, GLRX3_MOUSE, ITA5_MOUSE, SHLB1_MOUSE, GDIR1_MOUSE, TCPB_MOUSE, HNRPU_MOUSE, EF2_MOUSE, FERM2_MOUSE, EF1D_MOUSE, DDAH2_MOUSE, GRP75_MOUSE, KPYM_MOUSE, NPM_MOUSE, PRDX5_MOUSE, S4A7_MOUSE, PUR4_MOUSE, FSCN1_MOUSE, X3CL1_MOUSE, NSF_MOUSE, UB2V1_MOUSE, HNRPL_MOUSE, FHL1_MOUSE, RS8_MOUSE, SRSF3_MOUSE, S10A6_MOUSE, 4F2_MOUSE, GRP78_MOUSE, PDIA3_MOUSE, MYH10_MOUSE, IF5A1_MOUSE, ANXA6_MOUSE, TCPG_MOUSE, RL24_MOUSE, MYEF2_MOUSE, DEST_MOUSE, RS15_MOUSE, PPIC_MOUSE, RANG_MOUSE, IF4H_MOUSE, DPYL3_MOUSE, ENOA_MOUSE, CALM_MOUSE, SEPT9_MOUSE, TCPA_MOUSE, MIF_MOUSE, MCA3_MOUSE, VATB2_MOUSE, EZRI_MOUSE, KCRB_MOUSE, RL19_MOUSE	1.98	1.26E-11

GOTERM_CC_DIRECT	GO:0005840~ribosome	14	RL5_MOUSE, RL24_MOUSE, RS15_MOUSE, RL17_MOUSE, RL8_MOUSE, RS17_MOUSE, RS18_MOUSE, RLA0_MOUSE, RL13_MOUSE, RS8_MOUSE, RL21_MOUSE, RL7A_MOUSE, RS30_MOUSE, RL19_MOUSE	12.20	4.19E-09
GOTERM_CC_DIRECT	GO:0031012~extracellular matrix	15	ACTG_MOUSE, HNRPM_MOUSE, ATPB_MOUSE, FINC_MOUSE, ENPL_MOUSE, RS18_MOUSE, RS17_MOUSE, MFGM_MOUSE, TCPB_MOUSE, EF2_MOUSE, HNRPU_MOUSE, GANAB_MOUSE, GRP78_MOUSE, GRP75_MOUSE, KPYM_MOUSE	8.36	9.32E-08
GOTERM_CC_DIRECT	GO:0016020~membrane	73	ALDOA_MOUSE, NUCL_MOUSE, NP1L1_MOUSE, RL8_MOUSE, ENPL_MOUSE, DLG1_MOUSE, 1433E_MOUSE, RS17_MOUSE, RS18_MOUSE, GNAO_MOUSE, SC22B_MOUSE, EF1G_MOUSE, LIN7C_MOUSE, RLA0_MOUSE, CAPZB_MOUSE, ACTB_MOUSE, MPCP_MOUSE, XPO1_MOUSE, GANAB_MOUSE, RL21_MOUSE, ANXA3_MOUSE, RL7A_MOUSE, ACTG_MOUSE, RL5_MOUSE, SDCB1_MOUSE, GTR1_MOUSE, SYEP_MOUSE, CD81_MOUSE, ITA5_MOUSE, SHLB1_MOUSE, GDIR1_MOUSE, HNRPU_MOUSE, EF2_MOUSE, RL13_MOUSE, FERM2_MOUSE, VCAM1_MOUSE, K22E_MOUSE, VAMP5_MOUSE, VDAC1_MOUSE, NPM_MOUSE, THY1_MOUSE, S4A7_MOUSE, HNRPM_MOUSE, OST48_MOUSE, ERP29_MOUSE, X3CL1_MOUSE, LIMS1_MOUSE, HNRPL_MOUSE, RS8_MOUSE, RILP_MOUSE, APMAP_MOUSE, CLCA_MOUSE, S10A6_MOUSE, DDX17_MOUSE, 4F2_MOUSE, GRP78_MOUSE, IF5A1_MOUSE, RMXL1_MOUSE, ANXA6_MOUSE, ADT1_MOUSE, RL24_MOUSE, RS15_MOUSE, IF4H_MOUSE, ATPB_MOUSE, ENOA_MOUSE, HMOX1_MOUSE, MFGM_MOUSE, VATB2_MOUSE, EZRI_MOUSE, RL19_MOUSE, TMEDA_MOUSE, MYADM_MOUSE, FLOT2_MOUSE	1.71	7.22E-07
GOTERM_CC_DIRECT	GO:0022625~cytosolic large ribosomal subunit	9	RL5_MOUSE, RLA0_MOUSE, RL24_MOUSE, RL13_MOUSE, RL17_MOUSE, RL8_MOUSE, RL21_MOUSE, RL7A_MOUSE, RL19_MOUSE	16.20	2.03E-06
GOTERM_CC_DIRECT	GO:0009986~cell surface	17	THY1_MOUSE, NUCL_MOUSE, PEBP1_MOUSE, HNRPM_MOUSE, ATPB_MOUSE, ERP29_MOUSE, X3CL1_MOUSE, ITA5_MOUSE, MIF_MOUSE, HNRPU_MOUSE, FERM2_MOUSE, VCAM1_MOUSE, APMAP_MOUSE, GRP78_MOUSE, 4F2_MOUSE, PDIA3_MOUSE, VAMP5_MOUSE	4.43	2.96E-05

GOTERM_CC_DIRECT	GO:0005829~cytosol	28	NPM_MOUSE, PSA6_MOUSE, THY1_MOUSE, PRDX5_MOUSE, FSCN1_MOUSE, ENPL_MOUSE, 1433E_MOUSE, DLG1_MOUSE, RS18_MOUSE, HNRPL_MOUSE, ACTB_MOUSE, RILP_MOUSE, S10A6_MOUSE, MYH10_MOUSE, RABP1_MOUSE, ACTG_MOUSE, ANXA6_MOUSE, SDCB1_MOUSE, GTR1_MOUSE, DPYL3_MOUSE, CALM_MOUSE, HMOX1_MOUSE, GDIR1_MOUSE, MYO1D_MOUSE, SHLB1_MOUSE, EF2_MOUSE, VATB2_MOUSE, EZRI_MOUSE	2.57	1.39E-04
GOTERM_CC_DIRECT	GO:0005634~nucleus	60	PSA6_MOUSE, ALDOA_MOUSE, NUCL_MOUSE, PEBP1_MOUSE, NP1L1_MOUSE, ENPL_MOUSE, DLG1_MOUSE, VIGLN_MOUSE, RS18_MOUSE, EF1G_MOUSE, RLA0_MOUSE, MPCP_MOUSE, PCNA_MOUSE, XPO1_MOUSE, TBB6_MOUSE, RL7A_MOUSE, SARNP_MOUSE, BTF3_MOUSE, ACTG_MOUSE, RL5_MOUSE, SDCB1_MOUSE, RL17_MOUSE, GLRX3_MOUSE, GDIR1_MOUSE, EF2_MOUSE, HNRPU_MOUSE, FERM2_MOUSE, RL13_MOUSE, K22E_MOUSE, EF1D_MOUSE, GRP75_MOUSE, KPYM_MOUSE, NPM_MOUSE, VDAC1_MOUSE, PRDX5_MOUSE, TIF1B_MOUSE, HNRPM_MOUSE, UB2V1_MOUSE, HNRPL_MOUSE, FHL1_MOUSE, RS8_MOUSE, SRSF3_MOUSE, DDX17_MOUSE, S10A6_MOUSE, GRP78_MOUSE, 4F2_MOUSE, PDIA3_MOUSE, MYH10_MOUSE, IF5A1_MOUSE, RMXL1_MOUSE, ANXA6_MOUSE, ADT1_MOUSE, MYEF2_MOUSE, RANG_MOUSE, ATPB_MOUSE, ENOA_MOUSE, CALM_MOUSE, HMOX1_MOUSE, MIF_MOUSE, MCA3_MOUSE	1.63	2.24E-04
GOTERM_CC_DIRECT	GO:0005790~smooth endoplasmic reticulum	5	MYO1D_MOUSE, GRP78_MOUSE, ENPL_MOUSE, PDIA3_MOUSE, ERP29_MOUSE	28.25	5.07E-04
GOTERM_CC_DIRECT	GO:0045121~membrane raft	10	EF2_MOUSE, VDAC1_MOUSE, THY1_MOUSE, SDCB1_MOUSE, EZRI_MOUSE, GTR1_MOUSE, SERPH_MOUSE, DLG1_MOUSE, FLOT2_MOUSE, MYADM_MOUSE	6.25	5.49E-04
GOTERM_CC_DIRECT	GO:0001725~stress fiber	6	FERM2_MOUSE, ACTB_MOUSE, MYL9_MOUSE, FSCN1_MOUSE, MYH10_MOUSE, SEPT9_MOUSE	14.67	8.86E-04
GOTERM_CC_DIRECT	GO:0005844~polysome	5	IF4B_MOUSE, EF2_MOUSE, PSA6_MOUSE, IF4H_MOUSE, RL17_MOUSE	19.05	0.001945

GOTERM_CC_DIRECT	GO:0005783~endoplasmic reticulum	21	THY1_MOUSE, SDCB1_MOUSE, TXD12_MOUSE, SERPH_MOUSE, OST48_MOUSE, ENPL_MOUSE, DLG1_MOUSE, ERP29_MOUSE, ITA5_MOUSE, HMOX1_MOUSE, SHLB1_MOUSE, EF1G_MOUSE, SC22B_MOUSE, VCAM1_MOUSE, APMAP_MOUSE, GANAB_MOUSE, EF1D_MOUSE, GRP78_MOUSE, PDIA3_MOUSE, IF5A1_MOUSE, TMEDA_MOUSE	2.60	0.001989
GOTERM_CC_DIRECT	GO:0022627~cytosolic small ribosomal subunit	5	RS15_MOUSE, RS8_MOUSE, RS18_MOUSE, RS17_MOUSE, RS30_MOUSE	15.46	0.003982
GOTERM_CC_DIRECT	GO:0048471~perinuclear region of cytoplasm	14	ANXA6_MOUSE, LIMS1_MOUSE, HNRPL_MOUSE, PRDX5_MOUSE, EZRI_MOUSE, IF4H_MOUSE, S10A6_MOUSE, ENPL_MOUSE, DLG1_MOUSE, X3CL1_MOUSE, VAMP5_MOUSE, SEPT9_MOUSE, HMOX1_MOUSE, FLOT2_MOUSE	3.31	0.004058
GOTERM_CC_DIRECT	GO:0044297~cell body	6	TCPB_MOUSE, TCPG_MOUSE, EZRI_MOUSE, DPYL3_MOUSE, GNAO_MOUSE, TCPA_MOUSE	9.64	0.004979
GOTERM_CC_DIRECT	GO:0005788~endoplasmic reticulum lumen	6	TXD12_MOUSE, SERPH_MOUSE, GRP78_MOUSE, ENPL_MOUSE, PDIA3_MOUSE, ERP29_MOUSE	9.45	0.005208
GOTERM_CC_DIRECT	GO:0005730~nucleolus	15	RL5_MOUSE, NPM_MOUSE, TIF1B_MOUSE, NUCL_MOUSE, RL8_MOUSE, HMOX1_MOUSE, EZRI_MOUSE, MPCP_MOUSE, DDX17_MOUSE, XPO1_MOUSE, EF1D_MOUSE, RL21_MOUSE, GRP75_MOUSE, RL7A_MOUSE, RL19_MOUSE	2.92	0.006693
GOTERM_CC_DIRECT	GO:0036464~cytoplasmic ribonucleoprotein granule	4	HNRPU_MOUSE, RLA0_MOUSE, NUCL_MOUSE, ACTB_MOUSE	23.41	0.007129
GOTERM_CC_DIRECT	GO:0015935~small ribosomal	4	NPM_MOUSE, RS15_MOUSE, RS18_MOUSE, RS30_MOUSE	22.60	0.007611
GOTERM_CC_DIRECT	GO:0005793~endoplasmic reticulum-Golgi intermediate compartment	5	SC22B_MOUSE, FINC_MOUSE, SERPH_MOUSE, GRP78_MOUSE, TMEDA_MOUSE	12.05	0.007733
GOTERM_CC_DIRECT	GO:0017101~aminoacyl-tRNA synthetase multienzyme complex	3	RL5_MOUSE, MCA3_MOUSE, SYEP_MOUSE	70.22	0.007783
GOTERM_CC_DIRECT	GO:0016324~apical plasma membrane	9	ANXA6_MOUSE, THY1_MOUSE, S4A7_MOUSE, EZRI_MOUSE, FINC_MOUSE, GTR1_MOUSE, 4F2_MOUSE, PDIA3_MOUSE, CD81_MOUSE	4.50	0.008418
GOTERM_CC_DIRECT	GO:0001772~immunological synapse	4	GDIR1_MOUSE, EZRI_MOUSE, DLG1_MOUSE, CD81_MOUSE	19.28	0.010553
GOTERM_CC_DIRECT	GO:0005902~microvillus	5	VATB2_MOUSE, EZRI_MOUSE, VCAM1_MOUSE, FSCN1_MOUSE, DLG1_MOUSE	10.78	0.010590
GOTERM_CC_DIRECT	GO:0005832~chaperonin-containing T-complex	3	TCPB_MOUSE, TCPG_MOUSE, TCPA_MOUSE	54.62	0.011152
GOTERM_CC_DIRECT	GO:0016323~basolateral plasma membrane	7	MYO1D_MOUSE, LIN7C_MOUSE, S4A7_MOUSE, EZRI_MOUSE, GTR1_MOUSE, DLG1_MOUSE, FLOT2_MOUSE	5.65	0.011900

GOTERM_CC_DIRECT	GO:0031941~filamentous actin	4	ACTG_MOUSE, FERM2_MOUSE, DPYL3_MOUSE, FSCN1_MOUSE	17.71	0.011926
GOTERM_CC_DIRECT	GO:0015629~actin cytoskeleton	7	ACTG_MOUSE, ALDOA_MOUSE, DEST_MOUSE, EZRI_MOUSE, FSCN1_MOUSE, MYH10_MOUSE, SEPT9_MOUSE	5.71	0.012000
GOTERM_CC_DIRECT	GO:0005911~cell-cell junction	7	LIMS1_MOUSE, LIN7C_MOUSE, GTR1_MOUSE, FSCN1_MOUSE, DLG1_MOUSE, ITA5_MOUSE, MYADM_MOUSE	5.71	0.012000
GOTERM_CC_DIRECT	GO:0005615~extracellular space	20	ACTG_MOUSE, ALDOA_MOUSE, PRDX5_MOUSE, SDCB1_MOUSE, PEBP1_MOUSE, DPYL3_MOUSE, FINC_MOUSE, SERPH_MOUSE, ENOA_MOUSE, X3CL1_MOUSE, GDIR1_MOUSE, MFGM_MOUSE, MIF_MOUSE, VCAM1_MOUSE, EZRI_MOUSE, ACTB_MOUSE, K22E_MOUSE, PDIA3_MOUSE, RMXL1_MOUSE, RS30_MOUSE	2.18	0.013250
GOTERM_CC_DIRECT	GO:0030864~cortical actin cytoskeleton	4	DEST_MOUSE, GTR1_MOUSE, FLOT2_MOUSE, MYADM_MOUSE	15.24	0.016917
GOTERM_CC_DIRECT	GO:0002199~zona pellucida receptor complex	3	TCPB_MOUSE, TCPG_MOUSE, TCPA_MOUSE	37.81	0.019447
GOTERM_CC_DIRECT	GO:0001726~ruffle	5	VATB2_MOUSE, EZRI_MOUSE, FSCN1_MOUSE, S10A6_MOUSE, MYADM_MOUSE	8.62	0.019558
GOTERM_CC_DIRECT	GO:0031982~vesicle	6	MIF_MOUSE, EZRI_MOUSE, GTR1_MOUSE, CD81_MOUSE, KPYM_MOUSE, FLOT2_MOUSE	5.99	0.022560
GOTERM_CC_DIRECT	GO:0043005~neuron projection	9	MYO1D_MOUSE, LIN7C_MOUSE, PEBP1_MOUSE, NP1L1_MOUSE, ENOA_MOUSE, CALM_MOUSE, DLG1_MOUSE, MYH10_MOUSE, GNAO_MOUSE	3.51	0.027522
GOTERM_CC_DIRECT	GO:0015934~large ribosomal subunit	3	NPM_MOUSE, RL17_MOUSE, RL8_MOUSE	27.31	0.034506
GOTERM_MF_DIRECT	GO:0044822~poly(A) RNA binding	46	IF4B_MOUSE, NPM_MOUSE, ALDOA_MOUSE, TIF1B_MOUSE, NUCL_MOUSE, HNRPM_MOUSE, PEBP1_MOUSE, NP1L1_MOUSE, SERPH_MOUSE, FSCN1_MOUSE, RL8_MOUSE, VIGLN_MOUSE, 1433E_MOUSE, RS17_MOUSE, RS18_MOUSE, RLA0_MOUSE, HNRPL_MOUSE, RS8_MOUSE, SRSF3_MOUSE, DDX17_MOUSE, GANAB_MOUSE, 4F2_MOUSE, RL21_MOUSE, PDIA3_MOUSE, RL7A_MOUSE, IF5A1_MOUSE, SARNP_MOUSE, BTF3_MOUSE, RS30_MOUSE, TCPG_MOUSE, RL5_MOUSE, RL24_MOUSE, MYEF2_MOUSE, RS15_MOUSE, IF4H_MOUSE, RL17_MOUSE, ENOA_MOUSE, GLRX3_MOUSE, TCPA_MOUSE, HNRPU_MOUSE, EF2_MOUSE, RL13_MOUSE, EZRI_MOUSE, GRP75_MOUSE, KPYM_MOUSE, RL19_MOUSE	6.06	1.85E-21

GOTERM_MF_DIRECT	GO:0098641~cadherin binding involved in cell-cell adhesion	21	ALDOA_MOUSE, RL24_MOUSE, SDCB1_MOUSE, RANG_MOUSE, IF4H_MOUSE, FSCN1_MOUSE, ENOA_MOUSE, DLG1_MOUSE, VIGLN_MOUSE, 1433E_MOUSE, SEPT9_MOUSE, EF1G_MOUSE, SHLB1_MOUSE, EF2_MOUSE, EZRI_MOUSE, CAPZB_MOUSE, EF1D_MOUSE, 4F2_MOUSE, GRP78_MOUSE, RL7A_MOUSE, KPYM_MOUSE	11.03	4.60E-13
GOTERM_MF_DIRECT	GO:0003723~RNA binding	24	IF4B_MOUSE, RL5_MOUSE, NPM_MOUSE, PSA6_MOUSE, NUCL_MOUSE, RS15_MOUSE, HNRPM_MOUSE, IF4H_MOUSE, ENOA_MOUSE, RL8_MOUSE, SYEP_MOUSE, ENPL_MOUSE, VIGLN_MOUSE, RS18_MOUSE, HNRPU_MOUSE, EF2_MOUSE, RL13_MOUSE, HNRPL_MOUSE, SRSF3_MOUSE, DDX17_MOUSE, XPO1_MOUSE, RL7A_MOUSE, IF5A1_MOUSE, RMXL1_MOUSE	4.51	2.11E-07
GOTERM_MF_DIRECT	GO:0003735~structural constituent of ribosome	15	RL5_MOUSE, ADT1_MOUSE, RL24_MOUSE, RS15_MOUSE, RL17_MOUSE, RL8_MOUSE, RS17_MOUSE, RS18_MOUSE, RLA0_MOUSE, RL13_MOUSE, RS8_MOUSE, MPCP_MOUSE, RL21_MOUSE, RS30_MOUSE, RL19_MOUSE	8.33	2.31E-07
GOTERM_MF_DIRECT	GO:0005515~protein binding	57	NUCL_MOUSE, SERPH_MOUSE, NP1L1_MOUSE, RL8_MOUSE, ENPL_MOUSE, DLG1_MOUSE, 1433E_MOUSE, GNAO_MOUSE, ACTB_MOUSE, CAPZB_MOUSE, PCNA_MOUSE, XPO1_MOUSE, GANAB_MOUSE, RL21_MOUSE, RL7A_MOUSE, SARNP_MOUSE, RS30_MOUSE, RL5_MOUSE, SDCB1_MOUSE, GTR1_MOUSE, RL17_MOUSE, CD81_MOUSE, GLRX3_MOUSE, ITA5_MOUSE, SHLB1_MOUSE, MYO1D_MOUSE, GDIR1_MOUSE, EF2_MOUSE, HNRPU_MOUSE, GRP75_MOUSE, KPYM_MOUSE, VDAC1_MOUSE, NPM_MOUSE, TIF1B_MOUSE, FINC_MOUSE, FSCN1_MOUSE, NSF_MOUSE, UB2V1_MOUSE, LIMS1_MOUSE, HNRPL_MOUSE, RILP_MOUSE, S10A6_MOUSE, GRP78_MOUSE, 4F2_MOUSE, PDIA3_MOUSE, MYH10_MOUSE, RMXL1_MOUSE, TCPG_MOUSE, ADT1_MOUSE, RS15_MOUSE, PPIC_MOUSE, DPYL3_MOUSE, ATPB_MOUSE, TCPA_MOUSE, MCA3_MOUSE, EZRI_MOUSE, KCRB_MOUSE	2.04	5.79E-07
GOTERM_MF_DIRECT	GO:0051082~unfolded protein binding	8	TCPB_MOUSE, TCPG_MOUSE, NPM_MOUSE, SERPH_MOUSE, GRP78_MOUSE, ENPL_MOUSE, GRP75_MOUSE, TCPA_MOUSE	15.64	3.76E-05

GOTERM_MF_DIRECT	GO:0000166~nucleotide binding	32	IF4B_MOUSE, VDAC1_MOUSE, PUR4_MOUSE, NUCL_MOUSE, HNRPM_MOUSE, PEBP1_MOUSE, ENPL_MOUSE, NSF_MOUSE, GNAO_MOUSE, HNRPL_MOUSE, ACTB_MOUSE, SRSF3_MOUSE, DDX17_MOUSE, GRP78_MOUSE, MYH10_MOUSE, TBB6_MOUSE, RMXL1_MOUSE, ACTG_MOUSE, TCPG_MOUSE, MYEF2_MOUSE, IF4H_MOUSE, ATPB_MOUSE, SYEP_MOUSE, SEPT9_MOUSE, TCPA_MOUSE, TCPB_MOUSE, MYO1D_MOUSE, EF2_MOUSE, HNRPU_MOUSE, KCRB_MOUSE, GRP75_MOUSE, KPYM_MOUSE	2.42	1.47E-04
GOTERM_MF_DIRECT	GO:0003746~translation elongation factor activity	5	EF1G_MOUSE, EF2_MOUSE, MCA3_MOUSE, EF1D_MOUSE, IF5A1_MOUSE	27.15	0.001187
GOTERM_MF_DIRECT	GO:0048306~calcium-dependent protein binding	6	ANXA6_MOUSE, MYO1D_MOUSE, HNRPM_MOUSE, S10A6_MOUSE, CALM_MOUSE, ANXA3_MOUSE	12.75	0.003522
GOTERM_MF_DIRECT	GO:0051015~actin filament binding	7	MYO1D_MOUSE, EF2_MOUSE, DEST_MOUSE, EZRI_MOUSE, CAPZB_MOUSE, FSCN1_MOUSE, MYH10_MOUSE	7.77	0.008436
GOTERM_MF_DIRECT	GO:0005178~integrin binding	6	MFGM_MOUSE, THY1_MOUSE, VCAM1_MOUSE, FINC_MOUSE, X3CL1_MOUSE, ITA5_MOUSE	8.80	0.016247
GOTERM_MF_DIRECT	GO:0019901~protein kinase binding	11	GDIR1_MOUSE, LIMS1_MOUSE, EF2_MOUSE, VDAC1_MOUSE, NPM_MOUSE, THY1_MOUSE, PEBP1_MOUSE, ACTB_MOUSE, DLG1_MOUSE, RS18_MOUSE, NSF_MOUSE	3.72	0.017089
GOTERM_MF_DIRECT	GO:0031625~ubiquitin protein ligase binding	9	ACTG_MOUSE, TCPB_MOUSE, TIF1B_MOUSE, KCRB_MOUSE, GRP78_MOUSE, 1433E_MOUSE, GRP75_MOUSE, UB2V1_MOUSE, TCPA_MOUSE	4.65	0.017678
INTERPRO	IPR012336:Thioredoxin-like fold	8	EF1G_MOUSE, MCA3_MOUSE, PRDX5_MOUSE, TXD12_MOUSE, SYEP_MOUSE, PDIA3_MOUSE, ERP29_MOUSE, GLRX3_MOUSE	10.18	0.004989
KEGG_PATHWAY	mmu03010:Ribosome	14	RL5_MOUSE, RL24_MOUSE, RS15_MOUSE, RL17_MOUSE, RL8_MOUSE, RS17_MOUSE, RS18_MOUSE, RLA0_MOUSE, RL13_MOUSE, RS8_MOUSE, RL21_MOUSE, RL7A_MOUSE, RS30_MOUSE, RL19_MOUSE	9.64	1.69E-07
SMART	SM00360:RRM	8	IF4B_MOUSE, HNRPL_MOUSE, MYEF2_MOUSE, NUCL_MOUSE, IF4H_MOUSE, HNRPM_MOUSE, SRSF3_MOUSE, RMXL1_MOUSE	5.52	0.039542

UP_KEYWORDS	Acetylation	79	PSA6_MOUSE, ALDOA_MOUSE, NUCL_MOUSE, PEBP1_MOUSE, NP1L1_MOUSE, SERPH_MOUSE, ABRAL_MOUSE, ENPL_MOUSE, VIGLN_MOUSE, 1433E_MOUSE, RS18_MOUSE, SC22B_MOUSE, EF1G_MOUSE, LIN7C_MOUSE, CAPZB_MOUSE, ACTB_MOUSE, MPCP_MOUSE, PCNA_MOUSE, XPO1_MOUSE, ANXA3_MOUSE, RL7A_MOUSE, SARNP_MOUSE, ACTG_MOUSE, RL5_MOUSE, IPYR_MOUSE, SDCB1_MOUSE, GTR1_MOUSE, SYEP_MOUSE, GLRX3_MOUSE, SHLB1_MOUSE, GDIR1_MOUSE, MYO1D_MOUSE, TCPB_MOUSE, MAOM_MOUSE, HNRPU_MOUSE, EF2_MOUSE, RL13_MOUSE, EF1D_MOUSE, GRP75_MOUSE, KPYM_MOUSE, IF4B_MOUSE, VDAC1_MOUSE, NPM_MOUSE, PRDX5_MOUSE, TIF1B_MOUSE, HNRPM_MOUSE, FSCN1_MOUSE, NSF_MOUSE, UB2V1_MOUSE, LIMS1_MOUSE, HNRPL_MOUSE, FHL1_MOUSE, RS8_MOUSE, SRSF3_MOUSE, APMAP_MOUSE, CLCA_MOUSE, MYL9_MOUSE, S10A6_MOUSE, GRP78_MOUSE, PDIA3_MOUSE, MYH10_MOUSE, IF5A1_MOUSE, RMXL1_MOUSE, ANXA6_MOUSE, TCPG_MOUSE, ADT1_MOUSE, RL24_MOUSE, DEST_MOUSE, RS15_MOUSE, RANG_MOUSE, IF4H_MOUSE, ATPB_MOUSE, ENOA_MOUSE, CALM_MOUSE, SEPT9_MOUSE, TCPA_MOUSE, MIF_MOUSE, MCA3_MOUSE, EZRI_MOUSE	4.74	5.76E-36

UP_KEYWORDS	Phosphoprotein	87	PSA6_MOUSE, ALDOA_MOUSE, NUCL_MOUSE, PEBP1_MOUSE, NP1L1_MOUSE, SERPH_MOUSE, ENPL_MOUSE, DLG1_MOUSE, VIGLN_MOUSE, 1433E_MOUSE, RS17_MOUSE, SC22B_MOUSE, RLA0_MOUSE, CAPZB_MOUSE, MPCP_MOUSE, PCNA_MOUSE, XPO1_MOUSE, GANAB_MOUSE, TBB6_MOUSE, ANXA3_MOUSE, SARNP_MOUSE, BTF3_MOUSE, RL5_MOUSE, IPYR_MOUSE, SDCB1_MOUSE, GTR1_MOUSE, SYEP_MOUSE, GLRX3_MOUSE, ITA5_MOUSE, SHLB1_MOUSE, GDIR1_MOUSE, TCPB_MOUSE, MYO1D_MOUSE, HNRPU_MOUSE, EF2_MOUSE, RL13_MOUSE, FERM2_MOUSE, K22E_MOUSE, EF1D_MOUSE, VAMP5_MOUSE, GRP75_MOUSE, KPYM_MOUSE, IF4B_MOUSE, VDAC1_MOUSE, NPM_MOUSE, PRDX5_MOUSE, TIF1B_MOUSE, S4A7_MOUSE, PUR4_MOUSE, HNRPM_MOUSE, FINC_MOUSE, FSCN1_MOUSE, ERP29_MOUSE, NSF_MOUSE, HNRPL_MOUSE, RS8_MOUSE, RILP_MOUSE, SRSF3_MOUSE, APMAP_MOUSE, MYL9_MOUSE, CLCA_MOUSE, S10A6_MOUSE, DDX17_MOUSE, 4F2_MOUSE, GRP78_MOUSE, PDIA3_MOUSE, MYH10_MOUSE, RMXL1_MOUSE, ANXA6_MOUSE, TCPG_MOUSE, ADT1_MOUSE, RL24_MOUSE, MYEF2_MOUSE, DEST_MOUSE, RANG_MOUSE, IF4H_MOUSE, ATPB_MOUSE, DPYL3_MOUSE, ENOA_MOUSE, CALM_MOUSE, SEPT9_MOUSE, HMOX1_MOUSE, TCPA_MOUSE, EZRI_MOUSE, KCRB_MOUSE, RL19_MOUSE, FLOT2_MOUSE	2.14	1.18E-15
UP_KEYWORDS	Isopeptide bond	31	NPM_MOUSE, VDAC1_MOUSE, PSA6_MOUSE, ALDOA_MOUSE, TIF1B_MOUSE, NUCL_MOUSE, HNRPM_MOUSE, FINC_MOUSE, NP1L1_MOUSE, FSCN1_MOUSE, RS17_MOUSE, EF1G_MOUSE, RLA0_MOUSE, HNRPL_MOUSE, FHL1_MOUSE, PCNA_MOUSE, DDX17_MOUSE, 4F2_MOUSE, GRP78_MOUSE, TBB6_MOUSE, RMXL1_MOUSE, RL5_MOUSE, TCPG_MOUSE, ADT1_MOUSE, CALM_MOUSE, GDIR1_MOUSE, EF2_MOUSE, HNRPU_MOUSE, RL13_MOUSE, KPYM_MOUSE, RL19_MOUSE	6.10	1.00E-13
UP_KEYWORDS	Ribonucleoprotein	18	RL5_MOUSE, RL24_MOUSE, RS15_MOUSE, HNRPM_MOUSE, RL17_MOUSE, RL8_MOUSE, RS17_MOUSE, RS18_MOUSE, HNRPU_MOUSE, RLA0_MOUSE, HNRPL_MOUSE, RL13_MOUSE, RS8_MOUSE, RL21_MOUSE, RL7A_MOUSE, RMXL1_MOUSE, RL19_MOUSE, RS30_MOUSE	10.95	3.04E-11

UP_KEYWORDS	Methylation	26	NUCL_MOUSE, HNRPM_MOUSE, NP1L1_MOUSE, HNRPL_MOUSE, ACTB_MOUSE, MPCP_MOUSE, DDX17_MOUSE, PCNA_MOUSE, GRP78_MOUSE, PDIA3_MOUSE, MYH10_MOUSE, BTF3_MOUSE, RMXL1_MOUSE, ACTG_MOUSE, MYEF2_MOUSE, IF4H_MOUSE, DPYL3_MOUSE, SYEP_MOUSE, CALM_MOUSE, EF2_MOUSE, HNRPU_MOUSE, K2C1B_MOUSE, K22E_MOUSE, GRP75_MOUSE, KPYM_MOUSE, TMEDA_MOUSE	5.08	1.18E-09
UP_KEYWORDS	Ribosomal protein	14	RL5_MOUSE, RL24_MOUSE, RS15_MOUSE, RL17_MOUSE, RL8_MOUSE, RS17_MOUSE, RS18_MOUSE, RLA0_MOUSE, RL13_MOUSE, RS8_MOUSE, RL21_MOUSE, RL7A_MOUSE, RS30_MOUSE, RL19_MOUSE	12.93	1.96E-09
UP_KEYWORDS	Cytoplasm	54	PSA6_MOUSE, ALDOA_MOUSE, NUCL_MOUSE, PEBP1_MOUSE, RL8_MOUSE, 1433E_MOUSE, VIGLN_MOUSE, RS18_MOUSE, RLA0_MOUSE, ACTB_MOUSE, CAPZB_MOUSE, XPO1_MOUSE, RABP1_MOUSE, TBB6_MOUSE, BTF3_MOUSE, ACTG_MOUSE, IPYR_MOUSE, RL5_MOUSE, SDCB1_MOUSE, GLRX3_MOUSE, SHLB1_MOUSE, TCPB_MOUSE, GDIR1_MOUSE, EF2_MOUSE, HNRPU_MOUSE, FERM2_MOUSE, DDAH2_MOUSE, KPYM_MOUSE, NPM_MOUSE, PRDX5_MOUSE, PUR4_MOUSE, FSCN1_MOUSE, NSF_MOUSE, HNRPL_MOUSE, FHL1_MOUSE, RS8_MOUSE, SRSF3_MOUSE, CLCA_MOUSE, S10A6_MOUSE, GRP78_MOUSE, IF5A1_MOUSE, ANXA6_MOUSE, TCPG_MOUSE, IF4H_MOUSE, PPIC_MOUSE, DPYL3_MOUSE, ENOA_MOUSE, CALM_MOUSE, SEPT9_MOUSE, TCPA_MOUSE, MIF_MOUSE, MCA3_MOUSE, EZRI_MOUSE, KCRB_MOUSE	2.30	1.46E-08
UP_KEYWORDS	RNA-binding	19	IF4B_MOUSE, RL5_MOUSE, NPM_MOUSE, MYEF2_MOUSE, NUCL_MOUSE, HNRPM_MOUSE, IF4H_MOUSE, RL8_MOUSE, SYEP_MOUSE, VIGLN_MOUSE, RS18_MOUSE, HNRPU_MOUSE, HNRPL_MOUSE, SRSF3_MOUSE, DDX17_MOUSE, XPO1_MOUSE, IF5A1_MOUSE, RMXL1_MOUSE, SARNP_MOUSE	5.93	6.87E-08

UP_KEYWORDS	Ubl conjugation	29	NPM_MOUSE, VDAC1_MOUSE, ALDOA_MOUSE, TIF1B_MOUSE, NUCL_MOUSE, HNRPM_MOUSE, FSCN1_MOUSE, RS17_MOUSE, EF1G_MOUSE, RLA0_MOUSE, HNRPL_MOUSE, FHL1_MOUSE, ACTB_MOUSE, PCNA_MOUSE, DDX17_MOUSE, 4F2_MOUSE, GRP78_MOUSE, RMXL1_MOUSE, RL5_MOUSE, TCPG_MOUSE, DEST_MOUSE, ENOA_MOUSE, CALM_MOUSE, GDIR1_MOUSE, EF2_MOUSE, HNRPU_MOUSE, RL13_MOUSE, KPYM_MOUSE, RL19_MOUSE	3.61	7.88E-08
UP_KEYWORDS	Elongation factor	5	EF1G_MOUSE, EF2_MOUSE, MCA3_MOUSE, EF1D_MOUSE, IF5A1_MOUSE	40.75	1.18E-04
UP_KEYWORDS	Protein biosynthesis	8	IF4B_MOUSE, EF1G_MOUSE, EF2_MOUSE, MCA3_MOUSE, IF4H_MOUSE, EF1D_MOUSE, SYEP_MOUSE, IF5A1_MOUSE	10.20	2.36E-04
UP_KEYWORDS	Nucleotide-binding	24	ACTG_MOUSE, TCPG_MOUSE, VDAC1_MOUSE, PUR4_MOUSE, PEBP1_MOUSE, ATPB_MOUSE, SYEP_MOUSE, ENPL_MOUSE, SEPT9_MOUSE, NSF_MOUSE, GNAO_MOUSE, TCPA_MOUSE, MYO1D_MOUSE, TCPB_MOUSE, HNRPU_MOUSE, EF2_MOUSE, ACTB_MOUSE, KCRB_MOUSE, DDX17_MOUSE, GRP78_MOUSE, MYH10_MOUSE, TBB6_MOUSE, GRP75_MOUSE, KPYM_MOUSE	2.56	6.73E-04
UP_KEYWORDS	Chaperone	8	TCPB_MOUSE, TCPG_MOUSE, NPM_MOUSE, SERPH_MOUSE, ENPL_MOUSE, GRP75_MOUSE, BTF3_MOUSE, TCPA_MOUSE	7.85	0.001065
UP_KEYWORDS	ATP-binding	19	ACTG_MOUSE, TCPG_MOUSE, PUR4_MOUSE, PEBP1_MOUSE, ATPB_MOUSE, SYEP_MOUSE, ENPL_MOUSE, NSF_MOUSE, TCPA_MOUSE, MYO1D_MOUSE, TCPB_MOUSE, HNRPU_MOUSE, ACTB_MOUSE, KCRB_MOUSE, DDX17_MOUSE, GRP78_MOUSE, MYH10_MOUSE, GRP75_MOUSE, KPYM_MOUSE	2.61	0.004037
UP_KEYWORDS	Cell shape	4	FERM2_MOUSE, EZRI_MOUSE, FINC_MOUSE, MYH10_MOUSE	28.84	0.004412
UP_KEYWORDS	Endoplasmic reticulum	14	SDCB1_MOUSE, TXD12_MOUSE, SERPH_MOUSE, OST48_MOUSE, ENPL_MOUSE, DLG1_MOUSE, ERP29_MOUSE, HMOX1_MOUSE, SC22B_MOUSE, GANAB_MOUSE, GRP78_MOUSE, PDIA3_MOUSE, IF5A1_MOUSE, TMEDA_MOUSE	2.63	0.028568
UP_SEQ_FEATURE	short sequence motif:Prevents secretion from ER	6	TXD12_MOUSE, SERPH_MOUSE, GRP78_MOUSE, ENPL_MOUSE, PDIA3_MOUSE, ERP29_MOUSE	14.76	0.021932
