# Supplementary material for: Proteomic analysis links alterations of bioenergetics, mitochondria-ER interactions and proteostasis in hippocampal astrocytes from 3xTg-AD mice
Source: Cell Death Dis. 2020 Aug 18;11(8):645. doi: 10.1038/s41419-020-02911-1 (PMC7434916; doi:10.1038/s41419-020-02911-1)
Supplement: Supplementary file 5 — Supplemental Table 3a [file 41419_2020_2911_MOESM5_ESM.pdf]

Supplementary Table 3a. DEPs of Volgyi et al., 2018 dataset.					
**Uniprot_ID**	**Uniprot_KB**	**Gene name**	**Description**	**FC**	***p* -value**
RS21_MOUSE	Q9CQR2	Rps21	40S ribosomal protein S21	2.988	1.19E-05
MIC13_MOUSE	Q8R404	Mic13	MICOS complex subunit MIC13	2.66	5.25E-03
TMED2_MOUSE	Q9R0Q3	Tmed2	Transmembrane emp24 domain-containing protein 2	2.601	1.13E-03
HNRPD_MOUSE	Q60668	Hnrnpd	Isoform 2 of Heterogeneous nuclear ribonucleoprotein D0	2.368	1.30E-02
A4_MOUSE	P12023	App	Isoform APP695 chimeric mouse human amyloid precursor protein	2.304	4.16E-08
SDF2_MOUSE	Q9DCT5	Sdf2	Stromal cell-derived factor 2	2.246	5.70E-03
ESYT1_MOUSE	Q3U7R1	Esyt1	Extended synaptotagmin-1	2.177	3.64E-03
HMGB1_MOUSE	P63158	Hmgb1	High mobility group protein B1	2.136	2.21E-03
TPC12_MOUSE	Q8K2L8	Trappc12	Trafficking protein particle complex subunit 12	2.077	1.26E-02
EMC10_MOUSE	Q3TAS6	Emc10	Isoform 2 of ER membrane protein complex subunit 10	2.04	1.40E-02
RL5_MOUSE	P47962	Rpl5	60S ribosomal protein L5	2.024	1.47E-05
SND1_MOUSE	Q78PY7	Snd1	Staphylococcal nuclease domain-containing protein 1	1.927	2.79E-03
SE1L1_MOUSE	Q9Z2G6	Sel1l	Protein sel-1 homolog 1	1.887	2.72E-02
RHOA_MOUSE	Q9QUI0	Rhoa	Transforming protein RhoA	1.867	1.88E-02
RS24_MOUSE	P62849	Rps24	Isoform 2 of 40S ribosomal protein S24	1.852	1.38E-03
FUND1_MOUSE	Q9DB70	Fundc1	FUN14 domain-containing protein 1	1.812	1.58E-02
TMED9_MOUSE	Q99KF1	Tmed9	Transmembrane emp24 domain-containing protein 9	1.771	3.70E-03
RACK1_MOUSE	P68040	Rack1	Guanine nucleotide-binding protein subunit beta-2-like 1	1.766	8.66E-04
VPS25_MOUSE	Q9CQ80	Vps25	Vacuolar protein-sorting-associated protein 25	1.732	2.34E-02
SURF4_MOUSE	Q64310	Surf4	Surfeit locus protein 4	1.708	2.11E-02
AMACR_MOUSE	O09174	Amacr	Alpha-methylacyl-CoA racemase	1.687	2.59E-02
RS14_MOUSE	P62264	Rps14	40S ribosomal protein S14	1.681	3.80E-02
RL17_MOUSE	Q9CPR4	Rpl17	60S ribosomal protein L17	1.681	2.45E-03
AN32A_MOUSE	O35381	Anp32a	Acidic leucine-rich nuclear phosphoprotein 32 family member A	1.676	7.94E-03
RL3_MOUSE	P27659	Rpl3	60S ribosomal protein L3	1.653	1.30E-02
ERMP1_MOUSE	Q3UVK0	Ermp1	Endoplasmic reticulum metallopeptidase 1	1.651	4.14E-02
RM39_MOUSE	Q9JKF7	Mrpl39	39S ribosomal protein L39, mitochondrial	1.634	3.44E-02
MPV17_MOUSE	P19258	Mpv17	Protein Mpv17	1.633	1.96E-02
NFH_MOUSE	P19246	Nefh	Neurofilament heavy polypeptide	1.632	5.09E-03
RS12_MOUSE	P63323	Rps12	40S ribosomal protein S12	1.626	1.75E-02
RS16_MOUSE	P14131	Rps16	40S ribosomal protein S16	1.584	3.91E-04
RL9_MOUSE	P51410	Rpl9	60S ribosomal protein L9	1.563	7.93E-04
RS15A_MOUSE	P62245	Rps15a	40S ribosomal protein S15a	1.555	7.81E-03
SYYM_MOUSE	Q8BYL4	Yars2	Tyrosine--tRNA ligase, mitochondrial	1.539	2.37E-02
RS9_MOUSE	Q6ZWN5	Rps9	40S ribosomal protein S9	1.535	3.64E-03
SYJ2B_MOUSE	Q9D6K5	Synj2bp	Isoform 2 of Synaptojanin-2-binding protein	1.527	3.79E-02

EF1B_MOUSE	O70251	Eef1b	Elongation factor 1-beta	1.495	3.31E-02
LYRM4_MOUSE	Q8K215	Lyrm4	LYR motif-containing protein 4	1.484	4.00E-02
RS13_MOUSE	P62301	Rps13	40S ribosomal protein S13	1.476	1.81E-04
SEPT4_MOUSE	P28661	Sept4	Isoform 2 of Septin-4	1.475	9.71E-03
RS5_MOUSE	P97461	Rps5	40S ribosomal protein S5	1.474	1.58E-02
RS7_MOUSE	P62082	Rps7	40S ribosomal protein S7	1.466	2.65E-02
NOL3_MOUSE	Q9D1X0	Nol3 Arc	Nucleolar protein 3	1.463	2.14E-04
HS71B_MOUSE	P17879	Hspa1b	Heat shock 70 kDa protein 1B	1.462	1.93E-02
RS3_MOUSE	P62908	Rps3	40S ribosomal protein S3	1.46	3.18E-03
CMPK2_MOUSE	Q3U5Q7	Cmpk2	UMP-CMP kinase 2, mitochondrial	1.451	6.42E-03
RSSA_MOUSE	P14206	Rpsa	40S ribosomal protein SA	1.451	3.68E-03
RS11_MOUSE	P62281	Rps11	40S ribosomal protein S11	1.449	9.69E-03
CC136_MOUSE	Q3TVA9	Ccdc136	Isoform 2 of Coiled-coil domain-containing protein 136	1.447	2.38E-02
RL21_MOUSE	O09167	Rpl21	60S ribosomal protein L21	1.447	7.16E-03
RS2_MOUSE	P25444	Rps2	40S ribosomal protein S2	1.443	1.80E-02
RL27_MOUSE	P61358	Rpl27	60S ribosomal protein L27	1.422	2.83E-02
CSDE1_MOUSE	Q91W50	Csde1	Cold shock domain-containing protein E1	1.408	2.88E-05
FPPS_MOUSE	Q920E5	Fdps	Farnesyl pyrophosphate synthase	1.405	4.68E-02
RL34_MOUSE	Q9D1R9	Rpl34	60S ribosomal protein L34	1.405	2.23E-02
SDCB1_MOUSE	O08992	Sdcbp	Syntenin-1	1.405	2.10E-02
RL10_MOUSE	Q6ZWV3	Rpl10	60S ribosomal protein L10	1.402	2.25E-02
RS25_MOUSE	P62852	Rps25	40S ribosomal protein S25	1.396	4.05E-02
NUCB1_MOUSE	Q02819	Nucb1	Nucleobindin-1	1.383	1.15E-02
RL14_MOUSE	Q9CR57	Rpl14	60S ribosomal protein L14	1.379	5.78E-03
MPPA_MOUSE	Q9DC61	Pmpca	Mitochondrial-processing peptidase subunit alpha	1.362	1.23E-02
RAB35_MOUSE	Q6PHN9	Rab35	Ras-related protein Rab-35	1.356	1.92E-02
RL35_MOUSE	Q6ZWV7	Rpl35	60S ribosomal protein L35	1.344	3.10E-02
RS10_MOUSE	P63325	Rps10	40S ribosomal protein S10	1.344	2.14E-02
COMT_MOUSE	O88587	Comt	Isoform Soluble of Catechol O-methyltransferase	1.333	2.91E-02
RS23_MOUSE	P62267	Rps23	40S ribosomal protein S23	1.309	5.24E-03
HNRPQ_MOUSE	Q7TMK9	Syncrip	Isoform 2 of Heterogeneous nuclear ribonucleoprotein Q	1.308	1.42E-02
NPL4_MOUSE	P60670	Nploc4	Nuclear protein localization protein 4 homolog	1.305	4.47E-02
CP46A_MOUSE	Q9WVK8	Cyp46a1	Cholesterol 24-hydroxylase	1.299	5.59E-03
FKBP8_MOUSE	O35465	Fkbp8	Isoform 2 of Peptidyl-prolyl cis-trans isomerase FKBP8	1.294	2.49E-02
ABCF2_MOUSE	Q99LE6	Abcf2	ATP-binding cassette sub-family F member 2	1.291	3.37E-02
REEP2_MOUSE	Q8VCD6	Reep2	Receptor expression-enhancing protein 2	1.288	2.52E-02
RS20_MOUSE	P60867	Rps20	40S ribosomal protein S20	1.285	3.76E-02
ACO13_MOUSE	Q9CQR4	Acot13	Acyl-coenzyme A thioesterase 13	1.283	4.47E-02
MAOX_MOUSE	P06801	Me1	NADP-dependent malic enzyme	1.255	7.04E-04

RAB1A_MOUSE	P62821	Rab1A	Ras-related protein Rab-1A	1.2	4.78E-02
RASK_MOUSE	P32883	Kras	Isoform 2B of GTPase KRas	1.199	2.17E-02
PDK3_MOUSE	Q922H2	Pdk3	[Pyruvate dehydrogenase (acetyl-transferring)] kinase isozyme 3, mitochondrial	-1.196	4.23E-02
HEMH_MOUSE	P22315	Fech	Ferrochelatase, mitochondrial	-1.232	3.86E-02
TPC_MOUSE	Q9DAM5	Slc25a19	Mitochondrial thiamine pyrophosphate carrier	-1.255	4.67E-02
MPC2_MOUSE	Q9D023	Mpc2	Mitochondrial pyruvate carrier 2	-1.274	3.94E-02
EHD4_MOUSE	Q9EQP2	Ehd4	EH domain-containing protein 4	-1.275	3.74E-03
MCAT_MOUSE	Q9Z2Z6	Slc25a20	Mitochondrial carnitine/acylcarnitine carrier protein	-1.276	2.72E-02
TOM22_MOUSE	Q9CPQ3	Tomm22	Mitochondrial import receptor subunit TOM22 homolog	-1.285	7.53E-03
P5CS_MOUSE	Q9Z110	Aldh18a1	Delta-1-pyrroline-5-carboxylate synthase	-1.29	2.03E-02
TDRKH_MOUSE	Q80VL1	Tdrkh	Tudor and KH domain-containing protein	-1.297	2.15E-02
UBQL1_MOUSE	Q8R317	Ubqln1	Isoform 2 of Ubiquilin-1	-1.297	1.02E-02
TIGAR_MOUSE	Q8BZA9	Tigar	Fructose-2,6-bisphosphatase TIGAR	-1.303	3.95E-02
VWA8_MOUSE	Q8CC88	Vwa8	von Willebrand factor A domain-containing protein 8	-1.306	4.37E-03
PUR8_MOUSE	P54822	Adsl	Adenylosuccinate lyase	-1.312	4.03E-02
RYR1_MOUSE	E9PZQ0	Ryr1	Ryanodine receptor 1	-1.313	3.75E-02
FBX6_MOUSE	Q9QZN4	Fbxo6	F-box only protein 6	-1.334	1.75E-02
NDUS3_MOUSE	Q9DCT2	Ndufs3	NADH dehydrogenase [ubiquinone] iron-sulfur protein 3, mitochondrial	-1.334	8.16E-03
CHCH2_MOUSE	Q9D1L0	Chchd2	Coiled-coil-helix-coiled-coil-helix domain-containing protein 2	-1.336	1.65E-02
ATAD1_MOUSE	Q9D5T0	Atad1	ATPase family AAA domain-containing protein 1	-1.355	3.29E-02
MTCH1_MOUSE	Q791T5	Mtch1	Isoform 2 of Mitochondrial carrier homolog 1	-1.381	5.07E-04
COASY_MOUSE	Q9DBL7	Coasy	Bifunctional coenzyme A synthase	-1.396	3.41E-02
NDUS8_MOUSE	Q8K3J1	Ndufs8	NADH dehydrogenase [ubiquinone] iron-sulfur protein 8, mitochondrial	-1.405	4.07E-03
RM19_MOUSE	Q9D338	Mrpl19	39S ribosomal protein L19, mitochondrial	-1.407	3.41E-02
MK08_MOUSE	Q91Y86	Mapk8	Mitogen-activated protein kinase 8	-1.416	1.05E-02
ODBA_MOUSE	P50136	Bckdha	2-oxoisovalerate dehydrogenase subunit alpha, mitochondrial	-1.429	3.40E-02
RM01_MOUSE	Q99N96	Mrpl1	39S ribosomal protein L1, mitochondrial	-1.431	2.43E-02
DHSD_MOUSE	Q9CXV1	Sdhd	Succinate dehydrogenase [ubiquinone] cytochrome b small subunit, mitochondrial	-1.436	4.54E-03
SC31A_MOUSE	Q3UPL0	Sec31a	Isoform 2 of Protein transport protein Sec31A	-1.438	1.18E-02
RT30_MOUSE	Q9D0G0	Mrps30	28S ribosomal protein S30, mitochondrial	-1.463	3.23E-02
EFGM_MOUSE	Q8K0D5	Gfm1	Elongation factor G, mitochondrial	-1.489	1.01E-02
MINP1_MOUSE	Q9Z2L6	Minpp1	Multiple inositol polyphosphate phosphatase 1	-1.548	4.50E-02
TIDC1_MOUSE	Q8BUY5	Timmdc1	Complex I assembly factor TIMMDC1, mitochondrial	-1.554	3.66E-02
RMND1_MOUSE	Q8CI78	Rmnd1	Required for meiotic nuclear division protein 1 homolog	-1.558	1.82E-02
SYTM_MOUSE	Q3UQ84	Tars2	Threonine--tRNA ligase, mitochondrial	-1.562	2.82E-02
PPTC7_MOUSE	Q6NVE9	Pptc7	Protein phosphatase PTC7 homolog	-1.571	6.28E-03
MCTS1_MOUSE	Q9DB27	Mcts1	Malignant T-cell-amplified sequence 1	-1.586	2.54E-04
MK10_MOUSE	Q61831	Mapk10	Isoform Alpha-1 of Mitogen-activated protein kinase 10	-1.591	1.58E-03
NDUB2_MOUSE	Q9CPU2	Ndufb2	NADH dehydrogenase [ubiquinone] 1 beta subcomplex subunit 2, mitochondrial	-1.593	5.32E-03

RM38_MOUSE	Q8K2M0	Mrpl38	39S ribosomal protein L38, mitochondrial	-1.7	1.72E-03
S2536_MOUSE	Q922G0	Slc25a36	Solute carrier family 25 member 36	-1.773	1.37E-02
RM21_MOUSE	Q9D1N9	Mrpl21	39S ribosomal protein L21, mitochondrial	-1.894	6.54E-03
NMDZ1_MOUSE	P35438	Grin1	Glutamate receptor ionotropic, NMDA 1	-2.075	4.68E-02
PACS2_MOUSE	Q3V3Q7	Pacs2	Phosphofurin acidic cluster sorting protein 2	-2.081	1.96E-02
CMC2_MOUSE	Q9QXX4	Slc25a13	Calcium-binding mitochondrial carrier protein Aralar2	-2.232	1.26E-02
TIM16_MOUSE	Q9CQV1	Pam16	Mitochondrial import inner membrane translocase subunit TIM16	-2.289	4.04E-02
TI17B_MOUSE	Q9Z0V7	Timm17b	Mitochondrial import inner membrane translocase subunit Tim17-B	-2.42	2.64E-04
RM49_MOUSE	Q9CQ40	Mrpl49	39S ribosomal protein L49, mitochondrial	-2.453	5.60E-04
RM40_MOUSE	Q9Z2Q5	Mrpl40	39S ribosomal protein L40, mitochondrial	-2.512	6.52E-04
PTCD3_MOUSE	Q14C51	Ptcd3	Pentatricopeptide repeat domain-containing protein 3, mitochondrial	-3.421	5.11E-04
ABCG1_MOUSE	Q64343	Abcg1	ATP-binding cassette sub-family G member 1	-6.365	3.96E-03
