# Supplementary material for: Proteomic analysis links alterations of bioenergetics, mitochondria-ER interactions and proteostasis in hippocampal astrocytes from 3xTg-AD mice
Source: Cell Death Dis. 2020 Aug 18;11(8):645. doi: 10.1038/s41419-020-02911-1 (PMC7434916; doi:10.1038/s41419-020-02911-1)
Supplement: Supplementary file 6 — Supplemental Table 3b [file 41419_2020_2911_MOESM6_ESM.pdf]

Supplementary Table 3b. Comparison of MERE fraction DEPs with DEPs of Volgyi et al., 2018 dataset.					
**Uniprot_ID**	**Protein name (Volgyi)**	**FC (Volgyi)**	***p*-v. (Volgyi)**	***p*-v. (MERE fr.)**	**FC (MERE fr.)**
RL21_MOUSE	60S ribosomal protein L21	1.447	0.00716	0.00961	-2.499
SDCB1_MOUSE	Syntenin-1	1.405	0.021	0.03294	1.873

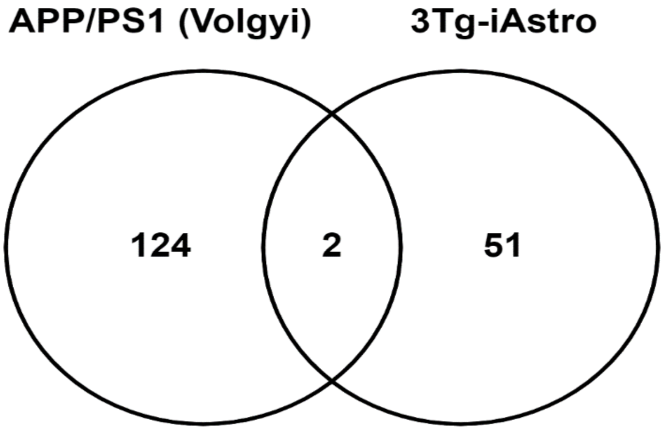

R command:: phyper(k-1, K, N-K, n, lower.tail=FALSE)

**Hypergeometric Te**	Volgyi ident.	Volgy quant.	MERE fraction ident. in WT
N	5957	3245	1089
K	126		
n	53		
k	2		
***p*-value**	**0.309291**	**0.617201**	**0.989627**
	0.309290686	0.617201298	0.9896267
**Expected value**	**1.121034078**	**2.057935285**	**6.1322314**
n*(K/N)			
