# Supplementary material for: Proteomic analysis links alterations of bioenergetics, mitochondria-ER interactions and proteostasis in hippocampal astrocytes from 3xTg-AD mice
Source: Cell Death Dis. 2020 Aug 18;11(8):645. doi: 10.1038/s41419-020-02911-1 (PMC7434916; doi:10.1038/s41419-020-02911-1)
Supplement: Supplementary file 7 — Supplemental Table 3c [file 41419_2020_2911_MOESM7_ESM.pdf]

Supplementary Table 3c. DEPs of Yu et al., 2018 dataset.			
**Uniprot_ID**	**Uniprot_KB**	**Gene name**	**Description**
**Hippocampal Mitochondrial Proteins**			
NDUV1_MOUSE	Q91YT0	Ndufv1	NADH dehydrogenase [ubiquinone] flavoprotein 1, mitochondrial (EC 7.1.1.2) (Complex I-51kD) (CI-51kD) (NADH-ubiquinone oxidoreductase 51 kDa subunit)
ATPA_MOUSE	Q03265	Atp5f1a Atp5a1	ATP synthase subunit alpha, mitochondrial (ATP synthase F1 subunit alpha)
ATP5H_MOUSE	Q9DCX2	Atp5pd Atp5h	ATP synthase subunit d, mitochondrial (ATPase subunit d) (ATP synthase peripheral stalk subunit d)
COX5A_MOUSE	P12787	Cox5a	Cytochrome c oxidase subunit 5A, mitochondrial (Cytochrome c oxidase polypeptide Va)
BLVRB_MOUSE	Q923D2	Blvrb	Flavin reductase (NADPH) (FR) (EC 1.5.1.30) (Biliverdin reductase B) (BVR-B) (EC 1.3.1.24) (Biliverdin-IX beta-reductase) (NADPH-dependent diaphorase) (NADPH-flavin reductase) (FLR)
ATP5J_MOUSE	P97450	Atp5pf Atp5j	ATP synthase-coupling factor 6, mitochondrial (ATPase subunit F6) (ATP synthase peripheral stalk subunit F6)
ETFA_MOUSE	Q99LC5	Etfa	Electron transfer flavoprotein subunit alpha, mitochondrial (Alpha-ETF)
NDUV2_MOUSE	Q9D6J6	Ndufv2	NADH dehydrogenase [ubiquinone] flavoprotein 2, mitochondrial (EC 7.1.1.2) (NADH-ubiquinone oxidoreductase 24 kDa subunit)
VATB2_MOUSE	P62814	Atp6v1b2 Atp6b2 Vat2	V-type proton ATPase subunit B, brain isoform (V-ATPase subunit B 2) (Endomembrane proton pump 58 kDa subunit) (Vacuolar proton pump subunit B 2)
ATPB_MOUSE	P56480	Atp5f1b Atp5b	ATP synthase subunit beta, mitochondrial (EC 7.1.2.2) (ATP synthase F1 subunit beta)
QCR1_MOUSE	Q9CZ13	Uqcrc1	Cytochrome b-c1 complex subunit 1, mitochondrial (Complex III subunit 1) (Core protein I) (Ubiquinol-cytochrome-c reductase complex core protein 1)
COX5B_MOUSE	P19536	Cox5b	Cytochrome c oxidase subunit 5B, mitochondrial (Cytochrome c oxidase polypeptide Vb)
DHE3_MOUSE	P26443	Glud1 Glud	Glutamate dehydrogenase 1, mitochondrial (GDH 1) (EC 1.4.1.3)
VATE1_MOUSE	P50518	Atp6v1e1 Atp6e Atp6e2	V-type proton ATPase subunit E 1 (V-ATPase subunit E 1) (V-ATPase 31 kDa subunit) (p31) (Vacuolar proton pump subunit E 1)
NDUS1_MOUSE	Q91VD9	Ndufs1	NADH-ubiquinone oxidoreductase 75 kDa subunit, mitochondrial (EC 7.1.1.2) (Complex I-75kD) (CI-75kD)
CH60_MOUSE	P63038	Hspd1 Hsp60	60 kDa heat shock protein, mitochondrial (EC 5.6.1.7) (60 kDa chaperonin) (Chaperonin 60) (CPN60) (HSP-65) (Heat shock protein 60) (HSP-60) (Hsp60) (Mitochondrial matrix protein P1)
VDAC2_MOUSE	Q60930	Vdac2 Vdac6	Voltage-dependent anion-selective channel protein 2 (VDAC-2) (mVDAC2) (Outer mitochondrial membrane protein porin 2) (Voltage-dependent anion-selective channel protein 6) (VDAC-6) (mVDAC6)
KCRU_MOUSE	P30275	Ckmt1	Creatine kinase U-type, mitochondrial (EC 2.7.3.2) (Acidic-type mitochondrial creatine kinase) (Mia-CK) (Ubiquitous mitochondrial creatine kinase) (U-MtCK)
DYN1_MOUSE	P39053	Dnm1 Dnm Kiaa4093	Dynamin-1 (EC 3.6.5.5)
ACTZ_MOUSE	P61164	Actr1a Ctrn1	Alpha-centractin (Centractin) (ARP1) (Actin-RPV) (Centrosome-associated actin homolog)
ACTB_MOUSE	P60710	Actb	Actin, cytoplasmic 1 (Beta-actin) [Cleaved into: Actin, cytoplasmic 1, N-terminally processed]
SSDH_MOUSE	Q8BWF0	Aldh5a1	Succinate-semialdehyde dehydrogenase, mitochondrial (EC 1.2.1.24) (Aldehyde dehydrogenase family 5 member A1) (NAD(+)-dependent succinic semialdehyde dehydrogenase)
KAD4_MOUSE	Q9WUR9	Ak4 Ak-4 Ak3b Ak3l1	Adenylate kinase 4, mitochondrial (AK 4) (EC 2.7.4.10) (EC 2.7.4.6) (Adenylate kinase 3-like) (Adenylate kinase isoenzyme 4) (GTP:AMP phosphotransferase AK4)

GBB1_MOUSE	P62874	Gnb1	Guanine nucleotide-binding protein G(I)/G(S)/G(T) subunit beta-1 (Transducin beta chain 1)
ALDOA_MOUSE	P05064	Aldoa Aldo1	Fructose-bisphosphate aldolase A (EC 4.1.2.13) (Aldolase 1) (Muscle-type aldolase)
ENOG_MOUSE	P17183	Eno2 Eno-2	Gamma-enolase (EC 4.2.1.11) (2-phospho-D-glycerate hydro-lyase) (Enolase 2) (Neural enolase) (Neuron-specific enolase) (NSE)
GUAD_MOUSE	Q9R111	Gda	Guanine deaminase (Guanase) (Guanine aminase) (EC 3.5.4.3) (Guanine aminohydrolase) (GAH)
**Cortical Mitochondrial Proteins**			
IDH3A_MOUSE	Q9D6R2	Idh3a	Isocitrate dehydrogenase [NAD] subunit alpha, mitochondrial (EC 1.1.1.41) (Isocitric dehydrogenase subunit alpha) (NAD(+)-specific ICDH subunit alpha)
DHSB_MOUSE	Q9CQA3	Sdhb	Succinate dehydrogenase [ubiquinone] iron-sulfur subunit, mitochondrial (EC 1.3.5.1) (Iron-sulfur subunit of complex II) (Ip)
NSF_MOUSE	P46460	Nsf Skd2	Vesicle-fusing ATPase (EC 3.6.4.6) (N-ethylmaleimide-sensitive fusion protein) (NEM-sensitive fusion protein) (Suppressor of K(+) transport growth defect 2) (Protein SKD2) (Vesicular-fusion protein NSF)
DPYL5_MOUSE	Q9EQF6	Dpysl5 Crmp5	Dihydropyrimidinase-related protein 5 (DRP-5) (Collapsin response mediator protein 5) (CRMP-5)
GRP78_MOUSE	P20029	Hspa5 Grp78	Endoplasmic reticulum chaperone BiP (EC 3.6.4.10) (78 kDa glucose-regulated protein) (GRP-78) (Binding-immunoglobulin protein) (BiP) (Heat shock protein 70 family protein 5) (HSP70 family protein 5) (Heat shock protein family A member 5) (Immunoglobulin heavy chain-binding protein)
LASP1_MOUSE	Q61792	Lasp1 Mln50	LIM and SH3 domain protein 1 (LASP-1) (Metastatic lymph node gene 50 protein) (MLN 50)
PPIA_MOUSE	P17742	Ppia	Peptidyl-prolyl cis-trans isomerase A (PPIase A) (EC 5.2.1.8) (Cyclophilin A) (Cyclosporin A-binding protein) (Rotamase A) (SP18) [Cleaved into: Peptidyl-prolyl cis-trans isomerase A, N-terminally processed]
