# Supplementary material for: Proteomic analysis links alterations of bioenergetics, mitochondria-ER interactions and proteostasis in hippocampal astrocytes from 3xTg-AD mice
Source: Cell Death Dis. 2020 Aug 18;11(8):645. doi: 10.1038/s41419-020-02911-1 (PMC7434916; doi:10.1038/s41419-020-02911-1)
Supplement: Supplementary file 8 — Supplemental Table 3d [file 41419_2020_2911_MOESM8_ESM.pdf]

Supplementary Table 3d. Comparison of MERE fraction DEPs with DEPs of Yu et al., 2018 dataset.					
**SwissProt ID**	**Protein name**	***p*-v. (MERE fr.)**	**FC (MERE fr.)**	**FC sign (Yu)**	**Location (Yu)**
VATB2_MOUSE	V-type proton ATPase subunit B	0.00674	0.632	DOWN	Hippocampal Mitochondrial Proteins
NSF_MOUSE	Vesicle-fusing ATPase	0.01152	2.035	DOWN	Cortical Mitochondrial Proteins

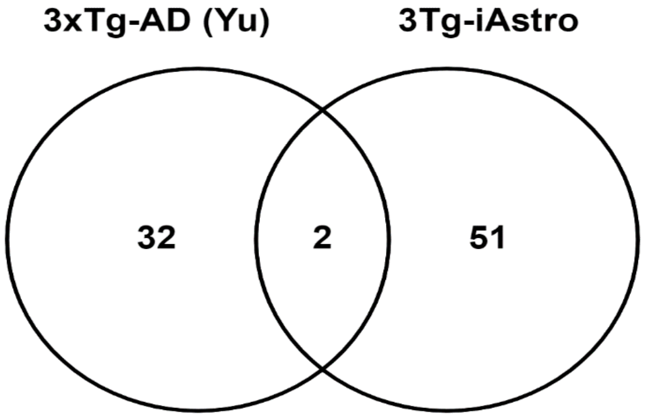

R command:: phyper(k-1, K, N-K, n, lower.tail=FALSE)

**Hypergeometric**	Volgyi ident.	Volgy quant.	MERE fraction ident. in WT
N	5957	3245	1089
K	34		
n	53		
k	2		
***p*-value**	**0.0363**	**0.1055**	**0.50086**
	0.036343998	0.105454569	0.500858575
**Expected value**	**0.302501259**	**0.555315871**	**1.654729109**
n*(K/N)			
